# Synthesis of Lactams via a Chiral Phosphoric Acid-Catalyzed Aniline Cyclization

**DOI:** 10.1021/acs.joc.4c01060

**Published:** 2024-08-09

**Authors:** Abigail H. Horchar, Jonathan E. Dean, Alexander R. Lake, Jessica E. Carsley, Tiana R. Lillevig, Shubin Liu, Kimberly S. Petersen

**Affiliations:** † Department of Chemistry and Biochemistry, The University of North Carolina at Greensboro, 301 McIver Street, Greensboro, North Carolina 27412, United States; ‡ Department of Chemistry, The University of North Carolina at Chapel Hill, 125 South Road, Chapel Hill, North Carolina 27514, United States

## Abstract

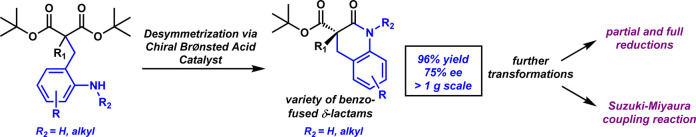

The enantioenriched lactams disclosed in this work are synthesized concisely in four steps. In the penultimate reaction, a benzylamine species complexes with a chiral phosphoric acid to produce benzo-fused δ-lactams equipped with an all-carbon quaternary stereocenter. Partial and full reductions were carried out on the ester and amide moieties, and a Suzuki–Miyaura cross-coupling expanded the molecule from the aromatic ring. Finally, our method was successful at a >1 g scale, indicating that the method has important practical use.

Increasing antimicrobial resistance and emerging diseases calls for the scientific community to uncover unique molecules and scaffolds. In this pursuit is the need for improved methods to assemble these structurally relevant motifs. One mission of synthesis is to construct molecules that emulate the functions of valuable natural products. However, a pressing problem is the ability of chemists to synthesize these complex compounds, for the sake of unlocking certain coveted properties, due to elaborate stereochemistry and functional groups. Consequently, chemists need more tools in their toolbox. This drives the development of methodologies to recreate target moieties such as *N*- and *O*-containing heterocycles—along with their specific configuration in space. Concurrently, there is considerable literature acknowledging the importance of lactams and their presence in compounds with biological applications. Moreover, works by Akiyama, List, Chen, Sun, and others detail the significance of CPA-catalyzed desymmetrizations.^1−4^ The method herein produces enantioenriched lactams with a chiral center α to the carbonyl through a desymmetrization via a chiral phosphoric acid (CPA) catalyst. The compounds in Figure 1 highlight the importance of the fused lactam motif.^5^

**Figure 1:**
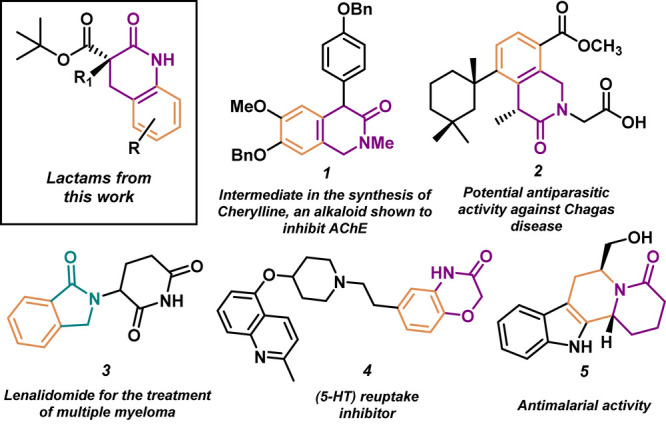
Significance of the chiral lactams produced in this work is highlighted by the featured lactam-containing compounds.

Chiral aryl lactams and their derivatives fulfill key roles as components of drugs and other bioactive natural products.^6^ Furthermore, a review by Roughly and Jordan investigated the types of reactions employed in the pursuit of drug candidates; the category titled “heterocycle formation” was dominated by *N*-containing heterocycle syntheses.^7^ Additionally, FDA databases disclose that approximately 60% of unique small-molecule drugs contain *N*-based heterocycles.^8^ Categorically, δ-lactams have received less attention as potential drugs than β- and γ-lactams;^9^ this presents an opportunity to develop more efficient methods to make them more accessible.

The synthetic strategy revealed in this work addresses a gap in desymmetrization and lactamization methodologies. While reports of lactamization techniques exist,^10−13^ metal-free enantioselective lactamization methods are scarce. Reports of transition-metal-catalyzed reactions exist that take advantage of palladium, rhodium, iridium, cobalt, nickel, and molybdenum (see entry A,^14^Scheme 1 for an example).^15−19^ Although metal-driven systems have accomplished impressive chemistry, metals are accompanied by challenges like high price, toxicity, pollution, waste treatment complications, and product contamination.^20^ Thus, it is attractive to explore organocatalytic options. Sumiyoshi et al. built on nonselective methodologies^21^ to synthesize chiral γ-lactams^22^ (entry B, Scheme 1). When compound **8** was exposed to (*S*)-TRIP (50 mol %), γ-lactams (**9**) were fashioned with ee’s ranging from 49% to 66%. While modest ee’s are observed, a major limitation is the utilization of 0.5 equiv of the chiral phosphoric acid TRIP, which is impractical on a moderate to large scale. Additionally, a limited substrate scope is explored. In entry C^23^ (Scheme 1), lactam **13** is prepared such that the stereocenter is previously established in precursor **12** before the heterocycle-forming step. While a chiral lactam is produced, its synthesis requires more than one step. Other published syntheses yield enantioenriched lactams, but the systems either are only diastereoselective (not enantioselective)^24^ or do not contain a chiral center on the lactam ring.^25^

**Scheme 1:**
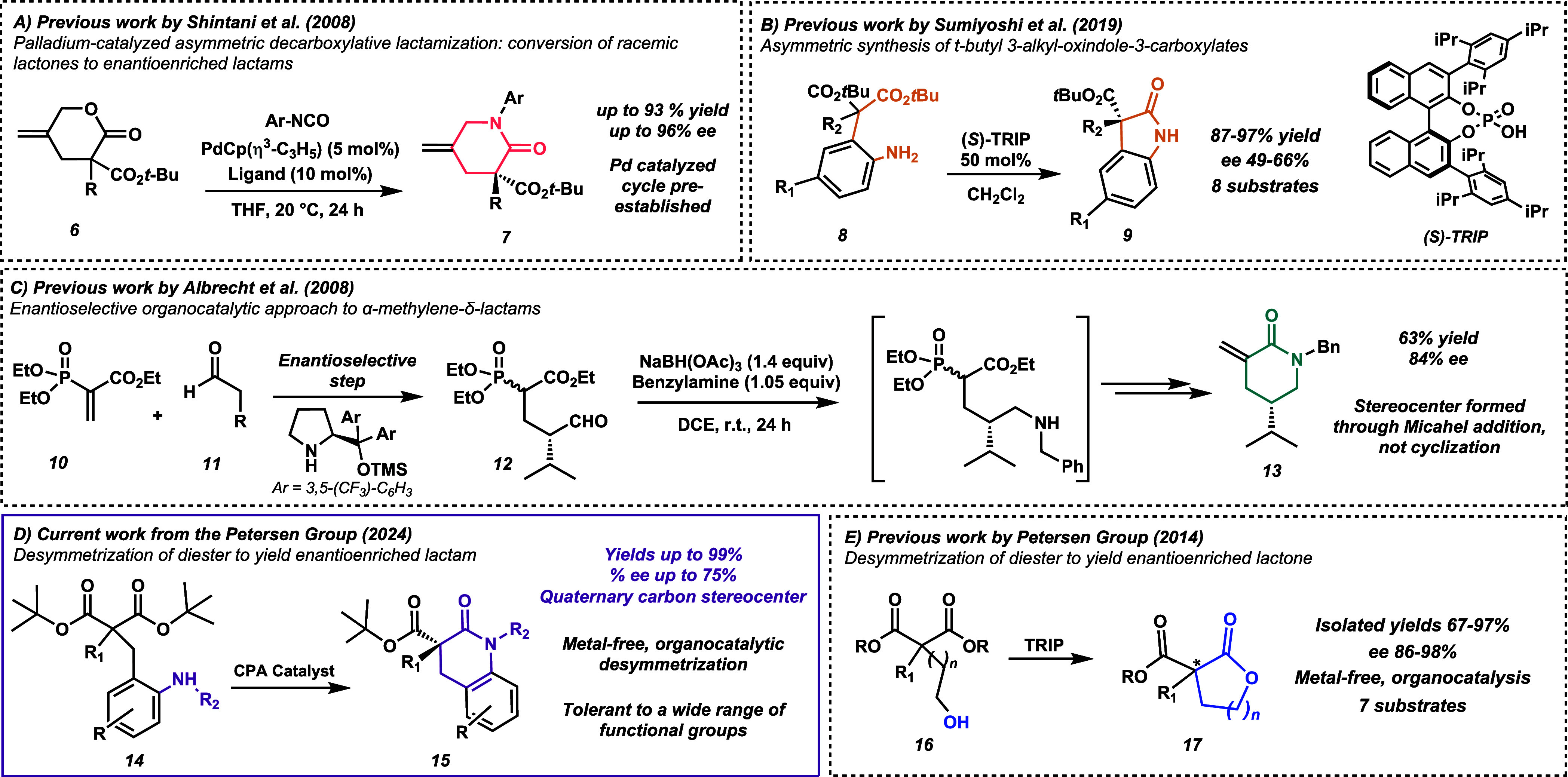
Literature Precedence, Other Lactamization Strategies, and Reaction Scheme for the Current Study TRIP = 3,3′-Bis(2,4,6-triisopropylphenyl)-1,1′-binaphthyl-2,2′-diyl hydrogenphosphate.

This study harnesses (*R*)-TRIP at only 5 mol % catalyst loading and produces δ-lactams with ee’s up to 75%. Our precursor amine **14** can be cyclized as a primary amine or secondary amine to yield lactam **15** (entry D, Scheme 1). The products contain substitution on the aromatic ring at the ortho, meta, and para positions with halogens, electron-donating groups, and electron-withdrawing groups. These enantioenriched lactams are synthesized in four steps; the concluding step is a metal-free desymmetrization catalyzed by the chiral Brønsted acid TRIP (3,3′-bis(2,4,6-triisopropylphenyl)-1,1′-binaphthyl-2,2′-diyl hydrogen phosphate), a CPA catalyst. This desymmetrization is elegant because it simultaneously forms a benzo-fused δ-lactam while establishing an all-carbon quaternary chiral center and does not rely on transition-metal catalysts or previously set chiral centers. The remaining ester handle and select substituents on the aromatic ring allow for further functionalization. Besides the readily available starting materials and straightforward reaction conditions, another attractive feature is the ability to generate a vast number of substrates through variation of the nitrobenzyl bromide species, the R group at the α-carbon, and the use of primary or secondary aniline nitrogen nucleophiles. This current desymmetrization strategy was motivated by previous work in the Petersen group on the cyclization of hydroxy-esters to form lactones **17** (entry E, Scheme 1).^26,27^ Though we draw inspiration from a previous strategy, we are excited to establish a new methodology to synthesize significantly more challenging nitrogen-based heterocycles.

## Results and Discussion

**Scheme 2:**
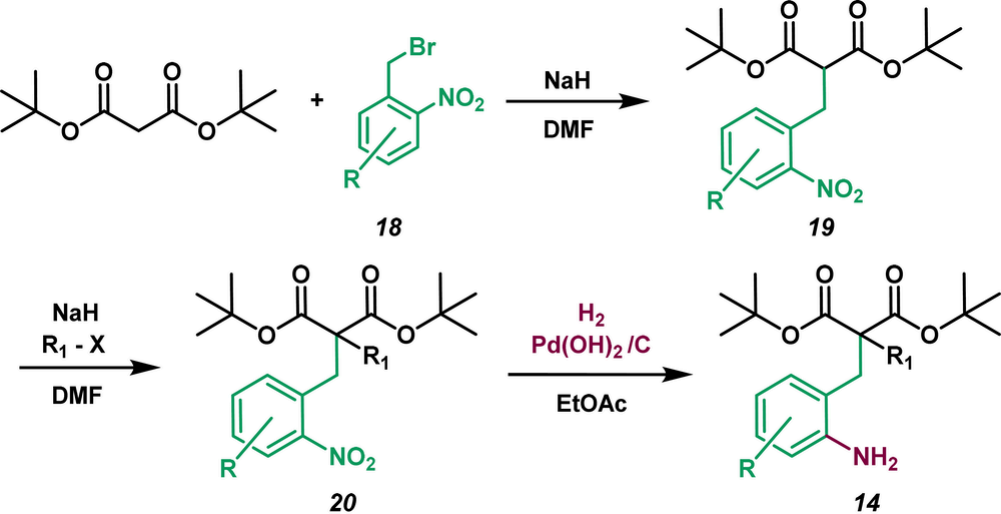
Synthetic Route to the Precursor Amines

Malonate esters provide a scaffold to conduct desymmetrization reactions in the pursuit of chiral lactams (**14** to **15**, entry D, Scheme 1). Our general strategy began with the alkylation of di-*tert*-butyl malonate with bromide **18** (Scheme 2). A second alkyl group (e.g., via methyl iodide) is added to **19**, giving the dialkylated product **20** (Scheme 2). The nitro group of **20** is hydrogenated to yield a free primary amine **14**, which is poised to undergo an intramolecular cyclization reaction at a carbonyl carbon. We hypothesize that resonance with the aromatic ring moderates the reactivity of the nitrogen, allowing it to be an ideal nucleophile for this desymmetrization. Consult the Supporting Information for compound numbering details.

**Figure 2:**
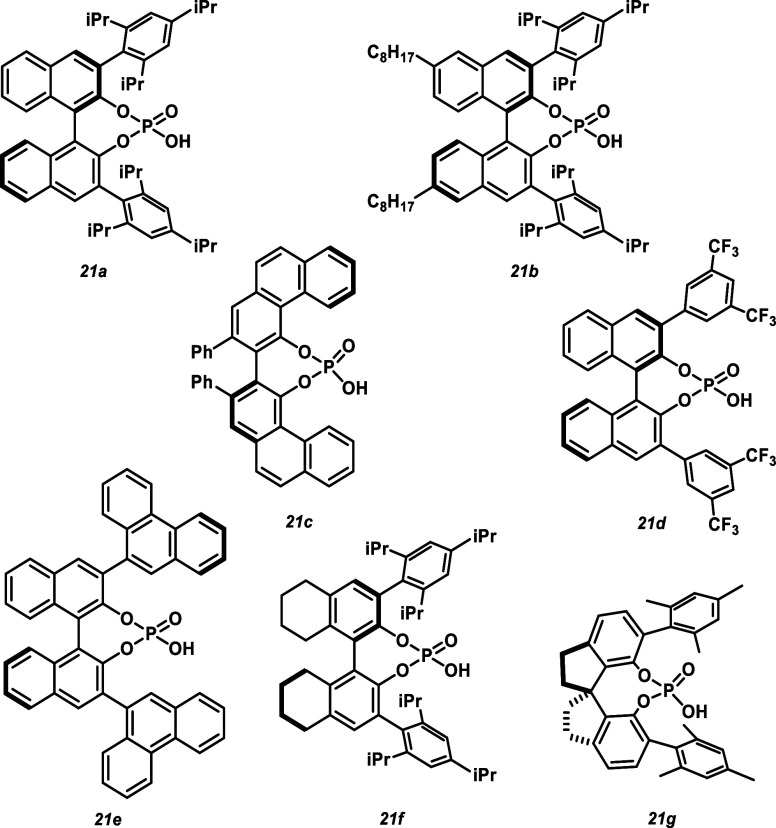
Chiral phosphoric acid catalysts tested in the optimization process.

The optimization process began with evaluating the cyclization of aniline **14aa** in the presence of various CPAs (Figure 2) to determine which CPA provided the best enantiomeric enrichment (Table 1, entries 1–7). Initial results indicated that catalyst **21a** yielded the best enantioselectivity (50% ee, entry 1, Table 1) at 10 mol % and was utilized for evaluating all subsequent reaction variables. Other catalysts such as **21c**, **21d**, and **21e** showed good reactivity but little enantioselectivity (Table 1, entries 3–5). Catalysts **21b**, **21f**, and **21g** showed some enantioenrichment of product but lacked the desired reactivity (Table 1, entries 2, 6, and 7). It is known that modifying the BINOL backbone of the CPA can tune the electronics and solubility of the catalyst.^28,29^ As seen in previous systems, this has various effects on experimental outcomes.^30,31^

**Table 1:**
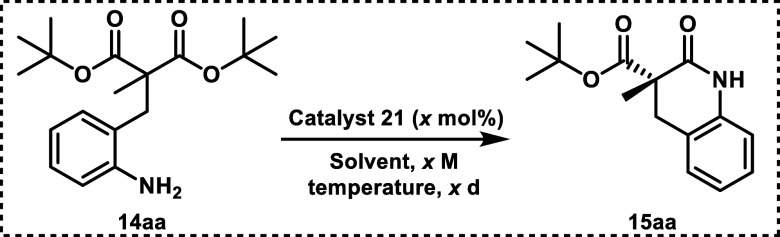
Reaction Optimization

entry	catalyst	cat. loading	solvent	concentration (M)	temperature (°C)	time (days)	% ee^c^	% yield^b^
1^a^	**21a**	10 mol %	1,2-DCE	0.025	rt	3	50	98
2	**21b**	10 mol %	1,2-DCE	0.025	rt	3	34	
3	**21c**	10 mol %	1,2-DCE	0.025	rt	3	2	89
4	**21d**	10 mol %	1,2-DCE	0.025	rt	3	7	90
5	**21e**	10 mol %	1,2-DCE	0.025	rt	3	0	34
6	**21f**	10 mol %	1,2-DCE	0.025	rt	3	28	
7	**21g**	10 mol %	1,2-DCE	0.025	rt	3	44^d^	
8	**21a**	10 mol %	hexanes	0.025	rt	3	61	99
9	**21a**	10 mol %	DCM	0.025	rt	3	55	75
10	**21a**	10 mol %	bromobenzene	0.025	rt	3	10	
11	**21a**	10 mol %	toluene	0.025	rt	3	67	93
12	**21a**	10 mol %	1,2-DCE	0.025	0	3	55	83
13	**21a**	10 mol %	1,2-DCE	0.25	rt	3	50	83
14	**21a**	10 mol %	toluene	0.25	rt	3	63	98
15	**21a**	10 mol %	toluene	0.0025	rt	3	72	99
16^e^	**21a**	5 mol %	toluene	0.0025	rt	3	73	97
17	**21a**	5 mol %	toluene	0.025	rt	3	69	97
18	**21a**	1 mol %	toluene	0.025	rt	3	68	53
19	**21a**	1 mol %	toluene	0.025	50	3	65	97
20	**21a**	1 mol %	toluene	0.0025	rt	7	66	61
21	**21a**	5 mol %	toluene, H_2_O/3 Å MS	0.025	rt	5	24	10
22	**21a**	5 mol %	toluene, 3 Å MS	0.025	rt	5	56	25
23	**21a**	5 mol %	toluene	0.025	–20	10	33	39

a Base conditions: 10 mol % of **21a**, 0.025 M in 1,2-DCE, stirring for 3 days at rt.

b qNMR yields based on ^1^H NMR analysis using 1,3,5-trimethoxybenzene as an internal standard.

c % ee values obtained via HPLC analysis.

d Opposite enantiomer was formed per HPLC analysis.

e Conditions giving the best results based first on % ee and then % yield. All optimization reactions conducted on a 10 mg scale. The full optimization table can be seen in the SI.

An examination of other nonpolar solvents (entries 8–11, Table 1) showed that toluene facilitated the best enantioenrichment, improving the ee of lactone **15aa** from 50% to 67% (compare entries 1 and 11, Table 1). Hexanes (entry 8, Table 1) also performed favorably. We hypothesize that the hydrophobicity of these two solvents drives the substrate and the catalyst into closer proximity to interact more successfully. Polar solvents were not examined here after previous work within the group determined that polar solvents interfered with the binding abilities of **21a**.^26,27^

Diluting the reaction concentration to 0.0025 M (with 10 mol % of **21a**) improved the ee to 72% while still providing a yield of 99% (entry 15, Table 1). Lowering the catalyst loading to 5 mol % and concentration to 0.0025 M provided the best ee of 73% while maintaining a 97% yield (entry 16, Table 1). While comparable ee’s were achieved with a 1 mol % catalyst loading (entries 18 and 20, Table 1), yields suffered even when the reactions were allowed to run up to 7 days. However, it was discovered that heating the reaction to 50 °C with 1 mol % **21a** enabled the reaction to run to completion with minimal decrease in ee (entry 19, Table 1). While we were able to salvage yield at 50 °C (entry 19), 5 mol % still offered better selectivity over 1 mol % (entries 16 and 17 vs entries 18–20) without requiring any heat and performed as well as 10 mol %, so we proceeded with the lower loading. Surprisingly, reducing the temperature to 0 °C (entry 12, Table 1) or subzero (entry 23, Table 1) did not improve the ee. In fact, −20 °C conditions resulted in remarkably lower ee and yield (entry 23, Table 1). The more dilute concentration afforded a slightly better ee (entries 15 and 16, Table 1). However, a 0.0025 M concentration is not convenient nor environmentally friendly on a practical scale. Therefore, while entry 16 afforded the best conditions technically (Table 1), the conditions that were used for the subsequent substrate scope were 0.025 M toluene with 5 mol % catalyst **21a** at room temperature for 3 days.

**Figure 3:**
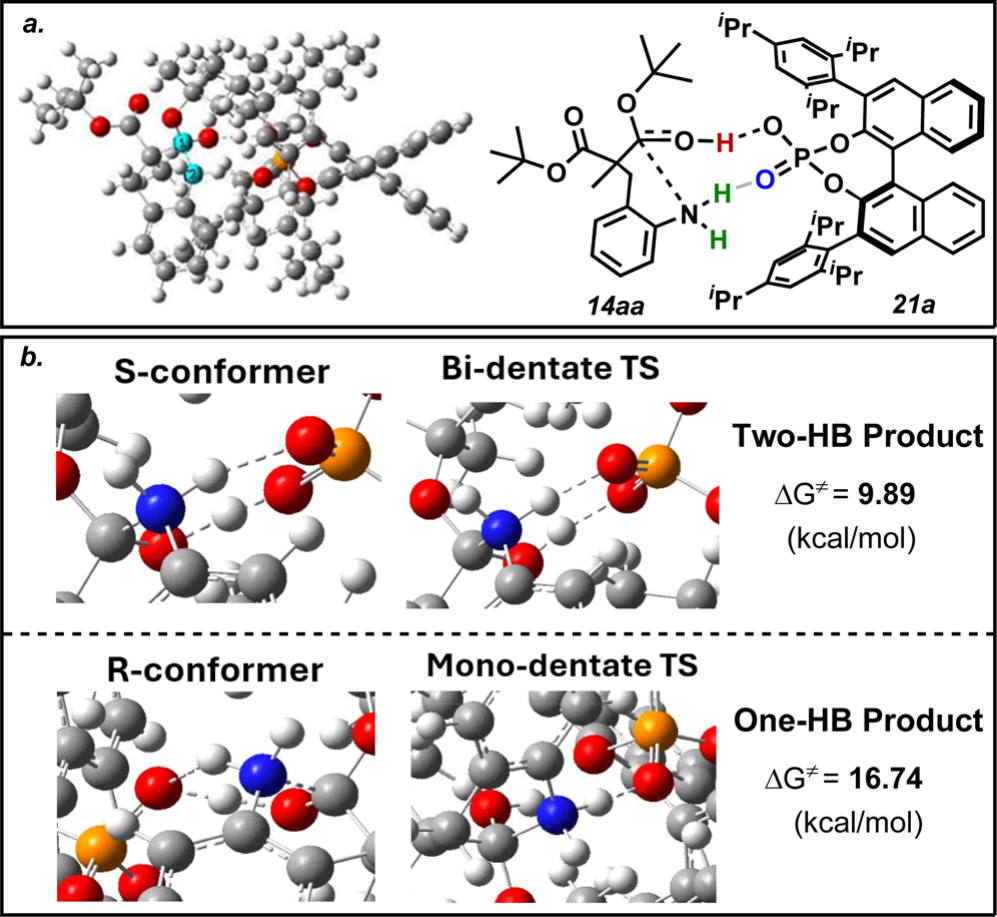
(a) DFT and skeleton models of the transition state leading to the *S* enantiomer between **21a** and **14aa**, showing the concerted formation of the C–N lactam bond and the tetrahedral intermediate. (b) DFT model showing the bidentate binding of the transition state leading to the *S* enantiomer versus the monodentate binding of the transition state leading to the *R* enantiomer

DFT studies confirm a dual-binding activation mode (Figure 3), which occurs within a small “active site” where the substrate is engulfed by the bulky triisopropylphenyl groups. The Lewis base portion of **21a** hydrogen bonds with an amine proton, while the Brønsted acid portion of **21a** hydrogen bonds with the carbonyl oxygen of one of the esters (Figure 3a). These interactions intensify the nucleophilicity of the nitrogen and the electrophilicity of the carbonyl carbon. The Brønsted acid hydrogen (red) is concertedly transferred to the carbonyl oxygen as the C–N lactam bond is established. The triisopropylphenyl groups and the axial chirality of the catalyst direct the cyclization to occur in one preferred direction. The tetrahedral intermediate of the heterocycle collapses, producing a δ-lactam with a positively charged nitrogen. Finally, the abstraction of the hydrogen on the nitrogen (green) to regenerate the CPA catalyst is barrierless (Figure 3a). More detailed DFT calculations were conducted to examine the C–N bond-forming step for both enantiomers. Formation of the *S* enantiomer of **15aa** follows a bidentate route, while the *R* enantiomer takes a monodentate route, leading to barrier heights of 9.89 and 16.74 kcal/mol for *S* and *R*, respectively (Figure 3b). These calculations demonstrate how the cyclization for the *S* enantiomer follows a more energetically favorable pathway than the *R* enantiomer. Full coordinates of this process are included in the Supporting Information. These data are consistent with previous DFT studies on similar desymmetrizations and kinetic resolutions.^32^ All calculations were performed with the Gaussian 16 C01 package with ultrafine grids and tight self-consistent-field convergence.^33^ Structures were optimized using the DFT M06-2X exchange-correlation energy density functional.^34^ The basis set was triple-ζ 6-311+G(d) for N, O, and P elements and 6-31G(d) for C and H elements.^35^ Toluene was the solvent included in the calculations with the CPCM^36^ implicit solvent model employed.

**Figure 4:**
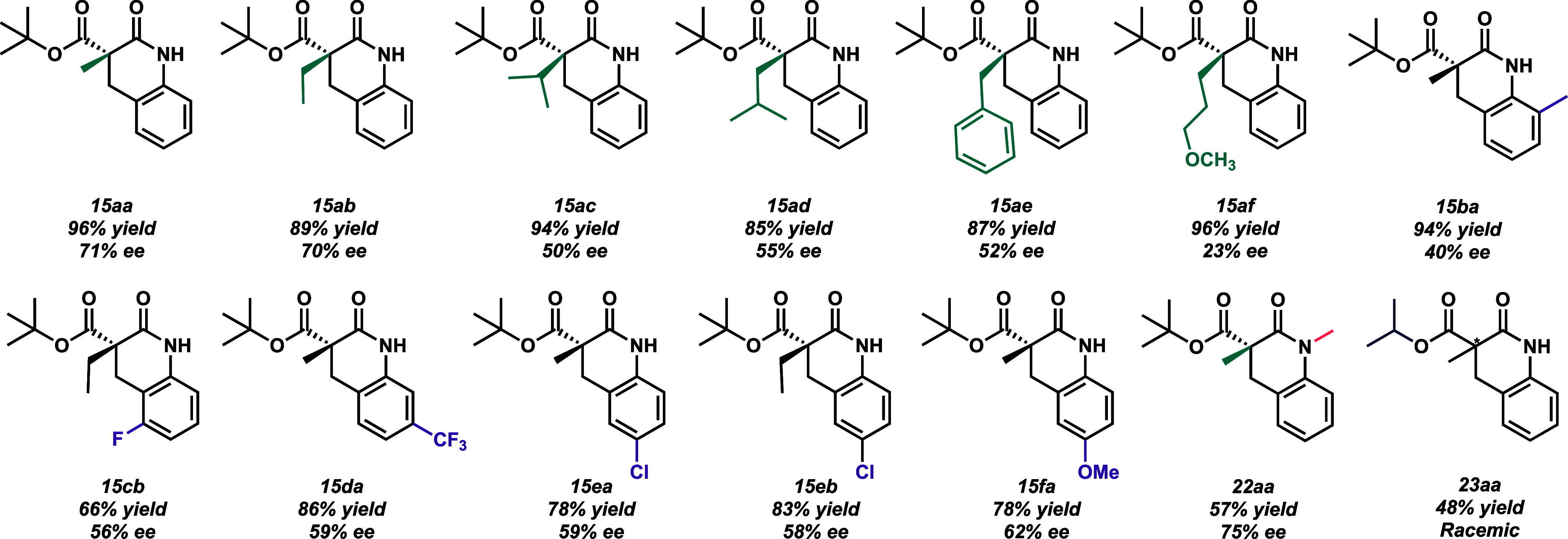
Scope of lactam substrates produced. Consult the Supporting Information for more detail.

We embarked on the substrate scope with the intent of showcasing the method’s versatility. Various alkylating groups and aryl electron-withdrawing groups and electron-donating groups were tested to determine what complimented or inhibited the system (Figure 4). The reactivity and resultant ee’s were not significantly impacted by the electronics of the various groups on the aniline ring. Substituents at the meta and para positions to the amine on the aromatic ring do not affect the efficacy of the reaction (**15cb–15fa**, Figure 4). We hypothesize that substrate **15ba** suffered lower enantioenrichment due to steric interference of the *ortho*-methyl group on the amine.

Generally, any alkyl moiety at the α-carbon was well tolerated. Bulkier groups like an isopropyl or *sec*-butyl (**15ac** and **15ad**, Figure 4) can be attached without significant negative impacts being observed. The presence of the heteroatom in the alkylating chain (at the α-carbon) resulted in a significantly lower ee (23%), while a high yield (96%) was retained (**15af**). We theorize that the oxygen atom interrupts the hydrogen bonding network between catalyst and substrate. We validated that the di-*tert*-butyl esters were optimal for cyclization by preparing the less bulky diisopropyl ester. Upon hydrogenating the dialkylated diisopropyl malonate to the amine, a sizable portion cyclized spontaneously in situ to form the racemic lactam (**23aa**). Uncyclized amine underwent spontaneous cyclization, even when material was stored at 4–5 °C. We then prepared and cyclized secondary amines to manufacture lactams with a methylated nitrogen (Figure 4, compound **22aa**). The synthetic route to the secondary amines was analogous to that of the primary amines. We were excited to produce **22aa** with 75% ee from a secondary amine subjected to 5 mol % **21a**. Consult Scheme S1 in the SI for the full synthetic route.

The absolute configuration of lactam **15aa** was determined to be *S* via X-ray crystallography. All other lactams were assigned based on analogy. Consult the SI for details on the crystal growth method.

Moreover, we can show that recrystallization of lactams leads to improved enantioenrichment with satisfactory recovery of mass. Several recrystallization attempts were conducted (entries 1–6, Table 2) before obtaining crystals with 87% ee in 60% recovery (entry 6, Table 2). The enantioselective recrystallization was an exercise in balance; some entries provided excellent ee with very poor recovery (entry 3), while others had good recovery but only marginally improved the ee (entry 4). Fortunately, in all cases, we could reconcentrate the mother liquor to recover all of the material to attempt a better recrystallization. We felt that 0.43 g of crystals with 87% ee was satisfactory. These results do show, however, that it is possible to obtain enantiomerically pure crystals.

**Table 2:** Summary of Results from Recrystallization Study

entry	starting mass (g)	starting ee	recovered mass (g)	% recovery	ee of recrystallization
1	0.76	64%	0.17	23%	91%
2	0.74	64%	0.48	65%	72%
3	0.73	64%	0.03	3.5%	98%
4	0.73	64%	0.66	91%	70%
5	0.72	64%	0.49	68%	78%
6	0.72	64%	0.43	60%	87%

**Scheme 3:**
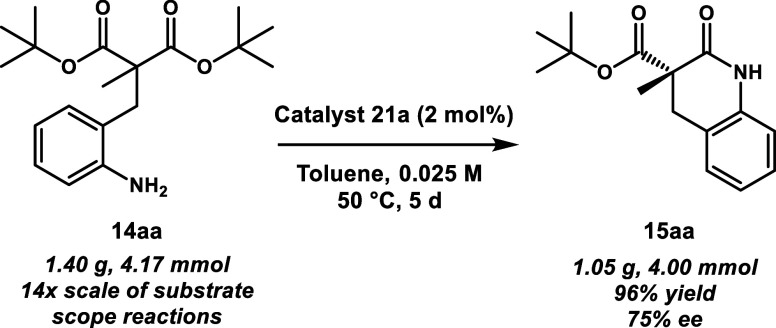
Large-Scale Cyclization Using Decreased Catalyst Loading

Additionally, while the reactions for the substrate scope (Figure 4) were performed on a 100 mg scale with 5 mol % **21a**, our method can be adapted to larger scale (>1 g) with lower catalyst loading (2 mol % **21a**). The model substrate **14aa** was subjected to a cyclization in toluene at 0.025 M at 50 °C with 2 mol % **21a**. This large-scale lactamization gave **15aa** in 96% yield and 75% ee (1.05 g, 4.00 mmol) from 1.40 g (4.17 mmol) of **14aa**, as depicted in Scheme 3.

**Scheme 4:**
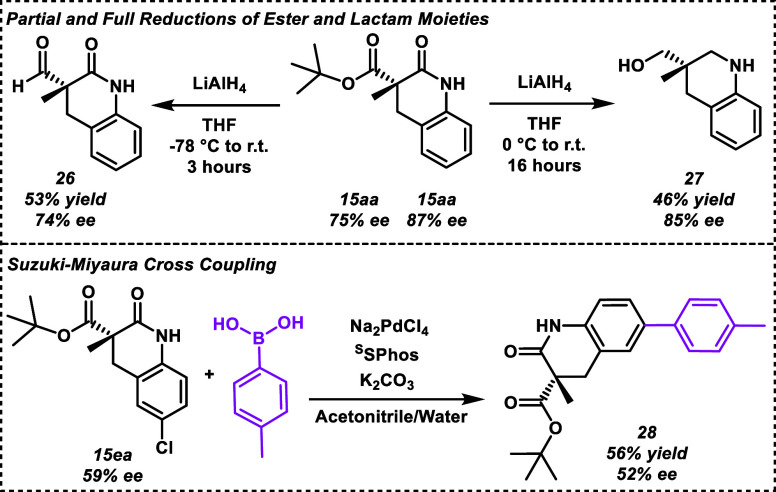
Substrates Can Be Further Manipulated To Increase Complexity and Adjust Chemical Properties

To demonstrate the applicability and practical usefulness of the chiral lactam products, we have conducted further manipulations on select substrates (Scheme 4). These transformations are relevant because they can expand the molecule so that it fits the needs of specific targets or can emulate valuable bioactive motifs. The remaining ester moiety in compound **15aa** can be reduced to yield an aldehyde **26**, or the lactam can be further reduced to give a 6-membered amine heterocycle **27**. Alternatively, incorporation of a halogen in the aniline ring as seen in substrates **15ea** and **15eb** allows for potential coupling reactions. Highlighting this use, we were able to perform a Suzuki–Miyaura coupling^37^ of **15ea** and 4-tolylboronic acid to produce **28** in 56% yield as shown in Scheme 4.

## Conclusion

We have reported an elegant methodology to synthesize benzo-fused δ-lactams in yields up to 96% and enantiomeric excess up to 75% through the desymmetrization of disubstituted malonic esters in the presence of a chiral Brønsted acid organocatalyst. Benzo-fused δ-lactams are seen in an array of valuable compounds; accessing this motif through our methodology will significantly aid in the ability to synthesize these compounds or necessary analogues. To the best of our knowledge, this is the first enantioselective Brønsted acid-catalyzed lactamization resulting in benzo-fused δ-lactams. Properly harnessing nitrogen as a nucleophile is challenging; nitrogen can be nonreactive, too basic (deprotonating the catalyst and killing reaction), or too nucleophilic (attacking the carbonyl too rapidly and foregoing selectivity). We found that an aniline nitrogen maintained a satisfactory balance between nucleophilicity and reactivity. The substrate scope allows for diversification in structure, which lends to variation in how each lactam can be subsequently utilized. Both primary and secondary amines can successfully complex with TRIP to produce enantioenriched δ-lactams and *N*-methylated δ-lactams. The substrates can be further manipulated via reductions or a coupling reaction. This lactamization strategy can also be scaled up; merely 2 mol % **21a** can achieve 75% ee and 96% yield on a >1 g scale. We expect that the work reported will provide a facile and efficient means for chemists to produce desired molecules.

## Experimental Details

### General Methods

Unless noted, all solvents and reagents were obtained from commercial sources and used without further purification; anhydrous solvents were dried following standard procedures. Compounds **18a**–**18e** were purchased from a chemical supplier (1PlusChem, TCI, Sigma-Aldrich, or Chemcia Scientific, LLC); compound **18f** was synthesized by brominating 5-methoxy-2-nitrotoluene (please consult the Supporting Information for a full compound numbering guide). Purity and identity analysis was conducted on compound **18b**. The ^1^H and ^13^C (proton decoupled) nuclear magnetic resonance (NMR) spectra were plotted on a 400 MHz spectrometer using CDCl_3_ and acetone-*d*_6_ as solvents at room temperature. The NMR chemical shifts (δ) are reported in parts per million. Abbreviations for ^1^H NMR: s = singlet, d = doublet, m = multiplet, br s = broad singlet, t = triplet, q = quartet, sept = septet, hept = heptet, dd = doublet of doublets, dt = doublet of triplets, td = triplet of doublets, dq = doublet of quartets. The reactions were monitored by TLC using silica G F254 precoated plates. Flash chromatography was performed using flash-grade silica gel (particle size 40–63 μm, 230 × 400 mesh). Enantiomeric excess was determined by HPLC analysis. High-resolution mass spectra were acquired on an Orbitrap XL MS system. The specific rotations were acquired on an analytical polarimeter.

### General Method for Quantitative NMR

^1^H NMR was used to quantify yields for the optimization table reactions (10 mg scale). After the cyclization reaction of amine **14aa** with catalyst **21a**, the reaction mixture was concentrated. Crude lactam **15aa** was diluted with CDCl_3_ and added to an NMR tube. An internal standard solution of 1,3,5-trimethoxybenzene in CDCl_3_ was prepared. A known amount of internal standard solution was added to the NMR tube containing product **15aa**. The most shielded aromatic peak of **15aa** was compared to the aromatic singlet of the internal standard. Stoichiometric calculations in an Excel spreadsheet revealed the percent yield based on the integrations of the peaks, how many protons each peak represented, and theoretical yield for each specific reaction and spectrum.

#### Compound **18b**

To verify the identity and purity of the brominated nitrotoluene species purchased commercially (Chemcia Scientific, LLC; CAS no. 55324-02-2), **18b** was characterized according to ^1^H NMR, ^13^C{^1^H} NMR, HRMS, and melting point. ^1^H NMR (400 MHz, CDCl_3_) δ 7.35 (m, 2H), 7.26 (m, 1H), 4.46 (s, 2H), 2.34 (s, 3H); ^13^C{^1^H} NMR (100 MHz, CDCl_3_) δ 150.7, 132.0, 131.0, 130.8, 129.8, 129.1, 27.0, 17.8; mp = 55.2–56.5 °C. HRMS (ESI) *m*/*z*: [M + H]^+^ calcd 229.9811 for C_8_H_8_BrNO_2_; found 229.9818.

#### Compound **18f**

To a flame-dried flask were added 5-methoxy-2-nitrotoluene (3.1 g, 18 mmol) and *n*-bromosuccinimide (3.6 g, 20 mmol) under argon. Benzene (50 mL) was then added to the flask, followed by azobisisobutyronitrile (0.45 g, 2.7 mmol). The solution was stirred at 90 °C for 4 h with a reflux condenser attached. The reaction was cooled to room temperature and filtered through a silica plug with excess hexanes. The organic material was concentrated. The residue was purified by flash chromatography on silica gel (5% → 100% EtOAc in hexanes) to afford compound **18f** as a light-yellow oil (1.4 g, 31% yield). ^1^H NMR (400 MHz, CDCl_3_) δ 8.15 (d, *J* = 9.3 Hz, 1H), 7.02 (d, *J* = 2.8 Hz, 1H), 6.92 (dd, *J* = 9.1, 2.8 Hz, 1H), 4.86 (s, 2H), 3.92 (s, 3H); ^13^C NMR (100 MHz, CDCl_3_) δ 163.6, 140.8, 135.9, 128.6, 117.7, 114.2, 56.2, 30.1. Characterization data matches previously reported data.^38^

#### Compound **19a**

To a solution of sodium hydride (60% dispersion in mineral oil, 0.57 g, 14 mmol) in dimethylformamide (30 mL) in a flame-dried flask was added di-*tert*-butyl malonate (2.7 mL, 12 mmol) at 0 °C under argon. The solution was stirred for several minutes to allow for gas evolution. To the solution at 0 °C was added **18a** (2.5 g, 11 mmol). The solution was warmed to 21 °C, transferred to an oil bath set to 40 °C, and stirred for 16 h. The reaction was quenched with 30 mL of a 1:1 ratio of ammonium chloride and water and was extracted with ethyl acetate (3 × 20 mL). The combined organic phases were dried over MgSO_4_ and concentrated. The residue was purified by flash chromatography on silica gel (5% → 100% EtOAc in hexanes) to afford compound **19a** as a light yellow oil (3.8 g, 93% yield). ^1^H NMR (400 MHz, CDCl_3_) δ 7.95 (d, *J* = 8.2 Hz, 1H), 7.48 (t, *J* = 7.5 Hz, 1H), 7.36 (t, *J* = 7.2 Hz, 2 H), 3.62 (t, *J* = 7.8 Hz, 1H), 3.39 (d, *J* = 8.0 Hz, 2H), 1.36 (s, 18H); ^13^C{^1^H} NMR (100 MHz, CDCl_3_) δ 168.0, 149.3, 133.7, 133.3, 133.2, 128.1, 125.2, 81.9, 53.9, 32.2, 27.9. HRMS (ESI) *m*/*z*: [M + H]^+^ calcd 352.1755 for C_18_H_25_NO_6_; found 352.1713.

#### Compound **19b**

To a solution of sodium hydride (60% dispersion in mineral oil, 0.17 g, 4.4 mmol) in dimethylformamide (8 mL) in a flame-dried flask was added di-*tert*-butyl malonate (0.52 mL, 2.4 mmol) at 0 °C under argon. The solution was stirred for several minutes to allow for gas evolution. To the solution at 0 °C was added **18b** (0.49 g, 2.2 mmol). The solution was warmed to 21 °C, transferred to an oil bath set to 40 °C, and stirred for 16 h. The reaction was quenched with 8 mL of a 1:1 ammonium chloride and water solution and was extracted with ethyl acetate (3 × 20 mL). The combined organic phases were dried over MgSO_4_ and concentrated. The residue was purified by flash chromatography on silica gel (5% → 100% EtOAc in hexanes) to afford compound **19b** as a light yellow oil (0.72 g, 91% yield). ^1^H NMR (400 MHz, CDCl_3_) δ 7.26 (m, 1H), 7.16 (m, 2H), 3.51 (t, *J* = 7.8 Hz, 1H), 3.09 (d, *J* = 7.8 Hz, 2H), 2.30 (s, 3 H), 1.40 (s, 18 H); ^13^C{^1^H} NMR (100 MHz, CDCl_3_) δ 167.8, 151.9, 147.0, 130.1, 130.02, 129.95, 128.8, 82.1, 54.2, 30.2, 27.9, 17.7. HRMS (ESI) *m*/*z*: [M + Na]^+^ calcd 388.1730 for C_19_H_27_NO_6_Na; found 388.1724.

#### Compound **19c**

To a solution of sodium hydride (60% dispersion, 170 mg, 4.3 mmol) in DMF (7 mL) in a flame-dried round-bottom flask was added di-*tert*-butyl malonate (0.49 mL, 2.4 mmol) at 0 °C under argon. After the ceasing of gas evolution, **18c** was added (500 mg, 2.1 mmol). The solution was warmed to 21 °C, transferred to an oil bath set to 40 °C, and stirred for 16 h. The reaction was quenched with 8 mL of a 1:1 10% HCl and water solution and was extracted with ethyl acetate (3 × 20 mL). The combined organic layers were dried over MgSO_4_, filtered, and concentrated in vacuo. The oil residue was purified by flash chromatography on silica gel (20% → 100% EtOAc in hexanes) to afford compound **19c** as a light-yellow oil (740 mg, 94% yield). ^1^H NMR (400 MHz, CDCl_3_) δ 7.75 (d, *J* = 8.0 Hz, 1H), 7.33 (m, 1H), 3.55 (t, *J* = 7.7 Hz, 1H), 3.46 (d, *J* = 7.9 Hz, 2H), 1.38 (s, 18H); ^13^C{^1^H} NMR (100 MHz, CDCl_3_) δ 167.6, 160.3, 128.5, 122.1, 120.7, 120.3, 81.8, 53.1, 27.8, 23.8; ^19^F NMR (376 MHz, CDCl_3_) δ −110.0 ppm. HRMS (ESI) *m*/*z*: [M + H]^+^ calcd 370.1660 for C_18_H_24_FNO_6_; found 370.1656.

#### Compound **19e**

To a solution of sodium hydride (60% dispersion, 320 mg, 7.9 mmol) in DMF (14 mL) in a flame-dried round-bottom flask was added di-*tert*-butyl malonate (0.96 mL, 4.4 mmol) at 0 °C under argon. The solution was stirred for several minutes to allow for gas evolution. To the solution at 0 °C was added **18e** (1.0 g, 3.9 mmol). The solution was warmed to 21 °C, transferred to an oil bath set to 40 °C, and stirred for 16 h. The reaction was quenched with 14 mL of a 1:1 ammonium chloride and water solution and was extracted with ethyl acetate (3 × 20 mL). The combined organic layers were dried over MgSO_4_, filtered, and concentrated in vacuo. The residue was purified by flash chromatography on silica gel (5% → 100% EtOAc in hexanes) to afford compound **19e** as a white, fluffy solid (1.2 g, 78% yield). ^1^H NMR (400 MHz, CDCl_3_) δ 7.96 (d, *J* = 8.6 Hz, 1H), 7.38 (d, *J* = 2.2 Hz, 1H), 7.35 (dd, *J* = 8.5, 2.4 Hz, 1H), 3.62 (t, *J* = 7.8 Hz, 1H), 3.38 (d, *J* = 7.6 Hz, 2H), 1.40 (s, 18H); ^13^C{^1^H} NMR (100 MHz, CDCl_3_) δ 167.7, 147.4, 139.5, 135.8, 133.1, 128.2, 126.7, 82.2, 53.7, 32.1, 27.9; mp = 74.1–76.6 °C. HRMS (ESI) *m*/*z*: [M + Na]^+^ calcd 408.1184 for C_18_H_24_ClNO_6_Na; found 408.1181.

#### Compound **31a**

To a solution of sodium hydride (60% dispersion, 560 mg, 14 mmol) in DMF (24 mL) in a flame-dried round-bottom flask was added diisopropyl malonate (1.7 mL, 8.7 mmol) at 0 °C under argon. The solution was stirred for several minutes to allow for gas evolution. To the solution at 0 °C was added **18a** (1.5 g, 6.9 mmol). The solution was warmed to 21 °C, transferred to an oil bath set to 40 °C, and stirred for 16 h. The reaction was quenched with 24 mL of a 1:1 ammonium chloride and water solution and was extracted with ethyl acetate (3 × 20 mL). The combined organic layers were dried over MgSO_4_, filtered, and concentrated in vacuo. The residue was purified by flash chromatography on silica gel (5% → 100% EtOAc in hexanes) to afford compound **31a** as a light-yellow oil (1.9 g, 85% yield). ^1^H NMR (400 MHz, CDCl_3_) δ 7.94 (d, *J* = 8.2 Hz, 1H), 7.47 (t, *J* = 7.4 Hz, 1H), 7.36 (t, *J* = 7.4 Hz, 2H), 4.95 (sept, *J* = 6.4 Hz, 2H), 3.74 (t, *J* = 7.8 Hz, 1H), 3.43 (d, *J* = 7.7 Hz, 2H), 1.16 (d, *J* = 6.3 Hz, 6H), 1.10 (d, *J* = 6.4 Hz, 6H); ^13^C NMR (100 MHz, CDCl_3_) δ 168.2, 149.2, 133.3, 133.3, 133.2, 128.2, 125.2, 69.3, 52.6, 32.1, 21.6. HRMS (ESI) *m*/*z*: [M + H]^+^ calcd 324.1439 for C_16_H_21_NO_6_; found 324.1442.

#### Compound **20da**

To a solution of sodium hydride (60% dispersion, 140 mg, 3.5 mmol) in dry THF (10 mL) were slowly added di-*tert*-butyl 2-methylmalonate (0.45 mL, 1.9 mmol) followed by **18d** (500 mg, 1.7 mmol) at 0 °C. The reaction was allowed to warm to 21 °C and stirred for 16 h under argon. The reaction was cooled to 0 °C, quenched with 10 mL of a 1:1 10% HCl and water solution, and extracted with ethyl acetate (3 × 10 mL). The combined organic layers were dried over MgSO_4_, filtered, and concentrated in vacuo. The crude oil residue was purified by flash chromatography on silica gel (1% → 15% EtOAc in hexanes) to afford compound **20da** as a colorless oil (490 mg, 64% yield). ^1^H NMR (400 MHz, CDCl_3_) δ 8.12 (s, 1H), 7.72 (d, *J* = 8.24 Hz, 1H), 7.65 (d, *J* = 8.24 Hz, 1H), 3.61 (s, 2H), 1.41 (s, 18H), 1.26 (s, 3H) ppm; ^13^C{^1^H} NMR (100 MHz, CDCl_3_) δ 170.6, 150.7, 136.6, 134.3, 130.5 (q, *J* = 34.5 Hz), 128.7, 124.3, 122.0, 82.2, 56.1, 35.9, 28.0, 20.5 ppm; ^19^F NMR (376 MHz, CDCl_3_) δ 62.8 ppm. HRMS (ESI) *m*/*z*: [M + H]^+^ calcd 434.1785 for C_20_H_26_F_3_NO_6_; found 434.1791.

#### Compound **20fa**

To a solution of sodium hydride (60% dispersion, 350 mg, 8.6 mmol) in dry THF (35 mL) were slowly added di-*tert*-butyl 2-methylmalonate (1.6 g, 6.8 mmol) followed by **18f** (1.4 g, 5.7 mmol) at 0 °C. The reaction was allowed to warm to 21 °C and stirred for 16 h under argon. The reaction was cooled to 0 °C, quenched with 30 mL of a 1:1 1 M HCl and water solution, and extracted with ethyl acetate (3 × 20 mL). The combined organic layers were dried over MgSO_4_, filtered, and concentrated in vacuo. The crude oil residue was purified by flash chromatography on silica gel (5% → 15% EtOAc in hexanes) to afford compound **20fa** as a white powdery solid (1.2 g, 53% yield). ^1^H NMR (400 MHz, CDCl_3_) δ 7.97 (d, *J* = 9.2 Hz, 1H), 6.94 (d, *J* = 2.8 Hz, 1H), 6.82 (dd, *J* = 9.2, 2.8 Hz, 1H), 3.84 (s, 3H), 3.65 (s, 2H), 1.42 (s, 18H), 1.25 (s, 3H) ppm; ^13^C{^1^H} NMR (100 MHz, CDCl_3_) δ 171.0, 162.6, 143.8, 135.6, 127.6, 118.4, 112.5, 81.8, 56.3, 55.9, 36.3, 27.9, 20.1 ppm; mp = 67.2–68.4 °C. HRMS (ESI) *m*/*z*: [M + H]^+^ calcd 396.2017 for C_20_H_29_NO_7_; found 396.2007.

#### Compound **20aa**

To a flame-dried round-bottom flask, a solution of sodium hydride (60% dispersion, 370 mg, 9.2 mmol) in DMF (12 mL) was made. Compound **19a** (1.6 g, 4.6 mmol) was then added slowly at 0 °C. After the ceasing of gas evolution, methyl iodide was added (0.36 mL, 5.8 mmol). The reaction was stirred at 40 °C under argon for 16 h. The reaction was quenched with 12 mL of a 1:1 10% HCl and water solution and was extracted with ethyl acetate (3 × 15 mL). The combined organic layers were dried over MgSO_4_, filtered, and concentrated in vacuo. The oil residue was purified by flash chromatography on silica gel (5% → 25% EtOAc in hexanes) to afford compound **20aa** as a clear, pale-yellow oil (1.5 g, 89% yield). ^1^H NMR (400 MHz, CDCl_3_) δ 7.82 (d, *J* = 8.0 Hz, 1H), 7.44 (m, 2H), 7.35 (t, *J* = 7.4 Hz, 1H), 3.58 (s, 2H), 1.41 (s, 18H), 1.21 (s, 3H); ^13^C{^1^H} NMR (100 MHz, CDCl_3_) δ 170.8, 151.0, 133.1, 132.3, 132.1, 127.8, 124.7, 81.2, 56.1, 35.7, 27.9, 20.0. HRMS (ESI) *m*/*z*: [M + Na]^+^ calcd 388.1730 for C_19_H_27_NO_6_Na; found 388.1724.

#### Compound **20ab**

To a flame-dried round-bottom flask, a solution of sodium hydride (60% dispersion, 110 mg, 7.8 mmol) in DMF (13 mL) was made. Compound **19a** (1.5 g, 4.3 mmol) was then added slowly at 0 °C. After the ceasing of gas evolution, ethyl iodide was added (1.2 mL, 3.9 mmol). The reaction was stirred at 40 °C under argon for 16 h. The reaction was quenched with 14 mL of a 1:1 10% HCl and water solution and was extracted with ethyl acetate (3 × 15 mL). The combined organic layers were dried over MgSO_4_, filtered, and concentrated in vacuo. The oil residue was purified by flash chromatography on silica gel (5% → 25% EtOAc in hexanes) to afford compound **20ab** as a light-yellow powder (1.4 g, 92% yield). **20ab** was taken on to the next step without characterization (see compound 1**4ab**).

#### Compound **20ac**

To a flame-dried round-bottom flask, a solution of sodium hydride (60% dispersion, 320 mg, 7.9 mmol) in THF (30 mL) was made. Compound **19a** (2.2 g, 6.3 mmol) was then added slowly at 0 °C. After the ceasing of gas evolution, 2-iodopropane was added (1.3 mL, 13 mmol). The reaction was stirred at 40 °C under argon for 16 h. The reaction was quenched with 30 mL of a 1:1 10% HCl and water solution and was extracted with ethyl acetate (3 × 15 mL). The combined organic layers were dried over MgSO_4_, filtered, and concentrated in vacuo. The oil residue was purified by flash chromatography on silica gel (5% → 25% EtOAc in hexanes) to afford compound **20ac** as a clear, pale-yellow oil (1.7 g, 69% yield). **20ac** was taken on to the next step without characterization (see compound 1**4ac**).

#### Compound **20ad**

To a flame-dried round-bottom flask, a solution of sodium hydride (60% dispersion, 290 mg, 6.3 mmol) in DMF (20 mL) was made. Compound **19a** (1.1 g, 3.1 mmol) was then added slowly at 0 °C. After the ceasing of gas evolution, 1-bromo-2-methylpropane was added (0.60 mL, 3.8 mmol). The reaction was stirred at 40 °C under argon for 16 h. The reaction was quenched with 20 mL of a 1:1 10% HCl and water solution and was extracted with ethyl acetate (3 × 15 mL). The combined organic layers were dried over MgSO_4_, filtered, and concentrated in vacuo. The oil residue was purified by flash chromatography on silica gel (2% → 20% EtOAc in hexanes) to afford compound **20ad** as a pale-yellow oil (780 mg, 61% yield). **20ad** was taken on to the next step without characterization (see compound 1**4ad**).

#### Compound **20ae**

To a flame-dried round-bottom flask, a solution of sodium hydride (60% dispersion, 310 mg, 7.7 mmol) in DMF (13 mL) was made. Compound **19a** (1.3 g, 3.9 mmol) was then added slowly at 0 °C. After the ceasing of gas evolution, benzyl bromide was added (0.55 mL, 4.6 mmol). The reaction was stirred at 40 °C under argon for 16 h. The reaction was quenched with 14 mL of a 1:1 10% HCl and water solution and was extracted with ethyl acetate three times (3 × 15 mL). The combined organic layers were dried over MgSO_4_, filtered and concentrated in vacuo. The oil residue was purified by flash chromatography on silica gel (5% → 25% EtOAc in hexanes) to afford compound **20ae** as a clear, yellow oil (1.4 g, 87% yield). **20ae** was taken on to the next step without characterization (see compound 1**4ae**).

#### Compound **20af**

To a flame-dried round-bottom flask, a solution of sodium hydride (60% dispersion, 230 mg, 5.7 mmol) in DMF (10 mL) was made. Compound **19a** (1.0 g, 2.9 mmol) was then added slowly at 0 °C. After the ceasing of gas evolution, 1-bromo-3-methoxypropane was added (0.35 mL, 3.1 mmol). The reaction was stirred at 40 °C under argon gas for 16 h. The reaction was quenched with 10 mL of a 1:1 10% HCl and water solution and was extracted with ethyl acetate (3 × 10 mL). The combined organic layers were dried over MgSO_4_, filtered, and concentrated in vacuo. The oil residue was purified by flash chromatography on silica gel (5% → 25% EtOAc in hexanes) to afford compound **20af** as a yellow oil (1.1 g, 90% yield). **20af** was taken on to the next step without characterization (see compound 1**4af**).

#### Compound **20ba**

To a flame-dried round-bottom flask, a solution of sodium hydride (60% dispersion, 160 mg, 3.9 mmol) in DMF (10 mL) was made. Compound **19b** (710 mg, 1.9 mmol) was then added slowly at 0 °C. After the ceasing of gas evolution, methyl iodide was added (0.13 mL, 2.1 mmol). The reaction was stirred at 40 °C under argon for 16 h. The reaction was quenched with 10 mL of a 1:1 10% HCl and water solution and was extracted with ethyl acetate (3 × 10 mL). The combined organic layers were dried over MgSO_4_, filtered, and concentrated in vacuo. The oil residue was purified by flash chromatography on silica gel (5% → 25% EtOAc in hexanes) to afford compound **20ba** as a clear, pale-yellow oil (650 mg, 88% yield). **20ba** was taken on to the next step without characterization (see compound 1**4ba**).

#### Compound **20cb**

To a flame-dried round-bottom flask, a solution of sodium hydride (60% dispersion, 140 mg, 3.4 mmol) in DMF (8 mL) was made. Compound **19c** (510 mg, 1.4 mmol) was then added slowly at 0 °C. After the ceasing of gas evolution, ethyl iodide was added (0.22 mL, 2.7 mmol). The reaction was stirred at 40 °C under argon for 16 h. The reaction was quenched with 8 mL of a 1:1 10% HCl and water solution and was extracted with ethyl acetate three times (3 × 10 mL). The combined organic layers were dried over MgSO_4_, filtered, and concentrated in vacuo. The oil residue was purified by flash chromatography on silica gel (5% → 25% EtOAc in hexanes) to afford compound **20cb** as a yellow oil (430 mg, 80% yield). **20cb** was taken on to the next step without characterization (see compound 1**4cb**).

#### Compound **20ea**

To a flame-dried round-bottom flask, a solution of sodium hydride (60% dispersion, 250 mg, 6.2 mmol) in DMF (12 mL) was made. Compound **19e** (1.2 g, 3.1 mmol) was then added slowly at 0 °C. After the ceasing of gas evolution, methyl iodide was added (0.21 mL, 3.4 mmol). The reaction was stirred at 40 °C under argon for 16 h. The reaction was quenched with 12 mL of a 1:1 10% HCl and water solution and was extracted with ethyl acetate (3 × 15 mL). The combined organic layers were dried over MgSO_4_, filtered, and concentrated in vacuo. The oil residue was purified by flash chromatography on silica gel (5% → 25% EtOAc in hexanes) to afford compound **20ea** as a clear, yellow oil (1.1 g, 86% yield). **20ea** was taken on to the next step without characterization (see compound 1**4ea**).

#### Compound **20eb**

To a flame-dried round-bottom flask, a solution of sodium hydride (60% dispersion, 230 mg, 5.6 mmol) in DMF (12 mL) was made. Compound **19e** (1.1 g, 2.8 mmol) was then added slowly at 0 °C. After the ceasing of gas evolution, ethyl iodide was added (1.1 mL, 3.4 mmol). The reaction was stirred at 40 °C under argon for 16 h. The reaction was quenched with 12 mL of a 1:1 10% HCl and water solution and was extracted with ethyl acetate (3 × 15 mL). The combined organic layers were dried over MgSO_4_, filtered, and concentrated in vacuo. The oil residue was purified by flash chromatography on silica gel (5% → 30% EtOAc in hexanes) to afford compound **20eb** as a clear, yellow oil (790 mg, 68% yield). **20eb** was taken on to the next step without characterization (see compound 1**4eb**).

#### Compound **32aa**

To a solution of sodium hydride (60% dispersion, 0.47 g, 12 mmol) in DMF (20 mL) in a flame-dried dried round-bottom flask was added compound **31a** (1.9 g, 5.9 mmol) at 0 °C under argon. The solution was stirred for several minutes to allow for gas evolution. To the solution at 0 °C was added methyl iodide (0.40 mL, 6.5 mmol). The solution was warmed to 21 °C, transferred to an oil bath set to 40 °C, and stirred for 16 h. The reaction was quenched with 24 mL of a 1:1 ammonium chloride and water solution and was extracted with ethyl acetate (3 × 20 mL). The combined organic layers were dried over MgSO_4_, filtered, and concentrated in vacuo. The residue was purified by flash chromatography on silica gel (5% → 100% EtOAc in hexanes) to afford compound **32aa** as a yellow oil (1.5 g, 77% yield). **32aa** was confirmed via ^1^H NMR and ^13^C NMR and then taken on to the next step without further characterization (see compound **33aa**). ^1^H NMR (400 MHz, CDCl_3_) δ 7.82 (d, *J* = 8.1 Hz, 1H), 7.46 (t, *J* = 7.3 Hz, 1H), 7.35 (t, *J* = 7.5 Hz, 2H), 4.99 (hept, *J* = 6.2 Hz, 2H), 3.63 (s, 2H), 1.26 (s, 3H), 1.19 (t, *J* = 6.5 Hz, 12 H); ^13^C{^1^H} NMR (100 MHz, CDCl_3_) δ 171.1, 150.9, 133.1, 132.4, 131.6, 128.0, 124.8, 69.3, 54.9, 35.9, 21.6, 19.9.

#### Compound **14aa**

To a solution of compound **20aa** (2.0 g, 5.5 mmol) in ethyl acetate (30 mL) was added Pd(OH)_2_/C (340 mg, 2.5 mmol) at 21 °C. The solution was degassed and purged with hydrogen gas three times. The reaction was stirred at 21 °C under hydrogen gas for 18 h. The reaction was diluted with ethyl acetate (15 mL), passed through a silica plug to remove Pd(OH)_2_/C, and rinsed with additional ethyl acetate (20 mL). The ethyl acetate solution was concentrated in vacuo. The light-yellow solid was purified by flash chromatography on silica gel (15% → 40% EtOAc in hexanes) to afford compound **14aa** as a white crystalline solid (1.5 g, 80% yield). ^1^H NMR (400 MHz, CDCl_3_) δ 6.93 (d, *J* = 7.8 Hz, 1H), 6.90 (d, *J* = 7.7 Hz, 1H), 6.65 (d, *J* = 7.9 Hz, 1H), 6.50 (t, *J* = 7.5 Hz, 1H), 4.52 (s, 2H), 1.42 (s, 18H), 1.28 (s, 3H); ^13^C{^1^H} NMR (100 MHz, CDCl_3_) δ 171.3, 147.1, 131.8, 127.5, 120.6, 116.8, 115.6, 80.8, 56.0, 35.1, 27.2, 19.8; mp = 70.9–72.2 °C. HRMS (ESI) *m*/*z*: [M + H]^+^ calcd 336.2169 for C_19_H_29_NO_4_; found 336.2165.

#### Compound **14ab**

To a solution of compound **20ab** (340 mg, 0.89 mmol) in ethyl acetate (3.0 mL) was added Pd(OH)_2_/C (50 mg, 0.36 mmol) at 21 °C. The solution was degassed and purged with hydrogen gas five times. The reaction proceeded at 21 °C under hydrogen gas for 18 h. The reaction was diluted with ethyl acetate (15 mL), passed through a silica plug to remove Pd(OH)_2_/C, and rinsed with additional ethyl acetate (10 mL). The ethyl acetate solution was concentrated in vacuo. The oil residue was purified by flash chromatography on silica gel (10% → 100% EtOAc in hexanes) to afford compound **14ab** as a white powder (300 mg, 96% yield). ^1^H NMR (400 MHz, C_3_D_6_O) δ 6.90 (m, 2H), 6.64 (d, *J* = 8.5 Hz, 1H), 6.49 (t, *J* = 7.5 Hz, 1H), 3.00 (s, 2H), 1.88 (q, *J* = 7.4 Hz, 2H), 1.37 (s, 18H), 0.88 (t, *J* = 7.3 Hz, 3H); ^13^C{^1^H} NMR (100 MHz, C_3_D_6_O) δ 170.9, 147.1, 131.5, 127.3, 121.1, 116.8, 115.5, 80.8, 59.8, 32.3, 27.1, 26.8, 8.2; mp = 88–91 °C. HRMS (ESI) *m*/*z*: [M + H]^+^ calcd 350.2326 for C_20_H_31_NO_4_; found 350.2328.

#### Compound **14ac**

To a solution of compound **20ac** (1.3 g, 3.2 mmol) in ethyl acetate (30 mL) was added Pd(OH)_2_/C (180 mg, 1.3 mmol) at 21 °C. The solution was degassed and purged with hydrogen gas three times. The reaction was stirred at 21 °C under hydrogen gas for 18 h. The reaction was diluted with ethyl acetate (15 mL), passed through a silica plug to remove Pd(OH)_2_/C, and rinsed with additional ethyl acetate (20 mL). The ethyl acetate solution was concentrated in vacuo. The oil residue was purified by flash chromatography on silica gel (15% → 40% EtOAc in hexanes) to afford compound **14ac** as a light-yellow oil (720 mg, 61% yield). ^1^H NMR (400 MHz, CDCl_3_) δ 7.03 (d, *J* = 7.5 Hz, 1H), 6.96 (t, *J* = 7.6, 1H), 6.62 (t, *J* = 7.6, 2H), 3.09 (s, 2H), 2.45 (sept, *J* = 6.7 Hz, 1H), 1.28 (s, 18H), 1.06 (d, *J* = 6.8, 6H); ^13^C{^1^H} NMR (100 MHz, CDCl_3_) δ 171.1, 145.7, 132.5, 127.6, 123.5, 118.5, 116.3, 81.5, 63.8, 35.7, 34.1, 27.8, 18.9. HRMS (ESI) *m*/*z*: [M + H]^+^ calcd 364.2482 for C_21_H_33_NO_4_; found 364.2472.

#### Compound **14ad**

To a solution of compound **20ad** (110 mg, 0.26 mmol) in ethyl acetate (6 mL) was added Pd(OH)_2_/C (18 mg, 0.13 mmol) at 21 °C. The solution was degassed and purged with hydrogen gas three times. The reaction was stirred at 21 °C under hydrogen gas for 18 h. The reaction was diluted with ethyl acetate (25 mL), passed through a silica plug to remove Pd(OH)_2_/C, and rinsed with additional ethyl acetate (10 mL). The ethyl acetate solution was concentrated in vacuo. The oil residue was purified by flash chromatography on silica gel (15% → 40% EtOAc in hexanes) to afford compound **14ad** as a light-yellow oil (95 mg, 97% yield). ^1^H NMR (400 MHz, CDCl_3_) δ 7.00 (t, *J* = 8.7 Hz, 2H), 6.75 (dt, *J* = 14.9, 8.3 Hz, 2H), 3.12 (s, 2H), 1.96 (d, *J* = 6.3 Hz, 2H), 1.78 (sept, *J* = 6.5 Hz, 1H), 1.31 (s, 18H), 0.94 (d, *J* = 6.6 Hz, 6H); ^13^C{^1^H} NMR (100 MHz, CDCl_3_) δ 172.0, 144.5, 132.3, 127.8, 123.4, 119.2, 116.9, 81.9, 59.7, 44.8, 34.7, 27.9, 24.6, 24.3. HRMS (ESI) *m*/*z*: [M + H]^+^ calcd 378.2639 for C_22_H_35_NO_4_; found 378.2645.

#### Compound **14ae**

To a solution of compound **20ae** (7.2 g, 16 mmol) in ethyl acetate (50 mL) was added Pd(OH)_2_/C (910 mg, 6.5 mmol) at 21 °C. The solution was degassed and purged with hydrogen gas three times. The reaction was stirred at 21 °C under hydrogen gas for 18 h. The reaction was diluted with ethyl acetate (25 mL), passed through a silica plug to remove Pd(OH)_2_/C, and rinsed with additional ethyl acetate (15 mL). The ethyl acetate solution was concentrated in vacuo. The oil residue was purified by flash chromatography on silica gel (15% → 40% EtOAc in hexanes) to afford compound **14ae** as a light-yellow oil (4.3 g, 63% yield). ^1^H NMR (400 MHz, CDCl_3_) δ 7.20 (m, 5H), 7.07 (d, *J* = 7.9, 1H), 6.99 (t, *J* = 7.3, 1H), 6.65 (t, *J* = 7.4 Hz, 1H), 6.61 (d, *J* = 7.8 Hz, 1H), 3.35 (s, 2H), 3.03 (s, 2H), 1.32 (s, 18H); ^13^C{^1^H} NMR (100 MHz, CDCl_3_) δ 171.0, 145.6, 136.7, 131.6, 130.3, 128.2, 127.6, 126.9, 122.3, 118.4, 116.2, 82.0, 60.2, 41.4, 34.3, 27.8. HRMS (ESI) *m*/*z*: [M + H]^+^ calcd 412.2482 for C_25_H_33_NO_4_; found 412.2479.

#### Compound **14af**

To a solution of compound **20af** (1.1 g, 2.6 mmol) in ethyl acetate (9 mL) was added Pd(OH)_2_/C (180 mg, 1.3 mmol) at 21 °C. The solution was degassed and purged with hydrogen gas three times. The reaction was stirred at 21 °C under hydrogen gas for 18 h. The reaction was diluted with ethyl acetate (15 mL), passed through a silica plug to remove Pd(OH)_2_/C, and rinsed with additional ethyl acetate (15 mL). The ethyl acetate solution was concentrated in vacuo. The oil residue was purified by flash chromatography on silica gel (15% → 40% EtOAc in hexanes) to afford compound **14af** as a light-yellow solid (890 mg, 89% yield). ^1^H NMR (400 MHz, CDCl_3_) δ6.98 (t, *J* = 7.2 Hz, 2H), 6.64 (t, *J* = 7.1 Hz, 2H), 3.35 (t, *J* = 6.6 Hz, 2H), 3.30 (s, 3H), 3.07 (s, 2H), 1.94 (m, 2H), 1.55 (dq, *J* = 11.1, 6.7 Hz, 2H), 1.34 (s, 18H); ^13^C{^1^H} NMR (100 MHz, CDCl_3_) δ171.4, 145.3, 132.3, 127.8, 122.2, 118.6, 116.5, 81.7, 72.8, 59.9, 58.6, 33.9, 31.9, 27.8, 24.6; mp = 81.3–82.4 °C. HRMS (ESI) *m*/*z*: [M + H]^+^ calcd 394.2588 for C_22_H_35_NO_5_; found 394.2583.

#### Compound **14ba**

To a solution of compound **20ba** (650 mg, 1.7 mmol) in ethyl acetate (6 mL) was added Pd(OH)_2_/C (120 mg, 0.86 mmol) at 21 °C. The solution was degassed and purged with hydrogen gas three times. The reaction was stirred at 21 °C under hydrogen gas for 18 h. The reaction was diluted with ethyl acetate (10 mL), passed through a silica plug to remove Pd(OH)_2_/C, and rinsed with additional ethyl acetate (10 mL). The ethyl acetate solution was concentrated in vacuo. The oil residue was purified by flash chromatography on silica gel (15% → 40% EtOAc in hexanes) to afford compound **14ba** as a yellow oil (370 mg, 61% yield). ^1^H NMR (400 MHz, CDCl_3_) δ 6.94 (d, *J* = 7.3 Hz, 1H), 6.89 (d, *J* = 7.7 Hz, 1H), 6.62 (t, *J* = 7.4 Hz, 1H), 3.11 (s, 2H), 2.18 (s, 3H), 1.41 (s, 18H), 1.34 (s, 3H); ^13^C{^1^H} NMR (100 MHz, CDCl_3_) δ 172.2, 142.7, 130.3, 129.3, 123.4, 121.7, 118.4, 81.7, 56.1, 36.0, 28.0, 21.0, 18.4. HRMS (ESI) *m*/*z*: [M + H]^+^ calcd 350.2326 for C_20_H_31_NO_4_; found 350.2325.

#### Compound **14cb**

To a solution of compound **20cb** (430 mg, 1.1 mmol) in ethyl acetate (4 mL) was added Pd(OH)_2_/C (60 mg, 0.44 mmol) at 21 °C. The solution was degassed and purged with hydrogen gas five times. The reaction proceeded at 21 °C under hydrogen gas for 18 h. The reaction was diluted with ethyl acetate (15 mL), passed through a silica plug to remove Pd(OH)_2_/C, and rinsed with additional ethyl acetate (10 mL). The ethyl acetate solution was concentrated in vacuo. The oil residue was purified by flash chromatography on silica gel (10% → 100% EtOAc in hexanes) to afford compound **14cb** as a light-yellow powder (280 mg, 69% yield). ^1^H NMR (400 MHz, C_3_D_6_O) δ 6.90 (q, *J* = 7.5 Hz, 1H), 6.46 (d, *J* = 8.5 Hz, 1H), 6.25 (t, *J* = 8.9 Hz, 1H), 3.05 (s, 2H), 1.93 (q, *J* = 7.5 2H), 1.33 (s, 18H), 0.90 (t, *J* = 7.8 Hz, 3H); ^13^C{^1^H} NMR (100 MHz, C_3_D_6_O) δ 171.0, 163.0 (d, *J* = 240.0 Hz), 149.8 (d, *J* = 7.3 Hz), 128.3 (d, *J* = 11.1 Hz), 110.8, 109.1 (d, *J* = 18.6 Hz), 102.7 (d, *J* = 24.0 Hz), 81.1, 59.6, 27.0, 26.6, 8.1; ^19^F NMR (376 MHz, (CD_3_)_2_CO) δ −114.4 ppm; mp = 60–63 °C. HRMS (ESI) *m*/*z*: [M + H]^+^ calcd 368.2232 for C_20_H_30_FNO_4_; found 368.2222.

#### Compound **14da**

To a solution of compound **20da** (490 mg, 1.1 mmol) in ethyl acetate (5 mL) was added Pd(OH)_2_/C (80 mg, 0.57 mmol) at 21 °C. The solution was degassed and purged with hydrogen gas three times, after which the reaction was stirred at 21 °C under hydrogen for 18 h. The reaction was diluted with ethyl acetate (15 mL), passed through a silica plug to remove Pd(OH)_2_/C, and rinsed with additional ethyl acetate (10 mL). The ethyl acetate solution was concentrated in vacuo. The crude oil residue was purified by flash chromatography on silica gel (10% → 25% EtOAc in hexanes) to afford compound **14da** as a white solid (390 mg, 87% yield). ^1^H NMR (400 MHz, (CD_3_)_2_CO) δ 7.13 (d, *J* = 7.8 Hz, 1H), 6.98 (s, 1H), 6.79 (d, *J* = 7.9 Hz, 1H), 5.05 (br s, 2H), 3.09 (s, 2H), 1.42 (s, 18H), 1.30 (s, 3H) ppm; ^13^C{^1^H} NMR (100 MHz, (CD_3_)_2_CO) δ 171.9, 148.7, 133.2, 130.1 (q, *J* = 31.6 Hz), 125.4, 124.2, 113.3, 112.2, 81.9, 56.7, 35.7, 28.0, 20.6 ppm; ^19^F NMR (376 MHz, (CD_3_)_2_CO) δ 63.5 ppm; mp = 64.3–65.6 °C. HRMS (ESI) *m*/*z*: [M + H]^+^ calcd 404.2043 for C_20_H_28_F_3_NO_4_; found 404.2036.

#### Compound **14ea**

To a solution of compound **20ea** (1.7 g, 4.2 mmol) in ethyl acetate (17 mL) was added Pd(OH)_2_/C (240 mg, 1.7 mmol) at 21 °C. The solution was degassed and purged with hydrogen gas three times. The reaction was stirred at 21 °C under hydrogen gas for 18 h. The reaction was diluted with ethyl acetate (20 mL), passed through a silica plug to remove Pd(OH)_2_/C, and rinsed with additional ethyl acetate (20 mL). The ethyl acetate solution was concentrated in vacuo. The oil residue was purified by flash chromatography on silica gel (15% → 40% EtOAc in hexanes) to afford compound **14ea** as a thick dark orange oil (930 mg, 60% yield). ^1^H NMR (400 MHz, CDCl_3_) δ 6.92 (d, *J* = 8.8 Hz, 2H), 6.52 (dd, *J* = 8.4, 1.9 Hz, 1H), 4.06 (s, 2H), 3.00 (s, 2H), 1.39 (s, 18H), 1.32 (s, 3H); ^13^C{^1^H} NMR (100 MHz, CDCl_3_) δ 171.7, 144.5, 131.7, 127.7, 123.2, 122.5, 117.3, 81.9, 56.1, 35.6, 27.9, 21.0. HRMS (ESI) *m*/*z*: [M + H]^+^ calcd 370.1780 for C_19_H_28_ClNO_4_; found 370.1786.

#### Compound **14eb**

To a solution of compound **20eb** (130 mg, 0.31 mmol) in ethyl acetate (5 mL) was added Pd(OH)_2_/C (17 mg, 0.12 mmol) at 21 °C. The solution was degassed and purged with hydrogen gas three times. The reaction was stirred at 21 °C under hydrogen gas for 18 h. The reaction was diluted with ethyl acetate (10 mL), passed through a silica plug to remove Pd(OH)_2_/C, and rinsed with additional ethyl acetate (15 mL). The ethyl acetate solution was concentrated in vacuo. The oil residue was purified by flash chromatography on silica gel (15% → 40% EtOAc in hexanes) to afford compound **14eb** as a thick yellow oil (76 mg, 64% yield). ^1^H NMR (400 MHz, (CD_3_)_2_CO) δ 6.94 (d, *J* = 2.5 Hz, 1H), 6.90 (dd, *J* = 8.5, 2.4 Hz, 1H), 6.66 (d, *J* = 8.4 Hz, 1H), 4.72 (br s, 2H), 2.99 (s, 2H), 1.88 (q, *J* = 7.6 Hz, 2H), 1.38 (s, 18H), 0.89 (t, *J* = 7.6 Hz, 3H); ^13^C{^1^H} NMR (100 MHz, (CD_3_)_2_CO) δ 170.7, 146.3, 130.9, 127.1, 123.1, 120.6, 116.8, 81.1, 60.0, 32.4, 27.3, 27.2, 8.2. HRMS (ESI) *m*/*z*: [M + H]^+^ calcd 384.1936 for C_20_H_30_ClNO_4_; found 384.1925.

#### Compound **14fa**

To a solution of compound **20fa** (1.1 g, 2.7 mmol) in ethyl acetate (12 mL) was added Pd(OH)_2_/C (160 mg, 1.1 mmol) at 21 °C. The solution was degassed and purged with hydrogen gas three times, after which the reaction was stirred at 21 °C under hydrogen for 18 h. The reaction was diluted with ethyl acetate, passed through a silica plug to remove Pd(OH)_2_/C, and rinsed with additional ethyl acetate (15 mL). The ethyl acetate solution was concentrated in vacuo. The crude oil residue was purified by flash chromatography on silica gel (20% → 40% EtOAc in hexanes) to afford compound **14fa** as a dark-purple solid (570 mg, 57% yield). ^1^H NMR (400 MHz, (CD_3_)_2_CO) δ 6.64 (d, *J* = 8.7 Hz, 1H), 6.59 (m, 2H), 4.16 (br s, 2H), 3.65 (s, 3H), 3.05 (s, 2H), 1.46 (s, 18H), 1.31 (s, 3H) ppm; ^13^C{^1^H} NMR (100 MHz, (CD_3_)_2_CO) δ 172.2, 152.7, 141.6, 123.2, 118.2, 117.6, 114.0, 81.7, 56.9, 55.8, 36.0, 28.1, 20.5 ppm; mp = 58.8–60.1 °C. HRMS (ESI) *m*/*z*: [M + H]^+^ calcd 366.2275 for C_20_H_31_NO_5_; found 366.2265.

#### Compound **24aa**

To a solution of sodium hydride (60% dispersion, 14 mg, 0.35 mmol) and anhydrous DMF (10 mL) in a flame-dried round-bottom flask was added **14aa** (0.12 g, 0.35 mmol) at 21 °C. The reaction stirred for 10 min before methyl iodide (0.01 mL, 0.15 mmol) was added dropwise. The reaction stirred for 18 h at 21 °C before being quenched with 10 mL of saturated ammonium chloride and extracted with ethyl acetate (3 × 10 mL). The combined organic layers were dried over MgSO_4_, filtered, and concentrated in vacuo. The yellow liquid was purified by flash chromatography (10% → 50% EtOAc in hexanes) to afford compound **24aa** as a viscous yellow oil (55 mg, 45% yield). ^1^H NMR (400 MHz, (CD_3_)_2_CO) δ 7.04 (t, *J* = 7.8 Hz, 1H), 6.94 (d, *J* = 7.4 Hz, 1H), 6.50 (m, 2H), 4.79 (br s, 1H), 3.02 (s, 3H), 2.76 (d, *J* = 5.2 Hz, 3H), 1.41 (s, 18H), 1.25 (s, 3H); ^13^C{^1^H} NMR (100 MHz, (CD_3_)_2_CO) δ 171.4, 148.5, 131.5, 127.9, 120.9, 115.7, 109.7, 80.8, 55.9, 34.9, 29.9, 27.2, 19.8. HRMS (ESI) *m*/*z*: [M + H]^+^ calcd 350.2326 for C_20_H_31_NO_4_; found 350.2313.

#### Compound **33aa**

To a solution of compound **32aa** (0.36 g, 1.0 mmol) in ethyl acetate (4 mL) was added Pd(OH)_2_/C (66 mg, 0.47 mmol) at 21 °C. The solution was degassed and purged with hydrogen gas three times. The reaction was stirred at 21 °C under hydrogen gas for 18 h. The reaction was diluted with ethyl acetate (15 mL), passed through a silica plug to remove Pd(OH)_2_/C, and rinsed with additional ethyl acetate (20 mL). The ethyl acetate solution was concentrated in vacuo. The yellow liquid was purified by flash chromatography on silica gel (15% → 45% EtOAc in hexanes) to afford compound **33aa** as a thick yellow oil (31 mg, 9% yield). Most of the isolated material was the lactam (see **23aa**). ^1^H NMR (400 MHz, (CD_3_)_2_CO) δ 6.90 (m, 2H), 6.65 (d, *J* = 7.8 Hz, 1H), 6.50 (t, *J* = 7.4, 1H), 4.95 (hept, *J* = 6.2 Hz, 2H), 4.51 (br s, 1H), 3.09 (s, 2H), 1.32 (s, 3H), 1.20 (d, *J* = 6.5 Hz, 6H), 1.17 (d, *J* = 6.5 Hz, 6H); ^13^C{^1^H} NMR (100 MHz, (CD_3_)_2_CO) δ 171.5, 147.1, 131.6, 127.6, 120.3, 116.9, 115.7, 68.6, 54.9, 35.2, 21.0, 20.9, 19.4. HRMS (ESI) *m*/*z*: [M + H]^+^ calcd 308.1856 for C_17_H_25_NO_4_; found 308.1845.

#### Compound **15aa**

To a solution of compound **14aa** (110 mg, 0.32 mmol) in dry toluene (13 mL, 0.025 M) in a flame-dried two-neck round-bottom flask was added **21a** (12 mg, 0.016 mmol) at 21 °C. The reaction was stirred at 21 °C under argon for 3 days. The reaction was quenched with 14 mL of a 1:1 sodium bicarbonate and water solution and extracted with ethyl acetate (3 × 10 mL). The combined organic layers were dried over MgSO_4_, filtered, and concentrated in vacuo. The residue was purified by flash chromatography on silica gel (20% → 100% EtOAc in hexanes) to afford compound **15aa** as a white fluffy solid (81 mg, 96% yield, 71% ee). ^1^H NMR (400 MHz, CDCl_3_) δ 7.92 (br s, 1H), 7.16 (t, *J* = 7.7 Hz, 1H), 7.12 (d, *J* = 7.5 Hz, 1H), 6.96 (t, *J* = 7.5 Hz, 1H), 6.76 (d, *J* = 7.9 Hz, 1H), 3.26 (d, *J* = 15.7, Hz, 1H), 2.86 (d, *J* = 15.6 Hz, 1H), 1.48, (s, 3H) 1.21 (s, 9H); ^13^C{^1^H} NMR (100 MHz, CDCl_3_) δ 171.4, 171.2, 137.1, 128.2, 127.8, 123.2, 122.7, 115.0, 82.2, 50.1, 37.7, 27.6, 20.0; mp = 98.1–99.9 °C. HRMS (ESI) *m*/*z*: [M + H]^+^ calcd 262.1438 for C_15_H_19_NO_3_; found 262.1435. [α]_D_^21^ = +20.60 (*c* = 0.5, CHCl_3_).

#### Compound **15ab**

To a solution of compound **14ab** (100 mg, 0.29 mmol) in dry toluene (11 mL, 0.025 M) was added **21a** (11 mg, 0.014 mmol) at 21 °C. The solution was stirred at 50 °C under argon for 6 days. The reaction was quenched with 12 mL of a 1:1 sodium bicarbonate and water solution and extracted with ethyl acetate (3 × 15 mL). The combined organic layers were dried over MgSO_4_, filtered, and concentrated in vacuo. The oil residue was purified by flash chromatography on silica gel (10% → 100% EtOAc in hexanes) to afford compound **15ab** as a white powder (70 mg, 89% yield, 70% ee). ^1^H NMR (400 MHz, CDCl_3_) δ 7.40 (br s, 1H), 7.15 (m, 2H), 6.97 (t, *J* = 7.6 Hz, 1H), 6.69 (d, *J* = 8.3 Hz, 1H), 3.19 (d, *J* = 15.5, 1H), 2.94 (d, *J* = 16.2 Hz, 1H), 1.97 (m, 2H), 1.23 (s, 9H), 1.03 (t, *J* = 6.9 Hz, 3H); ^13^C{^1^H} NMR (100 MHz, CDCl_3_) δ 170.8, 170.4, 136.6, 128.2, 127.6, 123.2, 123.0, 115.0, 82.1, 54.0, 33.9, 27.5, 26.3, 9.3; mp = 73–75 °C. HRMS (ESI) *m*/*z*: [M + H]^+^ calcd 276.1594 for C_16_H_21_NO_3_; found 276.1514. [α]_D_^21^ = +11.48 (*c* = 0.5, CHCl_3_).

#### Compound **15ac**

To a solution of compound **14ac** (100 mg, 0.31 mmol) in dry toluene (13 mL, 0.025 M) was added **21a** (11 mg, 0.015 mmol) at 21 °C. The reaction was stirred at 50 °C under argon for 7 days. The reaction was quenched with 12 mL of a 1:1 sodium bicarbonate and water solution and extracted with ethyl acetate (3 × 15 mL). The combined organic layers were dried over MgSO_4_, filtered, and concentrated in vacuo. The crude oil residue was purified by flash chromatography on silica gel (15→ 30% EtOAc in hexanes) to afford compound **15ac** as a white powdery solid (70 mg, 94% yield, 50% ee). ^1^H NMR (400 MHz, CDCl_3_) δ 7.64 (br s, 1H), 7.16 (m, 1H), 7.12 (d, *J* = 7.7 Hz, 1H), 6.97 (t, *J* = 7.5 Hz, 1H), 6.70 (d, *J* = 7.8 Hz, 1H), 3.10 (d, *J* = 15.5 Hz, 1H), 2.97 (d, *J* = 15.4 Hz, 1H), 2.60 (sept, *J* = 6.9 Hz, 1H), 1.22 (s, 9H), 1.11 (d, *J* = 6.8 Hz, 3H), 1.06 (d, *J* = 7.0 Hz, 3H) ppm; ^13^C{^1^H} NMR (100 MHz, CDCl_3_) δ 169.9, 169.6, 136.3, 128.5, 127.6, 123.8, 123.3, 114.6, 82.2, 57.5, 31.5, 30.9, 19.0, 17.8 ppm; mp = 129.4–134.8 °C. HRMS (ESI) *m*/*z*: [M + H]^+^ calcd 290.1751 for C_17_H_23_NO_3_; found 290.1745. [α]_D_^21^ = +1.93 (*c* = 0.5, CHCl_3_).

#### Compound **15ad**

To a solution of compound **14ad** (120 mg, 0.35 mmol) in dry toluene (14 mL, 0.025 M) in a flame-dried two-neck round-bottom flask was added **21a** (13 mg, 0.017 mmol) at 21 °C. The reaction was stirred at 21 °C under argon for 6 days. The reaction was quenched with 14 mL of a 1:1 sodium bicarbonate and water solution and extracted with ethyl acetate (3 × 15 mL). The combined organic layers were dried over MgSO_4_, filtered, and concentrated in vacuo. The residue was purified by flash chromatography on silica gel (20% → 100% EtOAc in hexanes) to afford compound **15ad** as a light-yellow solid (82 mg, 85% yield, 55% ee). ^1^H NMR (400 MHz, CDCl_3_) δ 8.52 (br s, 1H), 7.15 (t, *J* = 7.8 Hz, 2H), 6.96 (t, *J* = 7.5 Hz, 1H), 6.78 (d, *J* = 7.6 Hz, 1H), 3.25 (d, *J* = 15.6 Hz, 1H), 2.96 (d, *J* = 15.7 Hz, 1H), 1.88 (s, 1H), 1.84 (m, 1H), 1.40 (s, 1H), 1.23 (s, 9H), 0.96 (d, *J* = 6.9 Hz, 3H), 0.94 (d, *J* = 6.4 Hz, 3H); ^13^C{^1^H} NMR (100 MHz, CDCl_3_) δ 171.1, 170.7, 136.7, 128.3, 127.6, 123.2, 123.1, 114.9, 82.1, 53.9, 40.9, 34.3, 27.7, 25.1, 24.4, 24.1; mp = 106.2–109.8 °C. HRMS (ESI) *m*/*z*: [M + H]^+^ calcd 304.1907 for C_18_H_25_NO_3_; found 304.1904. [α]_D_^21^ = +4.42 (*c* = 0.5, CHCl_3_).

#### Compound **15ae**

To a solution of compound **14ae** (300 mg, 0.73 mmol) in dry toluene (30 mL, 0.025 M) was added **21a** (27 mg, 0.036 mmol) at 21 °C. The reaction was stirred at 50 °C under argon for 7 days. The reaction was quenched with 30 mL of a 1:1 sodium bicarbonate and water solution and extracted with ethyl acetate (3 × 20 mL). The combined organic layers were dried over MgSO_4_, filtered, and concentrated in vacuo. The crude oil residue was purified by flash chromatography on silica gel (15→ 30% EtOAc in hexanes) to afford compound **15ae** as a white powdery solid (220 mg, 87% yield, 52% ee). ^1^H NMR (400 MHz, CDCl_3_) δ 9.54 (br s, 1H), 7.33 (d, *J* = 6.8 Hz, 2H), 7.23 (m, 3H), 7.16 (t, *J* = 7.8 Hz, 1H), 7.08 (d, *J* = 7.4 Hz, 1H), 6.95 (t, *J* = 7.5 Hz, 1H), 6.89 (dd, *J* = 7.8, 2.7 Hz, 1H), 3.48 (d, *J* = 13.5 Hz, 1H), 3.30 (d, *J* = 13.6 Hz, 1H), 3.13 (d, *J* = 15.6 Hz, 1H), 2.80 (d, *J* = 15.6 Hz, 1H), 1.20 (s, 9H); ^13^C{^1^H} NMR (100 MHz, CDCl_3_) δ 170.9, 170.3, 136.8, 136.5, 131.0, 128.2, 127.7, 127.0, 123.2, 122.94, 122.90, 115.3, 82.5, 55.1, 38.4, 34.0, 27.6; mp = 118.8–122.0 °C. HRMS (ESI) *m*/*z*: [M + H]^+^ calcd 338.1751 for C_21_H_23_NO_3_; found 338.1744. [α]_D_^21^ = +1.21 (*c* = 0.5, CHCl_3_).

#### Compound **15af**

To a solution of compound **14af** (120 mg, 0.30 mmol) in dry toluene (12 mL, 0.025 M) was added **21a** (11 mg, 0.015 mmol) at 21 °C. The reaction was stirred at 50 °C under argon for 6 days. The reaction was quenched with 12 mL of a 1:1 sodium bicarbonate and water solution and extracted with ethyl acetate (3 × 15 mL). The combined organic layers were dried over MgSO_4_, filtered, and concentrated in vacuo. The crude oil residue was purified by flash chromatography on silica gel (15→ 30% EtOAc in hexanes) to afford compound **15af** as a light orange solid (92 mg, 96% yield, 23% ee). ^1^H NMR (400 MHz, CDCl_3_) δ 8.40 (br s, 1H), 7.13 (m, 2H), 6.95 (t, *J* = 7.4 Hz, 1H), 6.75 (d, *J* = 7.8 Hz, 1H), 3.40 (t, *J* = 6.4 Hz, 2H), 3.31 (s, 3H), 3.20 (d, *J* = 15.5 Hz, 1H), 2.94 (d, *J* = 15.4 Hz, 1H), 1.94 (qd, *J* = 12.6, 4.9 Hz, 2H), 1.79 (dp, *J* = 17.9, 6.1 Hz, 1H), 1.68 (dp, *J* = 12.2, 6.0 Hz, 1H), 1.22 (s, 9H); ^13^C{^1^H} NMR (100 MHz, CDCl_3_) δ 170.8, 170.4, 136.7, 128.4, 127.8, 123.3, 122.9, 115.1, 82.3, 72.8, 58.6, 53.5, 34.6, 30.0, 27.7, 25.0; mp = 94.4–95.9 °C. HRMS (ESI) *m*/*z*: [M + H]^+^ calcd 320.1856 for C_18_H_25_NO_4_; found 320.1827. [α]_D_^21^ = +2.28 (*c* = 0.5, CHCl_3_).

#### Compound **15ba**

To a solution of compound **14ba** (200 mg, 0.57 mmol) in dry toluene (23 mL, 0.025 M) was added **21a** (22 mg, 0.029 mmol) at 21 °C. The reaction was stirred at 21 °C for 4 days and then 50 °C for 3 days under argon. The reaction was quenched with 24 mL of a 1:1 sodium bicarbonate and water solution and extracted with ethyl acetate (3 × 20 mL). The combined organic layers were dried over MgSO_4_, filtered, and concentrated in vacuo. The crude oil residue was purified by flash chromatography on silica gel (15→ 30% EtOAc in hexanes) to afford compound **15ba** as a white solid (150 mg, 94% yield, 40% ee). ^1^H NMR (400 MHz, CDCl_3_) δ 7.59 (br s, 1H), 7.02 (d, *J* = 7.6 Hz, 1H), 6.99 (d, *J* = 7.3 Hz, 1H), 6.88 (t, *J* = 7.5 Hz, 1H), 3.22 (d, *J* = 15.5 Hz, 1H), 2.87 (d, *J* = 15.4 Hz, 1H), 2.23 (s, 3H), 1.49 (s, 3H), 1.19 (s, 9H); ^13^C{^1^H} NMR (100 MHz, CDCl_3_) δ 171.3, 171.2, 135.4, 130.0, 126.0, 122.9, 122.7, 122.4, 82.2, 50.0, 38.0, 27.6, 19.9, 16.8; mp = 101.7–104.0 °C. HRMS (ESI) *m*/*z*: [M + H]^+^ calcd 276.1594 for C_16_H_21_NO_3_; found 276.1513. [α]_D_^24^ = +25.66 (*c* = 0.5, CHCl_3_).

#### Compound **15cb**

To a solution of compound **14cb** (100 mg, 0.27 mmol) in dry toluene (11 mL, 0.025 M) was added **21a** (10 mg, 0.014 mmol) at 21 °C. The reaction was stirred at 50 °C under argon for 6 days. The reaction was quenched with 12 mL of a 1:1 sodium bicarbonate and water solution and extracted with ethyl acetate (3 × 10 mL). The combined organic layers were dried over MgSO_4_, filtered, and concentrated in vacuo. The oil residue was purified by flash chromatography on silica gel (10% → 100% EtOAc in hexanes) to afford compound **15cb** as a yellow powder (53 mg, 66% yield, 56% ee). ^1^H NMR (400 MHz, CDCl_3_) δ 7.11 (q, *J* = 7.2 Hz, 1H), 6.72 (t, *J* = 8.5 Hz, 1H), 6.55 (d, *J* = 7.7 Hz, 1H), 3.44 (d, *J* = 15.6 Hz, 1H), 2.77 (d, *J* = 15.8 Hz, 1H), 2.04 (m, 1H), 1.94 (m, 1H), 1.27 (s, 9H), 1.04 (t, *J* = 7.4 Hz, 3H); ^13^C{^1^H} NMR (100 MHz, CDCl_3_) δ 170.7, 170.0, 160.3 (d, *J* = 244.9 Hz), 138.6 (d, *J* = 7.3 Hz), 128.6 (d, *J* = 9.5 Hz), 110.8 (d, *J* = 7.4 Hz), 110.5, 110.1 (d, *J* = 22.0 Hz), 82.3, 53.6, 27.7, 26.5, 26.4, 9.3; ^19^F NMR (376 MHz, CDCl_3_) δ −118.9 ppm; mp = 88–90 °C. HRMS (ESI) *m*/*z*: [M + H]^+^ calcd 294.1500 for C_16_H_20_FNO_3_; found 294.1499. [α]_D_^21^ = +4.20 (*c* = 0.5, CHCl_3_).

#### Compound **15da**

To a solution of compound **14da** (99 mg, 0.25 mmol) in dry toluene (10 mL, 0.025 M) was added **21a** (9.3 mg, 0.023 mmol) at 21 °C. The reaction was stirred at 21 °C under argon for 5 days. The reaction was quenched with 10 mL of a 1:1 sodium bicarbonate and water solution and extracted with ethyl acetate (3 × 10 mL). The combined organic layers were dried over MgSO_4_, filtered, and concentrated in vacuo. The crude oil residue was purified by flash chromatography on silica gel (15→ 30% EtOAc in hexanes) to afford compound **15da** as a white solid (71 mg, 86% yield, 59% ee). ^1^H NMR (400 MHz, CDCl_3_) δ 9.29 (br s, 1H), 7.25 (m, 1H), 7.10 (s, 1H), 3.37 (d, *J* = 15.8 Hz, 1H), 2.93 (d, *J* = 15.8 Hz, 1H), 1.53 (s, 3H), 1.26 (s, 9H); ^13^C{^1^H} NMR (100 MHz, CDCl_3_) δ 171.8, 170.7, 137.7, 130.4 (q, *J* = 32.6 Hz), 128.6, 126.6, 125.2, 122.5, 119.9, 112.1, 82.7, 49.9, 37.3, 27.7, 20.0; ^19^F NMR (376 MHz, (CD_3_)_2_CO) δ 62.5 ppm; mp = 113.7–120.1 °C. HRMS (ESI) *m*/*z*: [M + H]^+^ calcd 330.1312 for C_16_H_18_F_3_NO_3_; found 330.1308. [α]_D_^25^ = +22.70 (*c* = 0.5, CHCl_3_).

#### Compound **15ea**

To a solution of compound **14ea** (120 mg, 0.32 mmol) in dry toluene (13 mL, 0.025 M) was added **21a** (12 mg, 0.016 mmol) at 21 °C. The reaction was stirred at 21 °C for 3 days and then 50 °C for 3 days under argon. The reaction was quenched with 14 mL of a 1:1 sodium bicarbonate and water solution and extracted with ethyl acetate (3 × 15 mL). The combined organic layers were dried over MgSO_4_, filtered, and concentrated in vacuo. The crude oil residue was purified by flash chromatography on silica gel (15→ 30% EtOAc in hexanes) to afford compound **15ea** as a white solid (74 mg, 78% yield, 59% ee). ^1^H NMR (400 MHz, CDCl_3_) δ 8.78 (br s, 1H), 7.13 (m, 2H), 6.75 (d, *J* = 8.1 Hz, 1H), 3.25 (d, *J* = 15.8, 1H), 2.83 (d, *J* = 15.6 Hz, 1H), 1.48 (s, 3H), 1.25 (s, 9H); ^13^C{^1^H} NMR (100 MHz, CDCl_3_) δ 171.4, 170.8, 135.8, 128.09, 128.05, 127.8, 124.4, 116.3, 82.5, 50.0, 37.3, 27.7, 20.0; mp = 159.5–160.9 °C. HRMS (ESI) *m*/*z*: [M + H]^+^ calcd 296.1048 for C_15_H_18_ClNO_3_; found 296.1044. [α]_D_^20^ = +4.91 (*c* = 0.5, CHCl_3_).

#### Compound **15eb**

To a solution of compound **14eb** (130 mg, 0.33 mmol) in dry toluene (13 mL, 0.025 M) was added **21a** (13 mg, 0.017 mmol) at 21 °C. The reaction was stirred at 21 °C under argon for 7 days. The reaction was quenched with 14 mL of a 1:1 sodium bicarbonate and water solution and extracted with ethyl acetate (3 × 15 mL). The combined organic layers were dried over MgSO_4_, filtered, and concentrated in vacuo. The crude oil residue was purified by flash chromatography on silica gel (15→ 30% EtOAc in hexanes) to afford compound **15eb** as a white solid (86 mg, 83% yield, 58% ee). ^1^H NMR (400 MHz, CDCl_3_) δ 9.21 (br s, 1H), 7.10 (m, 2H), 6.74 (d, *J* = 8.3 Hz, 1H), 3.17 (d, *J* = 15.8 Hz, 1H), 2.89 (d, *J* = 15.7 Hz, 1H), 1.95 (ddt, *J* = 31.9, 14.1, 6.9 Hz, 2H), 1.26 (s, 9H), 1.01 (t, *J* = 7.4 Hz, 3H); ^13^C{^1^H} NMR (100 MHz, CDCl_3_) δ 171.0, 170.1, 135.5, 128.1, 128.0, 127.6, 124.8, 116.4, 82.4, 53.8, 33.6, 27.7, 26.3, 9.3; mp = 158.5–161.8 °C. HRMS (ESI) *m*/*z*: [M + H]^+^ calcd 310.1204 for C_16_H_20_ClNO_3_; found 310.1193. [α]_D_^21^ = +3.82 (*c* = 0.5, CHCl_3_).

#### Compound **15fa**

To a solution of compound **14fa** (100 mg, 0.28 mmol) in dry toluene (11 mL, 0.025 M) was added **21a** (10 mg, 0.014 mmol) at 21 °C. The reaction was stirred at 40 °C under argon for 5 days. The reaction was quenched with 12 mL of a 1:1 sodium bicarbonate and water solution and extracted with ethyl acetate (3 × 15 mL). The combined organic layers were dried over MgSO_4_, filtered, and concentrated in vacuo. The crude oil residue was purified by flash chromatography on silica gel (20% → 35% EtOAc in hexanes) to afford compound **15fa** as a white solid (62 mg, 78% yield, 62% ee). ^1^H NMR (400 MHz, CDCl_3_) δ 8.45 (br s, 1H), 6.71 (m, 3H), 3.76 (s, 3H), 3.25 (d, *J* = 16.0 Hz, 1H), 2.84 (d, *J* = 15.6 Hz, 1H), 1.48 (s, 3H), 1.26 (s, 9H) ppm; ^13^C{^1^H} NMR (100 MHz, CDCl_3_) δ 171.3, 171.2, 155.7, 130.6, 124.1, 116.0, 113.9, 112.9, 82.2, 55.6, 50.0, 37.9, 27.8, 20.0 ppm; mp = 139.2–143.5 °C. HRMS (ESI) *m*/*z*: [M + H]^+^ calcd 292.1543 for C_16_H_21_NO_4_; found 292.1543. [α]_D_^25^ = +3.08 (*c* = 0.5, CHCl_3_).

#### Compound **22aa**

To a solution of **24aa** (24 mg, 0.07 mmol) in dry toluene (3 mL) in a flame-dried round-bottom flask under argon was added (*R*)-TRIP (2.6 mg, 0.003 mmol). The solution was stirred at 50 °C for 7 days and stirred at 75 °C for an additional 3 days. The solution was then cooled to room temperature and quenched with 5 mL of a 1:1 ratio of sodium bicarbonate and water. The solution was extracted with ethyl acetate (3 × 5 mL), dried over MgSO_4_, filtered, and concentrated in vacuo. The yellow oil was purified by flash chromatography on silica gel (10% → 50% EtOAc in hexanes) to afford compound **22aa** as a thick yellow oil (11 mg, 57% yield, 75% ee). ^1^H NMR (400 MHz, (CD_3_)_2_CO) δ 7.25 (t, *J* = 7.8 Hz, 1H), 7.16 (d, *J* = 7.5 Hz, 1H), 7.06 (d, *J* = 8.0 Hz, 1H), 6.97 (t, *J* = 7.4 Hz, 1H), 3.31 (s, 3H), 3.13 (d, *J* = 15.1 Hz, 1H), 2.86 (d, *J* = 15.3 Hz, 1H), 1.37 (s, 3H), 1.11 (s, 9H); ^13^C{^1^H} NMR (100 MHz, (CD_3_)_2_CO) δ 171.2, 169.4, 140.9, 127.9, 127.8, 124.4, 122.4, 114.4, 80.9, 50.0, 37.2, 29.4, 26.9, 20.0. HRMS (ESI) *m*/*z*: [M + H]^+^ calcd 276.1594 for C_16_H_21_NO_3_; found 276.1597. [α]_D_^21^ = +64.11 (*c* = 0.5, CHCl_3_).

#### Compound **23aa**

Upon working up, purifying, and characterizing compound **33aa**, compound **23aa** was isolated and characterized due to its spontaneous formation in the pursuit to synthesize free amine **33aa**. The white powder was purified by flash chromatography on silica gel (20% → 50% EtOAc in hexanes) to afford compound **23aa** as a thick white solid (0.20 g, 63% yield). ^1^H NMR (400 MHz, CDCl_3_) δ 9.05 (br s, 1H), 7.15 (td, *J* = 7.7, 1.4 Hz, 1H), 7.11 (d, *J* = 7.4 Hz, 1H), 6.95 (td, *J* = 7.6, 1.4 Hz, 1H), 6.82 (dd, *J* = 8.0, 1.1 Hz, 1H), 4.90 (hept, *J* = 6.3 Hz, 1H), 3.36 (d, *J* = 15.6 Hz, 1H), 2.86 (d, *J* = 15.7 Hz, 1H), 1.50 (s, 3H), 1.09 (d, *J* = 6.3 Hz, 3H), 0.99 (d, *J* = 6.3 Hz, 3H); ^13^C{^1^H} NMR (100 MHz, CDCl_3_) δ 171.7, 171.5, 137.0, 128.1, 127.9, 123.2, 122.3, 115.4, 69.1, 49.6, 37.3, 21.5, 21.4, 20.0. HRMS (ESI) *m*/*z*: [M + H]^+^ calcd 248.1281 for C_14_H_17_NO_3_; found 248.1276.

#### Compound **26**

To a solution of lithium aluminum hydride (26 mg, 0.69 mmol) in dry THF (0.55 mL) was slowly added a solution of compound **15aa** (120 mg, 0.46 mmol, 75% ee) in dry THF (0.35 mL) at 0 °C. The reaction was stirred at −40 °C for 1 h under argon and then stirred at 0 °C for an additional 1 h under argon. At 0 °C, the reaction mixture was diluted with diethyl ether (2 mL), followed by 0.03 mL of water, 0.03 mL of sodium bicarbonate, and 0.09 mL of water. The slurry was stirred at 21 °C for 10 min before 30 mg of MgSO_4_ was added to the flask. The slurry was passed through a Celite plug, rinsed with ethyl acetate (15 mL), and extracted with ethyl acetate (3 × 10 mL). The solution was dried over MgSO_4_, filtered, and concentrated in vacuo. The oil residue was purified by flash chromatography on silica gel (30% → 100% EtOAc in hexanes) to afford compound **26** as a white solid (45 mg, 53% yield, 74% ee). ^1^H NMR (400 MHz, CDCl_3_) δ 9.63 (s, 1H), 9.43 (br s, 1H), 7.19 (m, 2H), 6.96 (m, 2H), 3.36 (d, *J* = 16.1 Hz, 1H), 2.78 (d, *J* = 15.8 Hz, 1H), 1.24 (s, 3H); ^13^C{^1^H} NMR (100 MHz, CDCl_3_) δ 199.4, 169.7, 137.3, 128.6, 127.7, 122.8, 121.3, 115.1, 52.2, 32.7, 16.5; mp = 132.4–136.0 °C. HRMS (ESI) *m*/*z*: [M + H]^+^ calcd 190.0863 for C_11_H_11_NO_2_; found 190.0857. [α]_D_^23^ = −19.51 (*c* = 0.5, CHCl_3_).

#### Compound **27**

To a solution of lithium aluminum hydride (44 mg, 1.2 mmol) in dry THF (0.90 mL) was slowly added a solution of compound **15aa** (80 mg, 0.31 mmol, 87% ee) in dry THF (0.3 mL) at 0 °C. The reaction was stirred at 0 °C under argon and warmed to 21 °C while running overnight. The reaction mixture was diluted with diethyl ether (3 mL), followed by 0.04 mL of water, 0.04 mL of sodium bicarbonate, and 0.12 mL of water. The slurry was stirred at 21 °C for 15 min before 40 mg of MgSO_4_ was added to the flask. The slurry was passed through a Celite plug, rinsed with ethyl acetate (15 mL), and extracted with ethyl acetate (3 × 10 mL). The solution was dried over MgSO_4_, filtered, and concentrated in vacuo. The oil residue was purified by flash chromatography on silica gel (30% → 100% EtOAc in hexanes) to afford compound **27** as a light-yellow oil (25 mg, 46% yield, 85% ee). ^1^H NMR (400 MHz, CDCl_3_) δ 6.97 (m, 2H), 6.64 (t, *J* = 7.4, 1H), 6.52 (d, *J* = 7.9, 1H), 3.53 (d, *J* = 10.9 Hz, 1H), 3.42 (d, *J* = 10.9 Hz, 1H), 3.16 (d, *J* = 11.3 Hz, 1H), 2.96 (d, *J* = 11.2 Hz, 1H), 2.83 (br s, 1H), 2.53 (m, 2H), 1.02 (s, 3H); ^13^C{^1^H} NMR (100 MHz, CDCl_3_) δ 143.4, 130.1, 126.8, 120.4, 117.9, 114.4, 68.9, 48.8, 36.4, 33.4, 22.2. HRMS (ESI) *m*/*z*: [M + H]^+^ calcd 192.1383 for C_11_H_15_NO; found 192.1376. [α]_D_^24^ = −2.50 (*c* = 0.5, CHCl_3_).

#### Compound **28**

To a solution of Na_2_PdCl_4_ (13 mg, 0.045 mmol) and sSPhos (55 mg, 0.11 mmol) in water (2.5 mL) was added **15ea** (66 mg, 0.22 mmol, 59% ee) in a vial. The solution was stirred at 60 °C for 15 min. A solution of potassium carbonate (120 mg, 0.89 mmol) and 4-tolylboronic acid (61 mg, 0.45 mmol) in a 4:1 mixture of water and acetonitrile (3.5 mL water, 0.85 mL acetonitrile) was then added to the solution containing **15ea**. The reaction mixture was then stirred at 80 °C overnight. The solution was diluted with brine (10 mL) and extracted with ethyl acetate (3 × 20 mL). The combined organic layers were washed with brine, dried over MgSO_4_, filtered, and concentrated in vacuo. The white solid was purified by flash chromatography on silica gel (25% → 100% EtOAc in hexanes) to afford compound **28** as an off-white crystalline solid (44 mg, 56% yield, 52% ee). ^1^H NMR (400 MHz, CDCl_3_) δ 8.50 (br s, 1H), 7.42 (t, *J* = 7.3, 2H), 7.37 (d, *J* = 10.0 Hz, 2H), 7.23 (m, 2H), 6.85 (dd, *J* = 9.1, 5.3 Hz, 1H), 3.33 (d, *J* = 15.6 Hz, 1H), 2.93 (d, *J* = 15.6 Hz, 1H), 2.38 (s, 3H), 1.52 (s, 3H), 1.24 (s, 9H); ^13^C{^1^H} NMR (100 MHz, CDCl_3_) δ 171.5, 171.2, 137.6, 137.0, 136.3, 136.2, 129.6, 126.7, 126.6, 126.3, 123.1, 115.4, 82.3, 50.2, 37.8, 27.7, 21.2, 20.0; mp = 166.7–169.9 °C. HRMS (ESI) *m*/*z*: [M + H]^+^ calcd 352.1907 for C_22_H_25_NO_3_; found 352.1899. [α]_D_^22^ = 12.00 (*c* = 0.5, CHCl_3_).

## Data Availability

The data underlying this study are available in the published article and its Supporting Information.

## Supporting Information Available

^1^H and ^13^C spectra of new compounds, HPLC traces of enantioenriched products, detailed guide to compound numbering, DFT details and Cartesian coordinates, X-ray crystallography information (PDF)
FAIR data, including the primary NMR FID files, for compounds **14aa–af**, **14ba**, **14da–fa**, **14cb**, **14eb**, **15aa–af**, **15ab**, **15da–fa**, **15cb**, **15eb**, **18f**, **19a–c**, **19e**, **20aa**, **20da**, **20fa**, **22aa**, **24aa**, **26**, **27**, **28**, **32aa**, and **33aa** (ZIP)
