# Supplementary material for: Synthesis of Lactams via a Chiral Phosphoric Acid-Catalyzed Aniline Cyclization
Source: J Org Chem. 2024 Aug 9;89(17):12725–38. doi: 10.1021/acs.joc.4c01060 (PMC11382273; doi:10.1021/acs.joc.4c01060)
Supplement: Supplementary file 1 — jo4c01060_si_001.pdf [file jo4c01060_si_001.pdf]

The Journal of Organic Chemistry
Supporting Information

for

Synthesis of Lactams via a Chiral Phosphoric Acid Catalyzed Aniline Cyclization

Abigail H. Horchar, Jonathan E. Dean, Alexander R. Lake, Jessica E. Carsley, Tiana R. Lillevig, Shubin Liu, Kimberly S. Petersen*

Department of Chemistry and Biochemistry, University of North Carolina at Greensboro

1400 Spring Garden St, Greensboro, NC 27412

Email: kspeters@uncg.edu

Contents:

1.	Full Optimization Table	S2
2.	Structure numbering guide	S3
3.	Synthetic route for **22aa**	S3
4.	Copies of NMR Spectra	S4 – S93
5.	Copies of Chiral Chromatograms	S94 – S108
6.	DFT Figure and Coordinates	S109 – 127
7.	Enantioselective Recrystallization Procedure	S127
8.	Crystallography Information	S127 – 139

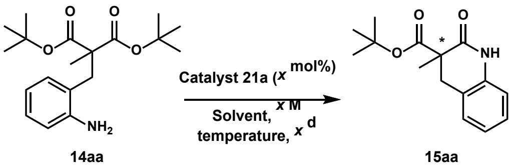

Entry	Catalyst	Cat. Loading	Solvent	Concentration (M)	Temperature (°C)	Time (d)	% ee[c]	% Yield[b]
1[a]	21a	10 mol%	1,2-DCE	0.025	r.t.	3	50	98
2	21b	10 mol%	1,2-DCE	0.025	r.t.	3	34	--
3	21c	10 mol%	1,2-DCE	0.025	r.t.	3	2	89
4	21d	10 mol%	1,2-DCE	0.025	r.t.	3	7	90
5	21e	10 mol%	1,2-DCE	0.025	r.t.	3	0	34
6	21f	10 mol%	1,2-DCE	0.025	r.t.	3	28	--
7	21g	10 mol%	1,2-DCE	0.025	r.t.	3	44[d]	--
8	21a	10 mol%	Hexanes	0.025	r.t.	3	61	99
9	21a	10 mol%	DCM	0.025	r.t.	3	55	75
10	21a	10 mol%	Bromobenzene	0.025	r.t.	3	10	--
11	21a	10 mol%	DCM/Hexanes 1:1	0.025	r.t.	3	59	31
12	21a	10 mol%	Toluene/DCM 1:1	0.025	r.t.	3	58	90
13	21a	10 mol%	Toluene/DCM 10:1	0.025	r.t.	3	67	92
14	21a	10 mol%	DCM/Toluene 10:1	0.025	r.t.	3	56	87
15	21a	10 mol%	Toluene/DCM 20:1	0.025	r.t.	3	68	66
16	21a	10 mol%	Hexane/1,2-DCE 1:1	0.025	r.t.	3	54	56
17	21a	10 mol%	Toluene	0.025	r.t.	3	67	93
18	21a	10 mol%	1,2-DCE	0.025	0	3	55	83
19	21a	10 mol%	Toluene	0.025	0	3	67	89
20	21a	10 mol%	1,2-DCE	0.25	r.t.	3	50	83
21	21a	10 mol%	Toluene	0.25	r.t.	3	63	98
22	21a	10 mol%	1,2-DCE	0.0025	r.t.	3	50	83
23	21a	10 mol%	Toluene	0.0025	r.t.	3	72	99
24	21a	5 mol%	Toluene	0.025	r.t.	3	69	97
25[e]	21a	5 mol%	Toluene	0.0025	r.t.	3	73	97
26	21a	1 mol%	Toluene	0.025	r.t.	3	68	53
27	21a	1 mol%	Toluene	0.0025	r.t.	3	71	48
28	21a	5 mol%	Toluene	0.025	r.t.	7	67	99
29	21a	5 mol%	Toluene	0.0025	r.t.	7	68	93
30	21a	1 mol%	Toluene	0.025	r.t.	7	55	47
31	21a	1 mol%	Toluene	0.0025	r.t.	7	66	61
32	21a	1 mol%	Toluene	0.025	50	3	65	97
33	21a	1 mol%	Toluene	0.0025	50	3	70	97
34	21a	5 mol%	Toluene; H_2O/3A MS	0.025	r.t.	5	24	10
35	21a	5 mol%	Toluene; 3A MS	0.025	r.t.	5	56	25
36	21a	5 mol%	Toluene; 3A MS	0.0025	r.t.	5	51	19
37	21a	5 mol%	Toluene	0.025	-20	10	33	39

Table S1: [a]*Base Conditions: 10 mol% 21a, 0.025 M in 1,2-DCE, stirring for 3 days at room temperature.* [b]*qNMR yields based on* ^1H *NMR analysis using 1,3,5-trimethoxybenzene as an internal standard.* [c]*% ee values obtained via HPLC analysis.* [d]*opposite enantiomer was formed per HPLC analysis.*[e]*Conditions giving the best results based first on % ee and second, % yield.*

Structure Numbering Elucidation Figure and 22aa Synthetic Scheme

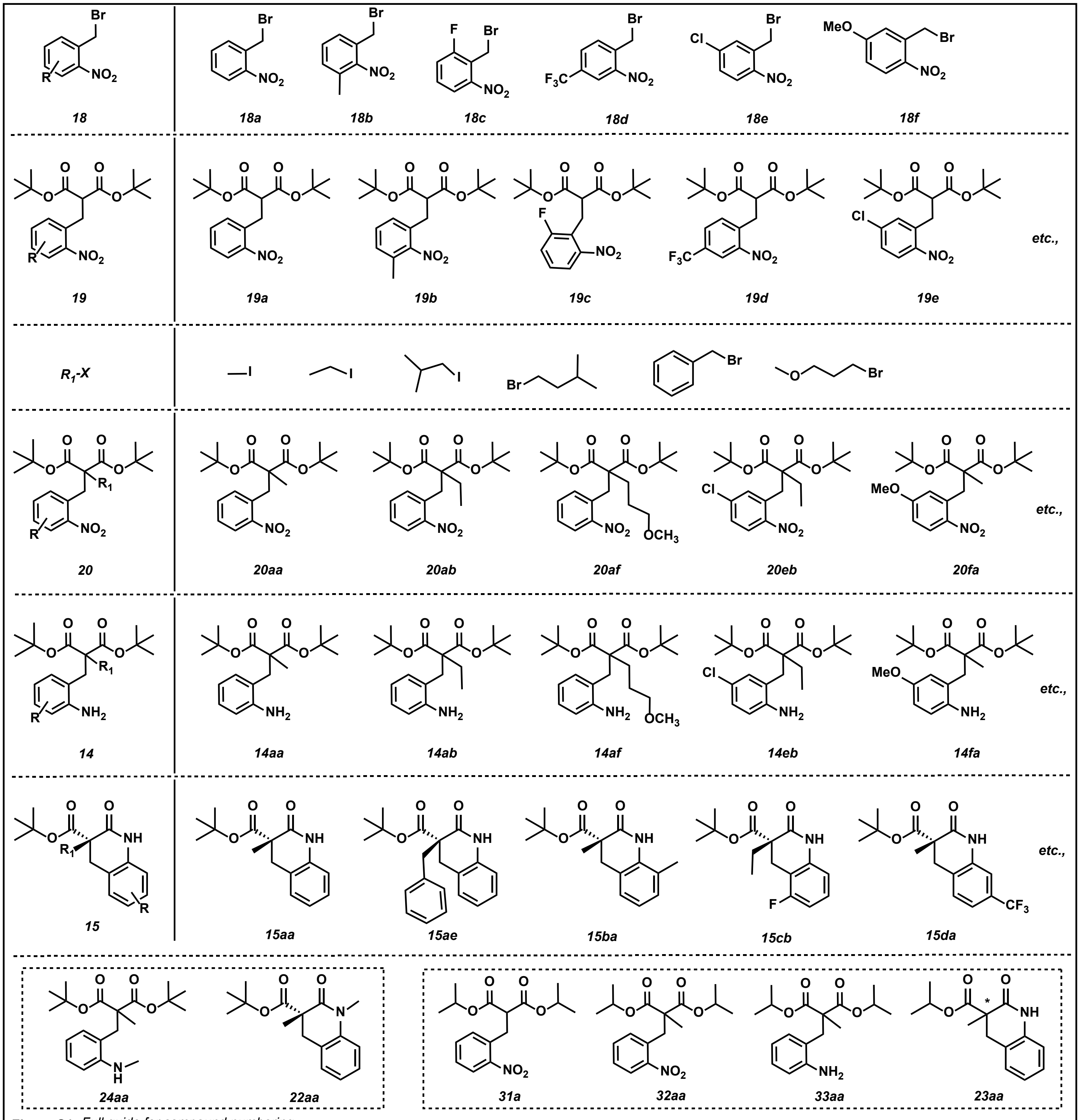

Figure S1: *Full guide for compound numbering.*

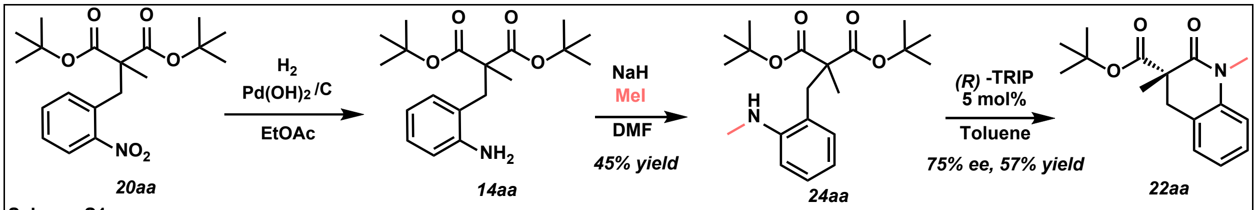

Scheme S1: *Synthesis of secondary amine and subsequent lactam.*

Copies of NMR Spectra

*Compound **18b***

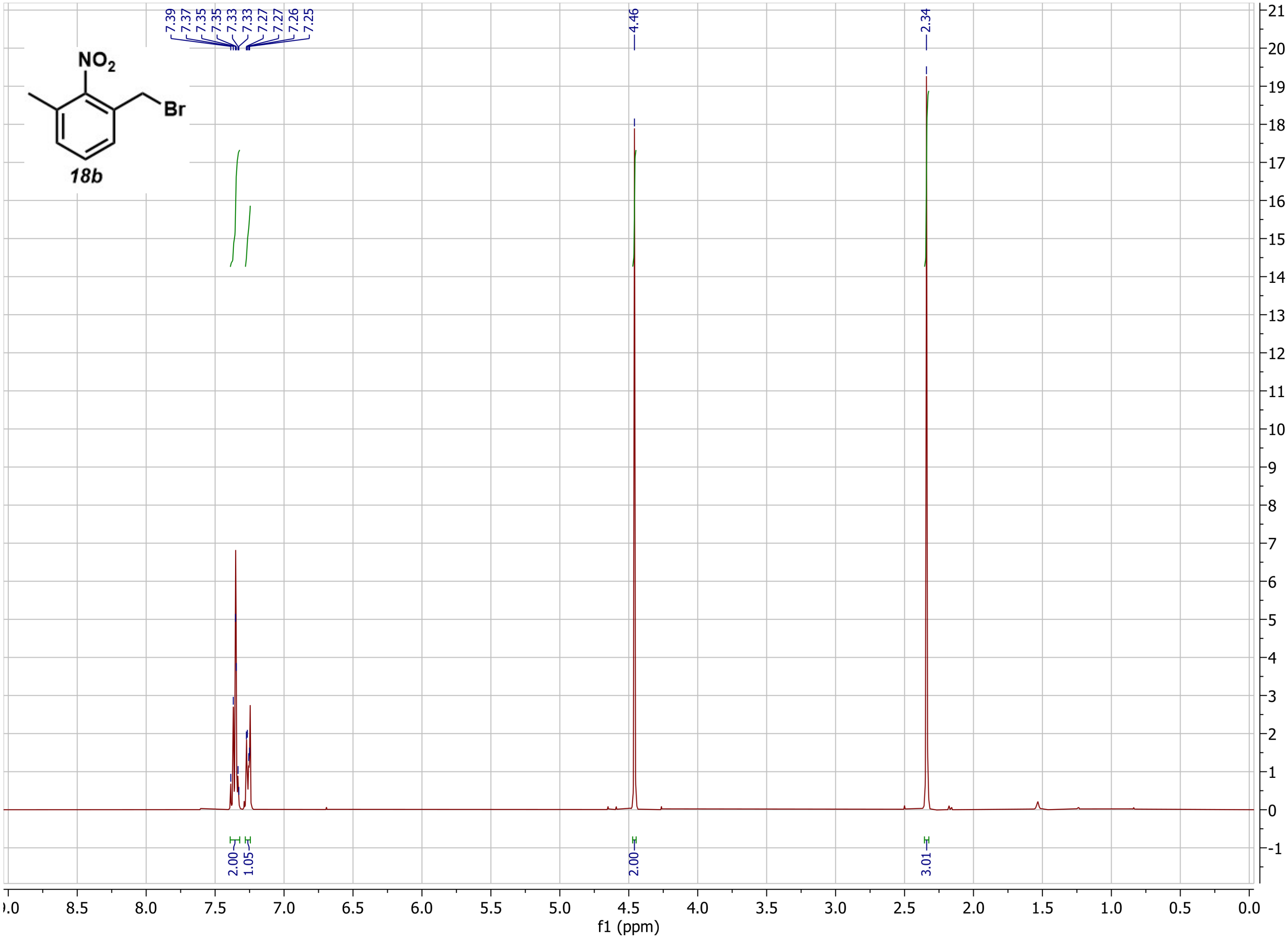

^{1}H NMR. Solvent: $CDCl_3$. B_0 = 400 MHz.

Compound ***18b***

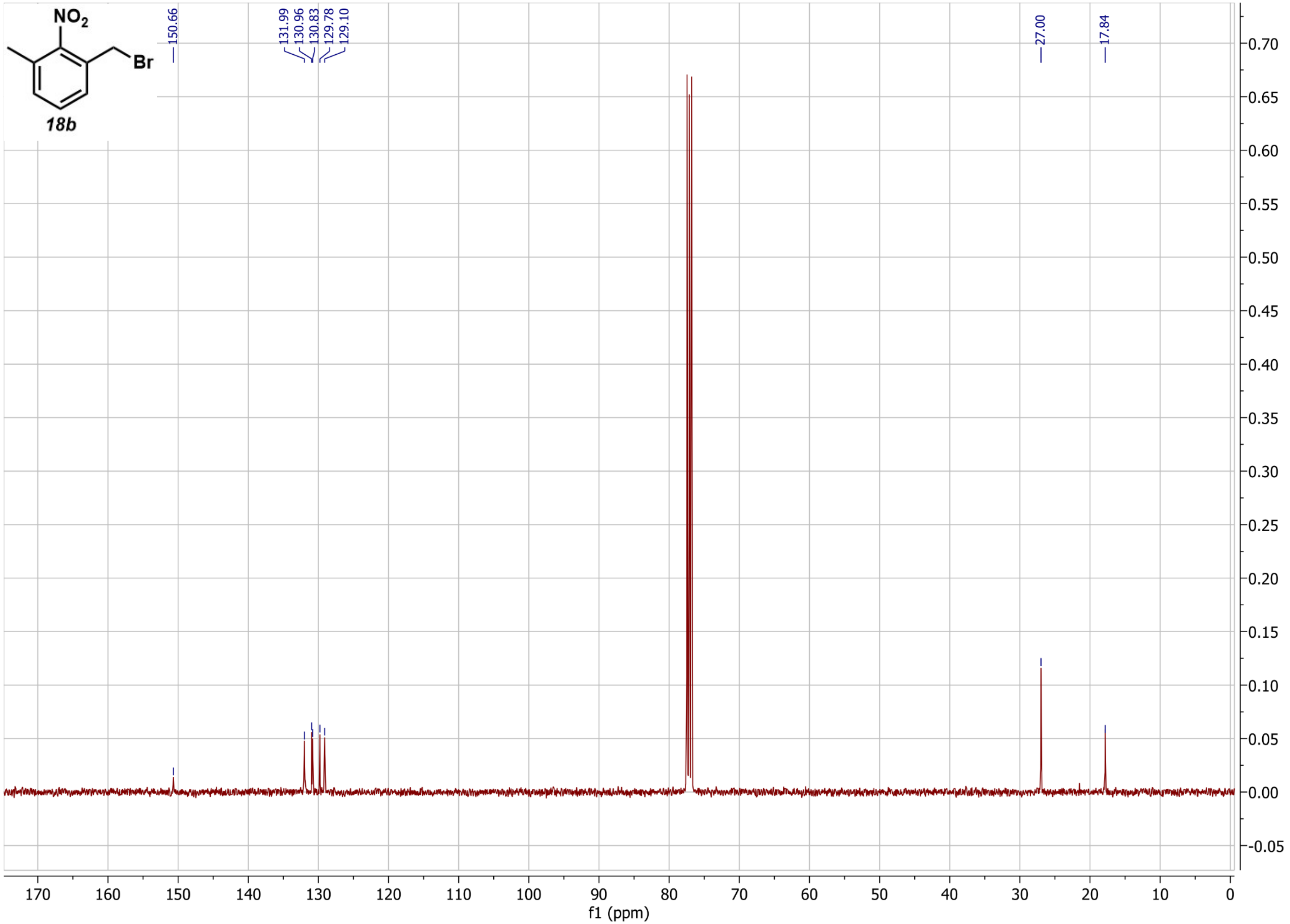

$^{13}C\{1H\}$ NMR. Solvent: $CDCl_3$. B_0 = 100 MHz.

*Compound **18f***

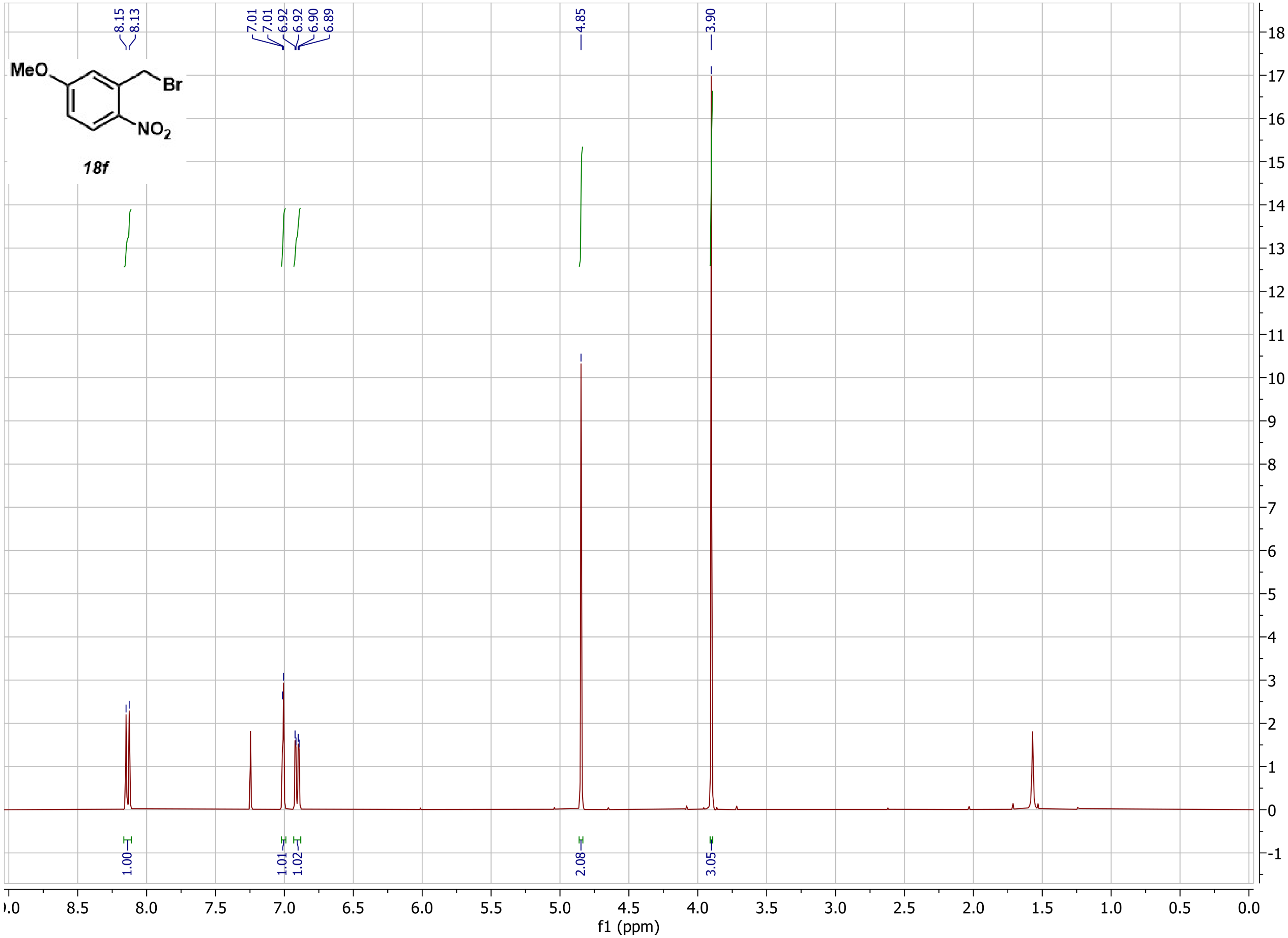

^{1}H NMR. Solvent: $CDCl_3$. B_0 = 400 MHz.

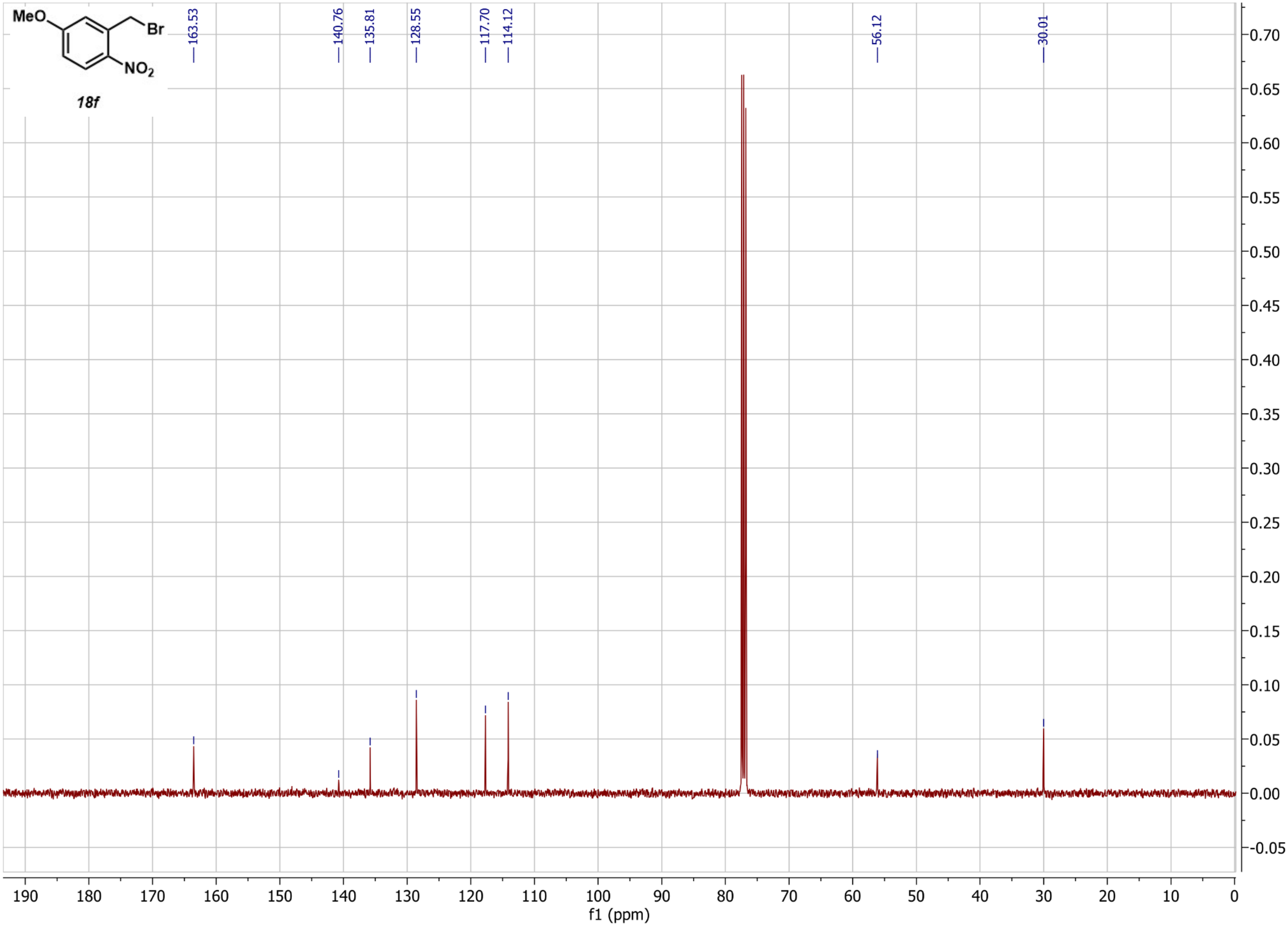

$^{13}C\{^1H\}$ NMR. Solvent: $CDCl_3$. B_0 = 100 MHz.

*Compound **19a***

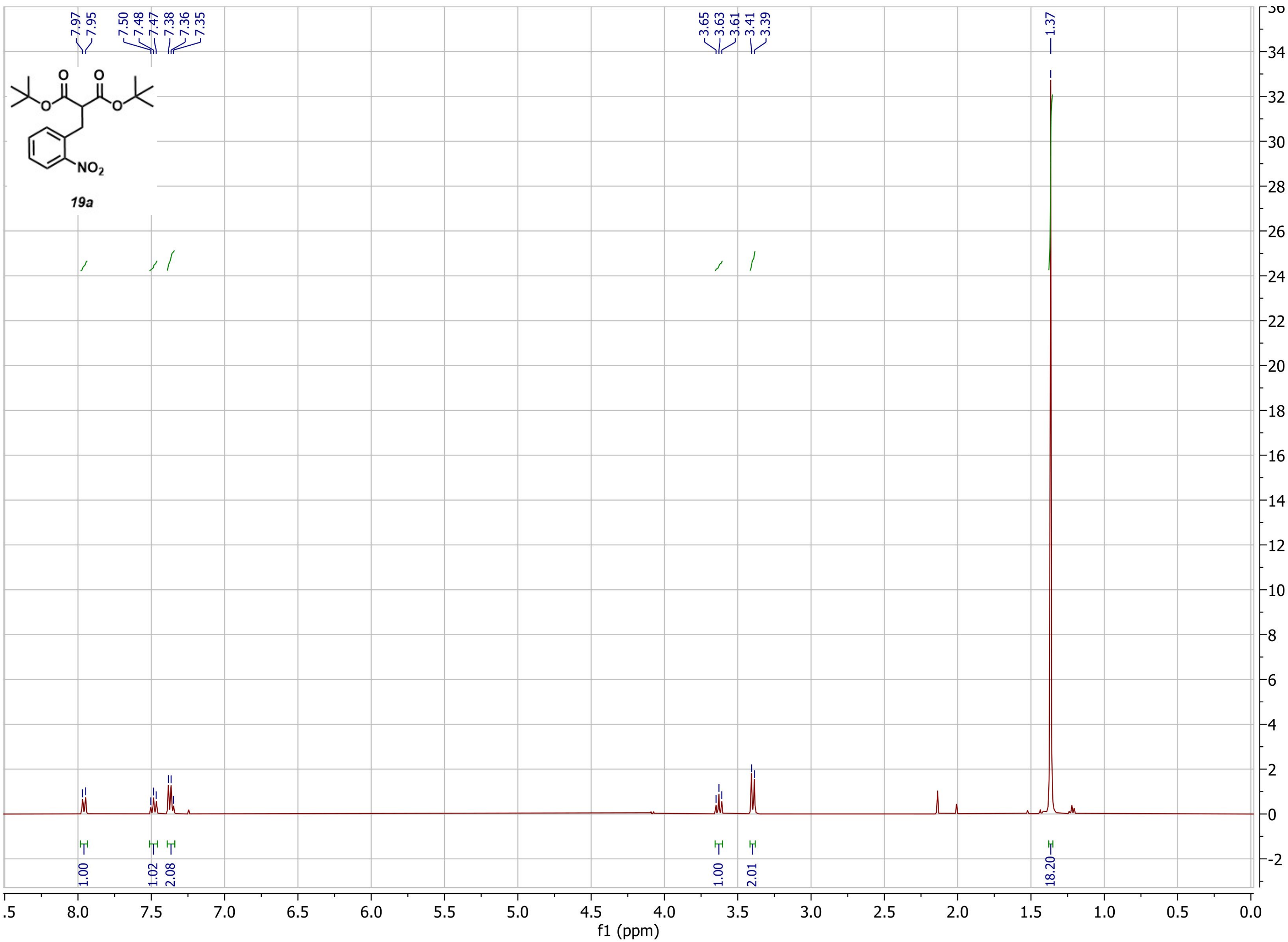

^{1}H NMR. Solvent: $CDCl_3$. B_0 = 400 MHz.

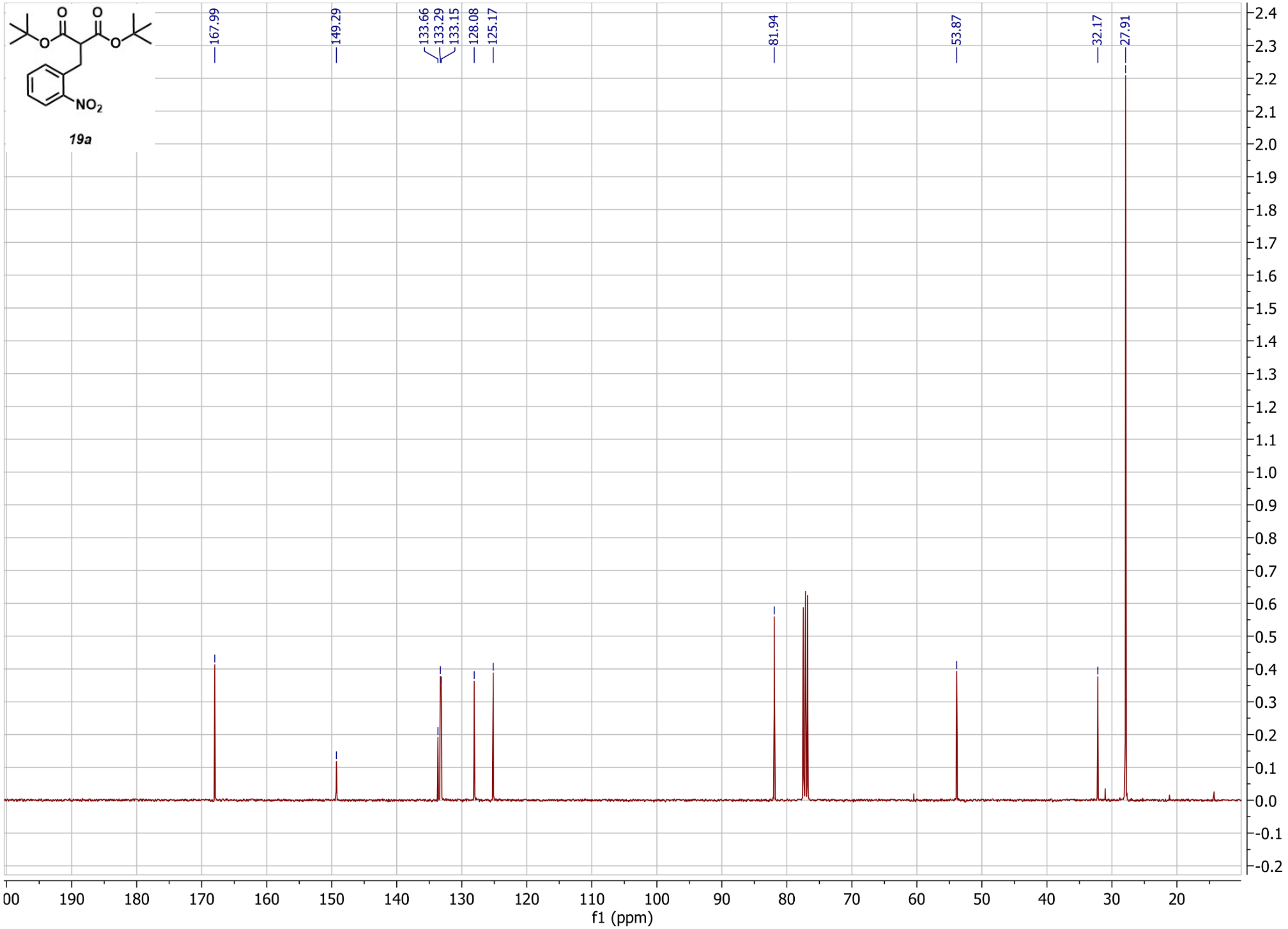

$^{13}C\{1H\}$ NMR. Solvent: $CDCl_3$. B_0 = 100 MHz.

*Compound **19b***

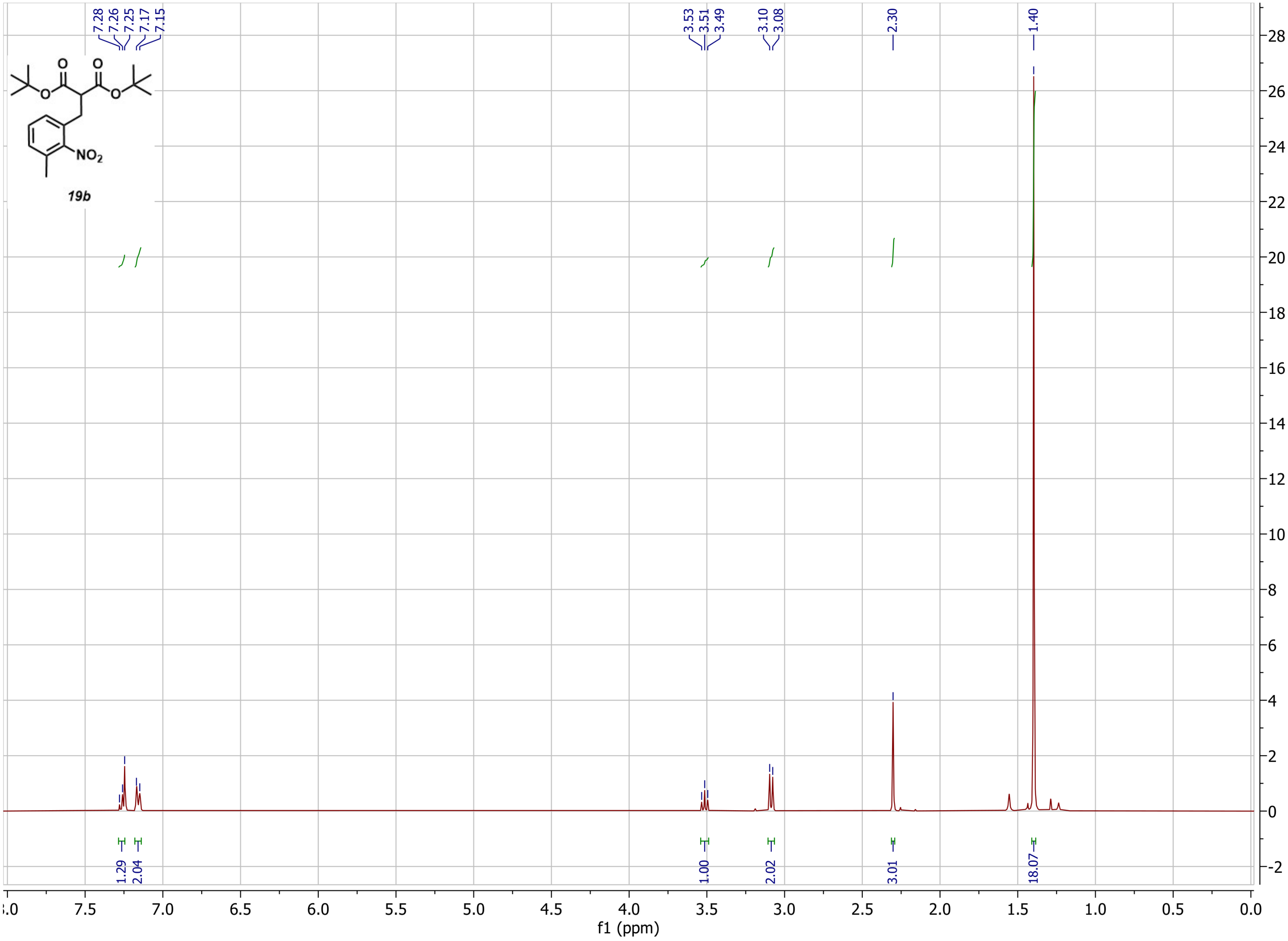

^{1}H NMR. Solvent: $CDCl_3$. B_0 = 400 MHz.

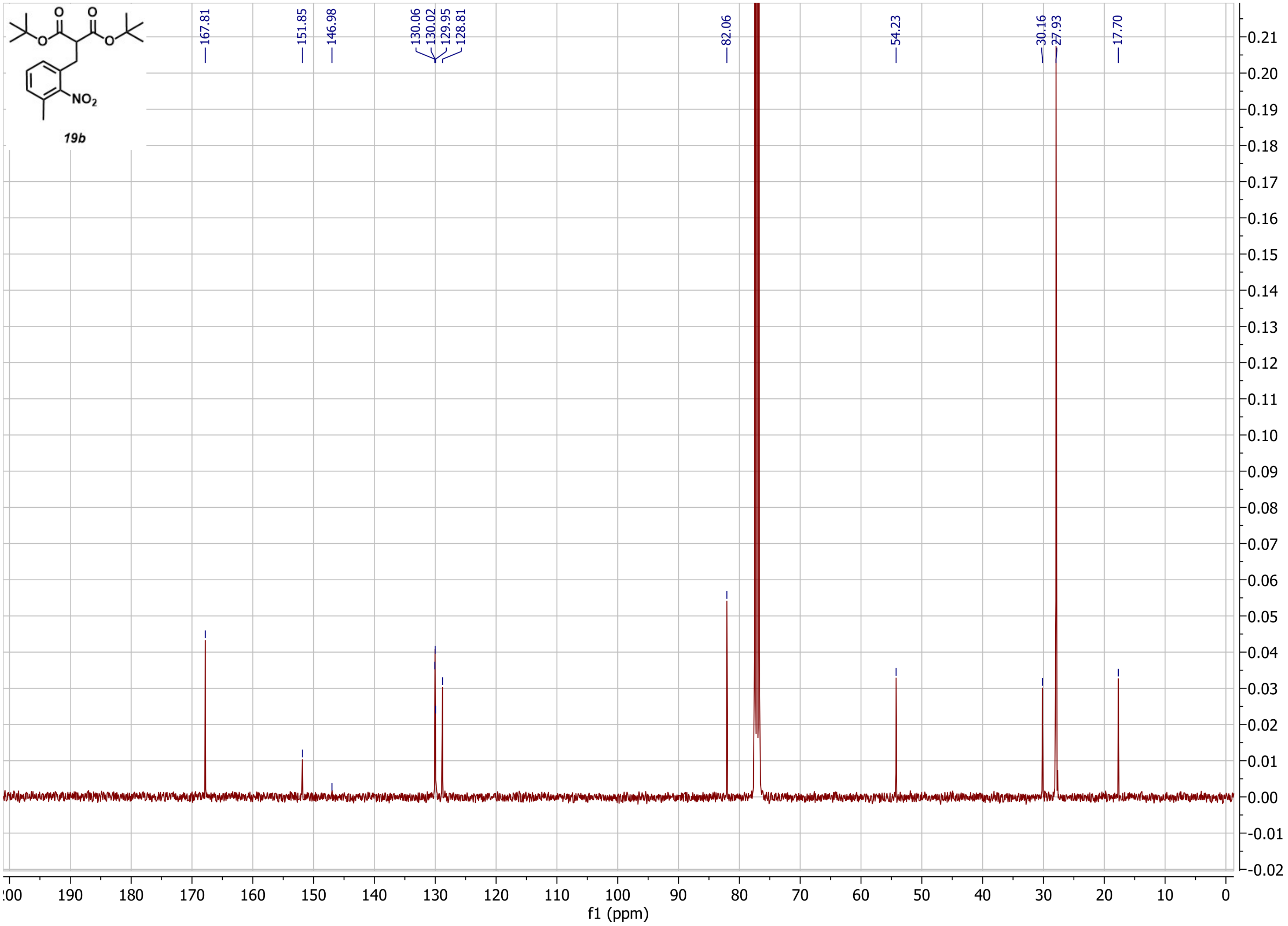

$^{13}C\{1H\}$ NMR. Solvent: $CDCl_3$. B_0 = 100 MHz.

Compound ***19c***

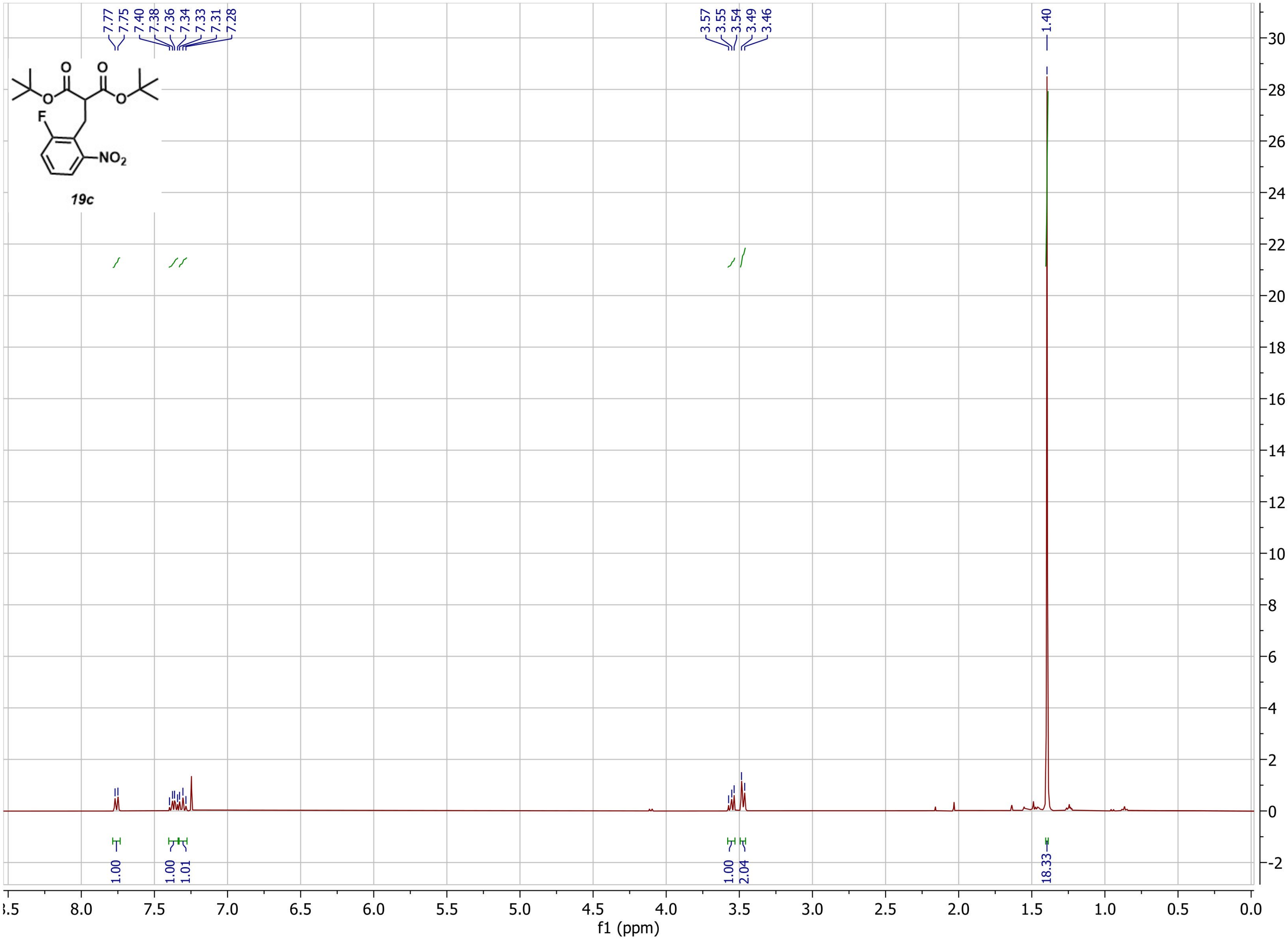

^{1}H NMR. Solvent: $CDCl_3$. B_0 = 400 MHz.

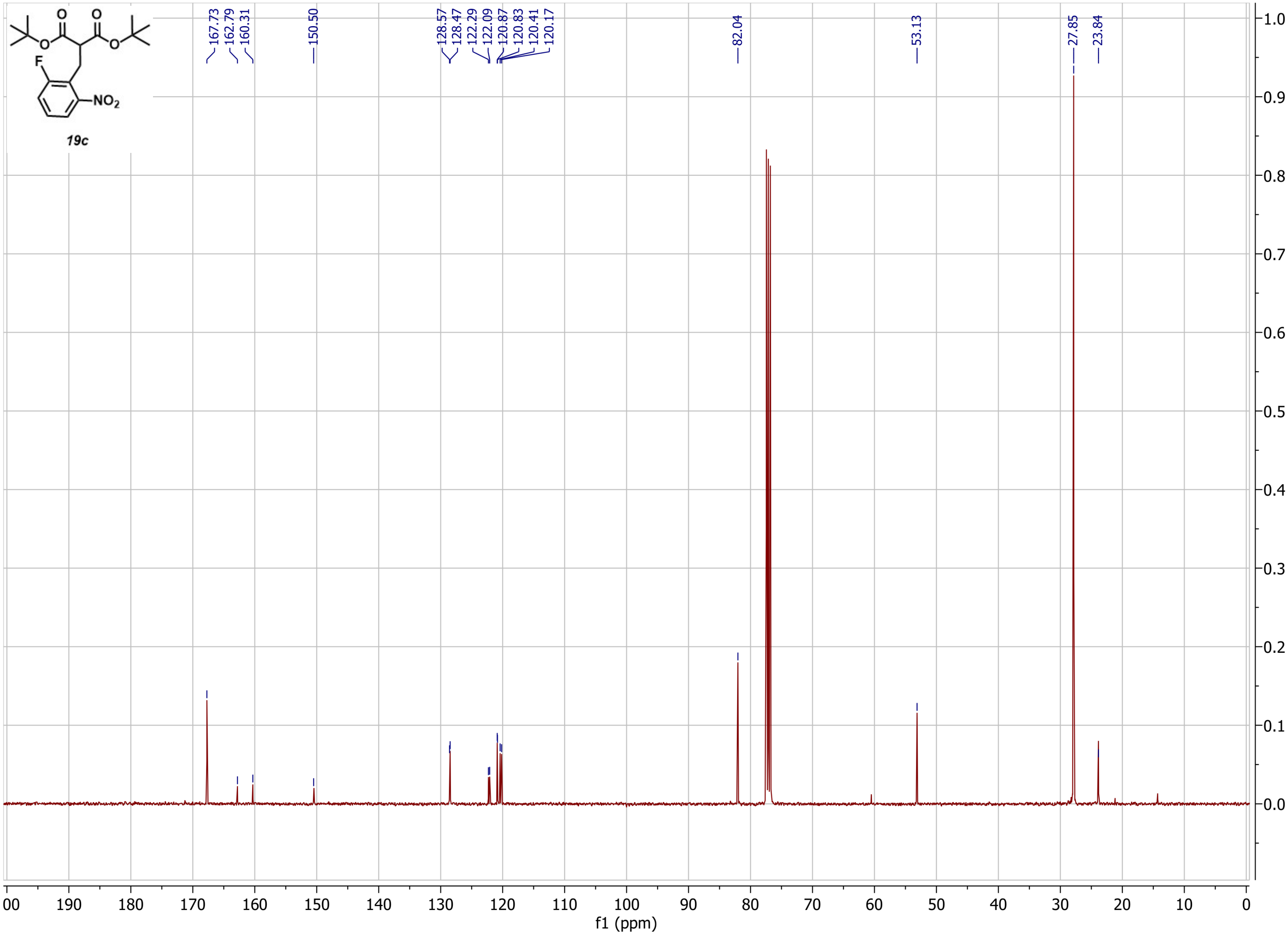

$^{13}C\{^1H\}$ NMR. Solvent: $CDCl_3$. B_0 = 100 MHz.

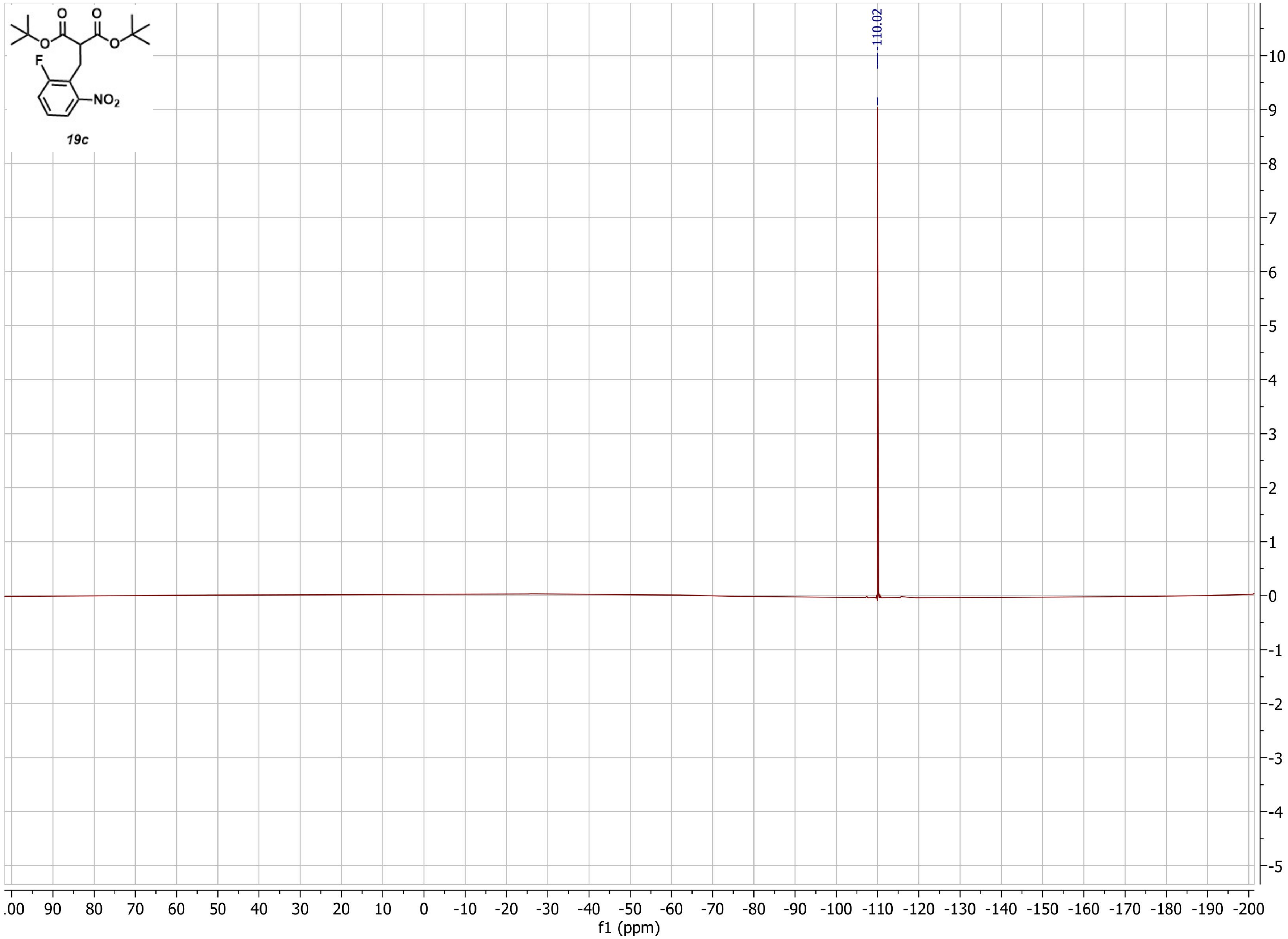

^{19}F NMR. Solvent: $CDCl_3$. B_0 = 400 MHz.

*Compound **19e***

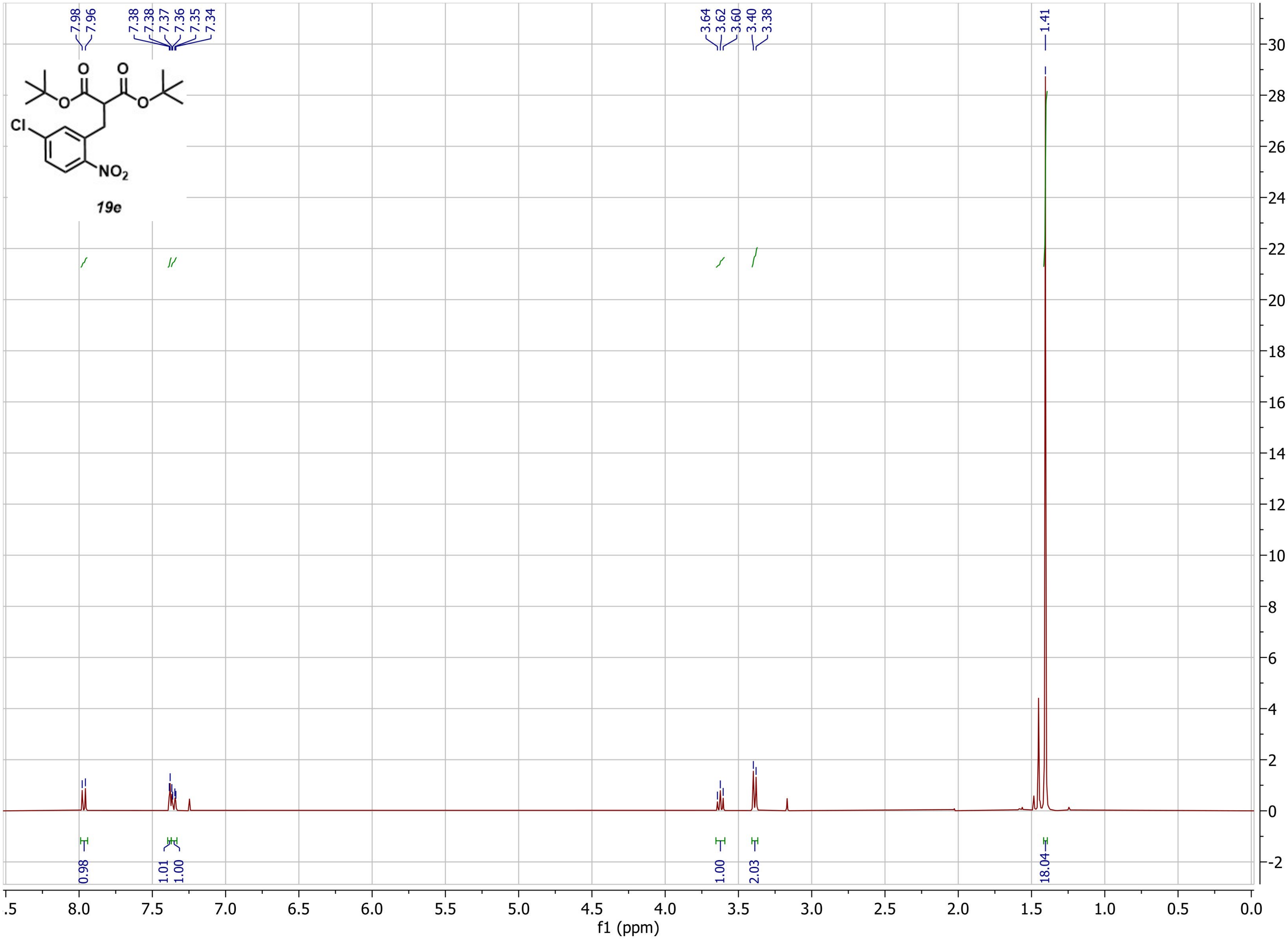

^{1}H NMR. Solvent: $CDCl_3$. B_0 = 400 MHz.

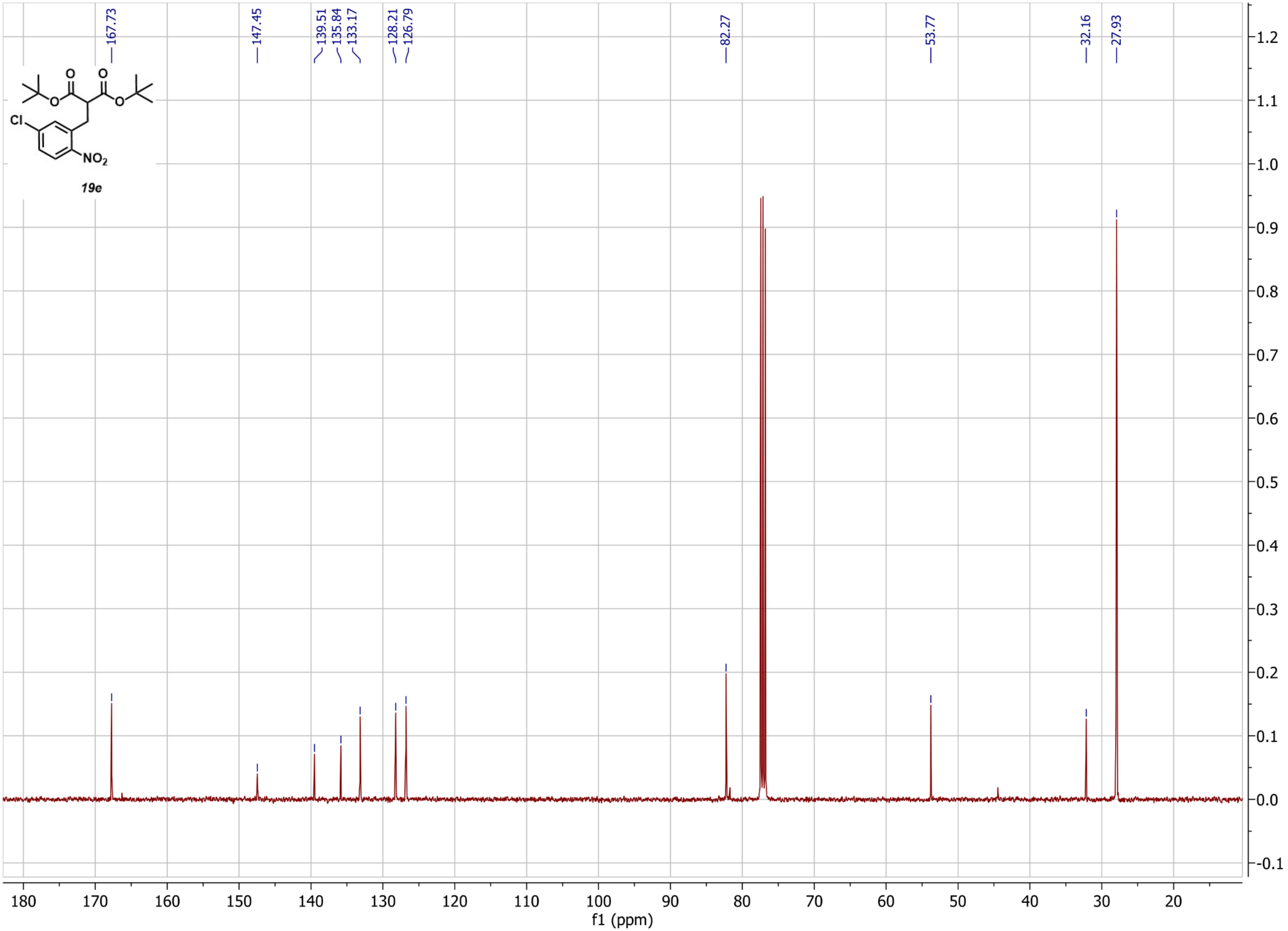

$^{13}C\{1H\}$ NMR. Solvent: $CDCl_3$. B_0 = 100 MHz.

*Compound **31a***

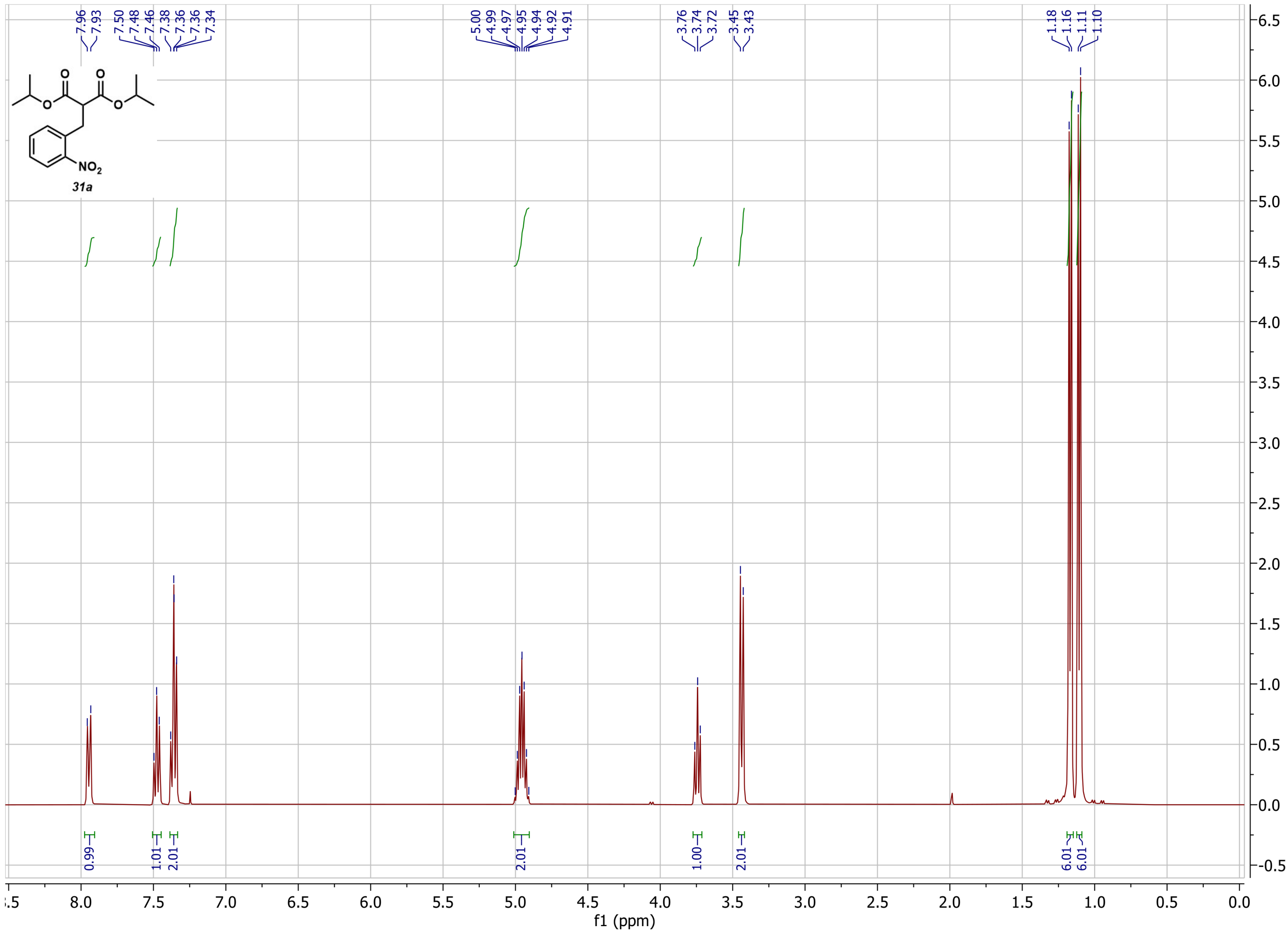

^{1}H NMR. Solvent: $CDCl_3$. B_0 = 400 MHz.

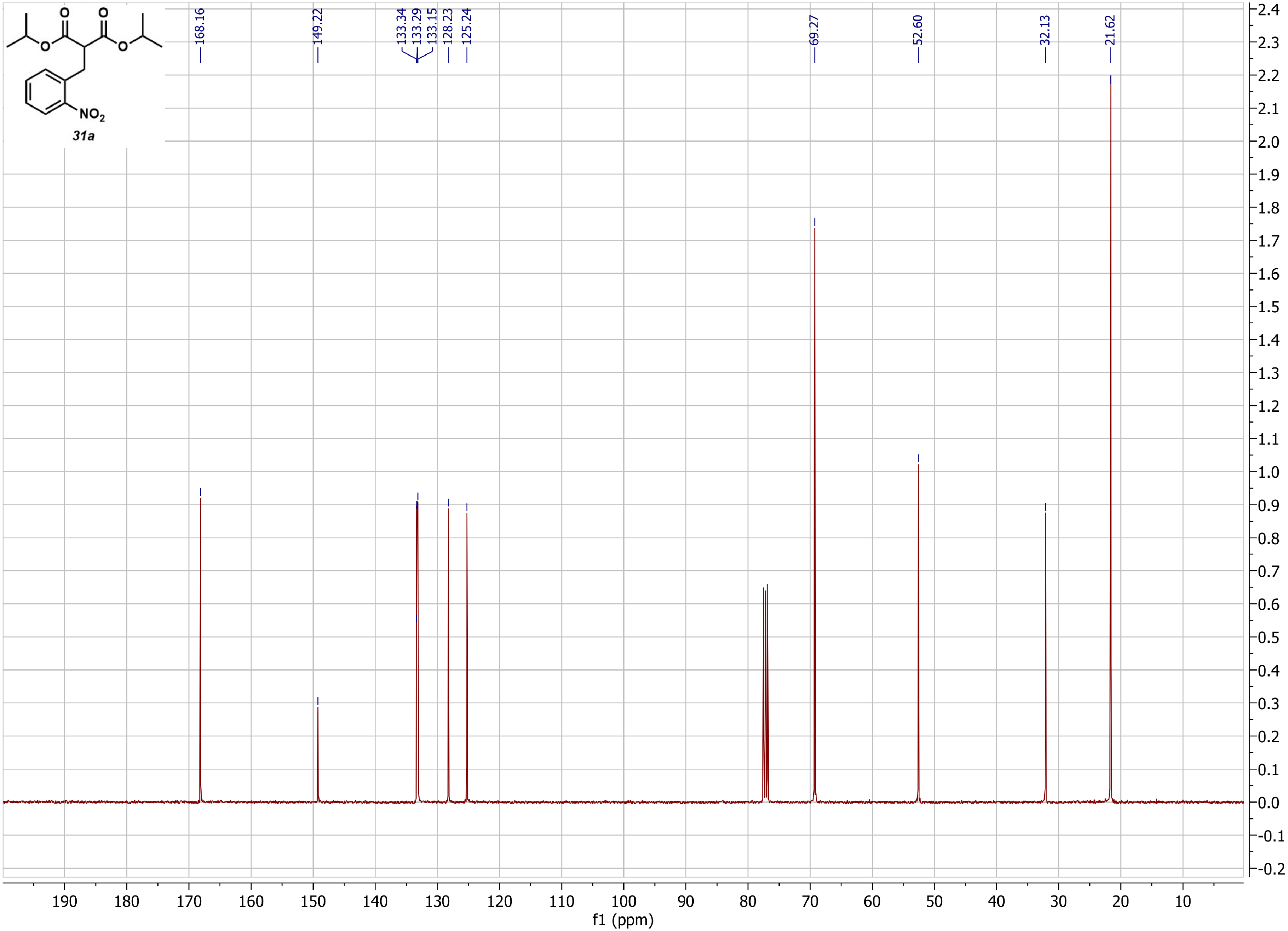

$^{13}C\{^1H\}$ NMR. Solvent: $CDCl_3$. B_0 = 100 MHz.

Compound ***20da***

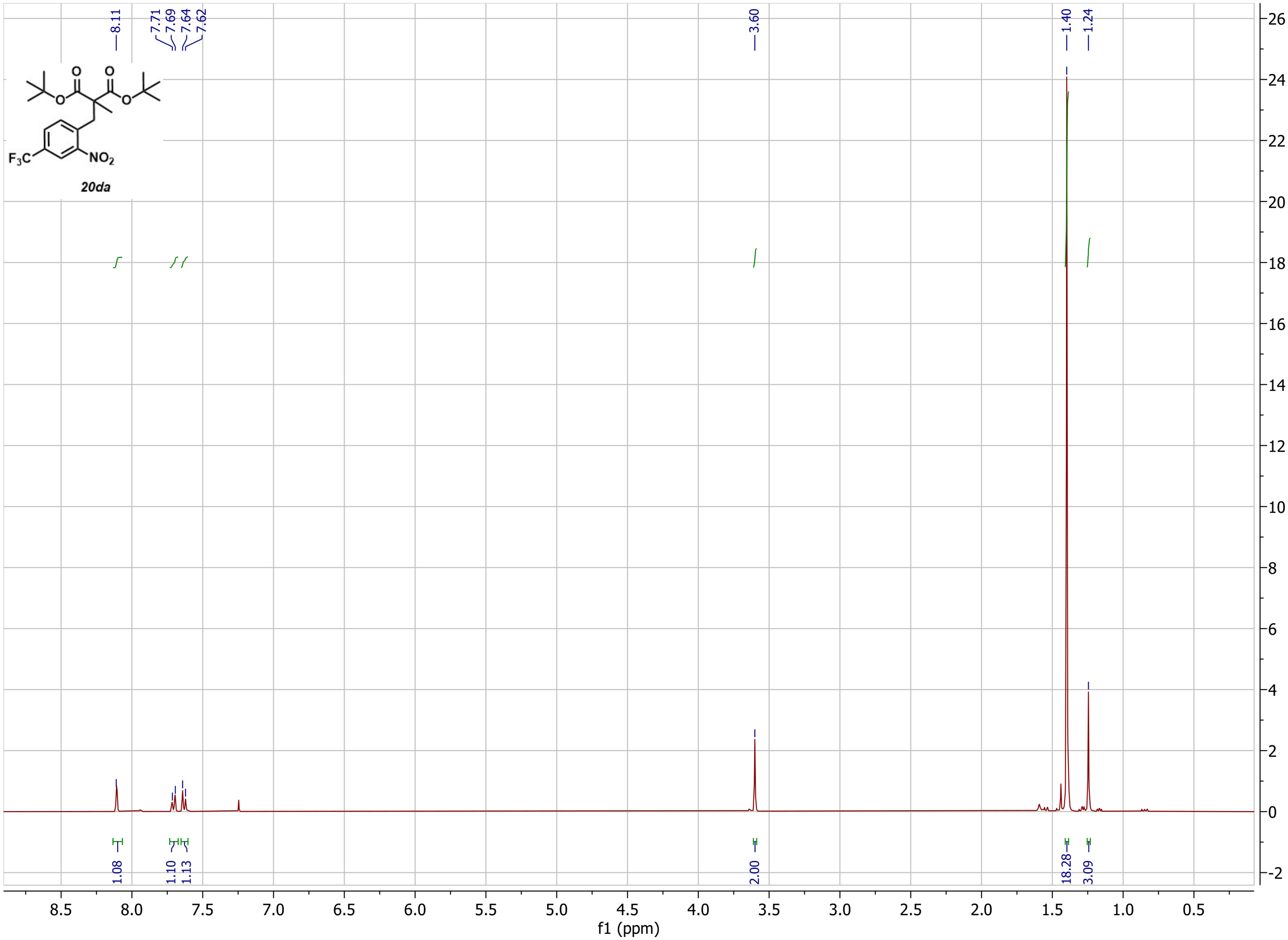

^{1}H NMR. Solvent: $CDCl_3$. B_0 = 400 MHz.

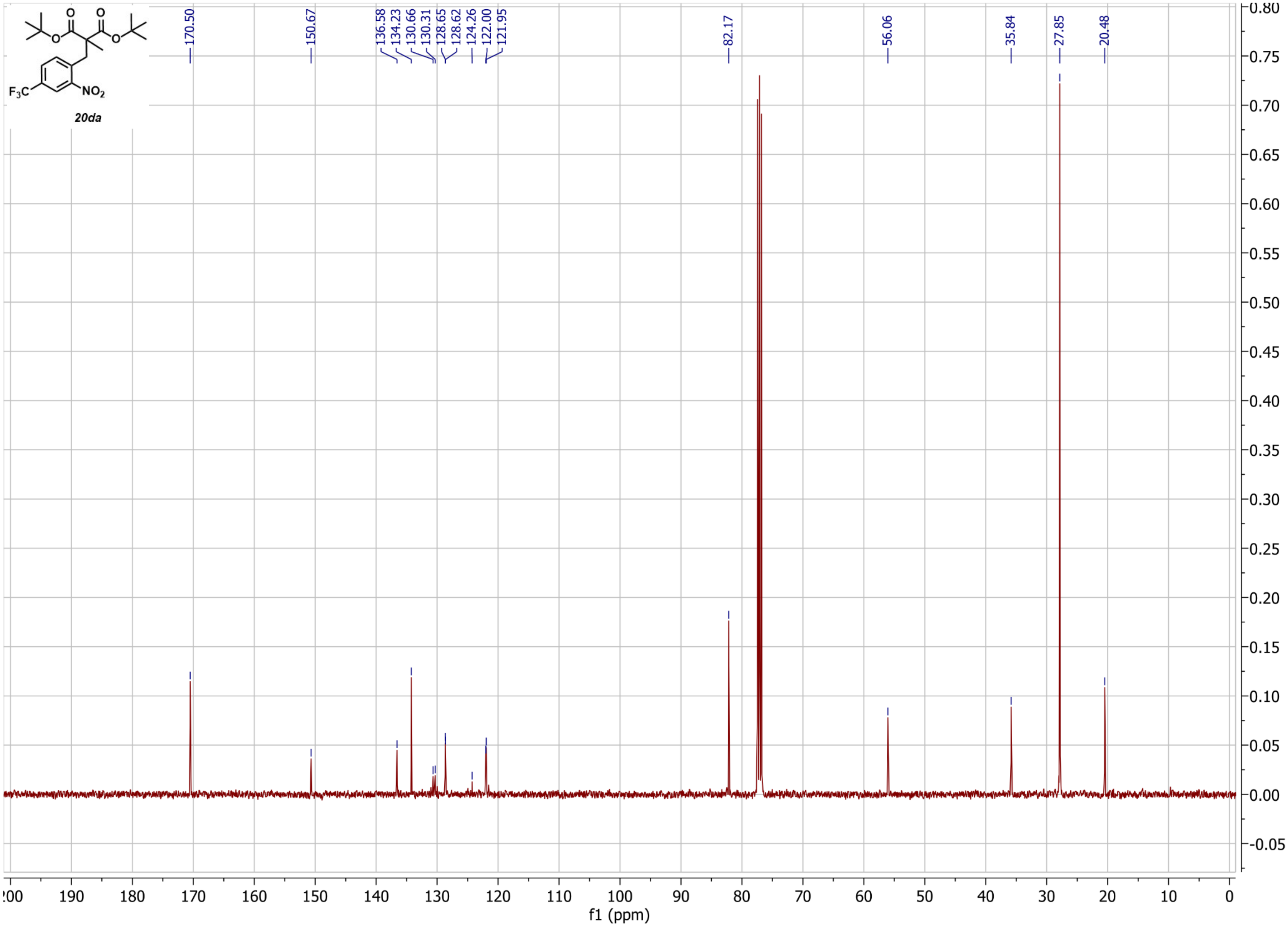

$^{13}C\{^1H\}$ NMR. Solvent: $CDCl_3$. B_0 = 100 MHz.

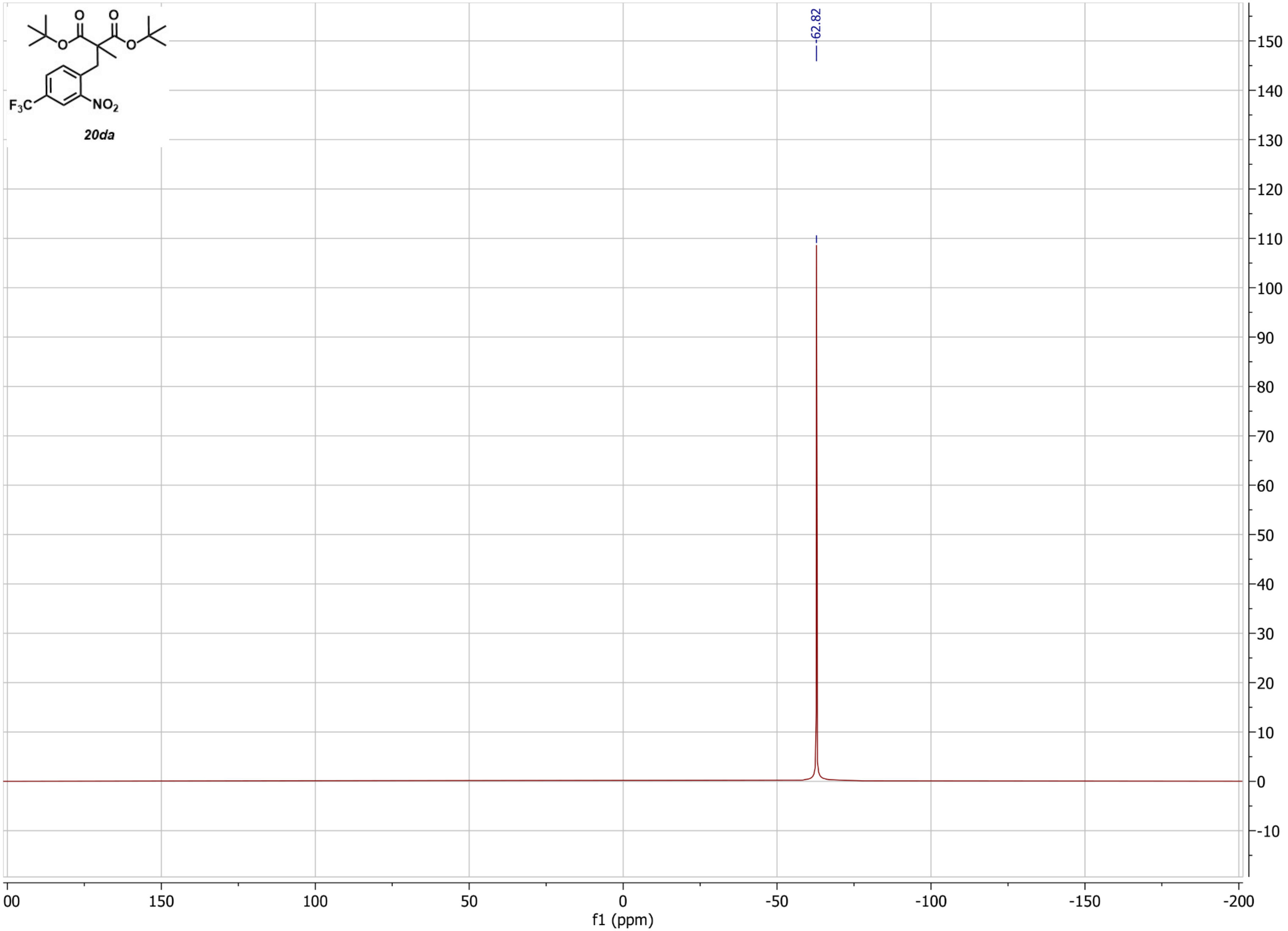

^{19}F NMR. Solvent: $CDCl_3$. B_0 = 376 MHz.

Compound ***20fa***

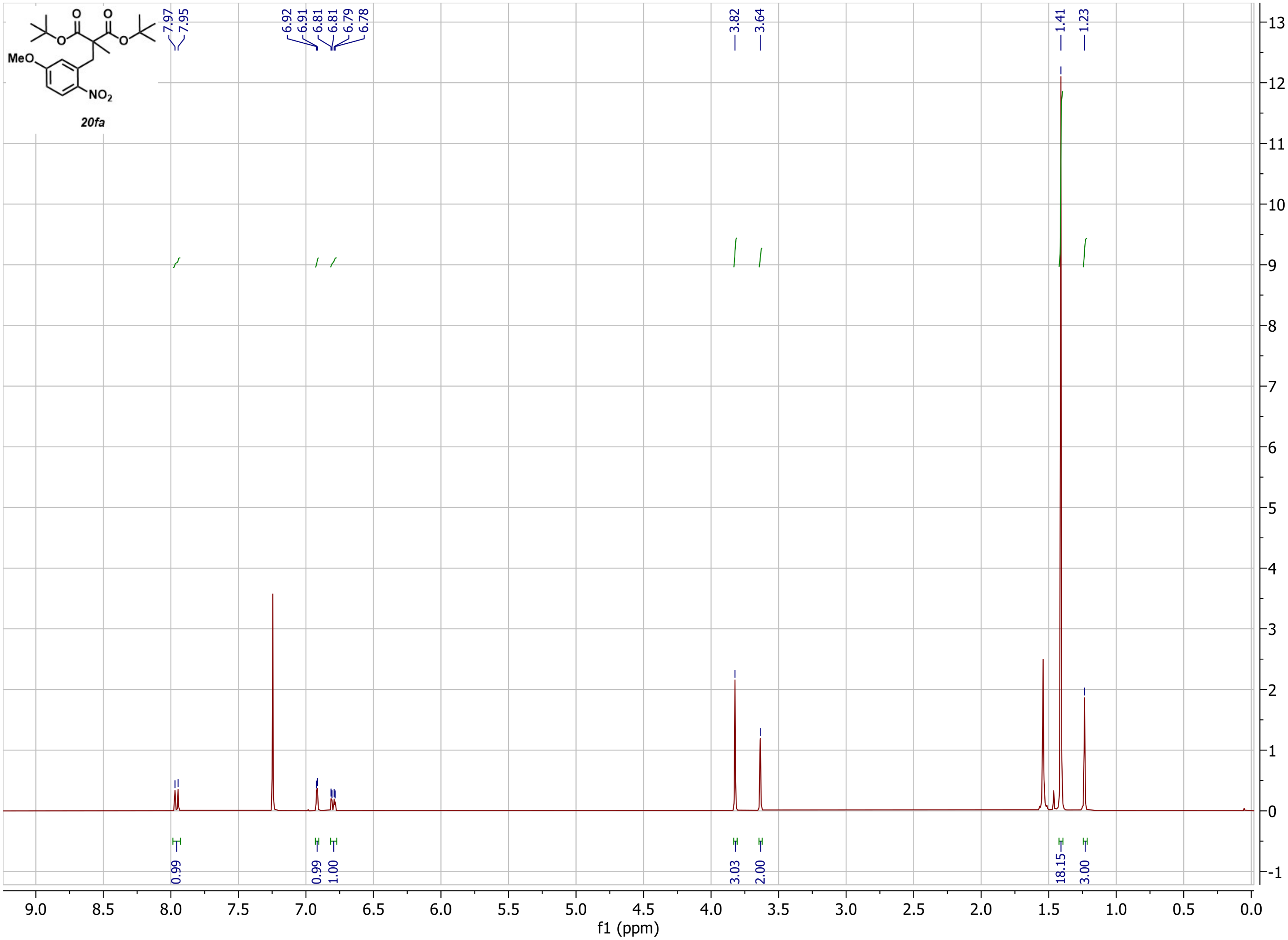

^{1}H NMR. Solvent: $CDCl_3$. B_0 = 400 MHz.

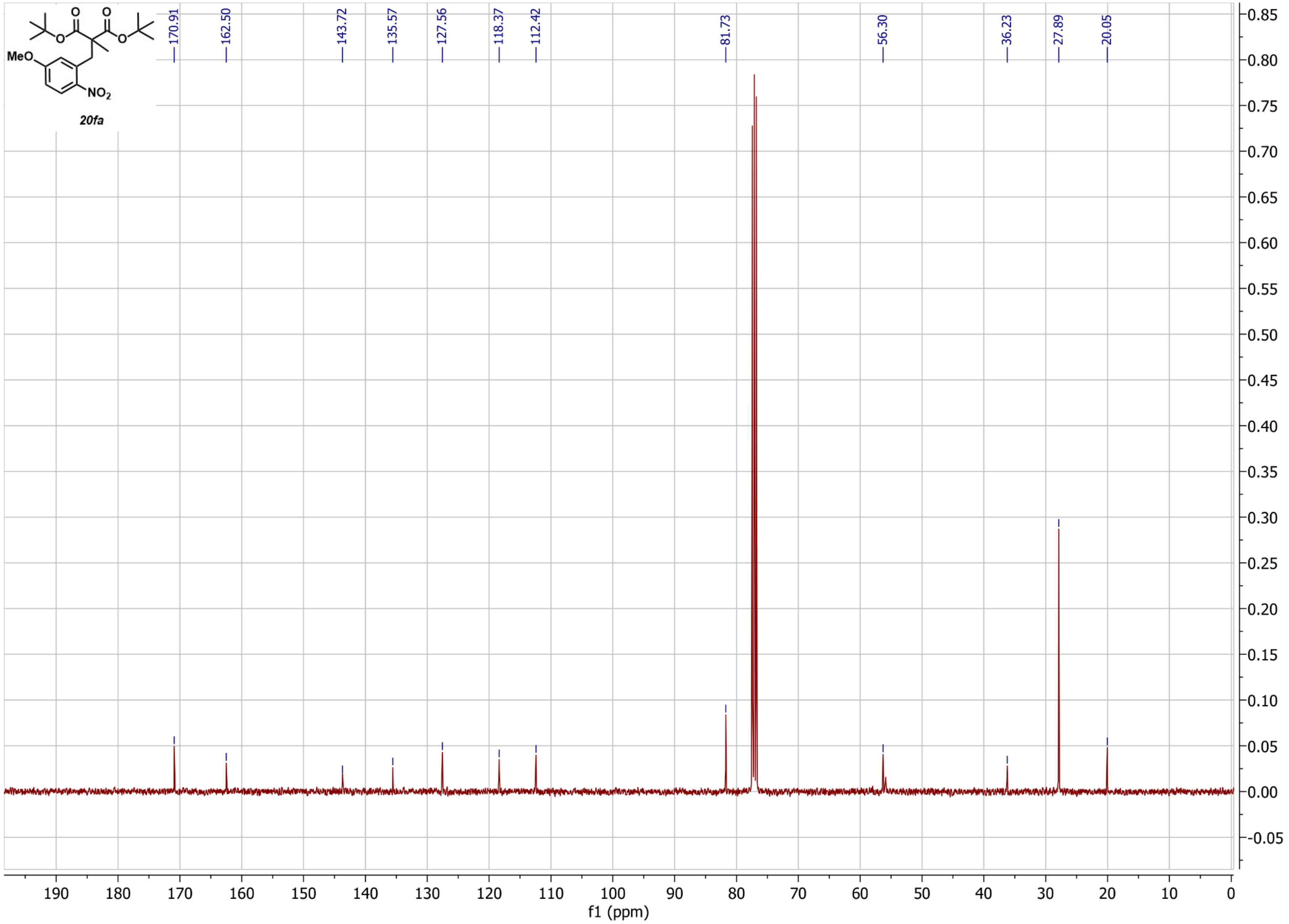

$^{13}C\{^1H\}$ NMR. Solvent: $CDCl_3$. B_0 = 100 MHz.

Compound ***20aa***

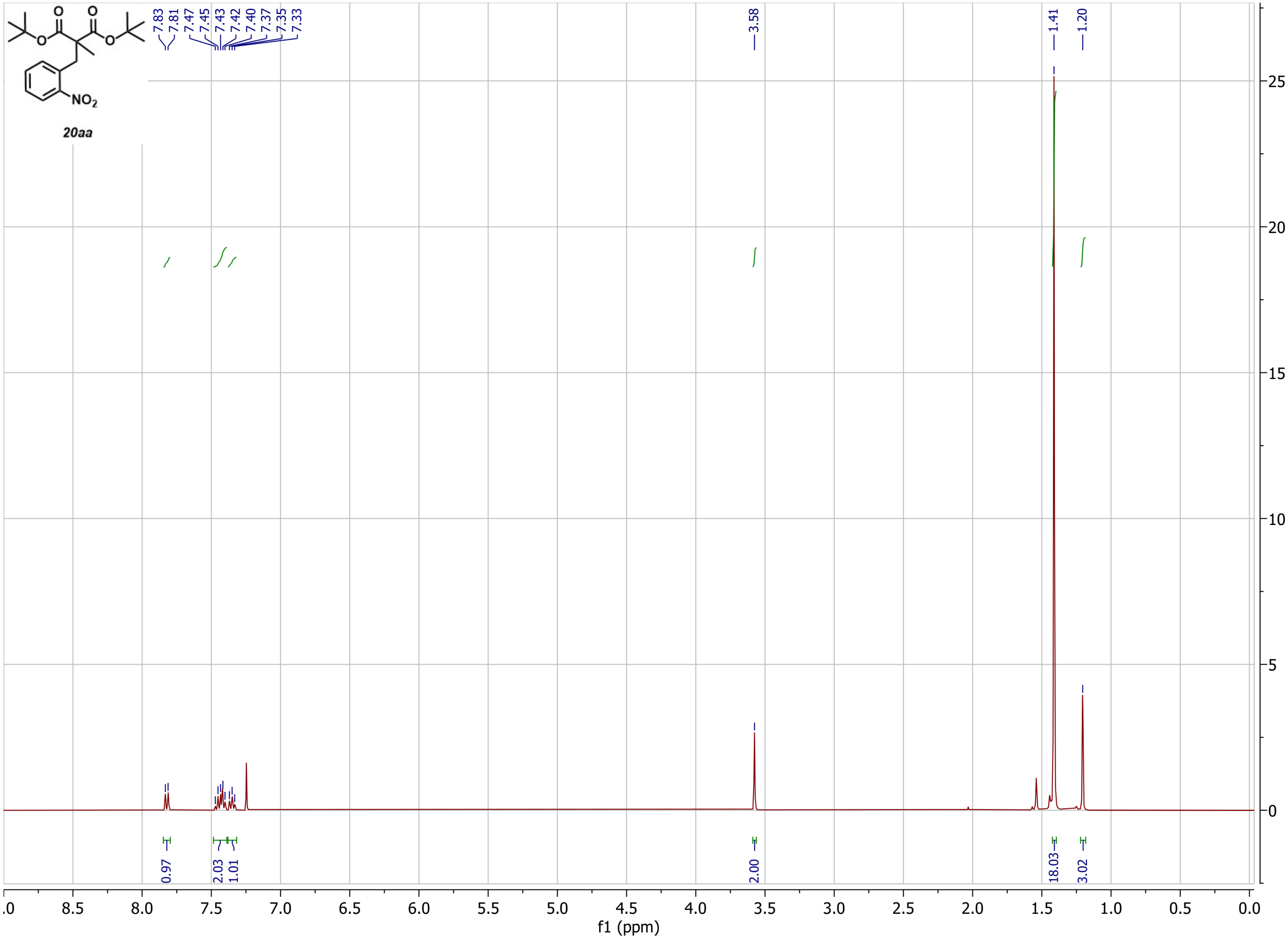

^{1}H NMR. Solvent: $CDCl_3$. B_0 = 400 MHz.

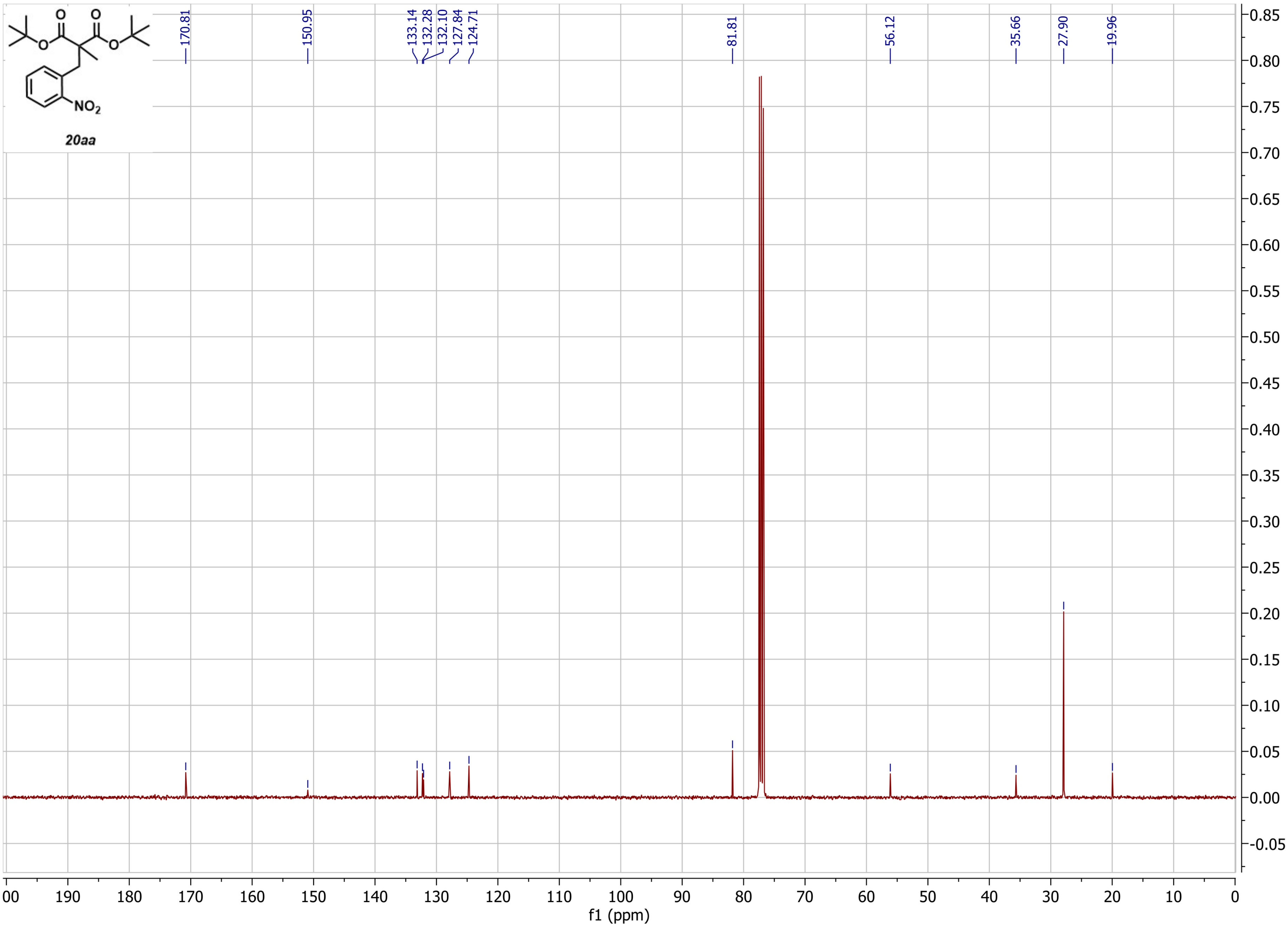

$^{13}C\{1H\}$ NMR. Solvent: $CDCl_3$. B_0 = 100 MHz.

*Compound **32aa***

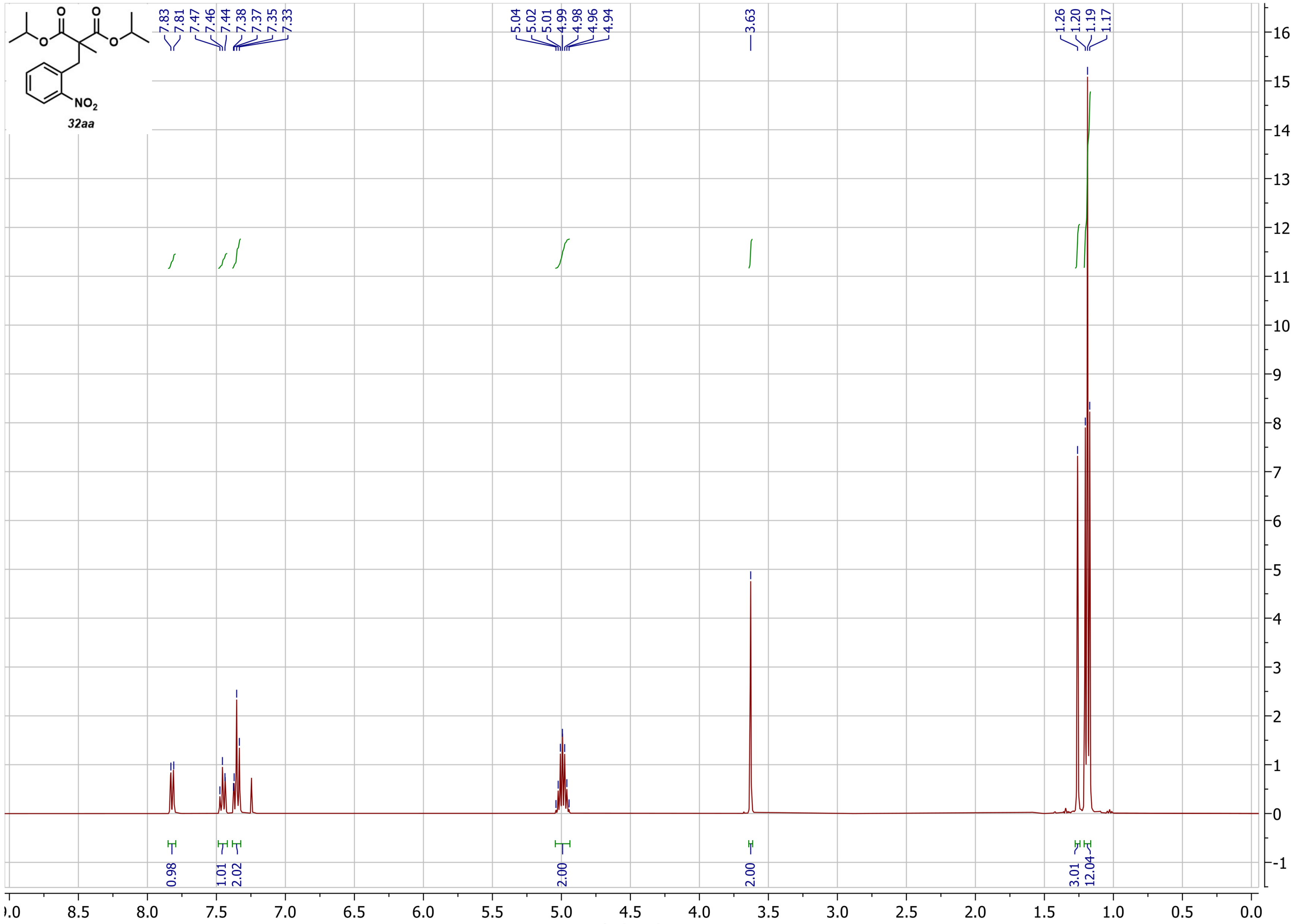

^1H NMR. Solvent: $CDCl_3$. B_0 = 400 MHz.

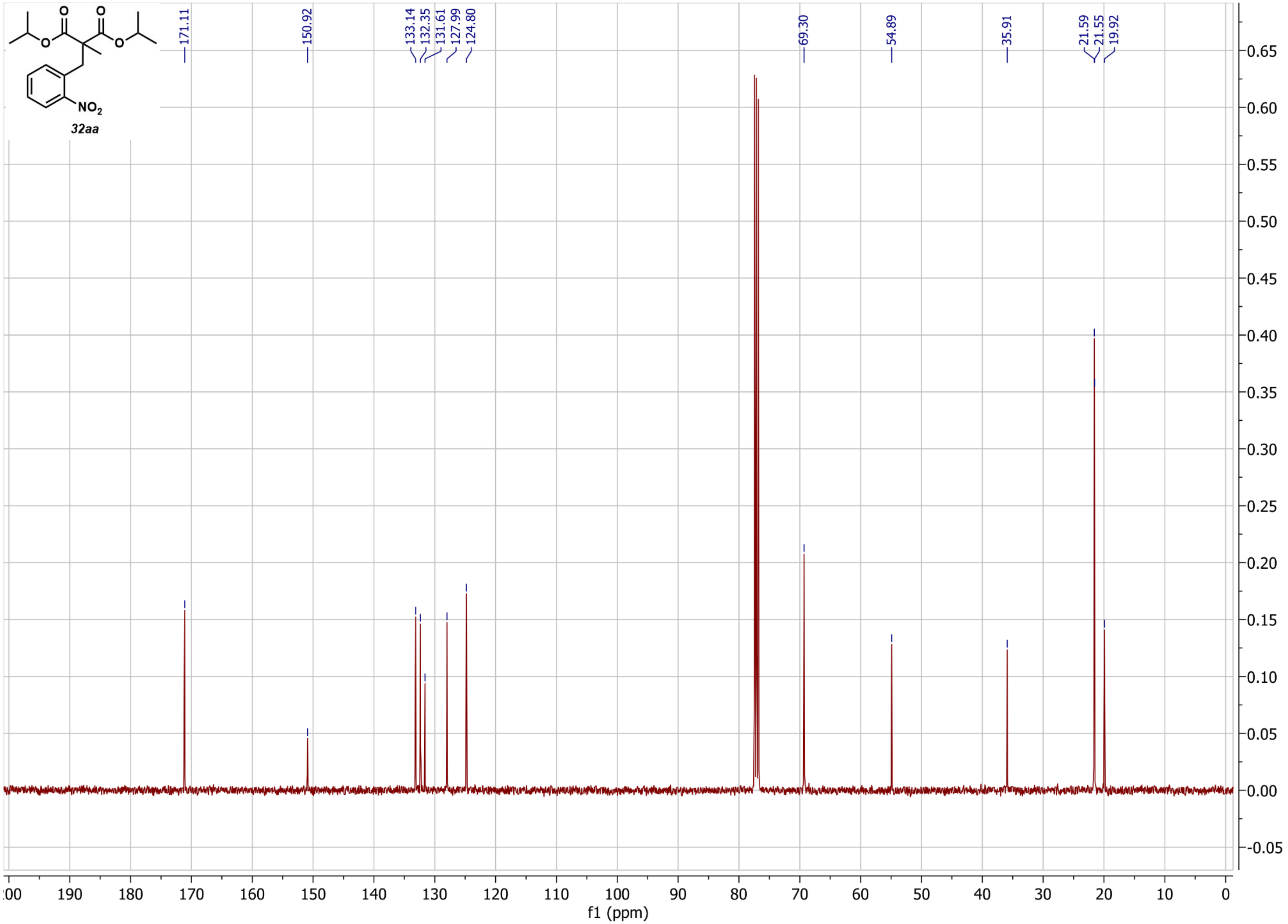

$^{13}C\{1H\}$ NMR. Solvent: $CDCl_3$. B_0 = 100 MHz.

*Compound **14aa***

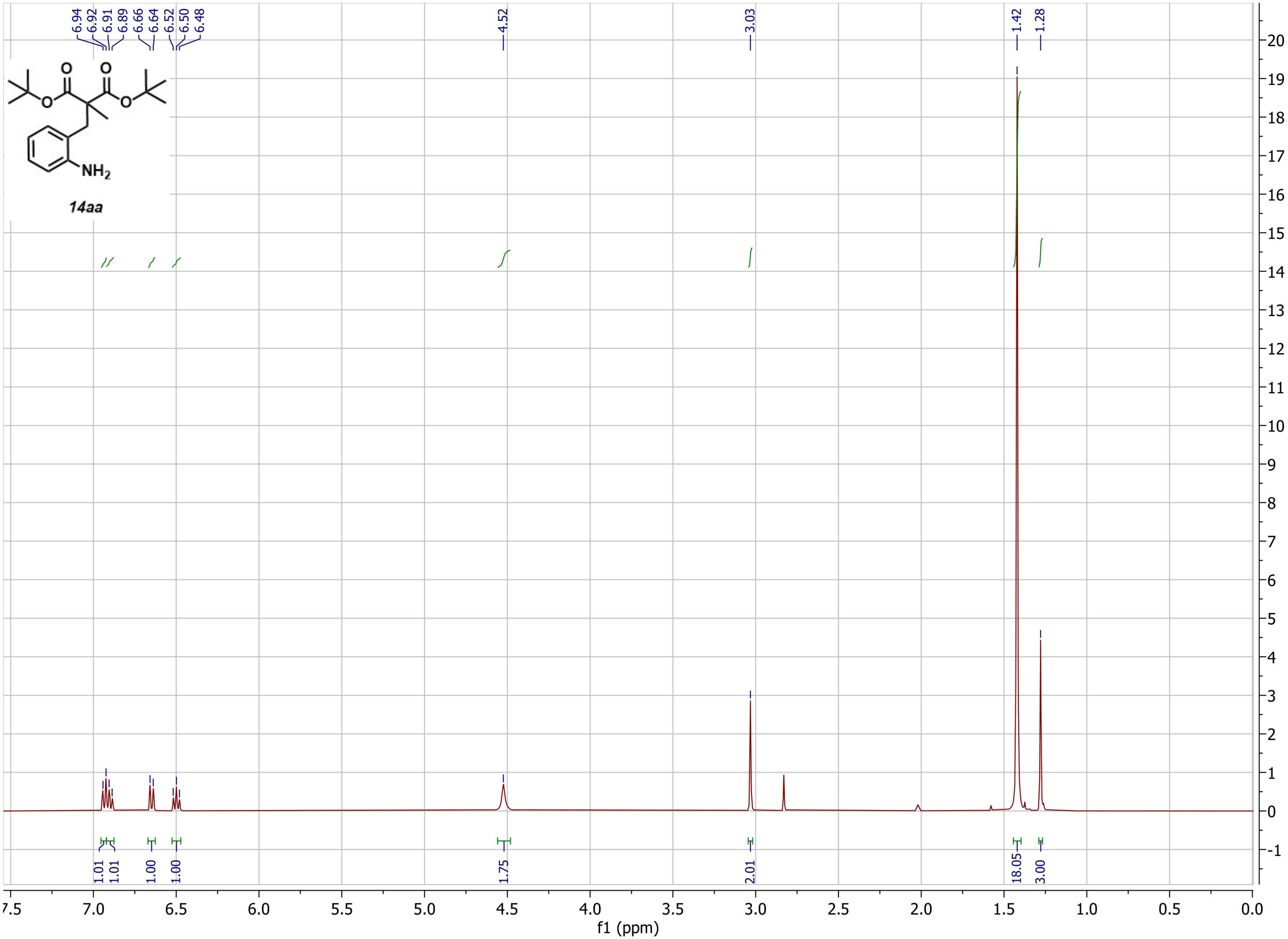

^{1}H NMR. Solvent: Acetone-d$_6$. B$_0$ = 400 MHz.

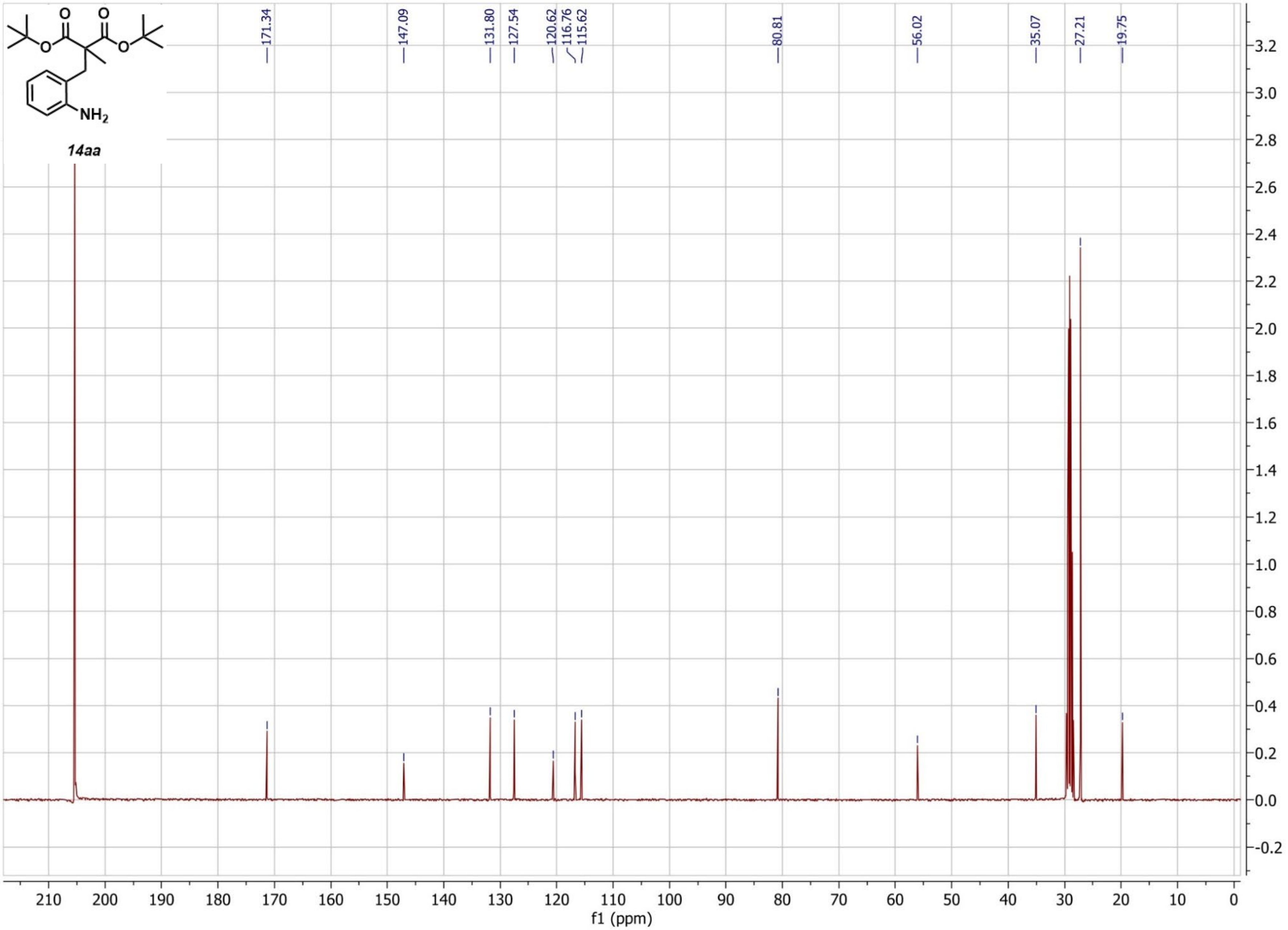

$^{13}C\{^1H\}$ NMR. Solvent: Acetone-d_6. B_0 = 100 MHz.

Compound ***14ab***

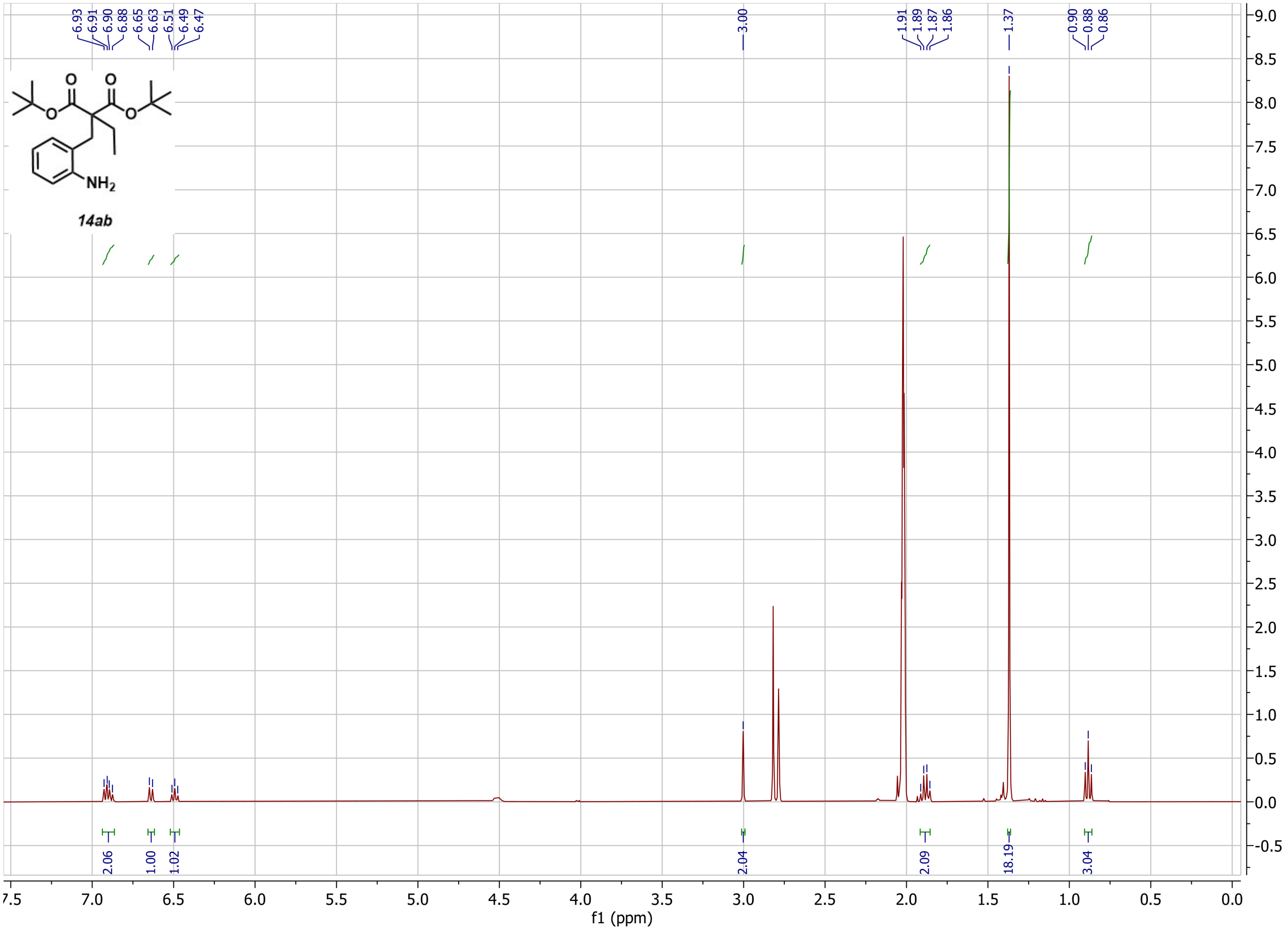

^{1}H NMR. Solvent: Acetone-d$_6$. B$_0$ = 400 MHz.

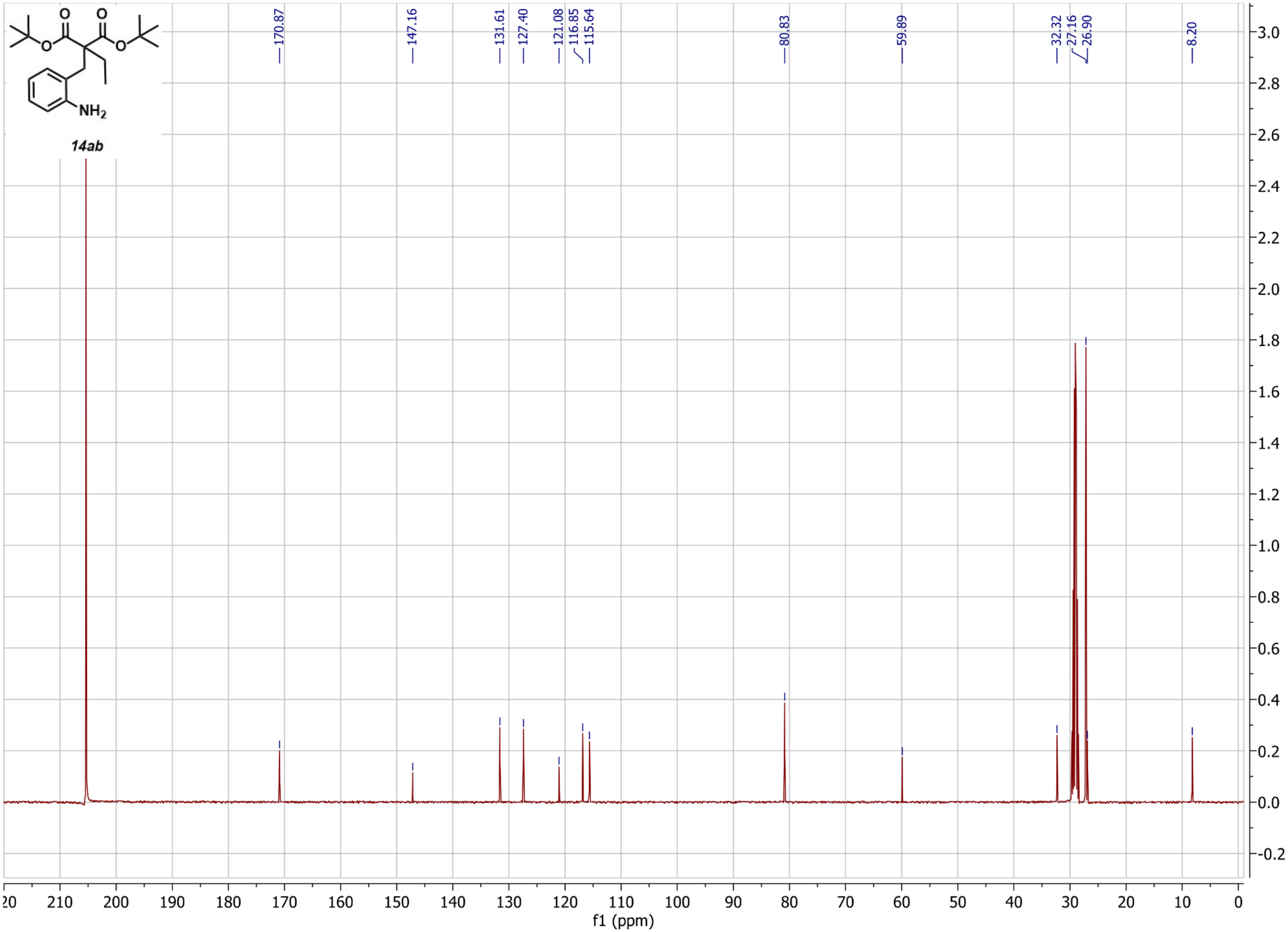

$^{13}C\{^1H\}$ NMR. Solvent: Acetone-d$_6$. B_0 =100 MHz.

*Compound **14ac***

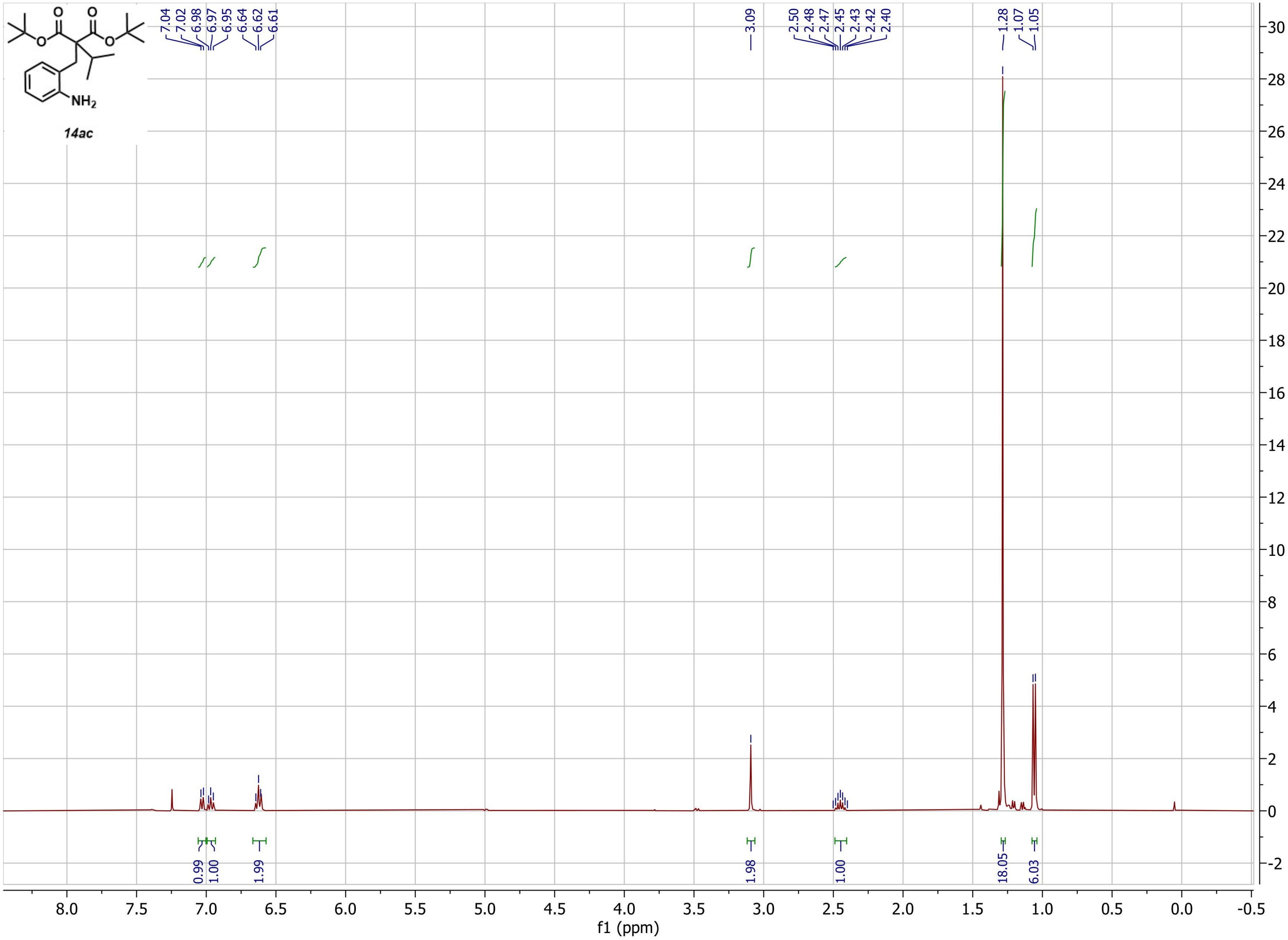

^1H NMR. Solvent: $CDCl_3$. B_0 = 400 MHz.

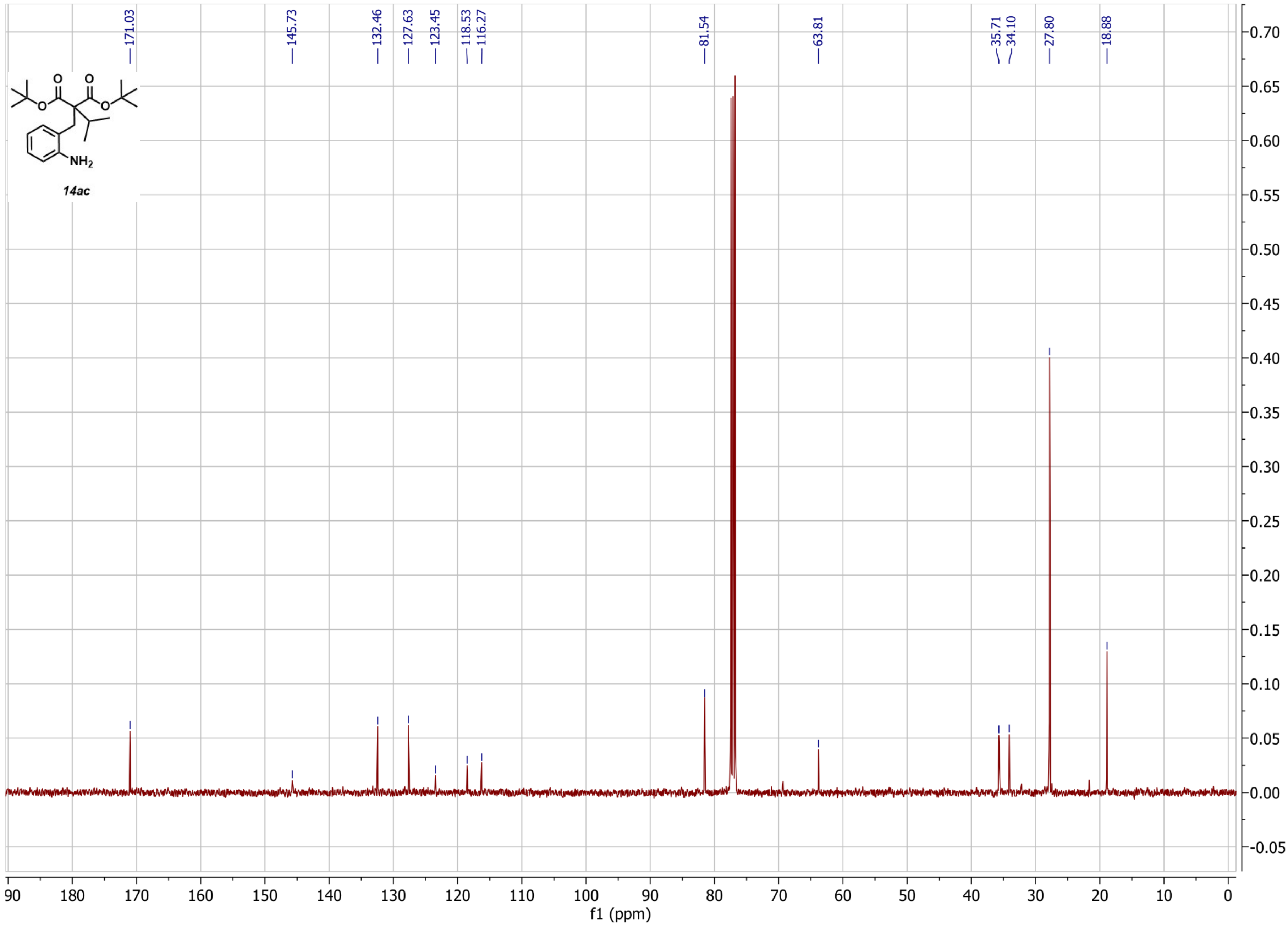

$^{13}C\{^1H\}$ NMR. Solvent: $CDCl_3$. B_0 = 100 MHz.

Compound ***14ad***

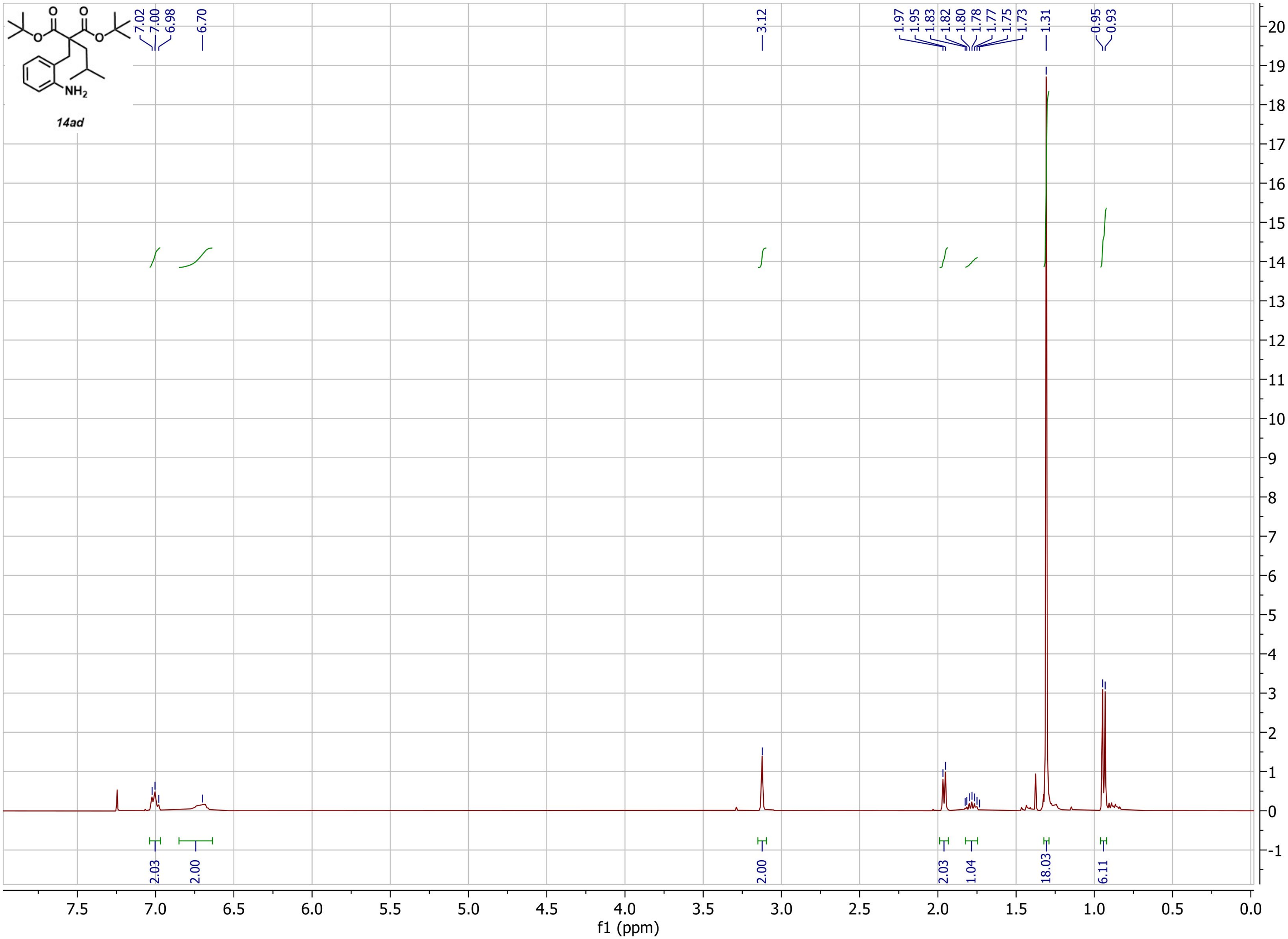

^{1}H NMR. Solvent: $CDCl_3$. B_0 = 400 MHz.

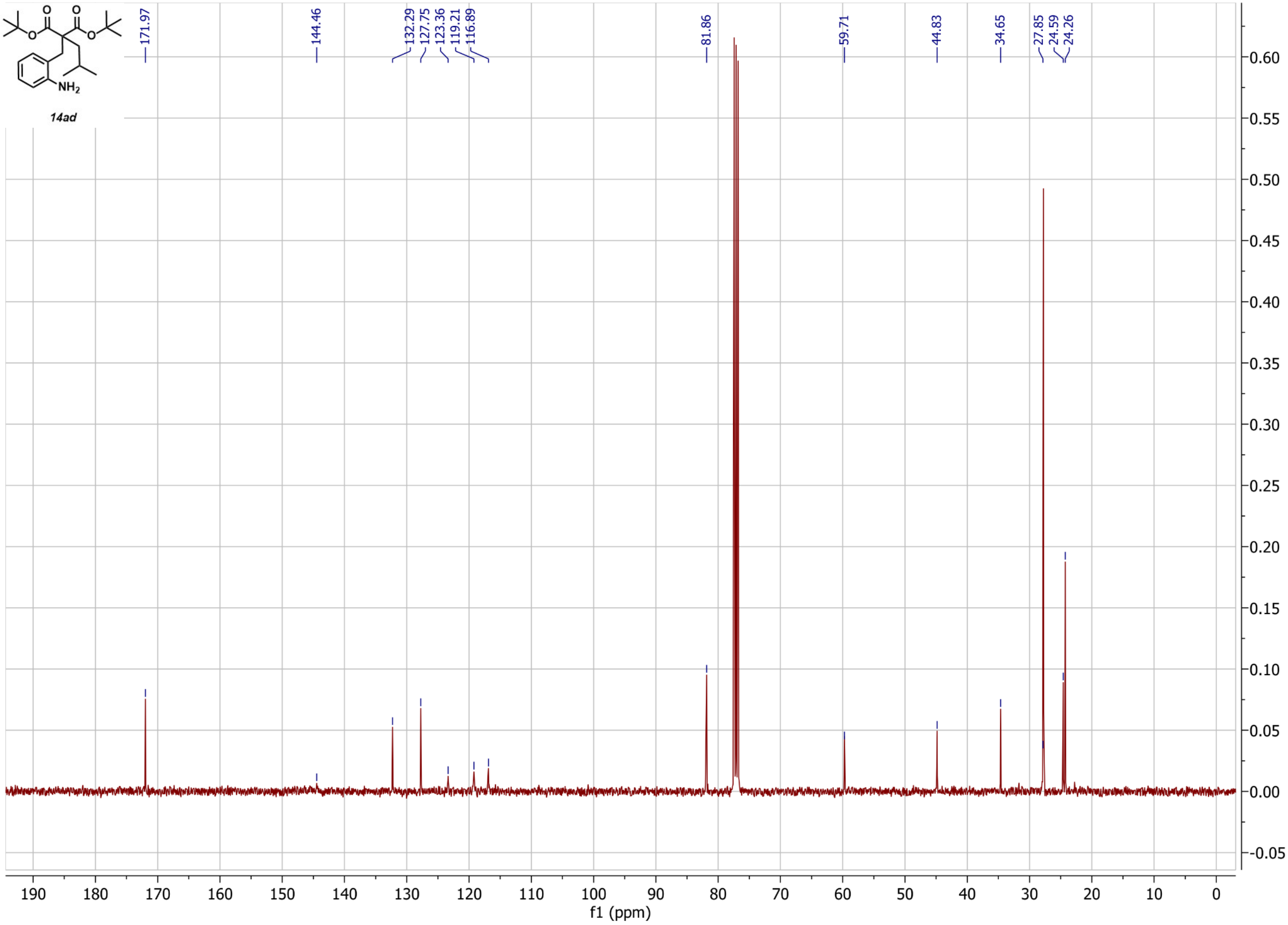

$^{13}C\{^1H\}$ NMR. Solvent: $CDCl_3$. B_0 = 100 MHz.

Compound ***14ae***

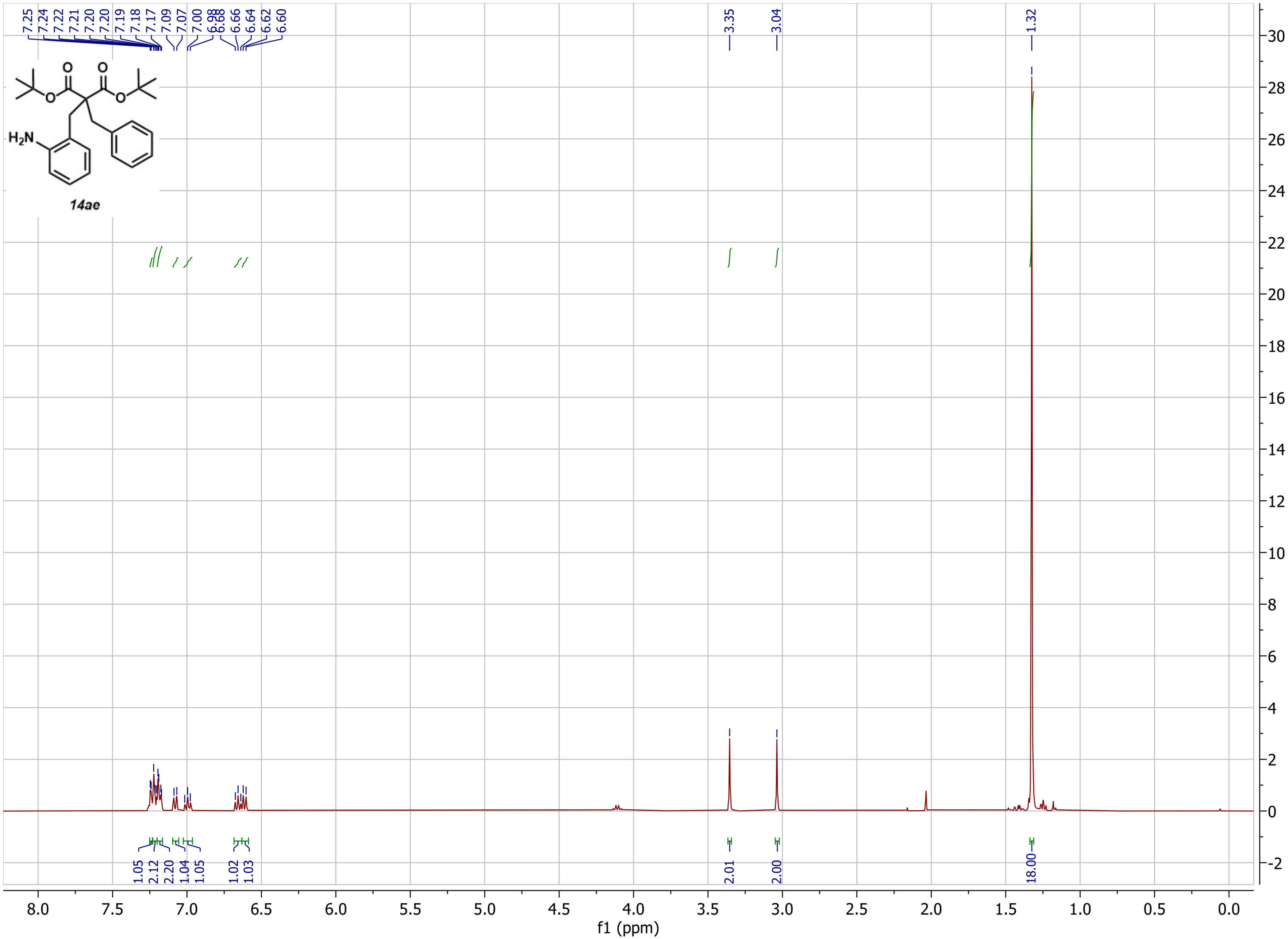

^{1}H NMR. Solvent: $CDCl_3$. B_0 = 400 MHz.

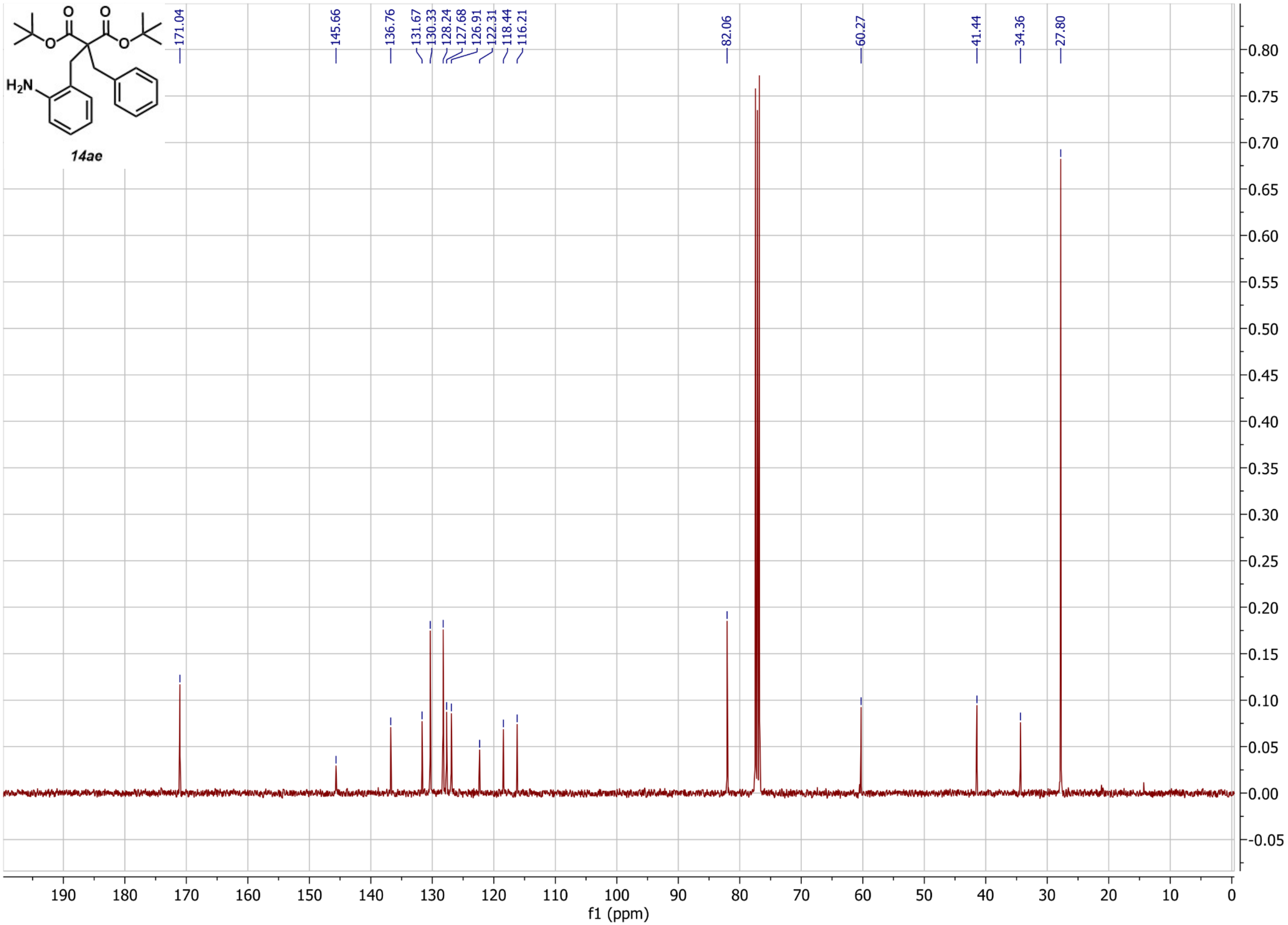

$^{13}C\{^1H\}$ NMR. Solvent: $CDCl_3$. B_0 = 100 MHz.

*Compound **14af***

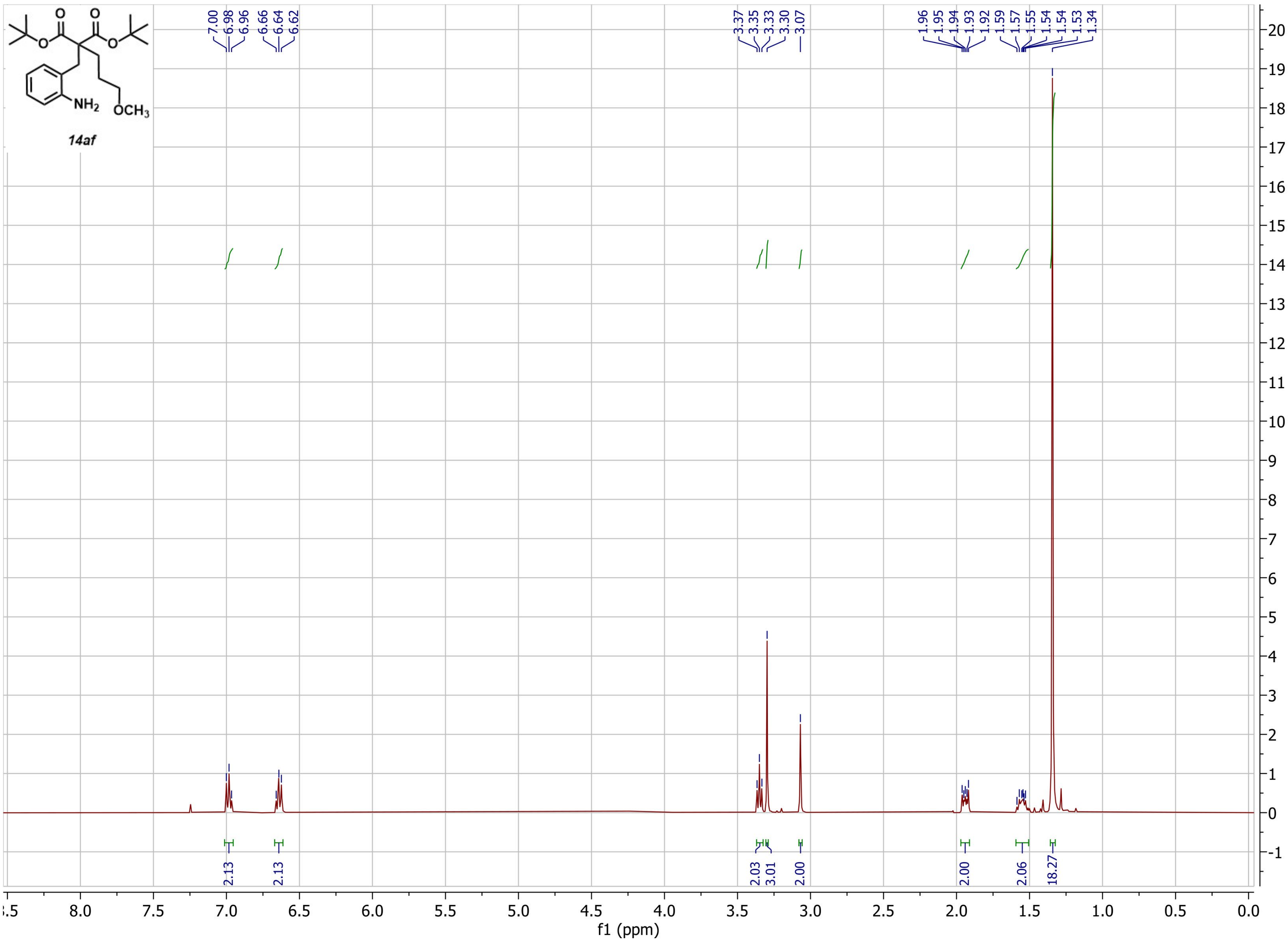

^1H NMR. Solvent: $CDCl_3$. B_0 = 400 MHz.

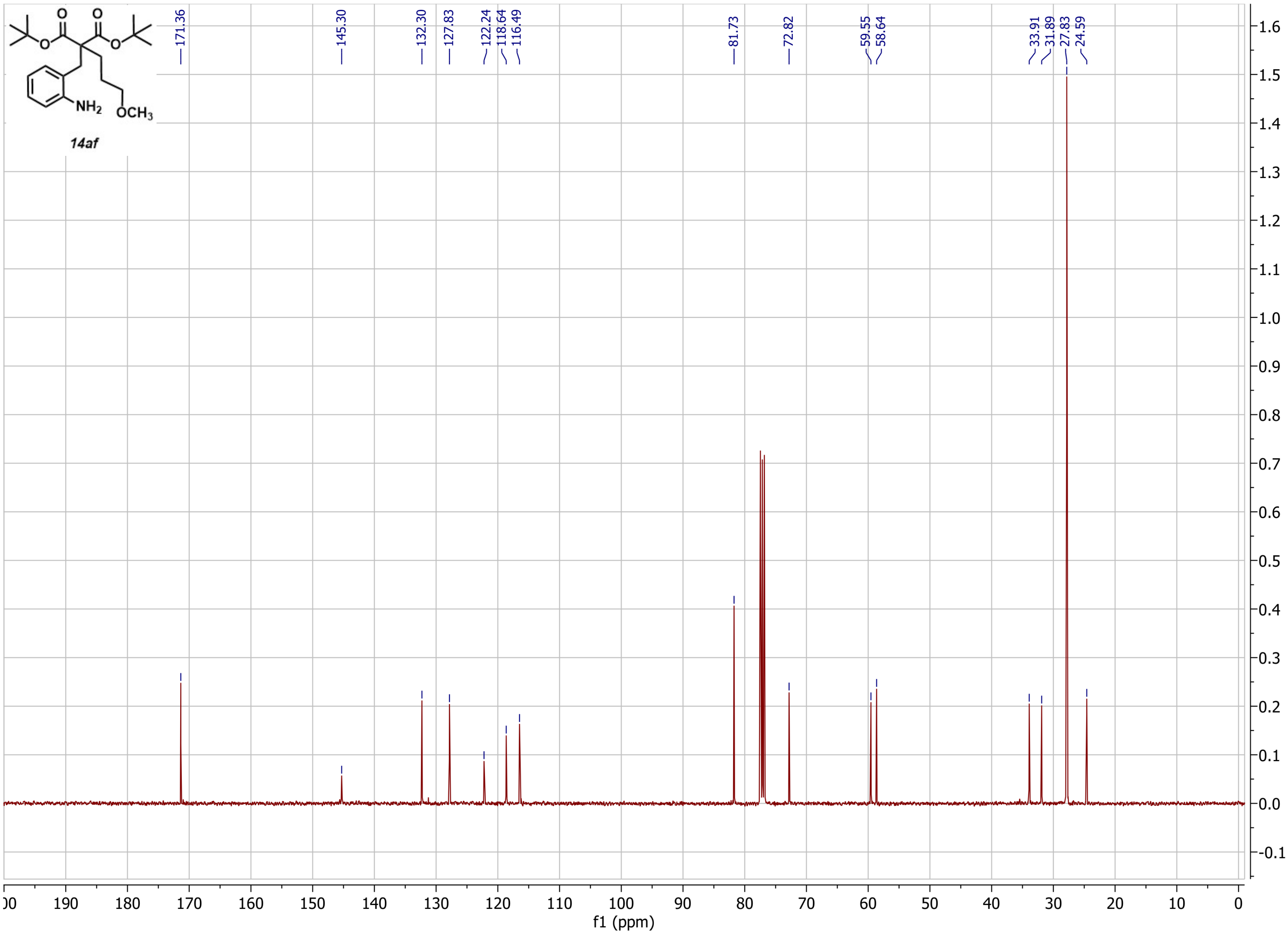

$^{13}C\{^1H\}$ NMR. Solvent: $CDCl_3$. B_0 = 100 MHz.

Compound ***14ba***

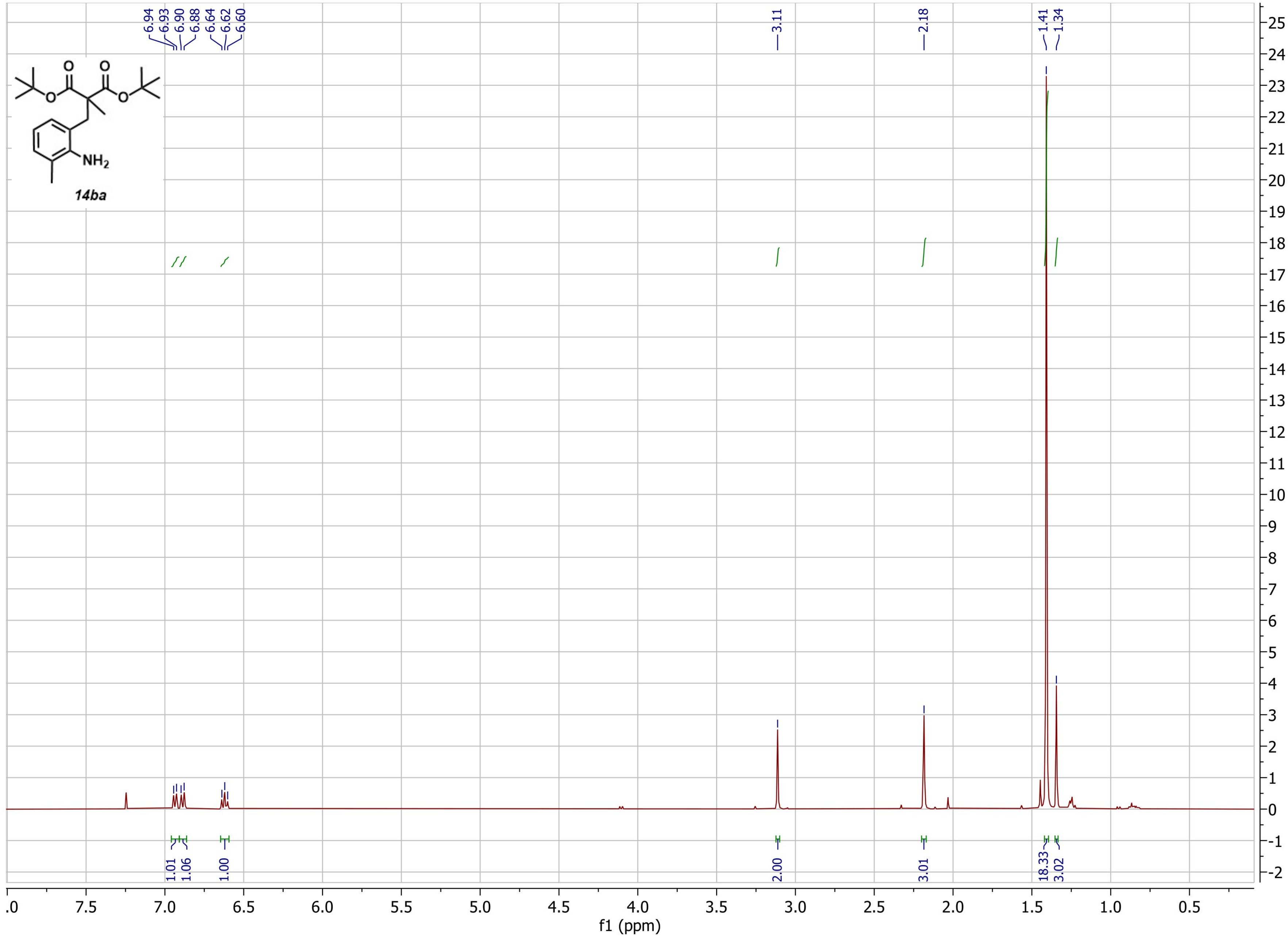

^{1}H NMR. Solvent: $CDCl_3$. B_0 = 400 MHz.

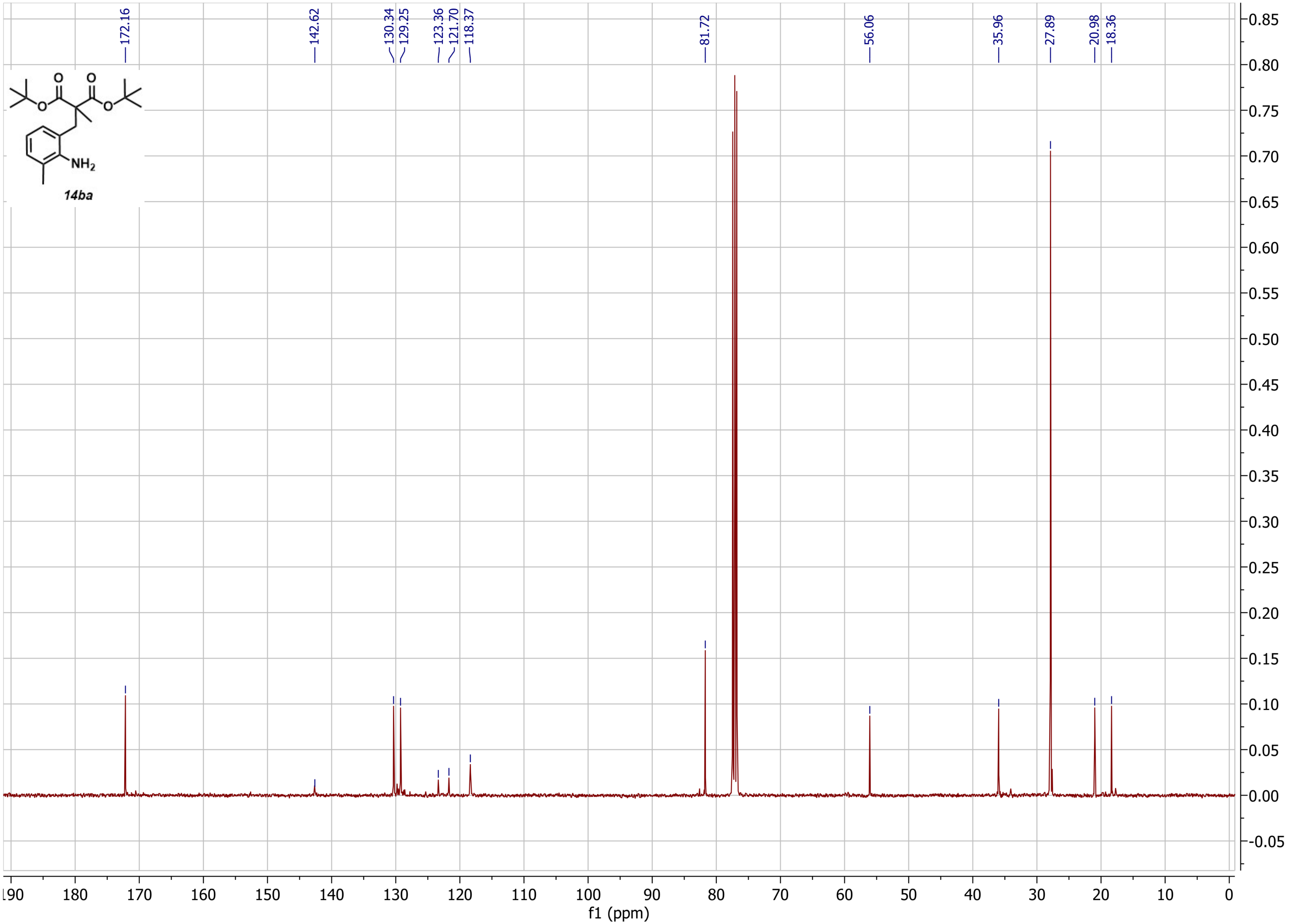

$^{13}C\{^1H\}$ NMR. Solvent: $CDCl_3$. B_0 = 100 MHz.

*Compound **14cb***

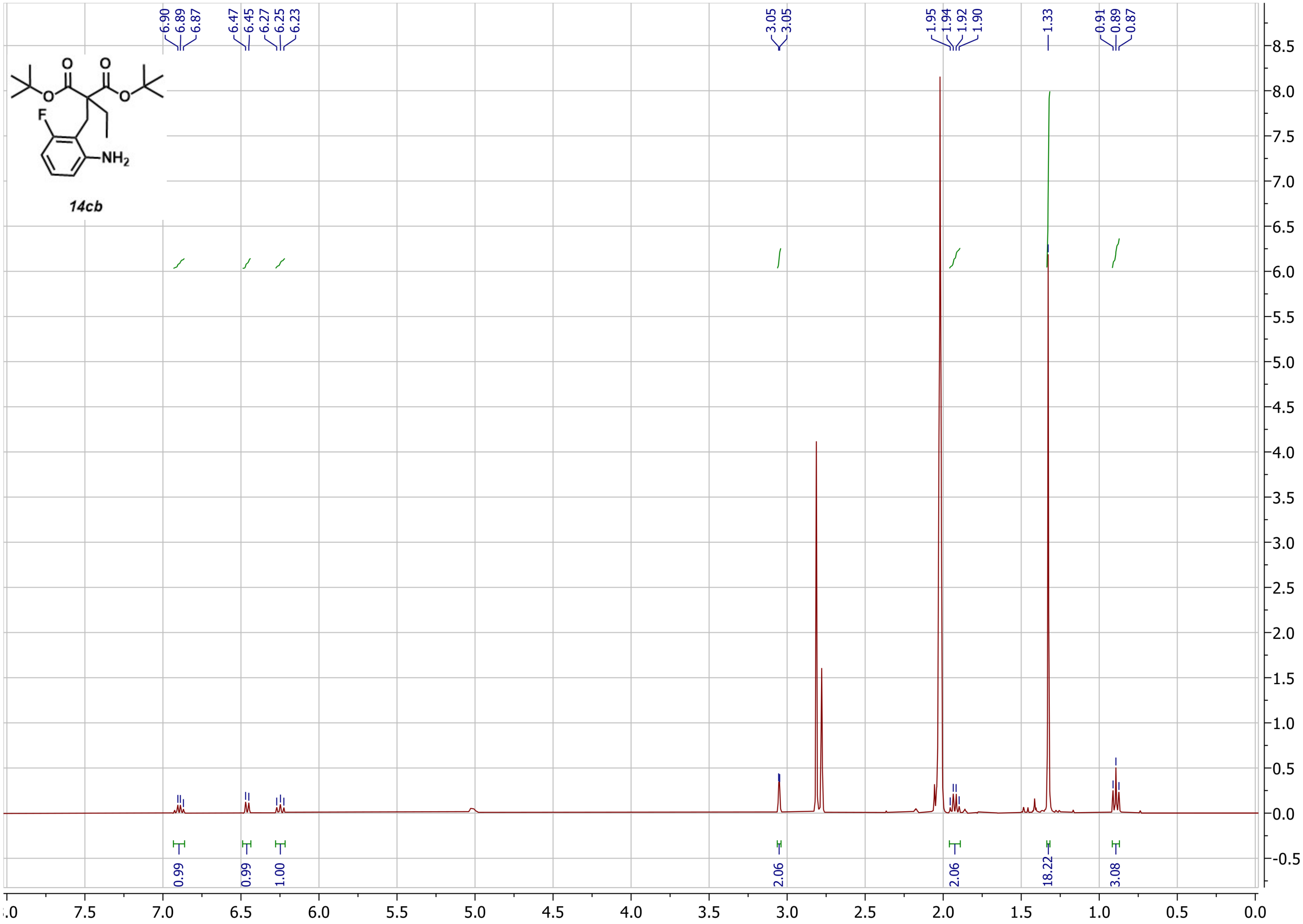

^{1}H NMR. Solvent: Acetone-d$_6$. B$_0$ = 400 MHz.

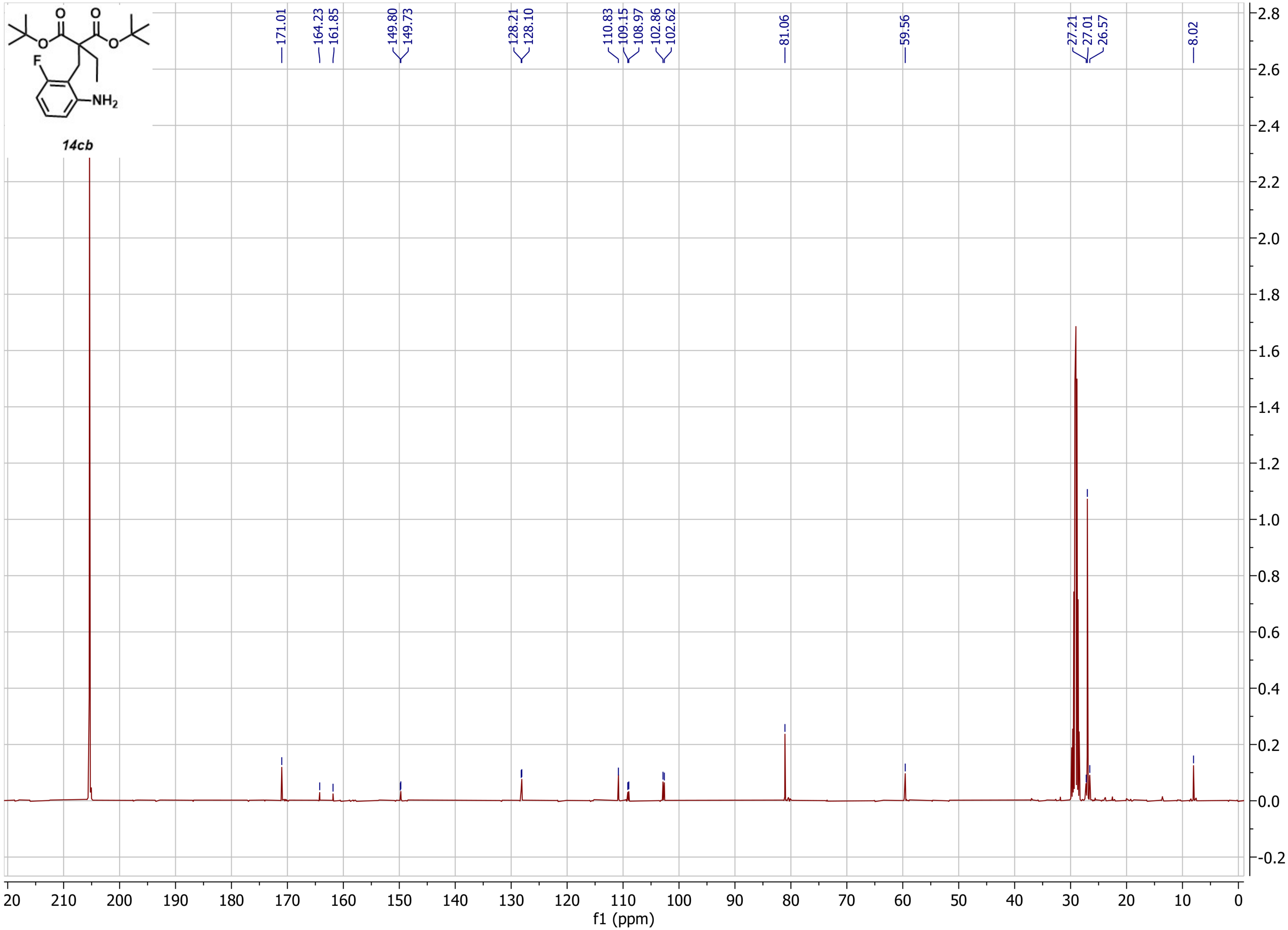

$^{13}C\{^1H\}$ NMR. Solvent: Acetone-d_6. B_0 =100 MHz.

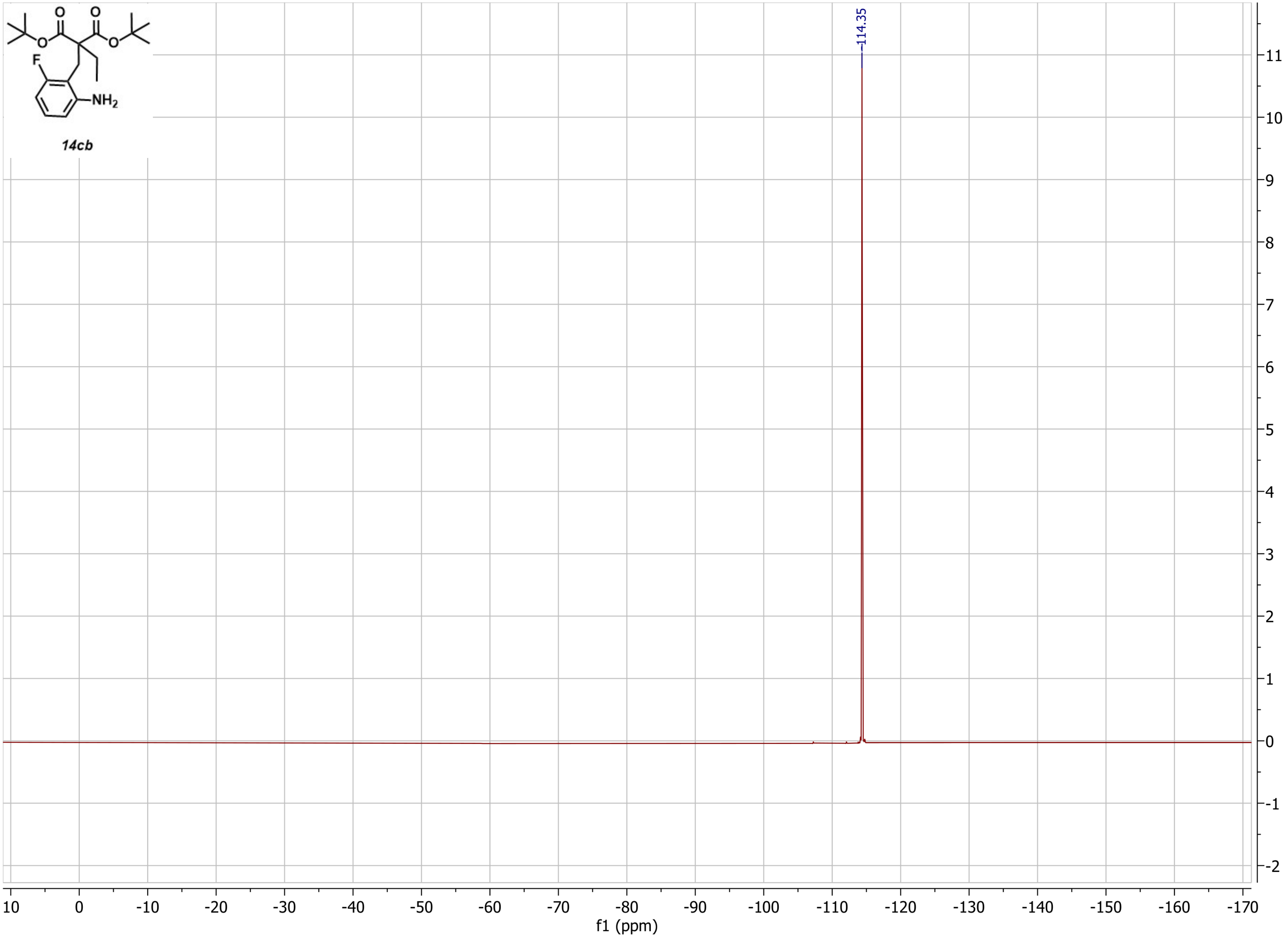

^{19}F NMR. Solvent: Acetone-d_6. B_0 = 376 MHz.

*Compound **14da***

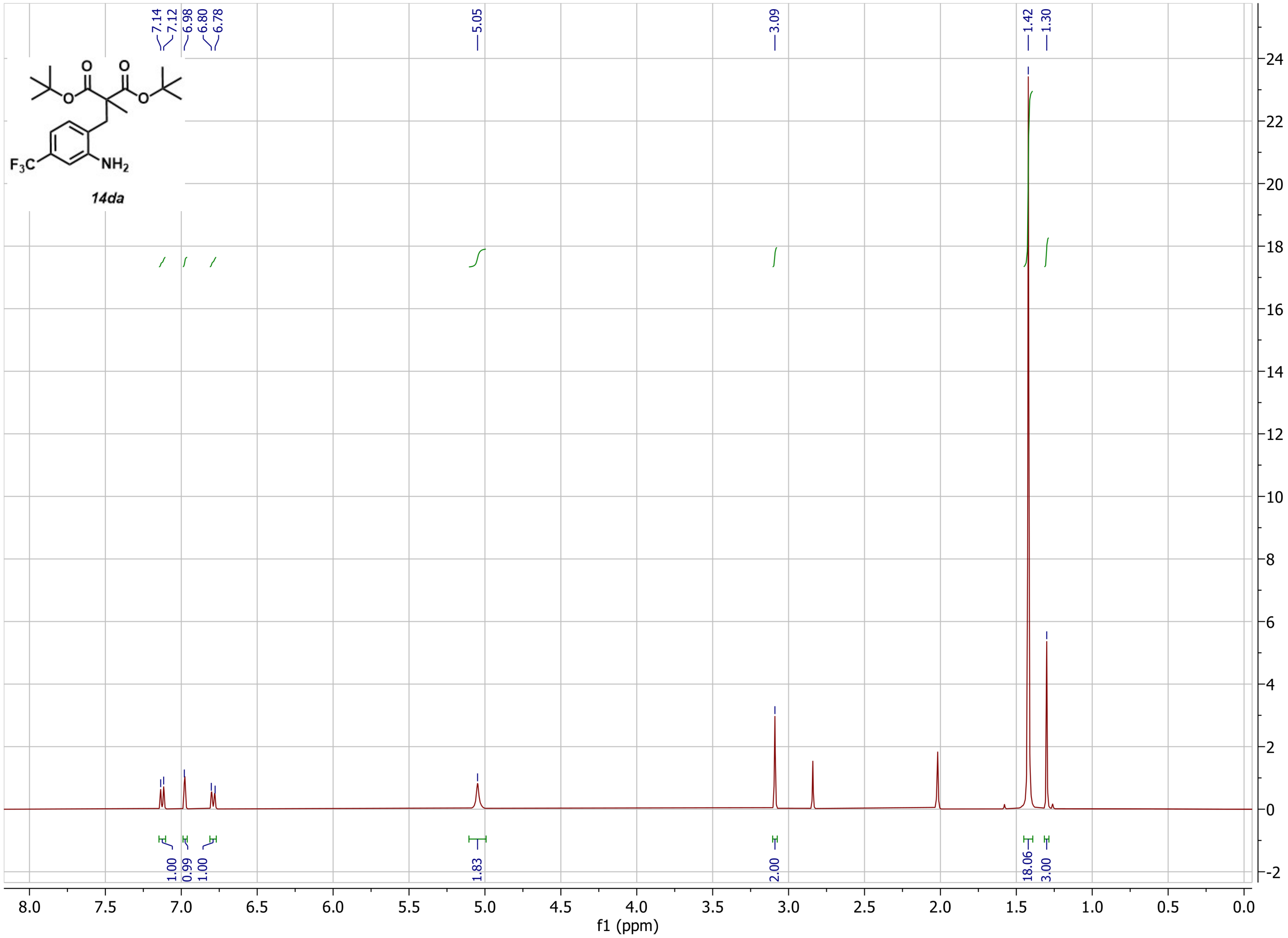

^1H NMR. Solvent: Acetone-d_6. B_0 = 400 MHz.

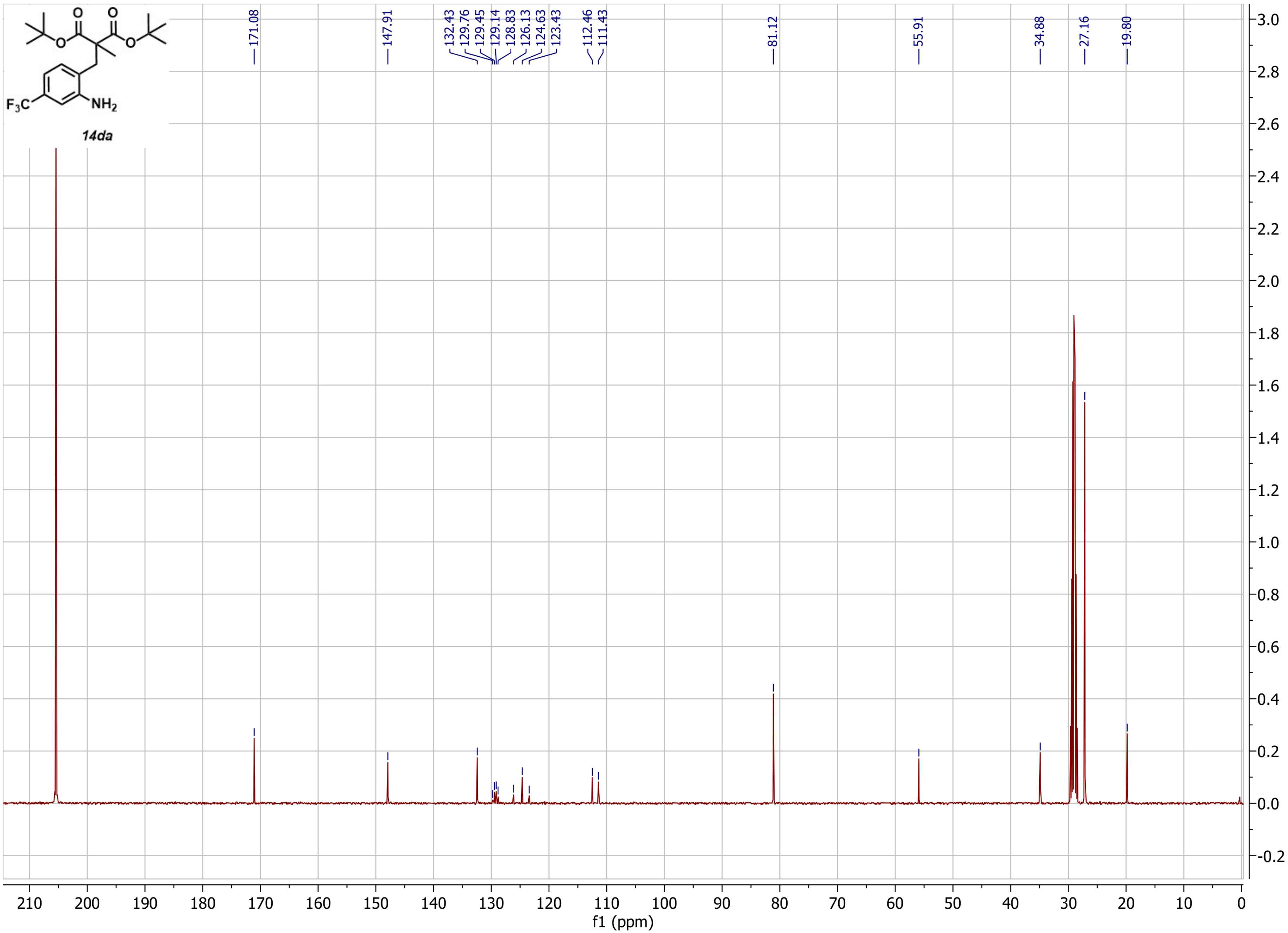

$^{13}C\{1H\}$ NMR. Solvent: Acetone-d_6. B_0 =100 MHz.

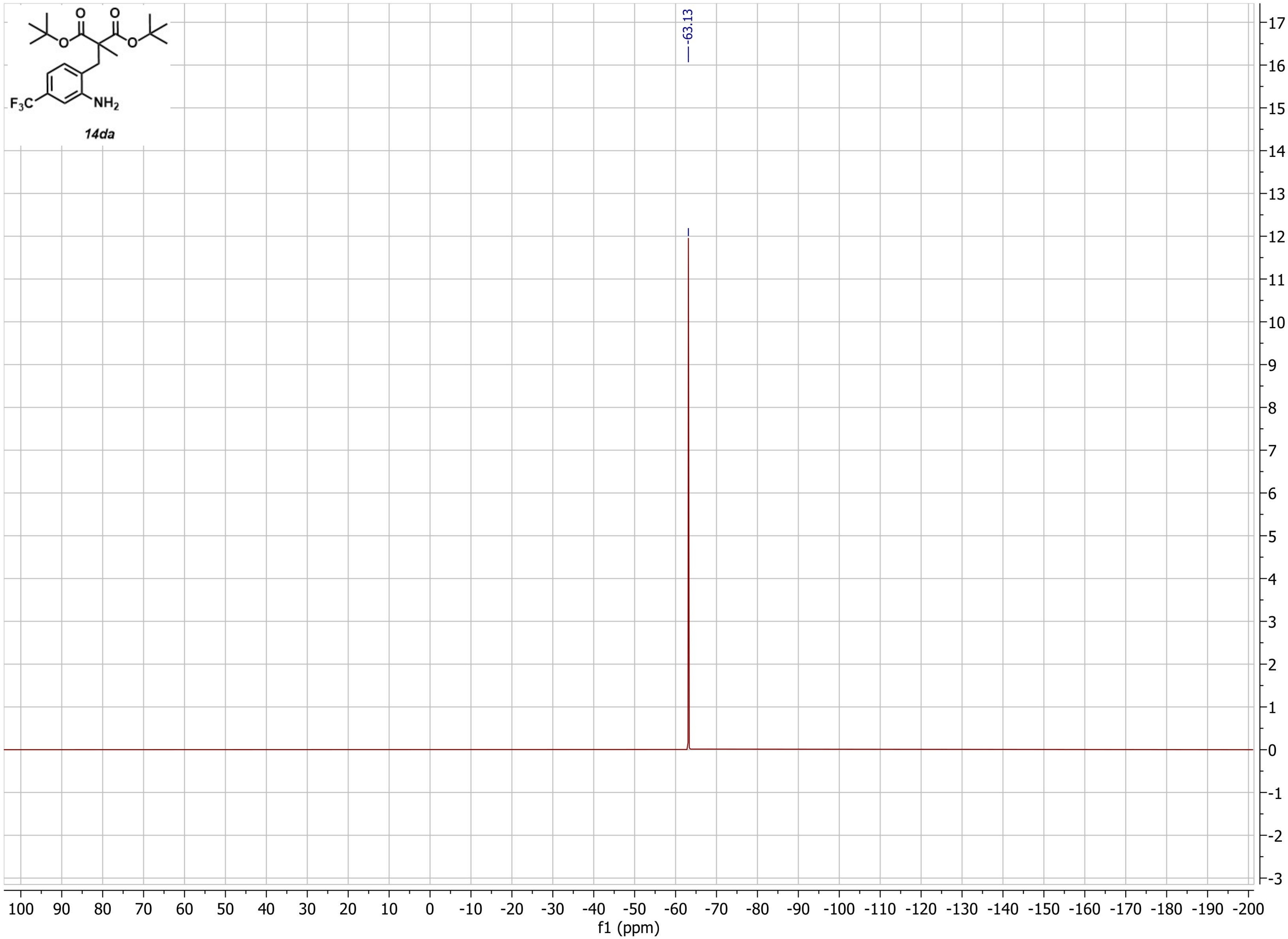

^{19}F NMR. Solvent: Acetone-d_6. B_0 = 376 MHz.

*Compound **14ea***

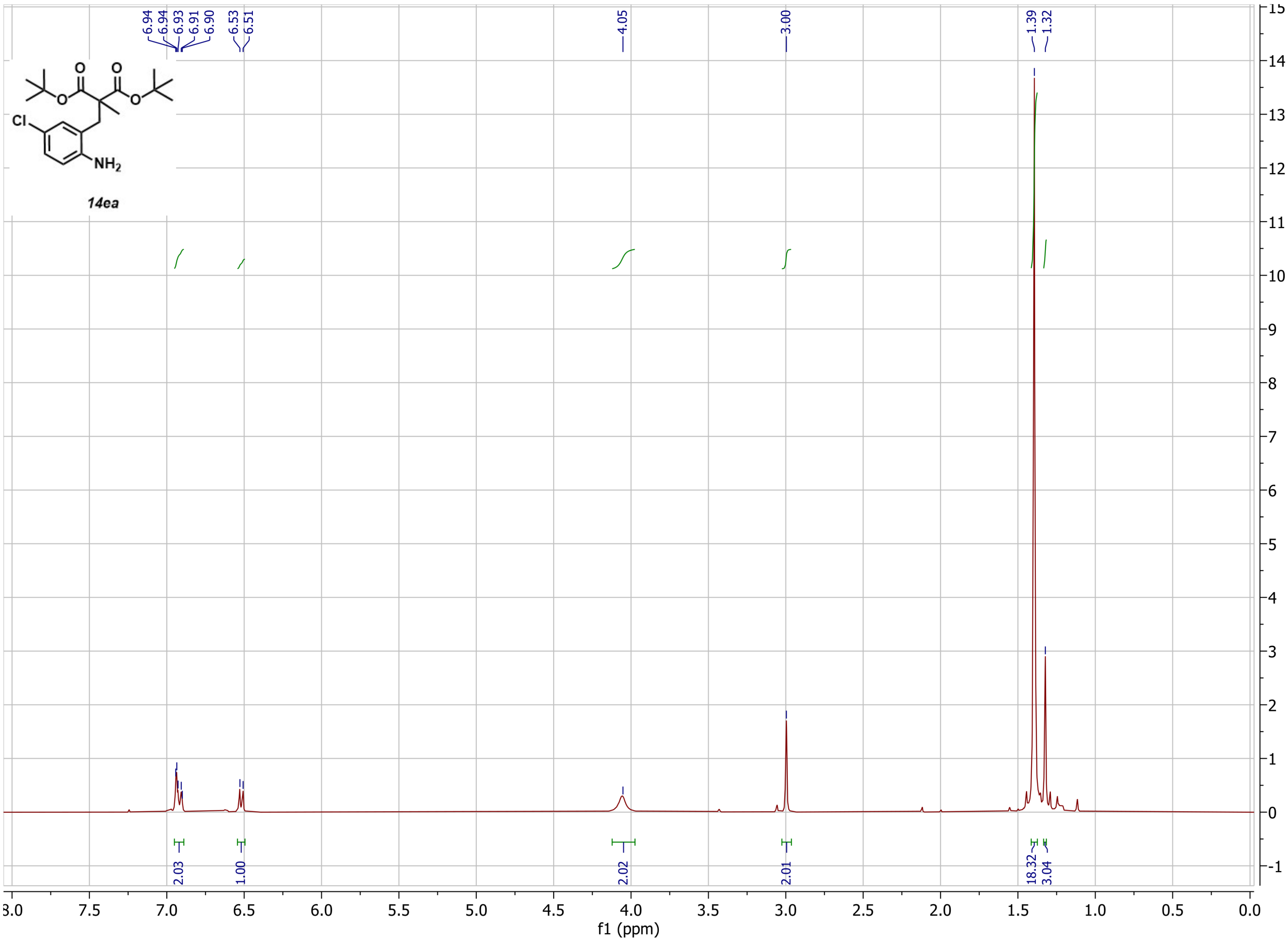

^{1}H NMR. Solvent: $CDCl_3$. B_0 = 400 MHz.

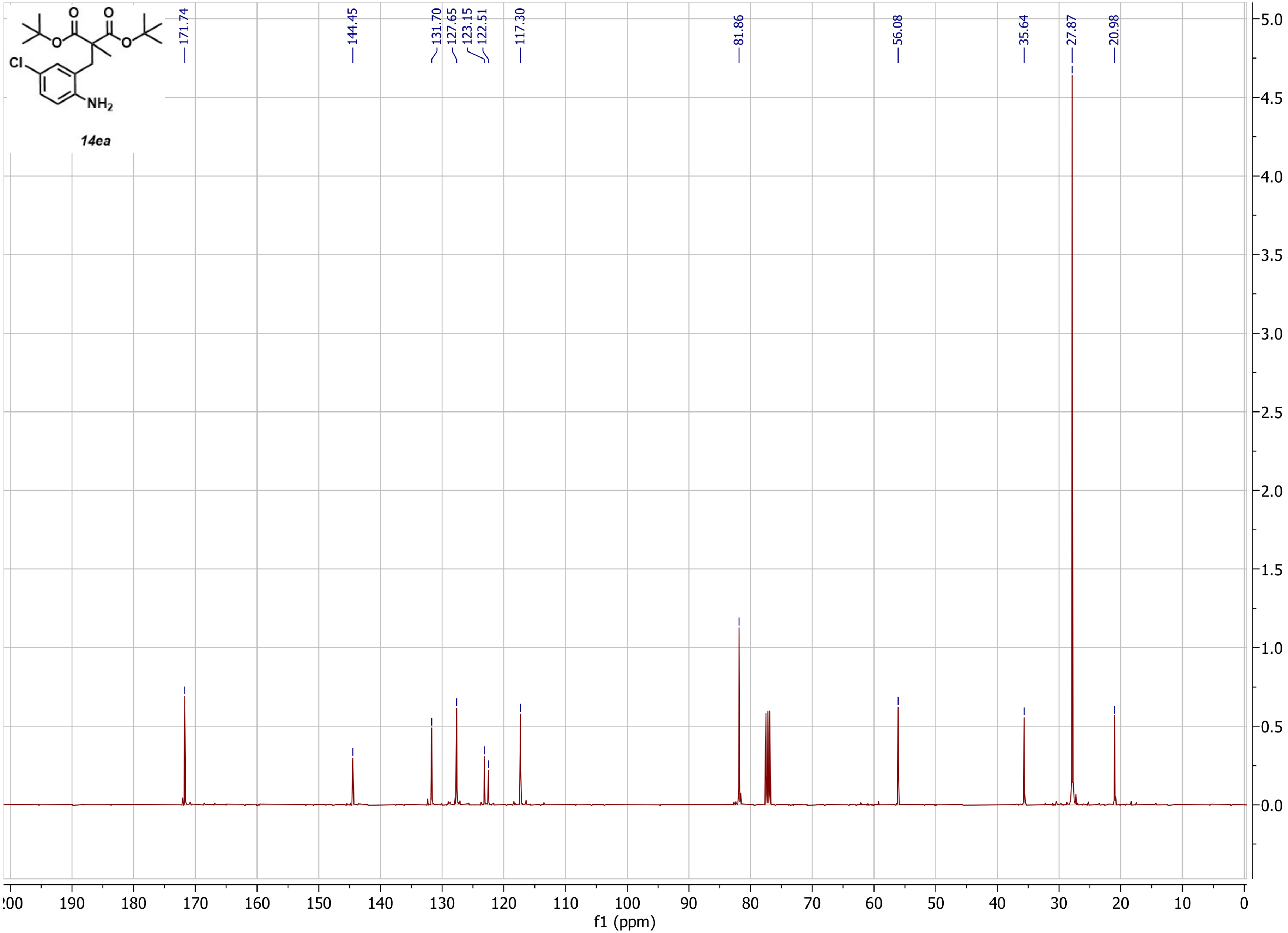

$^{13}C\{^1H\}$ NMR. Solvent: $CDCl_3$. B_0 = 100 MHz.

Compound ***14eb***

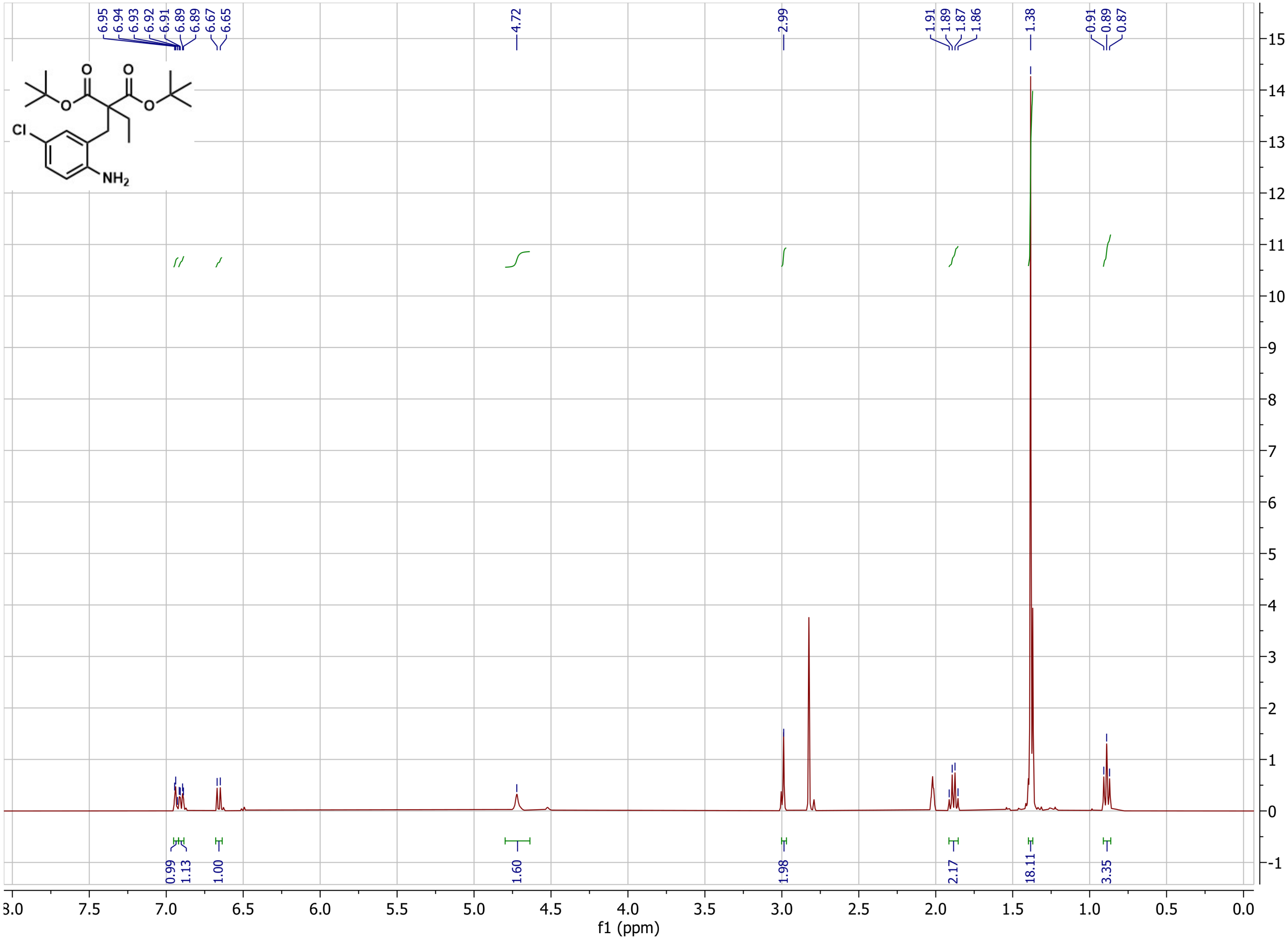

^{1}H NMR. Solvent: Acetone-d$_6$. B$_0$ = 400 MHz.

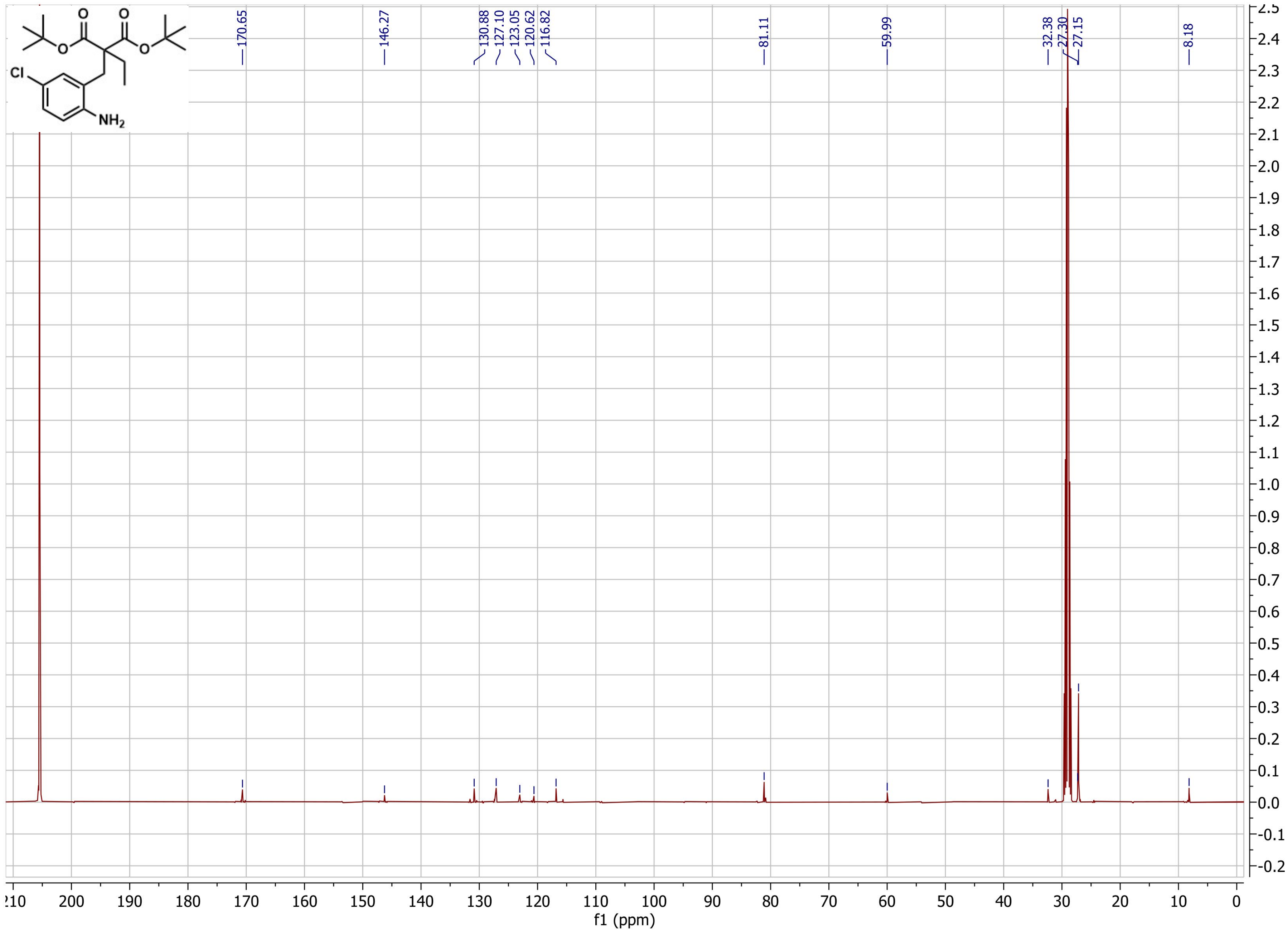

$^{13}C\{^1H\}$ NMR. Solvent: Acetone-d_6. B_0 =100 MHz.

*Compound **14fa***

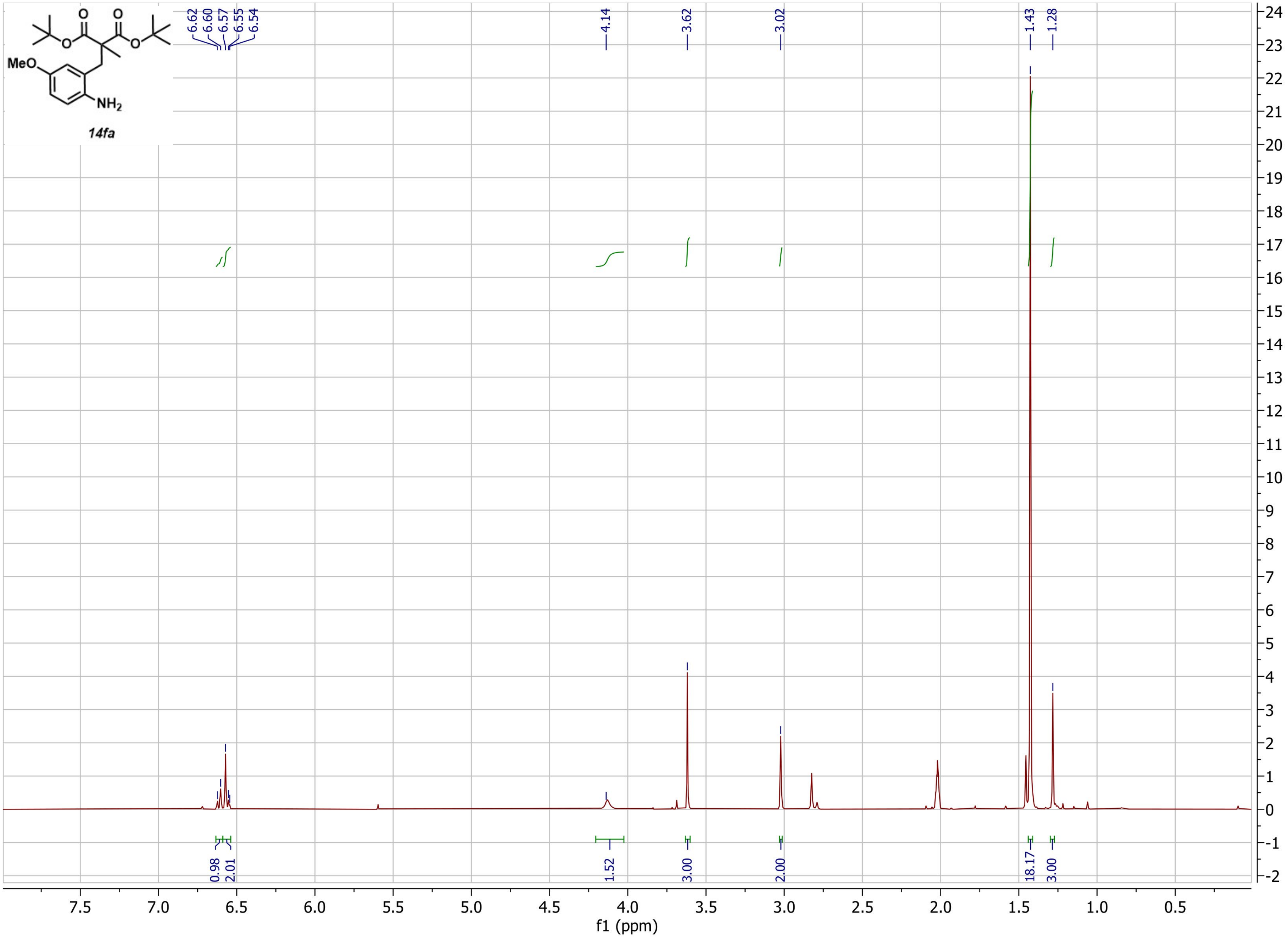

^{1}H NMR. Solvent: Acetone-d$_6$. B$_0$ = 400 MHz.

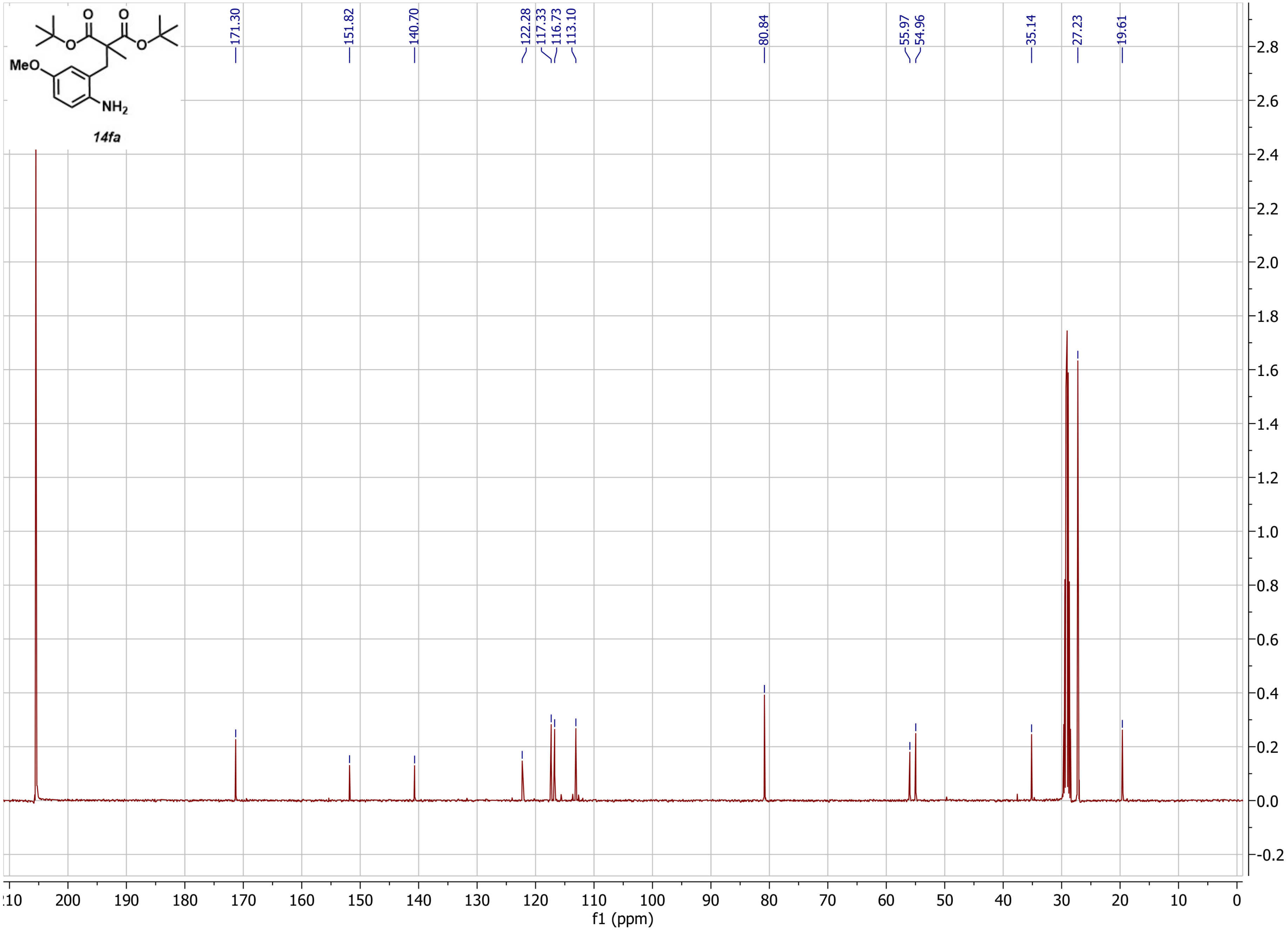

$^{13}C\{^1H\}$ NMR. Solvent: Acetone-d_6. B_0 =100 MHz.

Compound ***24aa***

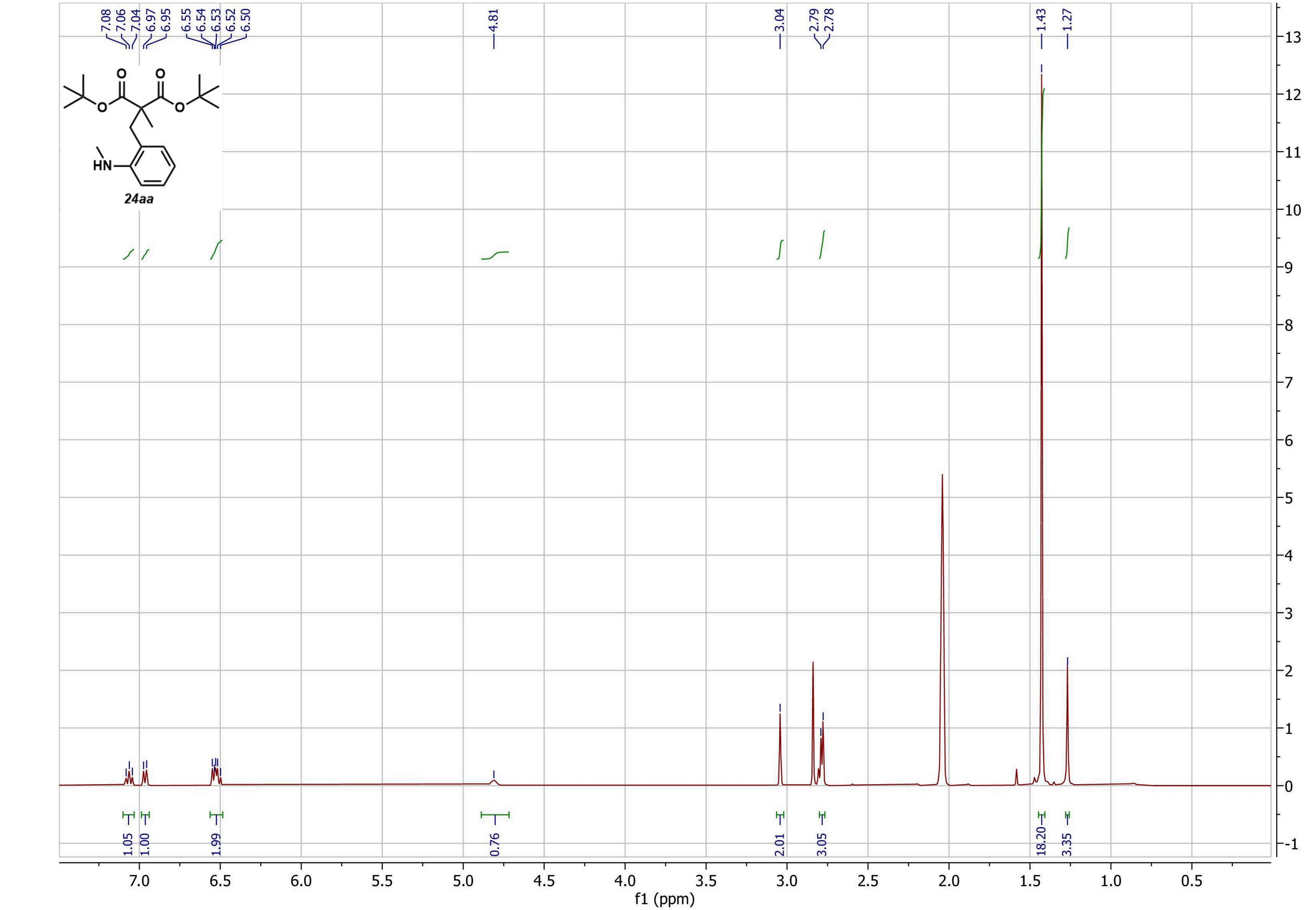

^{1}H NMR. Solvent: Acetone-d$_6$. B$_0$ = 400 MHz.

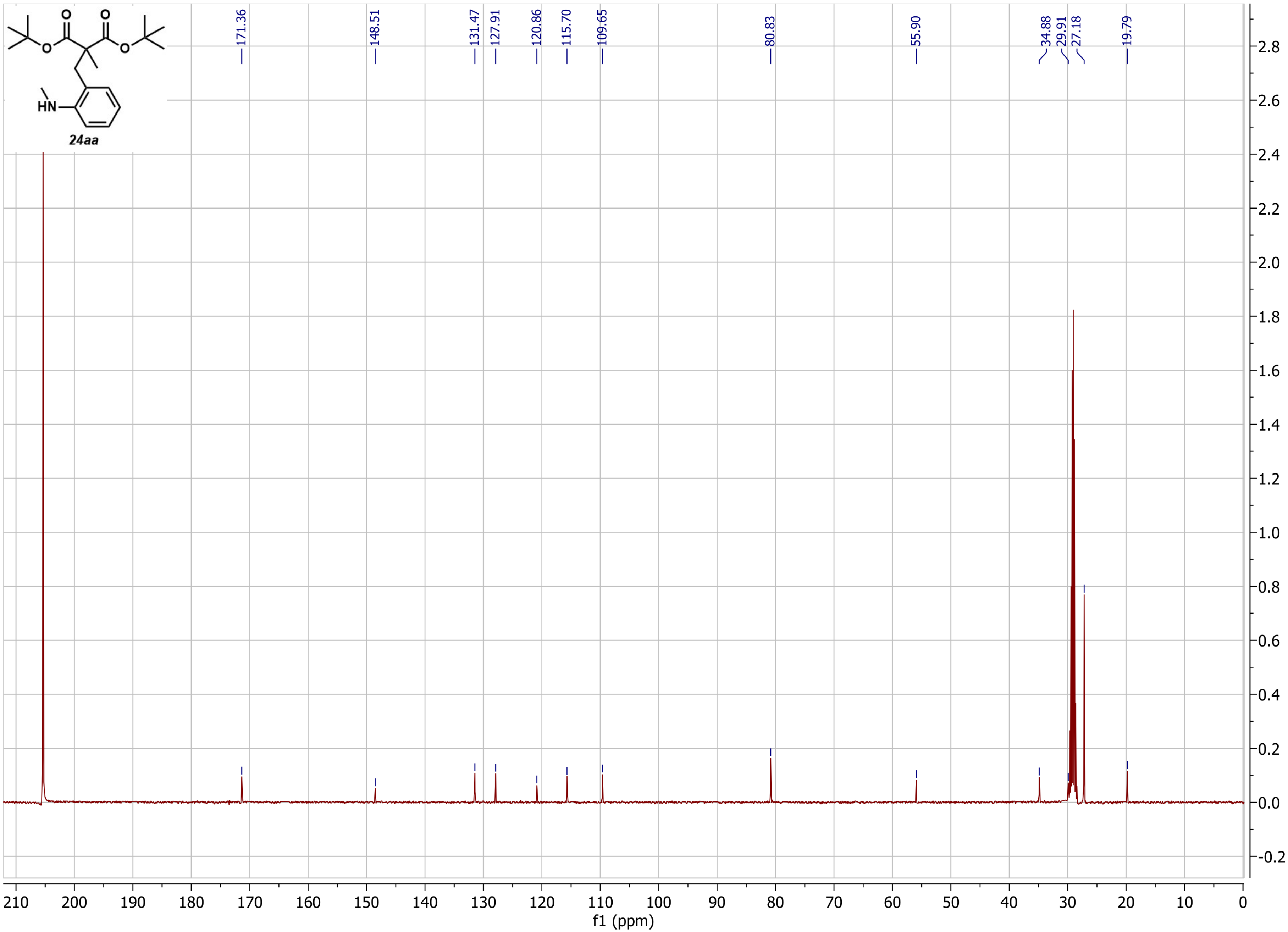

$^{13}C\{^1H\}$ NMR. Solvent: Acetone-d_6. B_0 =100 MHz.

*Compound **33aa***

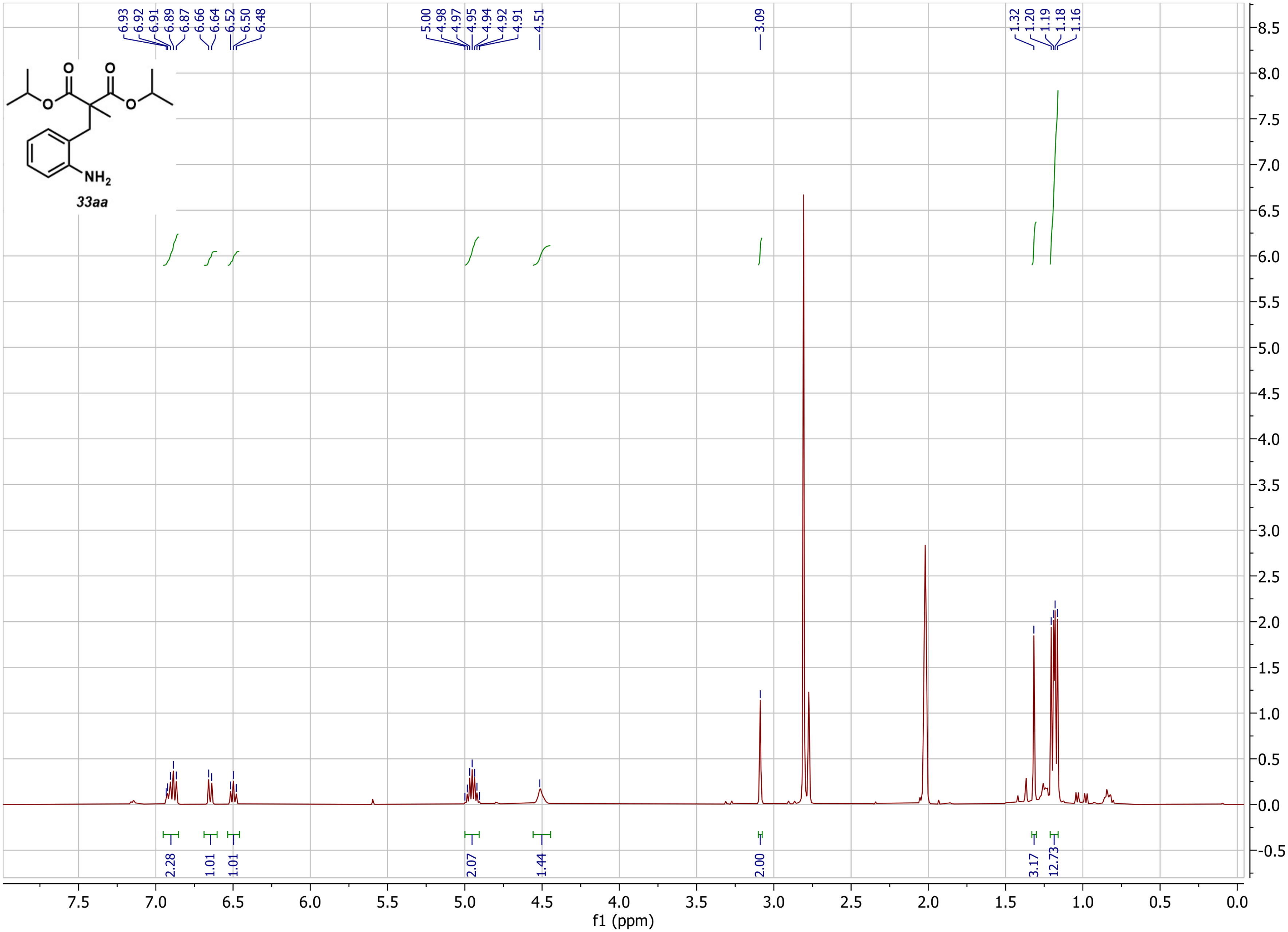

^{1}H NMR. Solvent: Acetone-d_6. B_0 = 400 MHz.

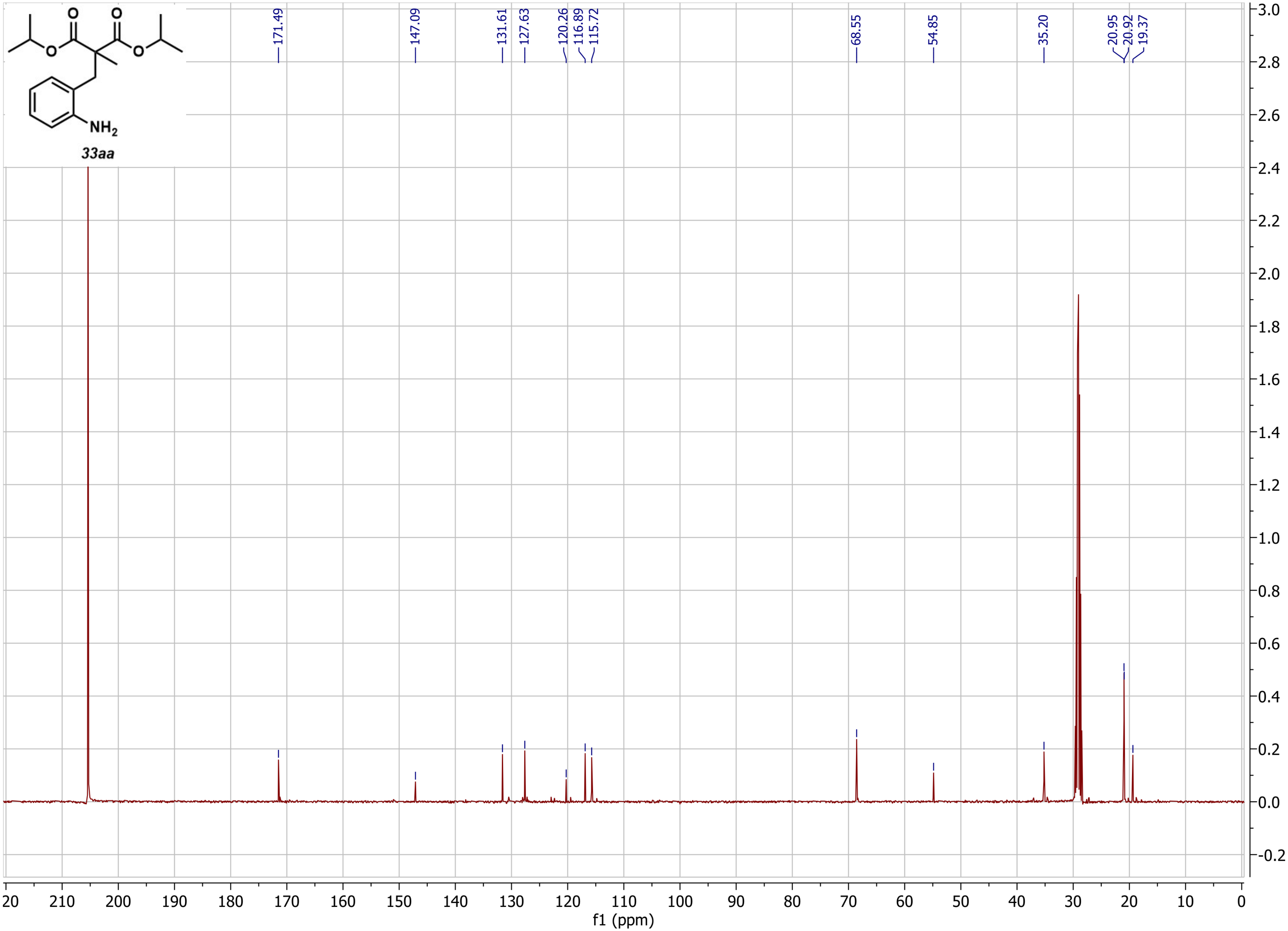

$^{13}C\{^1H\}$ NMR. Solvent: Acetone-d_6. B_0 = 100 MHz.

Compound ***15aa***

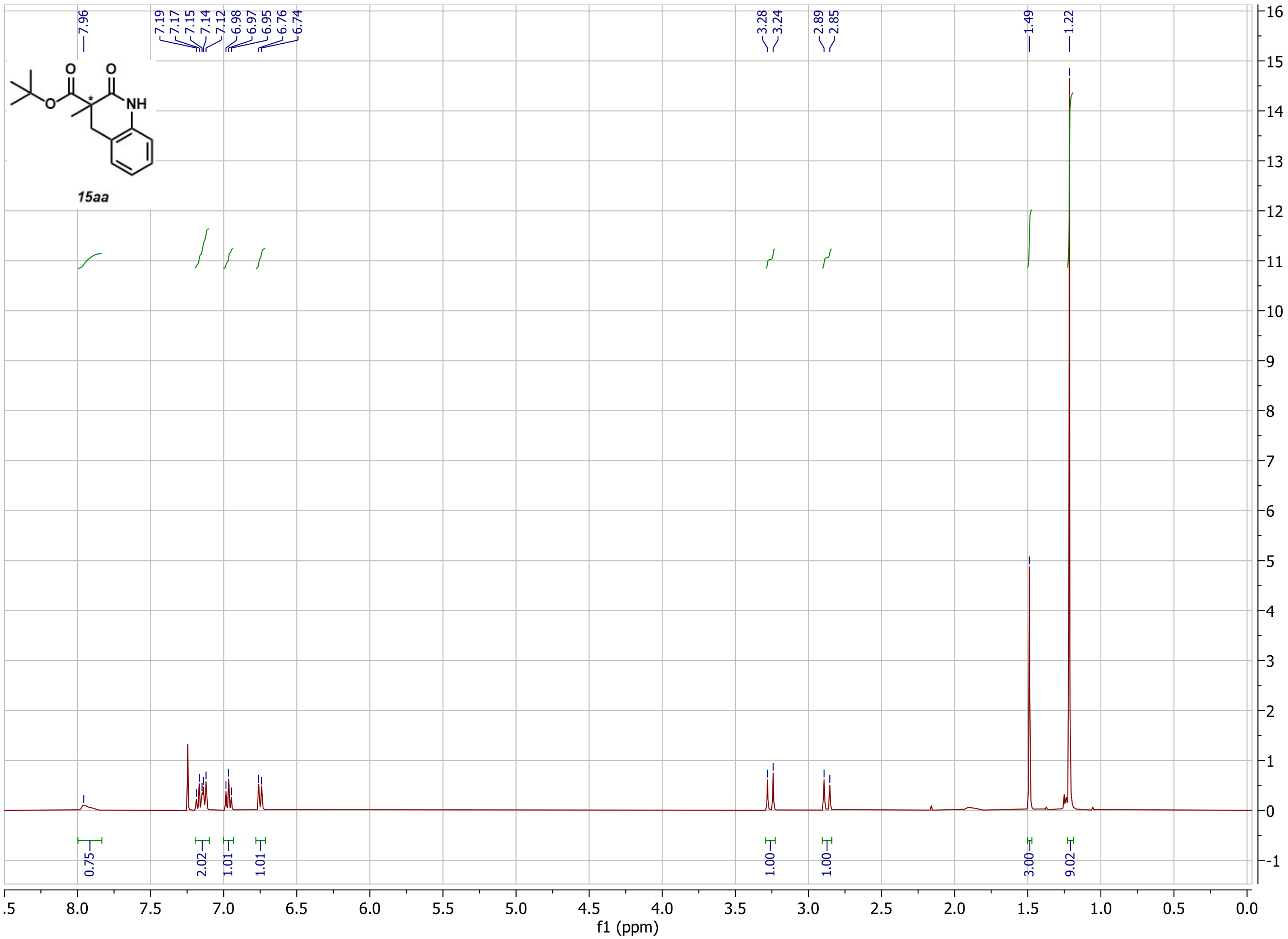

^{1}H NMR. Solvent: $CDCl_3$. B_0 = 400 MHz.

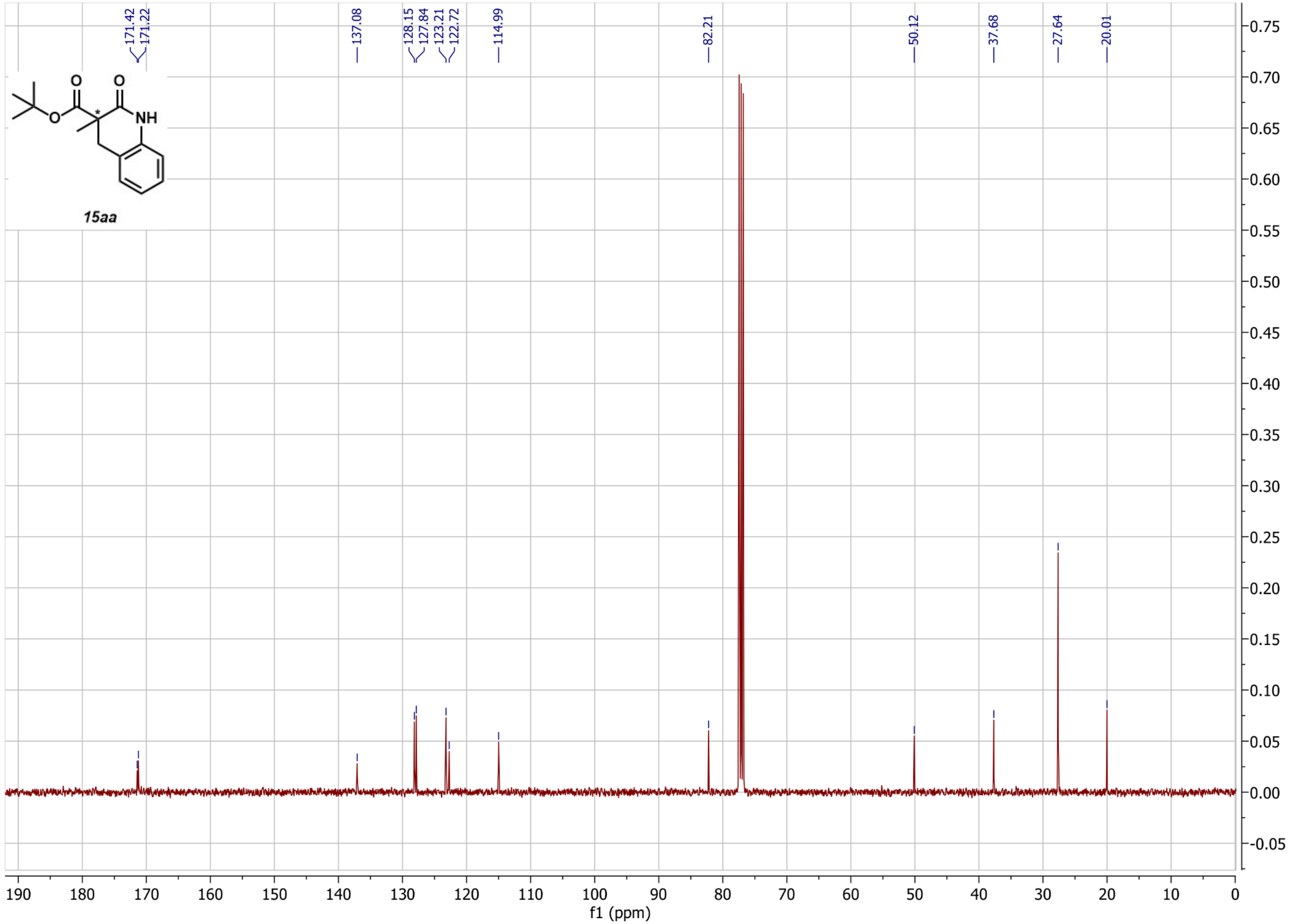

^{13}C{1H} NMR. Solvent: $CDCl_3$. B_0 = 100 MHz.

Compound ***15ab***

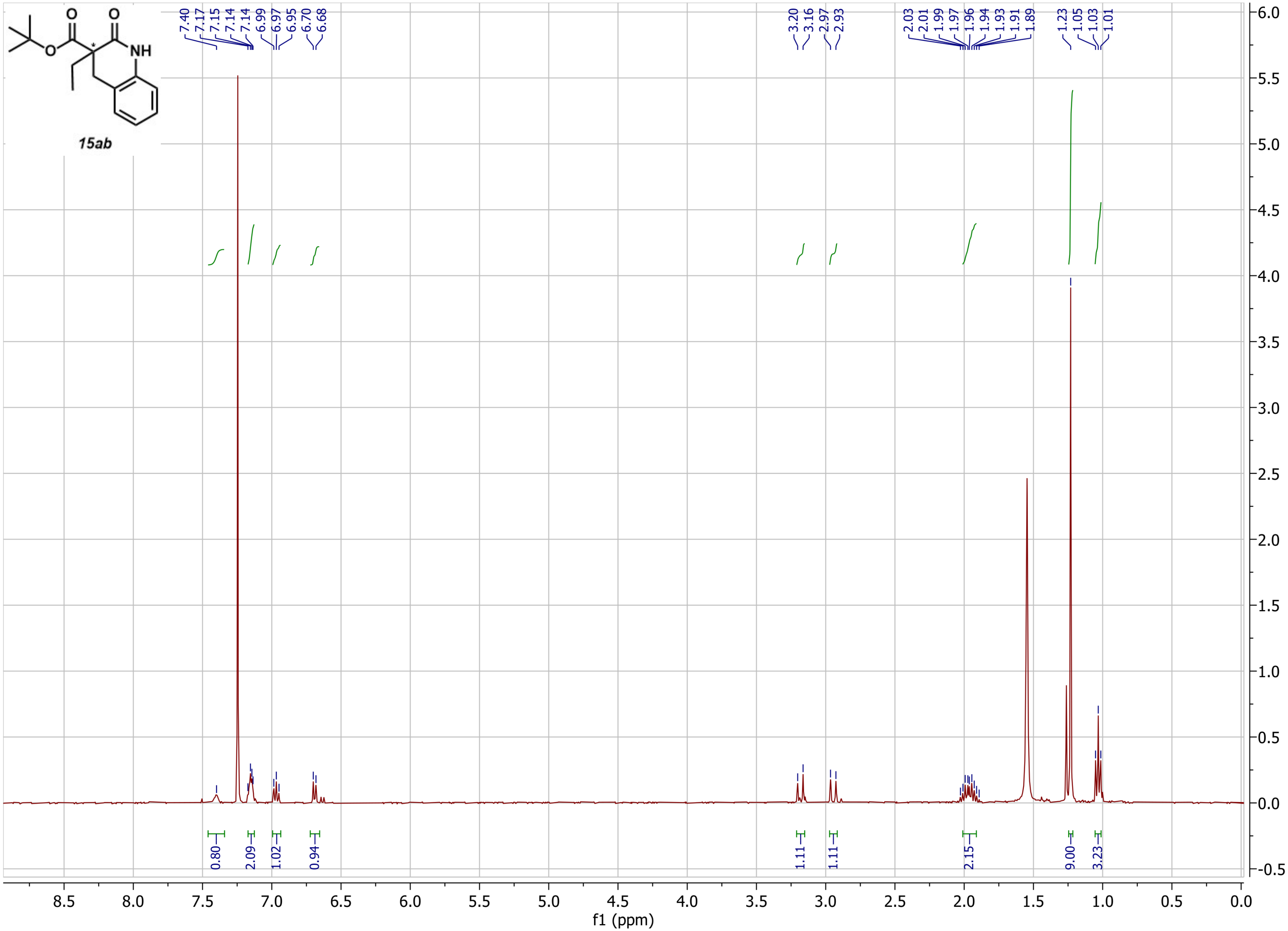

^{1}H NMR. Solvent: $CDCl_3$. B_0 = 400 MHz.

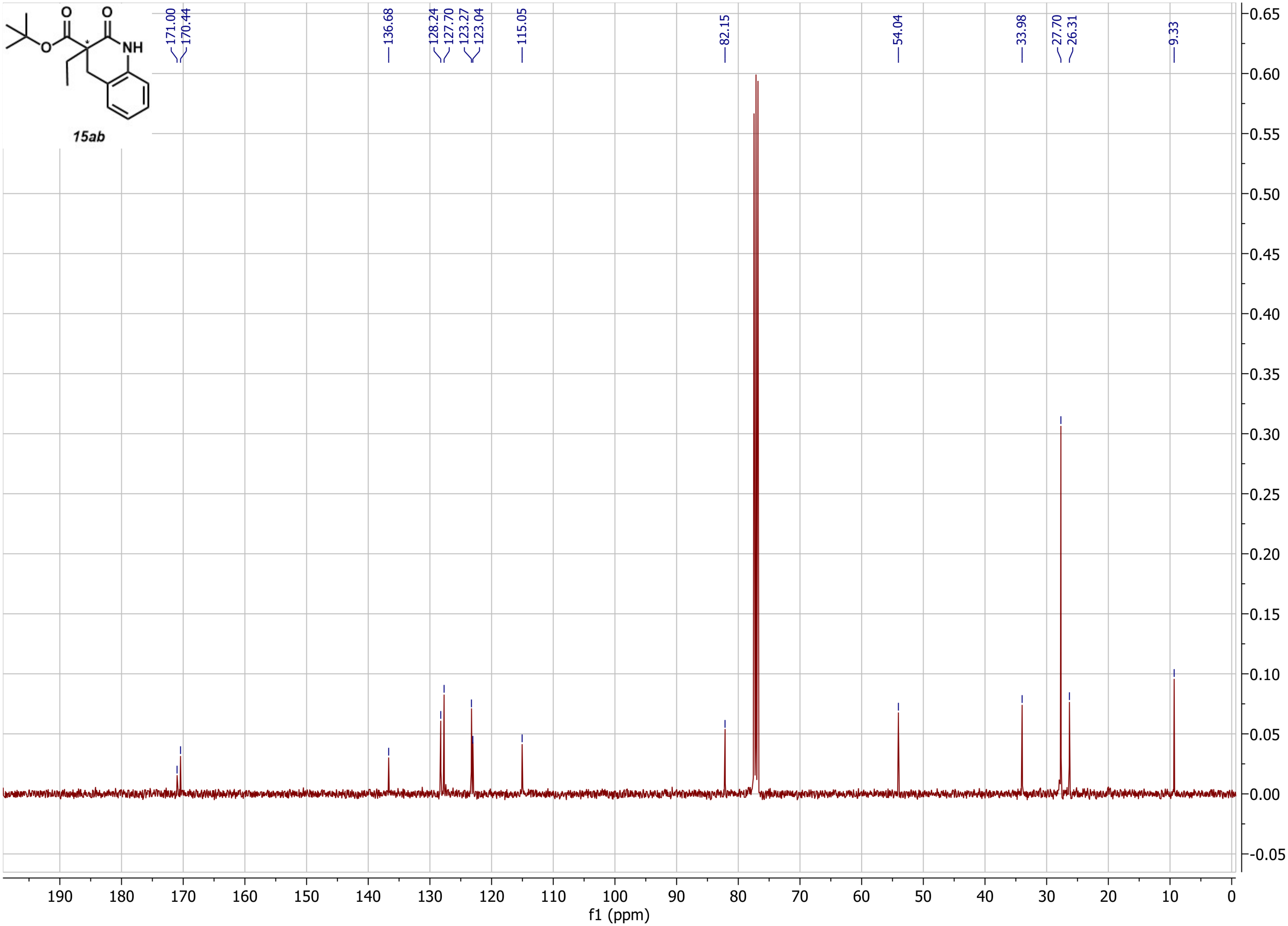

$^{13}C\{^1H\}$ NMR. Solvent: $CDCl_3$. B_0 = 100 MHz.

Compound ***15ac***

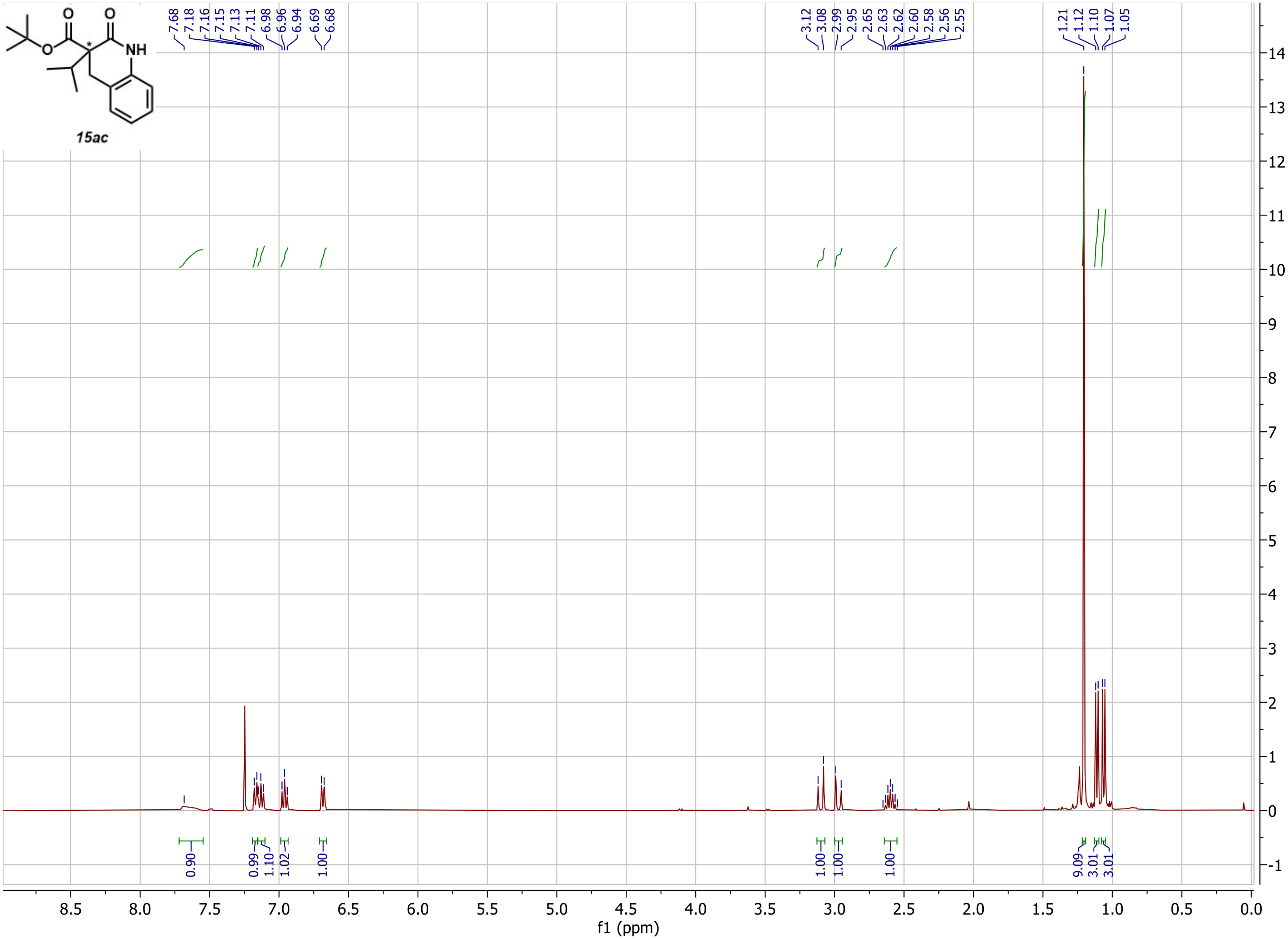

^{1}H NMR. Solvent: $CDCl_3$. B_0 = 400 MHz.

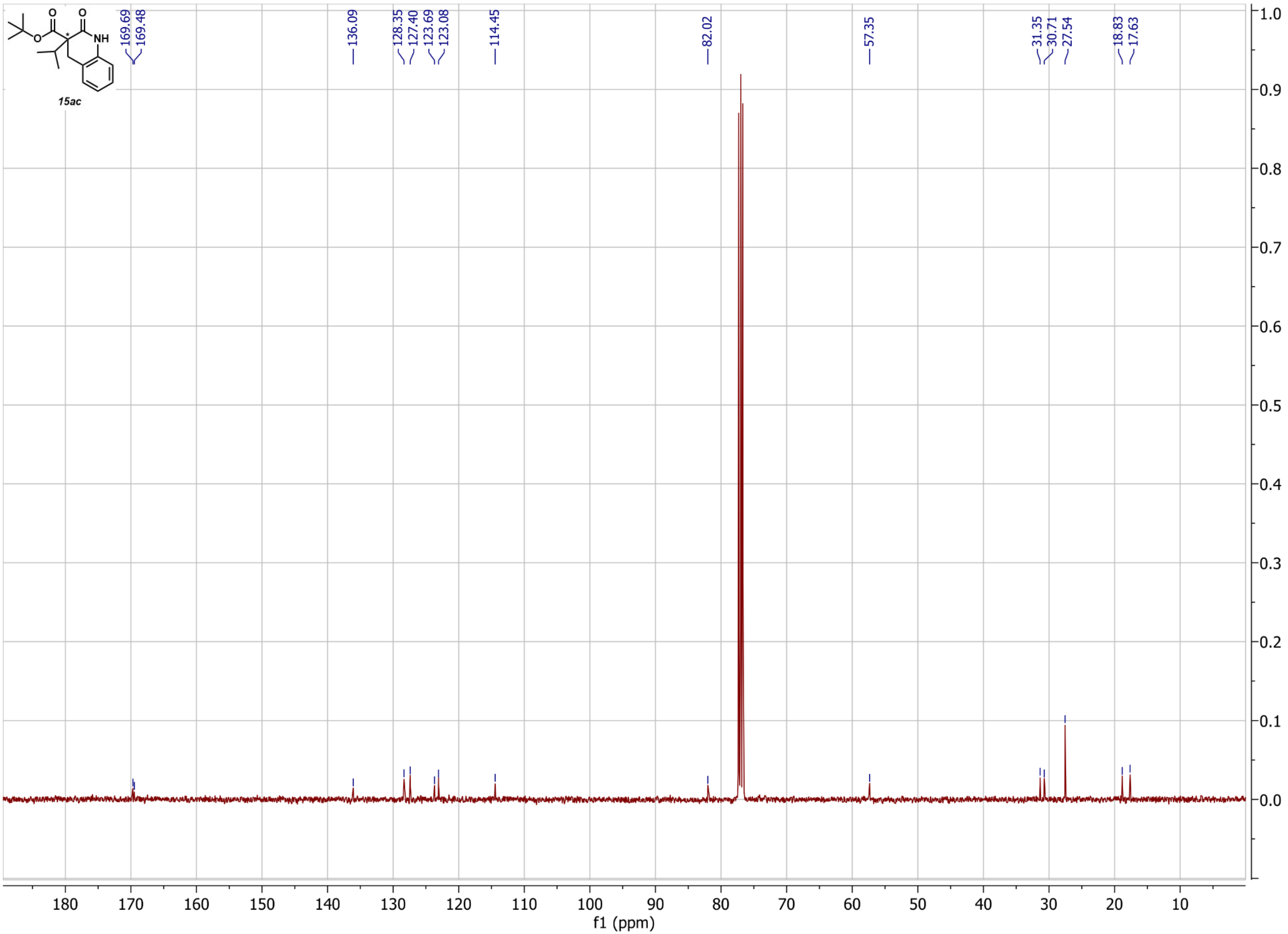

$^{13}C\{1H\}$ NMR. Solvent: $CDCl_3$. B_0 = 100 MHz.

Compound ***15ad***

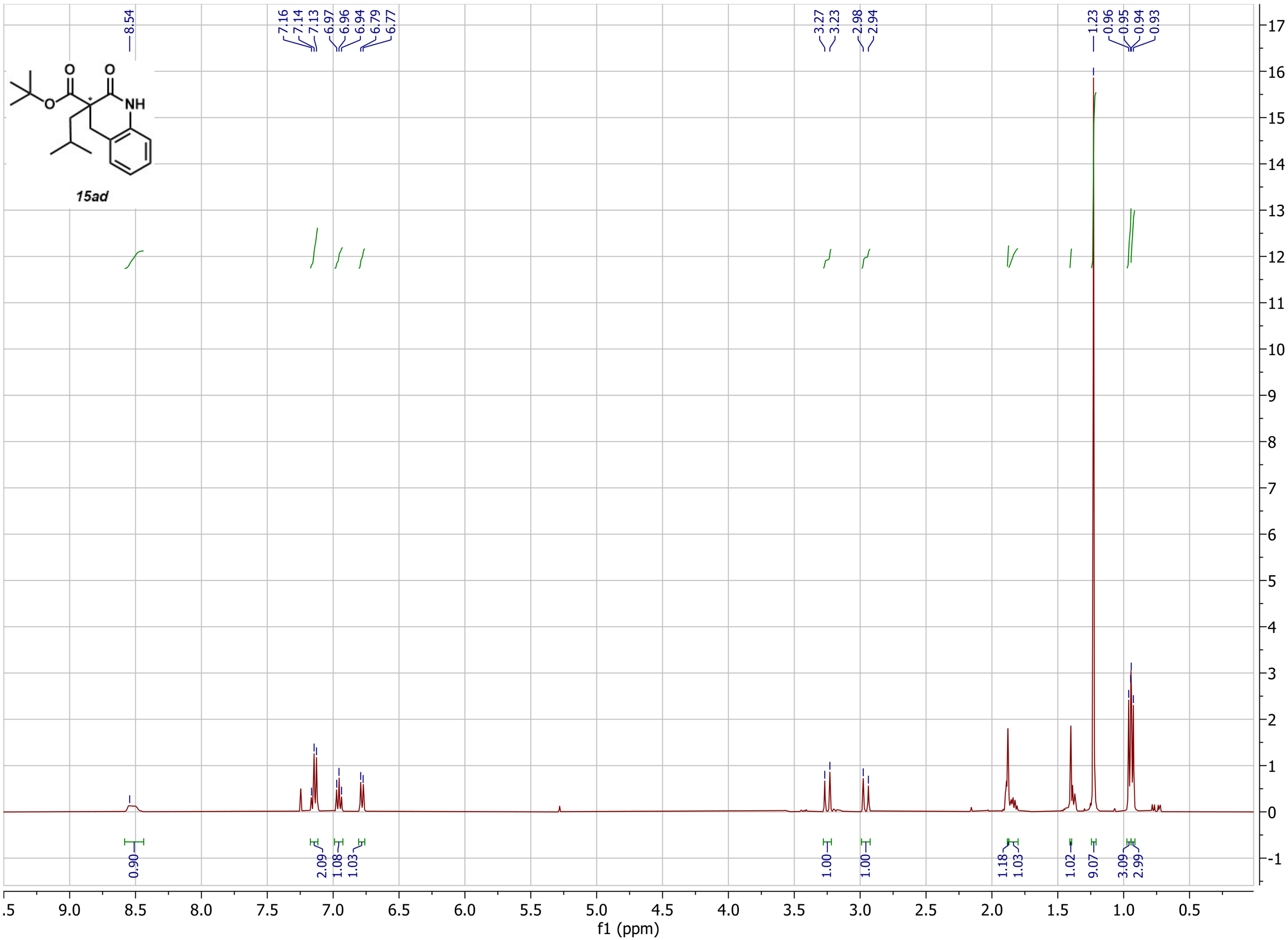

^{1}H NMR. Solvent: $CDCl_3$. B_0 = 400 MHz.

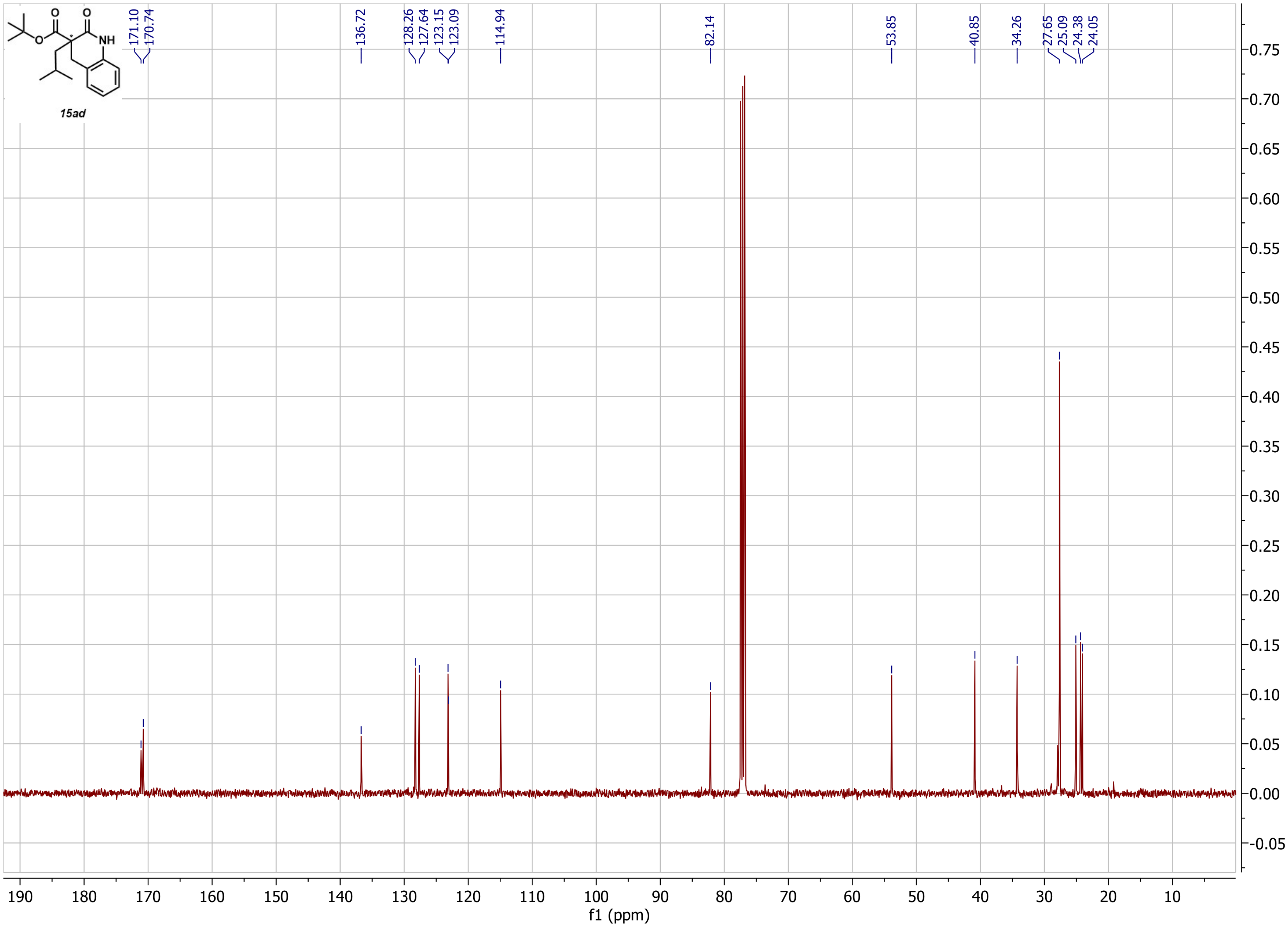

$^{13}C\{1H\}$ NMR. Solvent: $CDCl_3$. B_0 = 100 MHz.

*Compound **15ae***

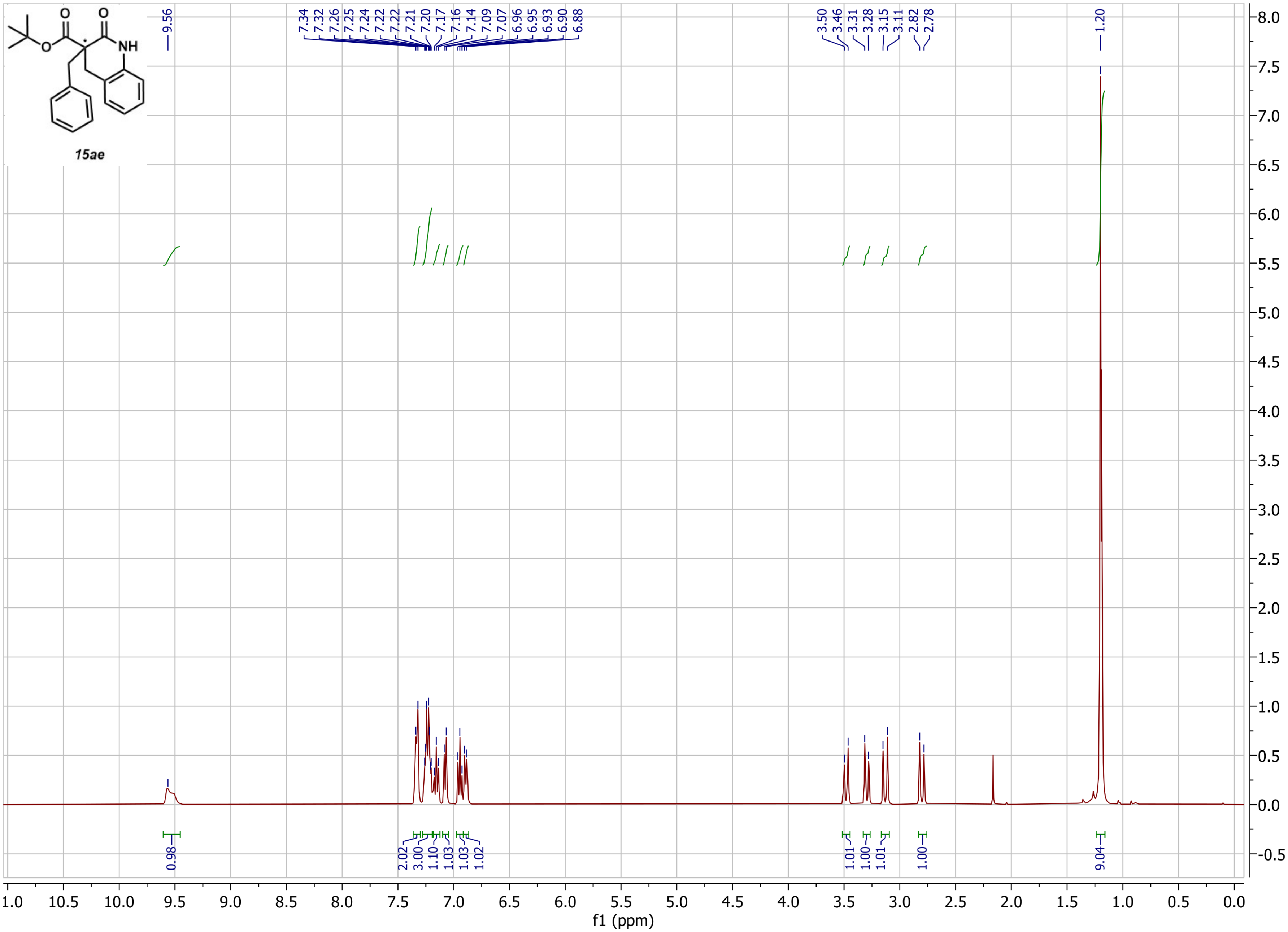

^{1}H NMR. Solvent: $CDCl_3$. B_0 = 400 MHz.

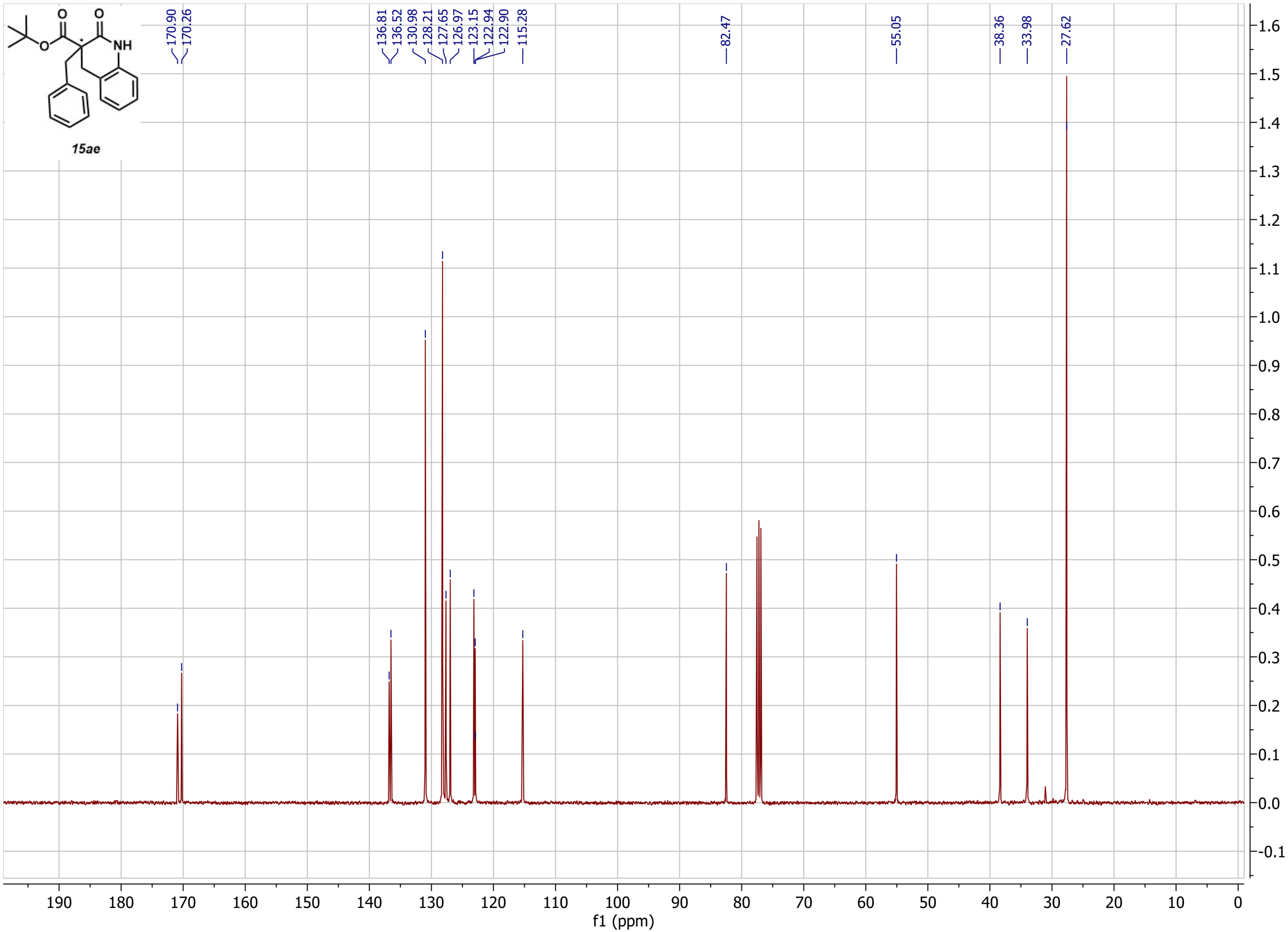

$^{13}C\{^1H\}$ NMR. Solvent: $CDCl_3$. B_0 = 100 MHz.

*Compound **15af***

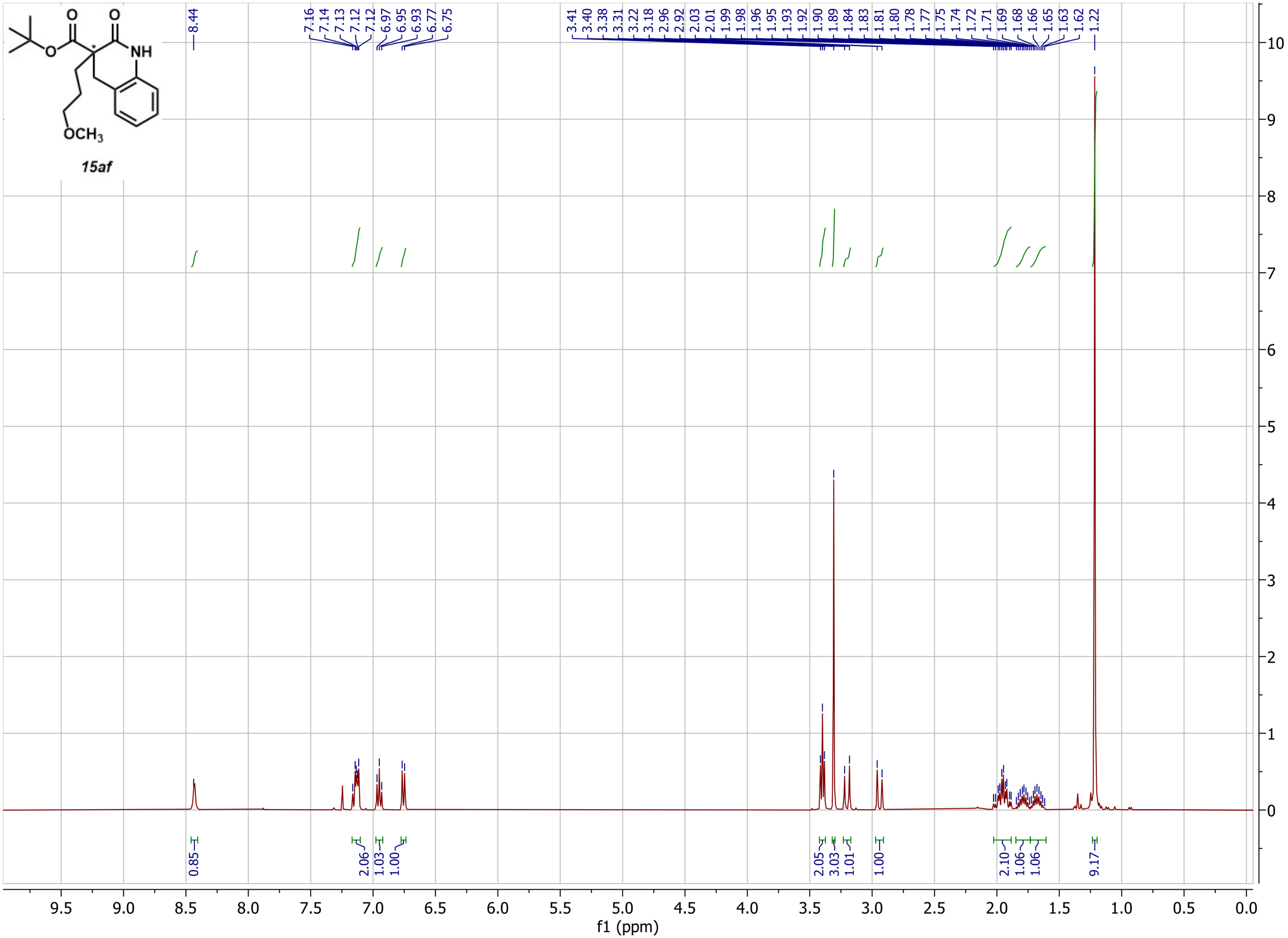

^{1}H NMR. Solvent: $CDCl_3$. B_0 = 400 MHz.

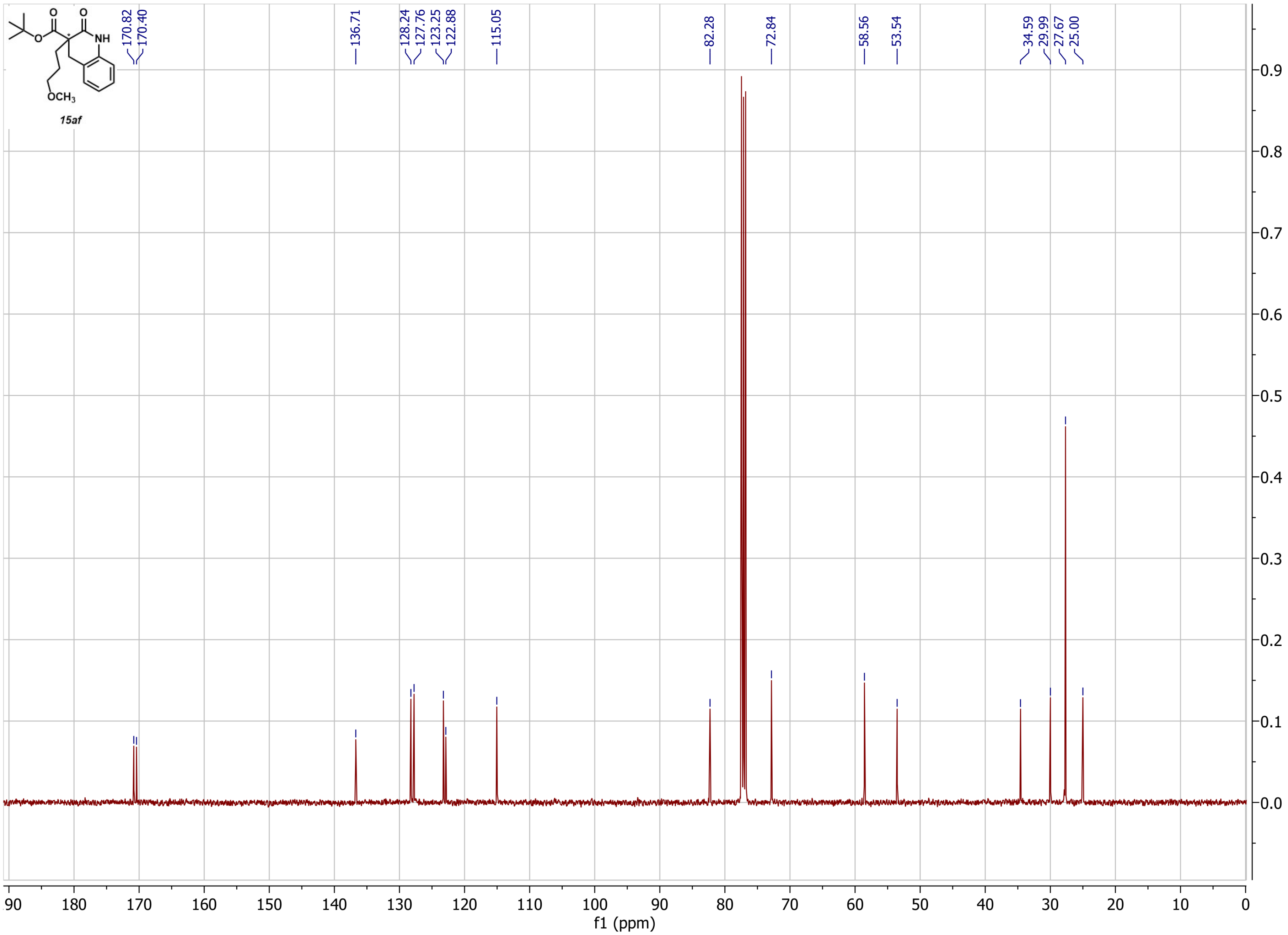

$^{13}C\{^1H\}$ NMR. Solvent: $CDCl_3$. B_0 = 100 MHz.

Compound ***15ba***

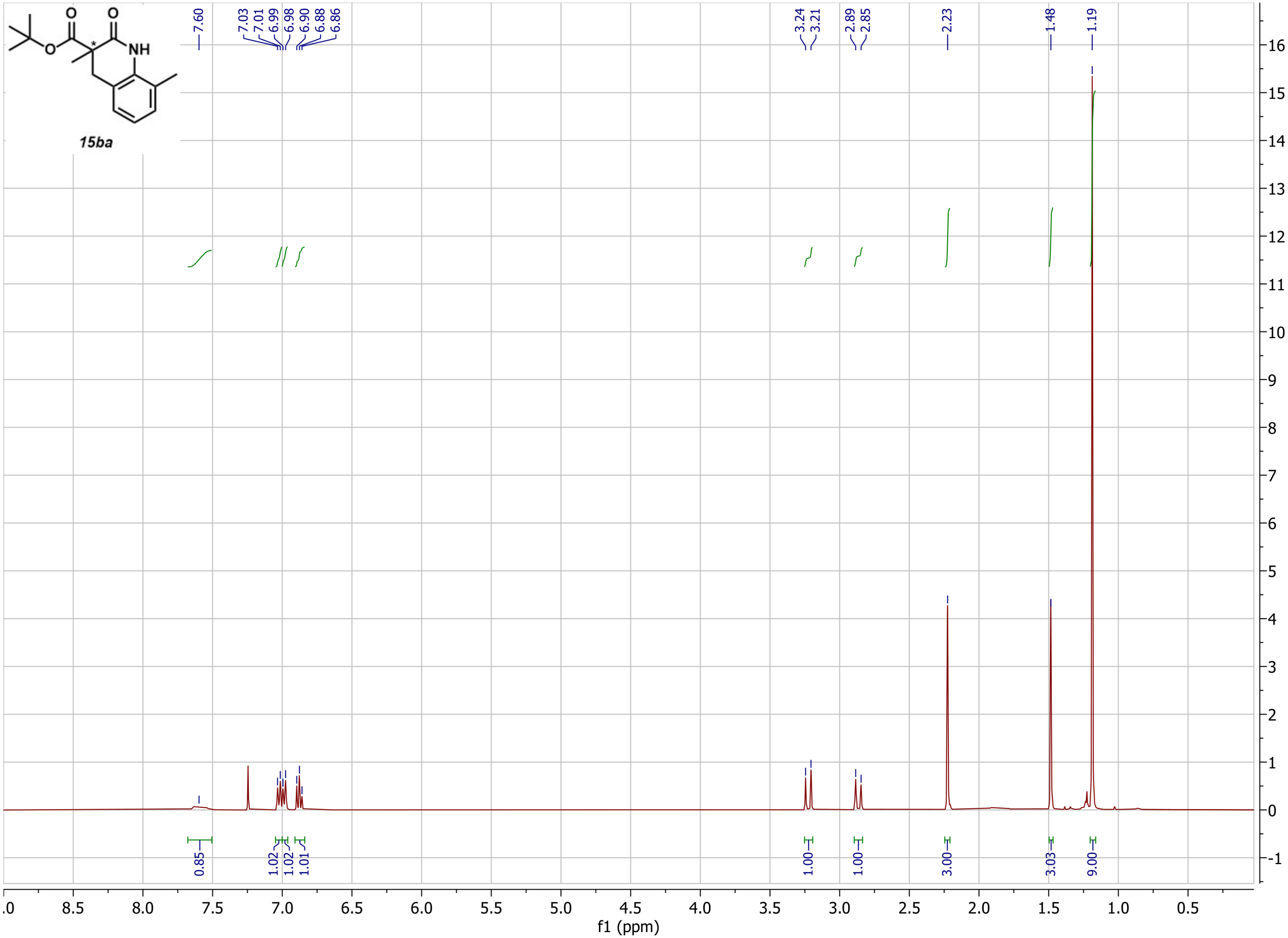

^{1}H NMR. Solvent: $CDCl_3$. B_0 = 400 MHz.

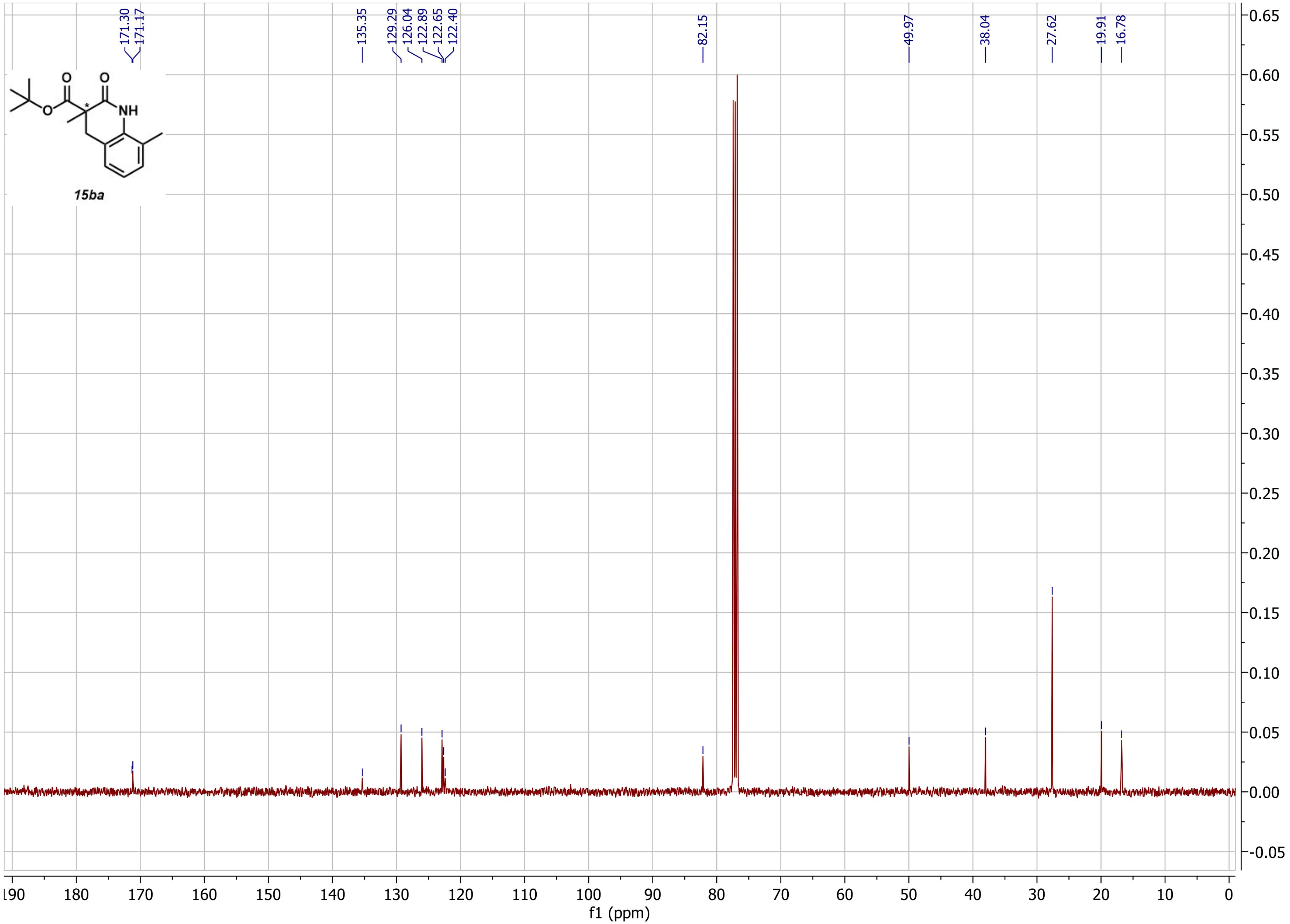

$^{13}C\{^{1}H\}$ NMR. Solvent: $CDCl_3$. B_0 = 100 MHz.

*Compound **15cb***

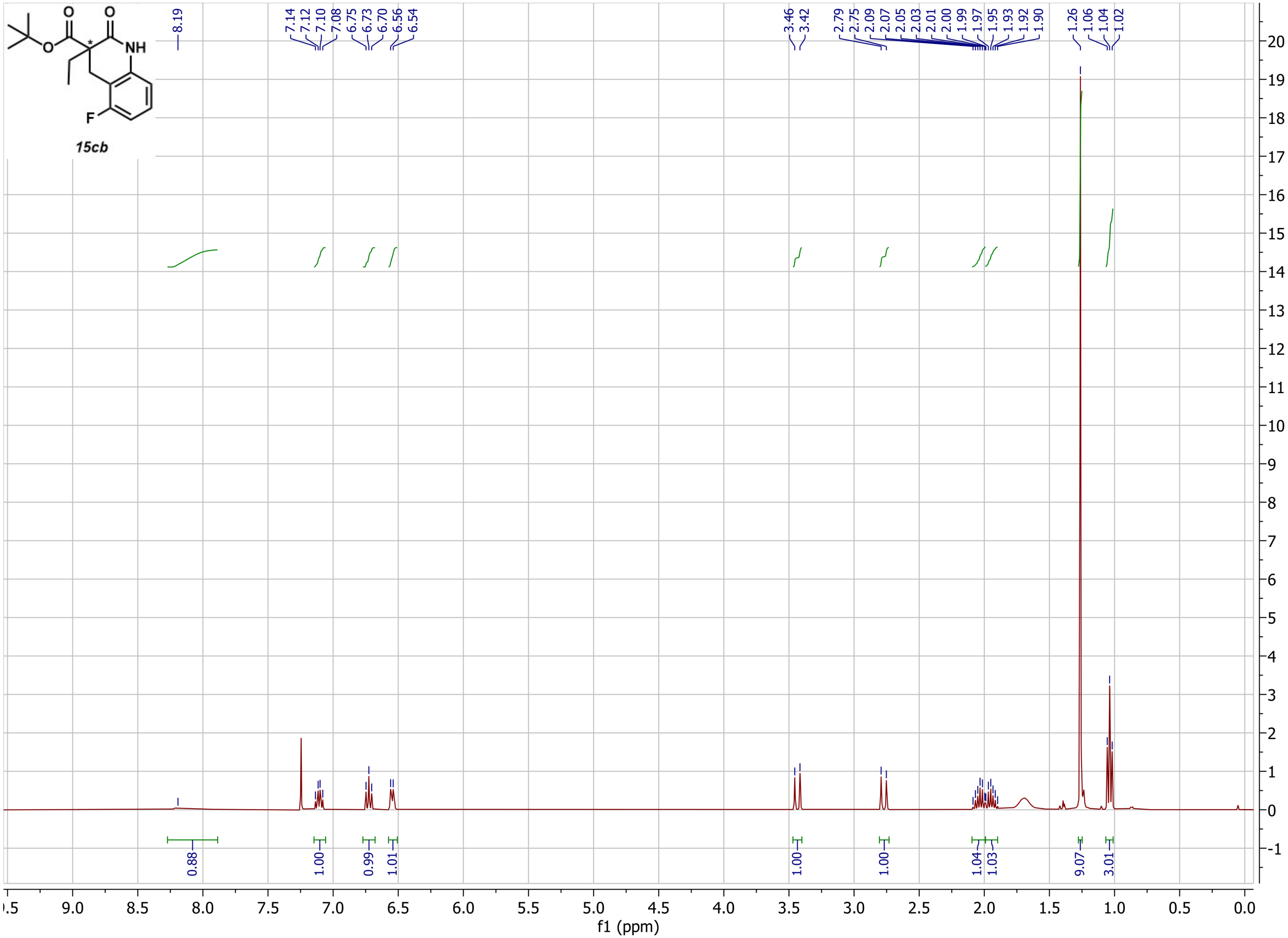

^{1}H NMR. Solvent: $CDCl_3$. B_0 = 400 MHz.

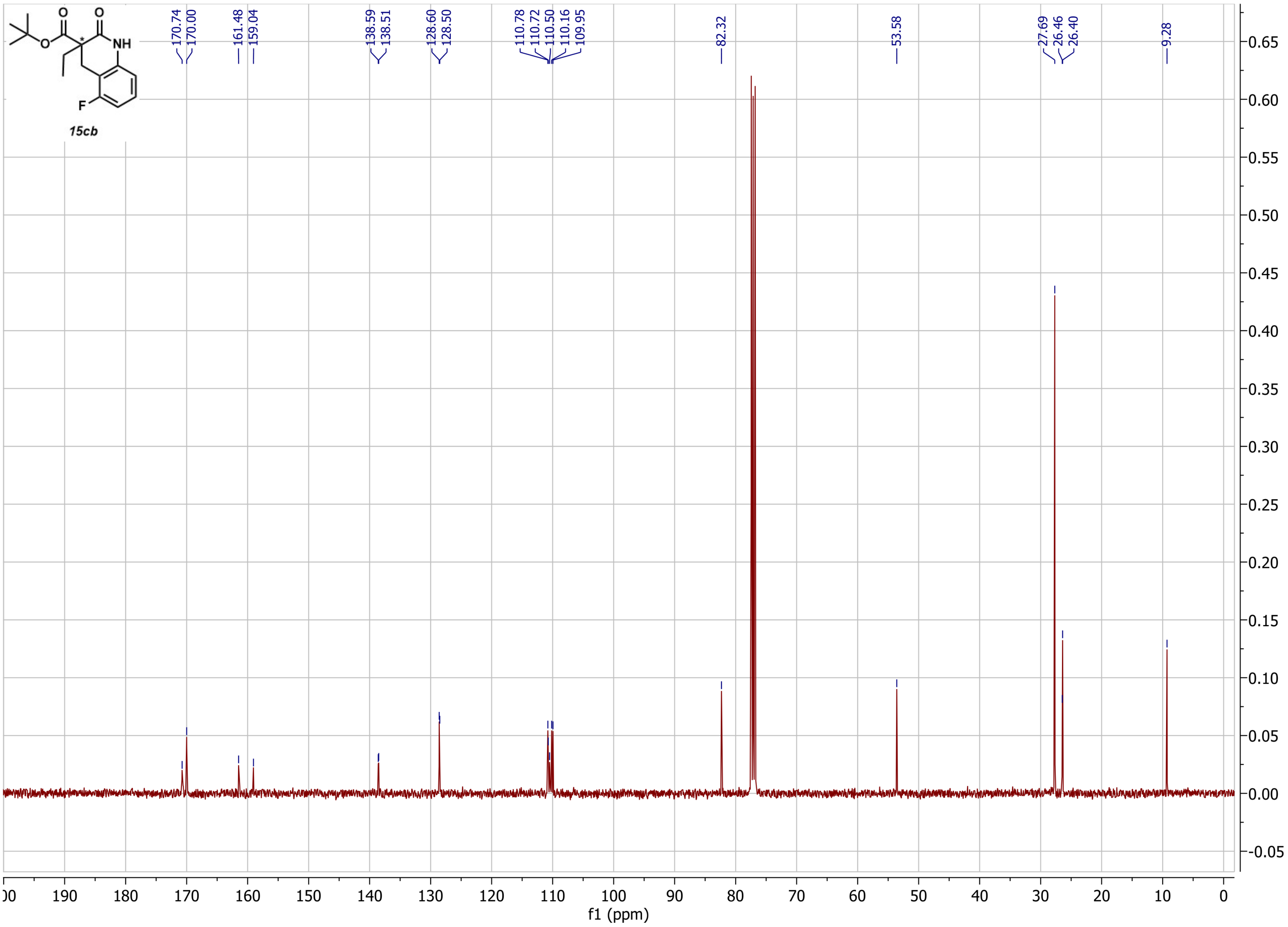

$^{13}C\{^1H\}$ NMR. Solvent: $CDCl_3$. B_0 = 100 MHz.

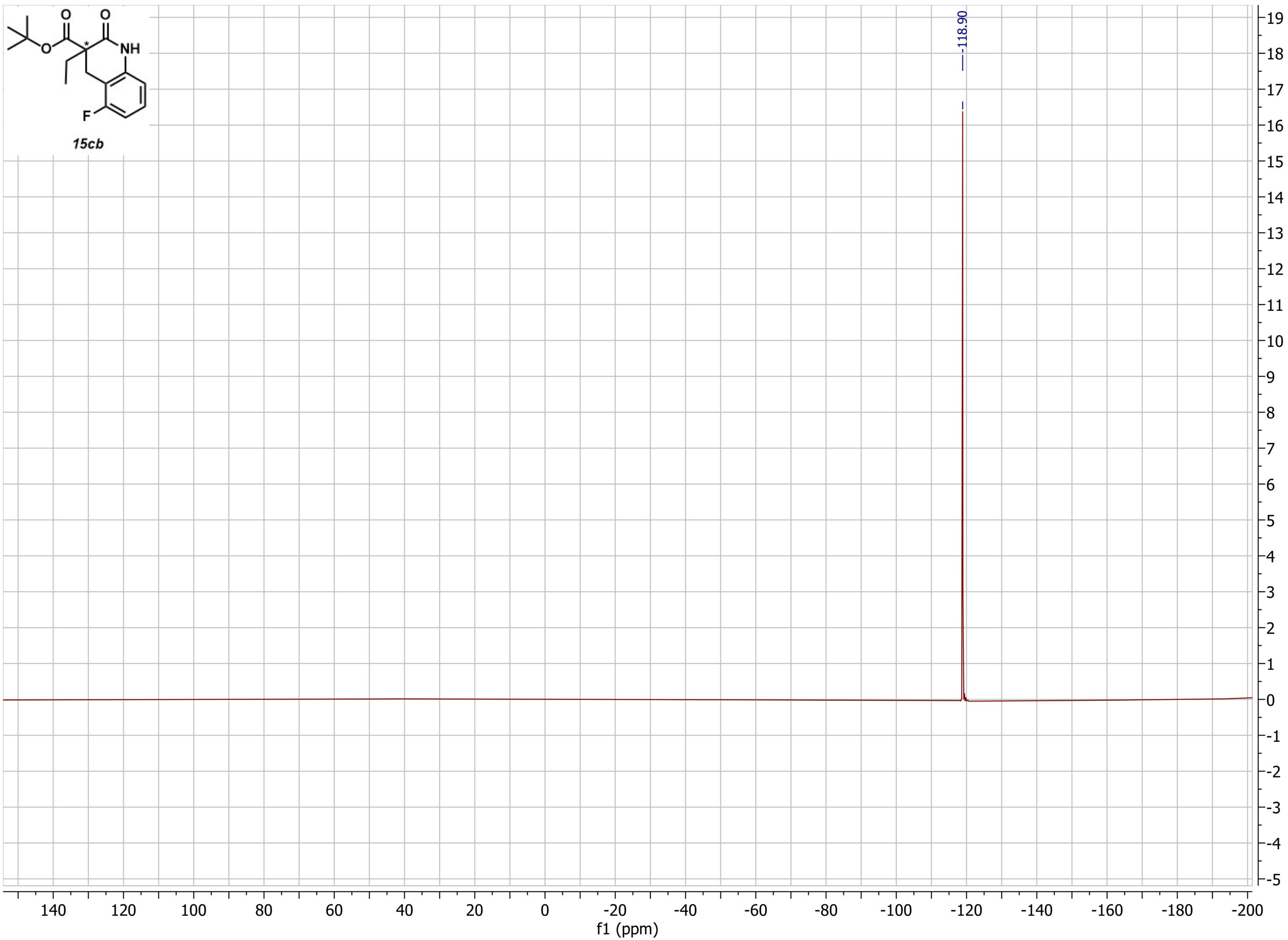

^{19}F NMR. Solvent: $CDCl_3$. B_0 = 376 MHz.

Compound ***15da***

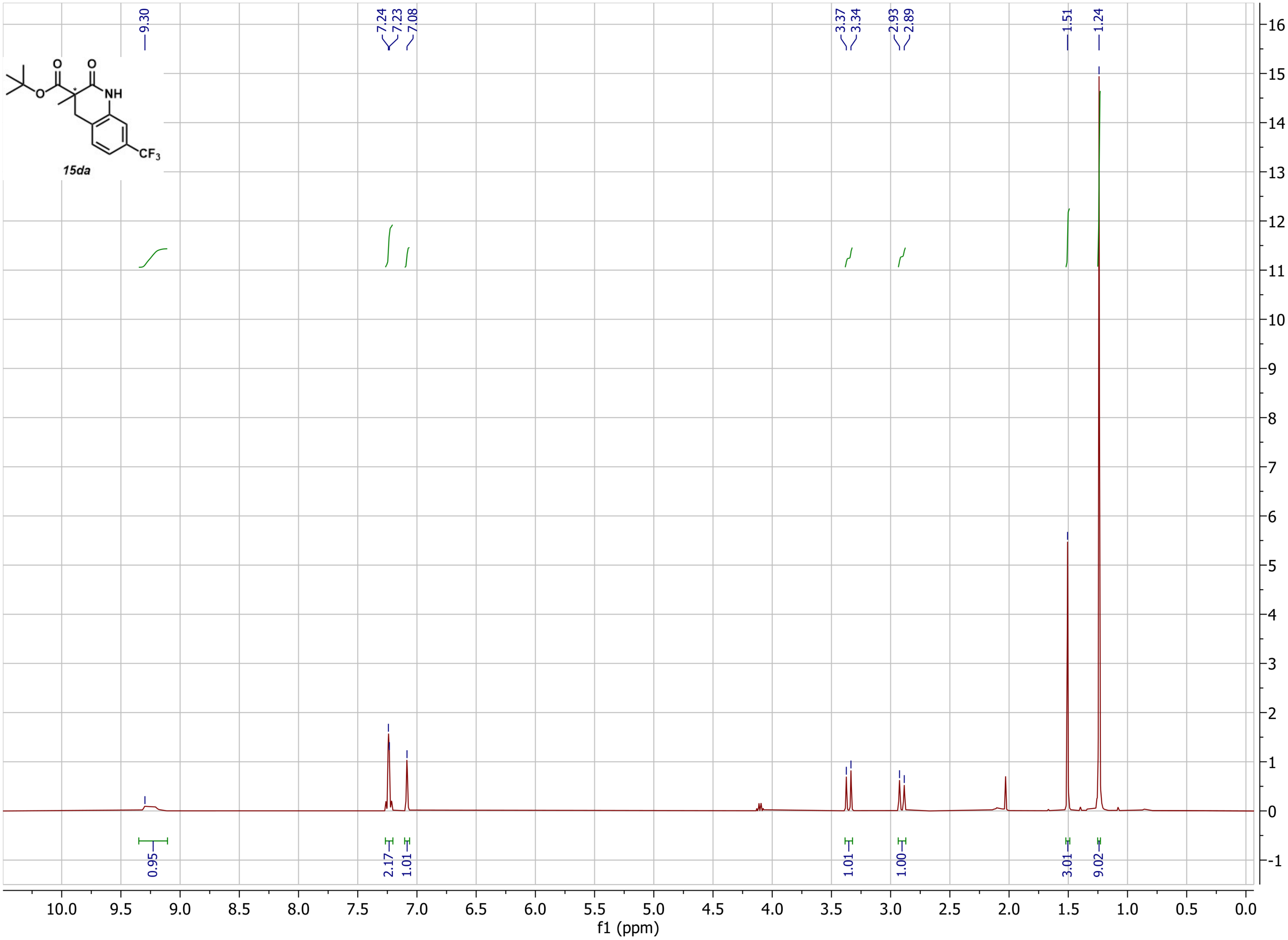

^{1}H NMR. Solvent: $CDCl_3$. B_0 = 400 MHz.

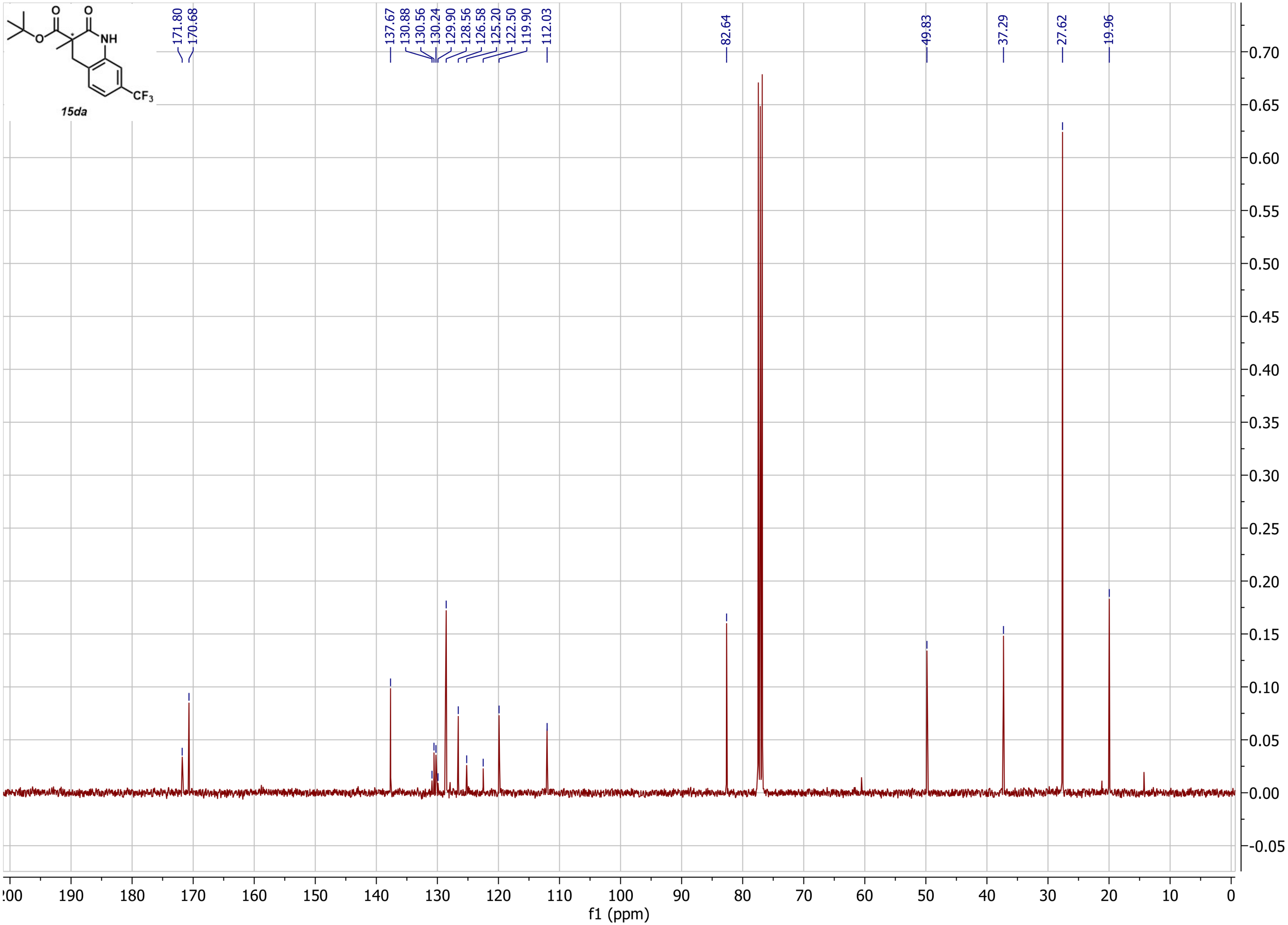

$^{13}C\{1H\}$ NMR. Solvent: $CDCl_3$. B_0 = 100 MHz.

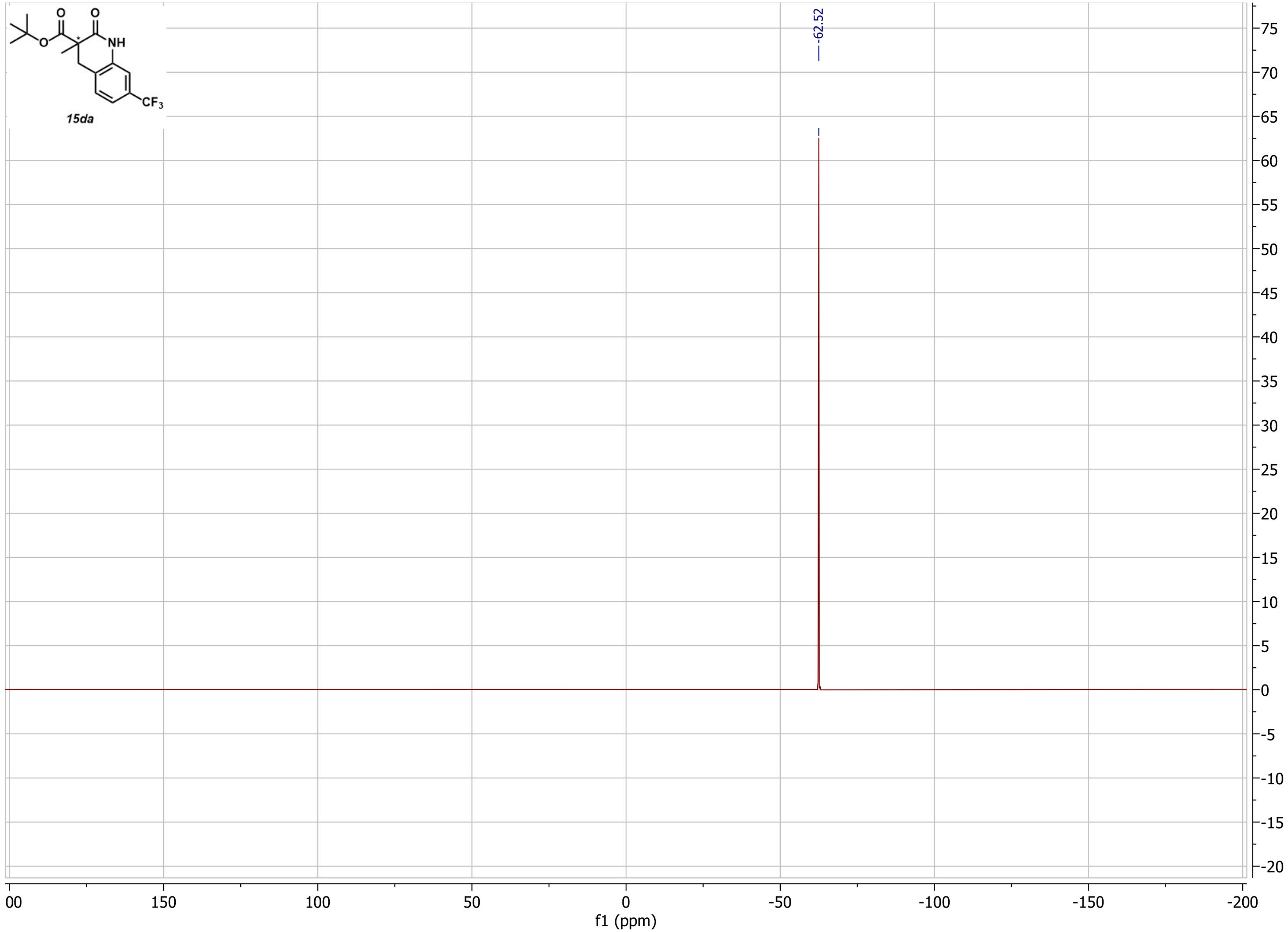

^{19}F NMR. Solvent: $CDCl_3$. B_0 = 376 MHz.

*Compound **15ea***

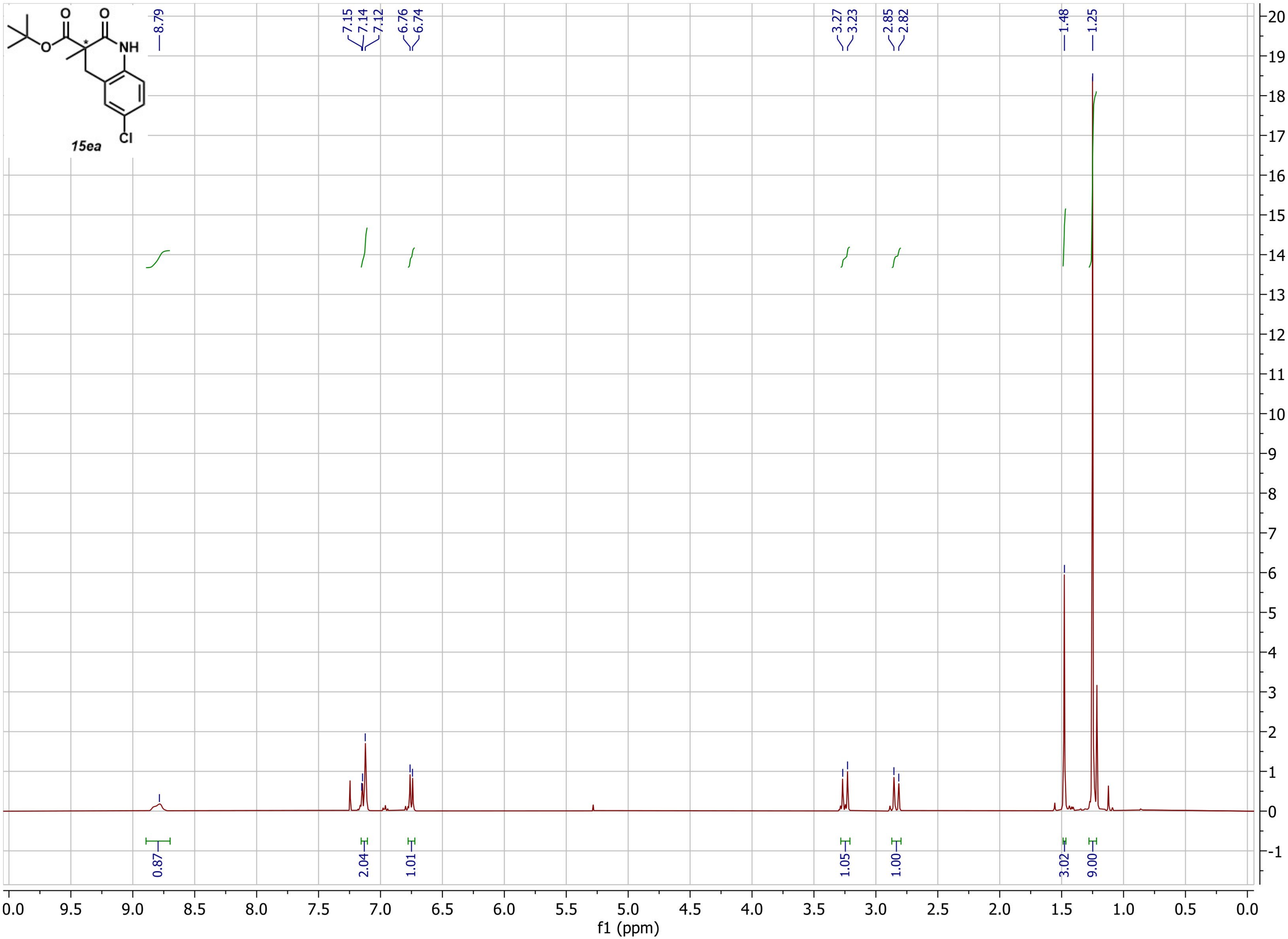

^{1}H NMR. Solvent: $CDCl_3$. B_0 = 400 MHz.

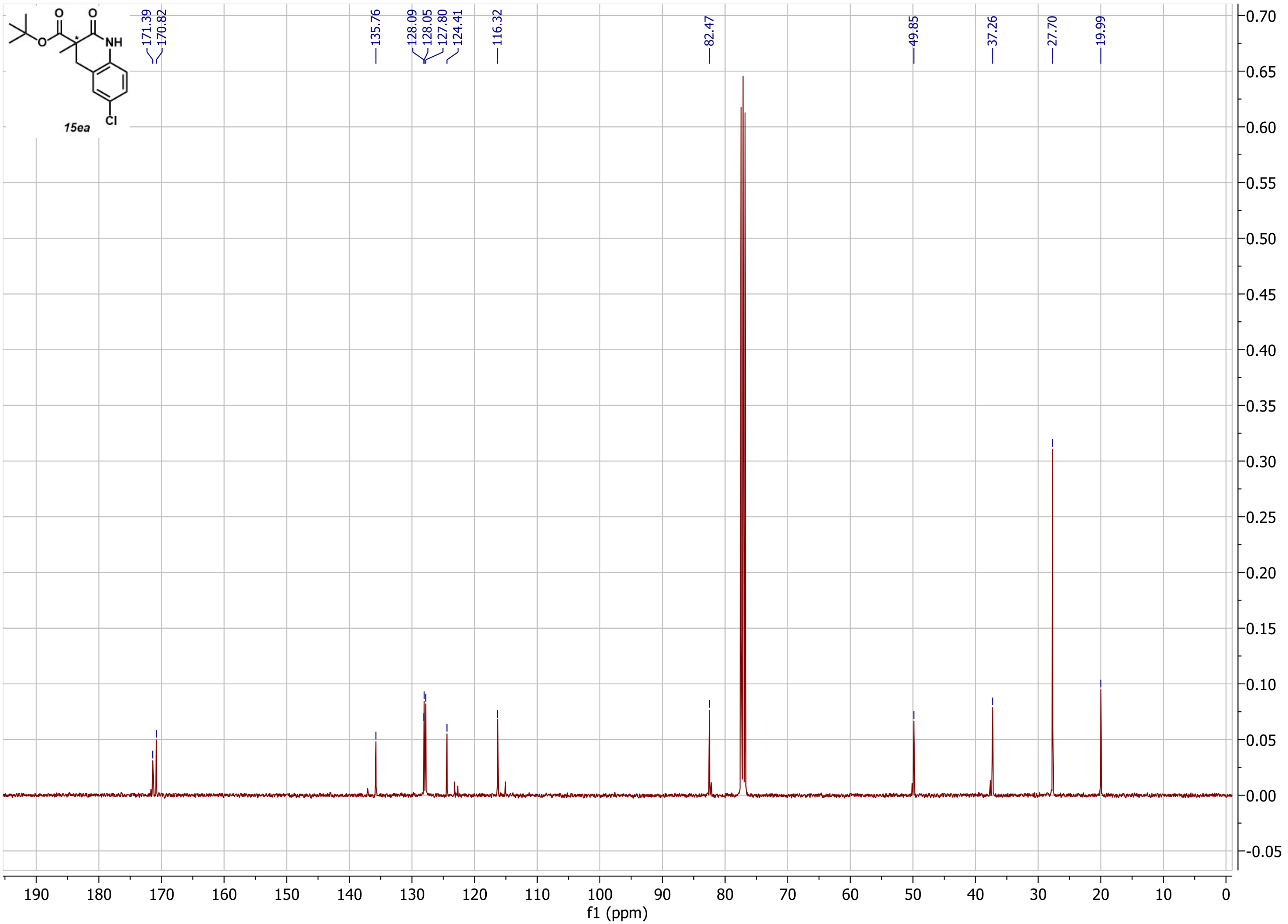

$^{13}C\{^1H\}$ NMR. Solvent: $CDCl_3$. B_0 = 100 MHz.

*Compound **15eb***

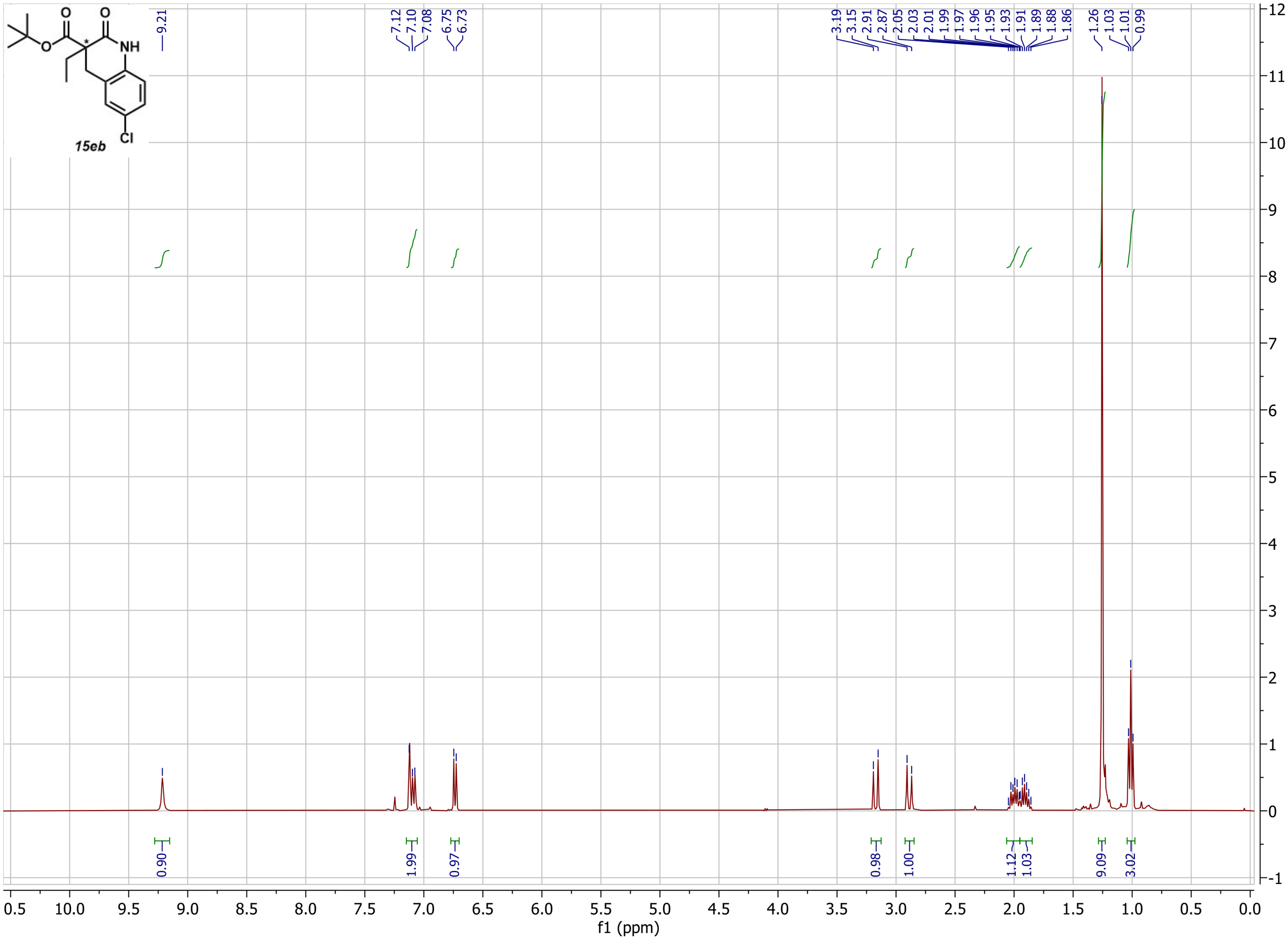

^{1}H NMR. Solvent: $CDCl_3$. B_0 = 400 MHz.

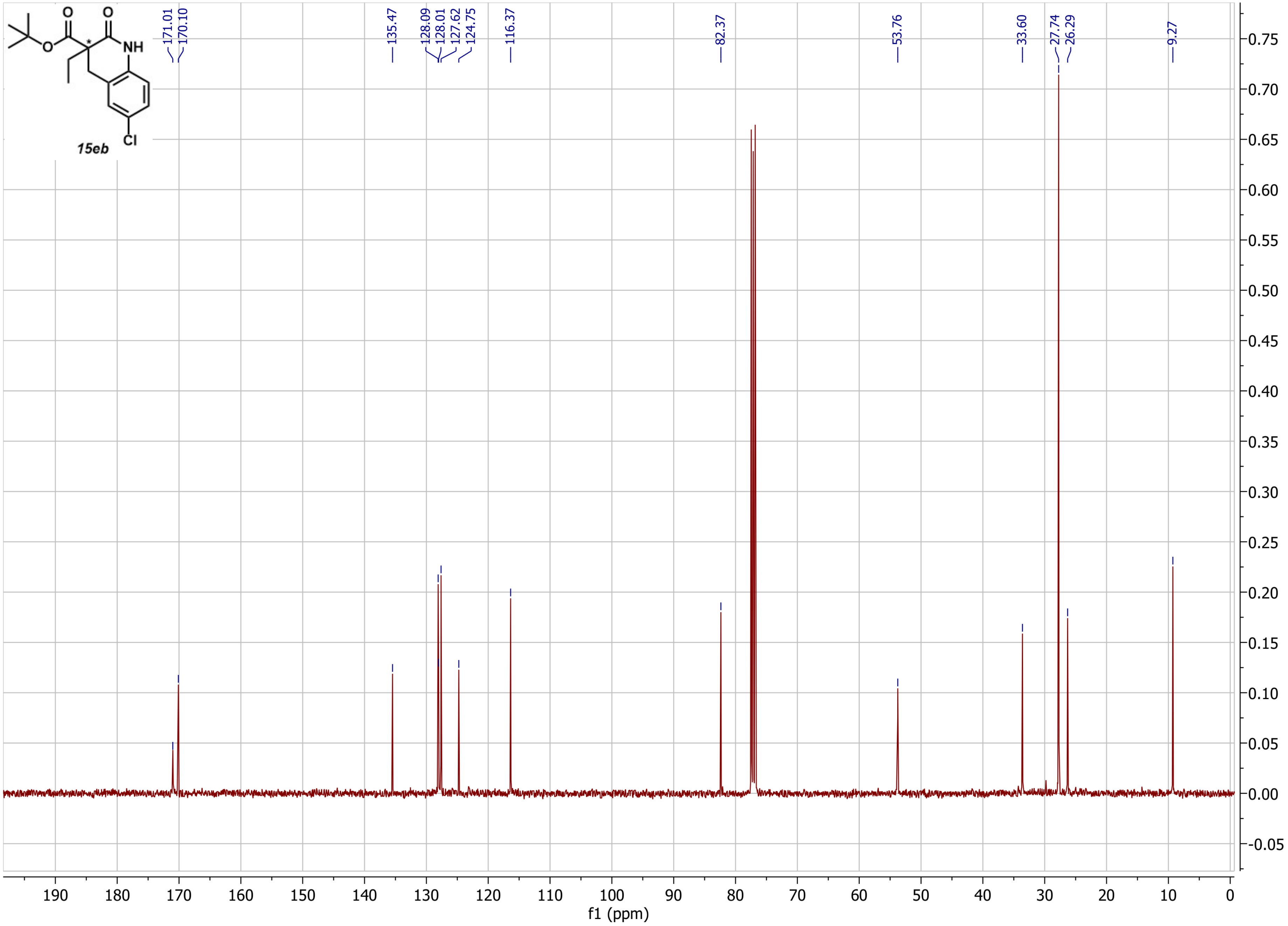

$^{13}C\{^1H\}$ NMR. Solvent: $CDCl_3$. B_0 = 100 MHz.

Compound ***15fa***

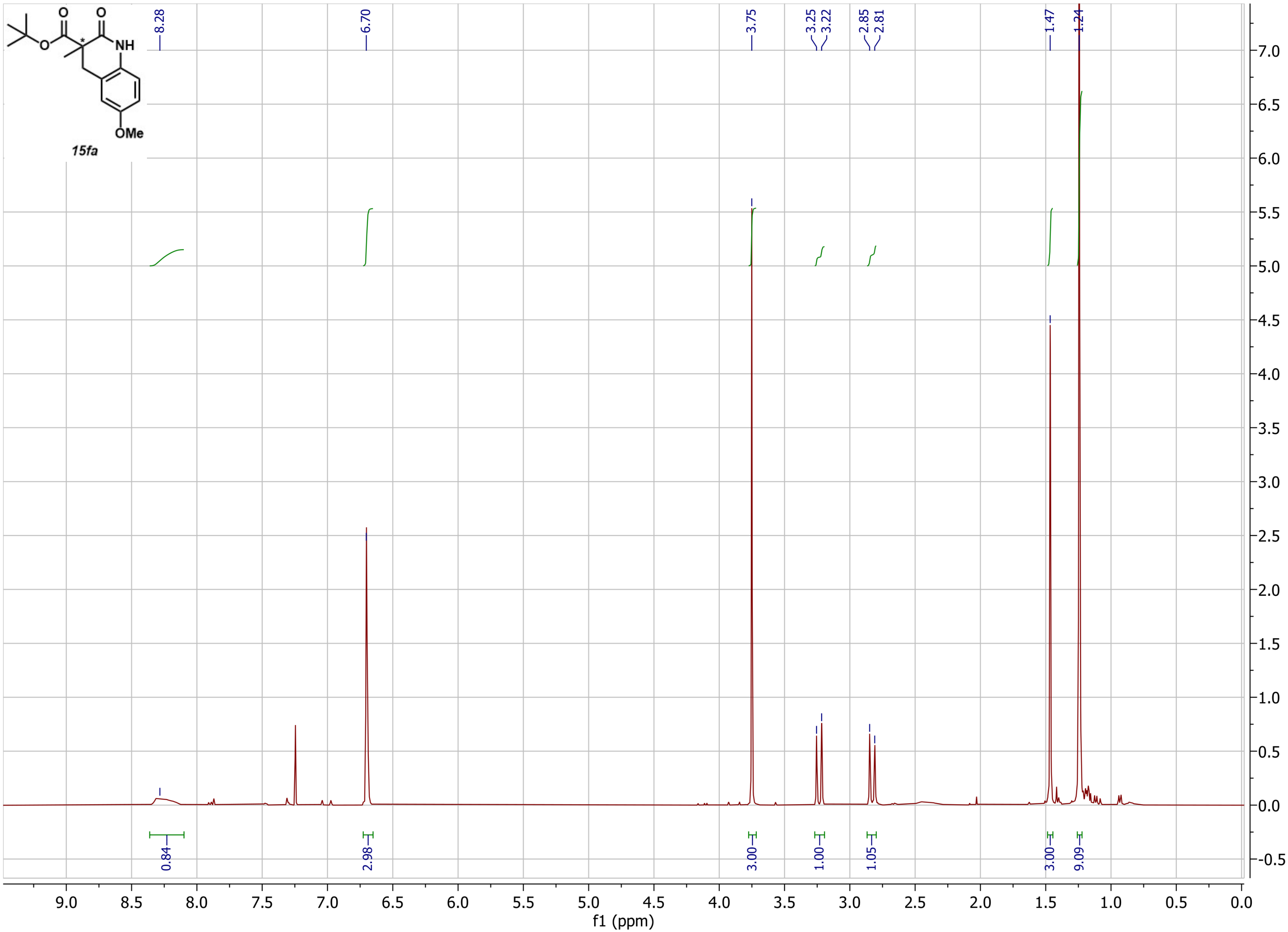

^{1}H NMR. Solvent: $CDCl_3$. B_0 = 400 MHz.

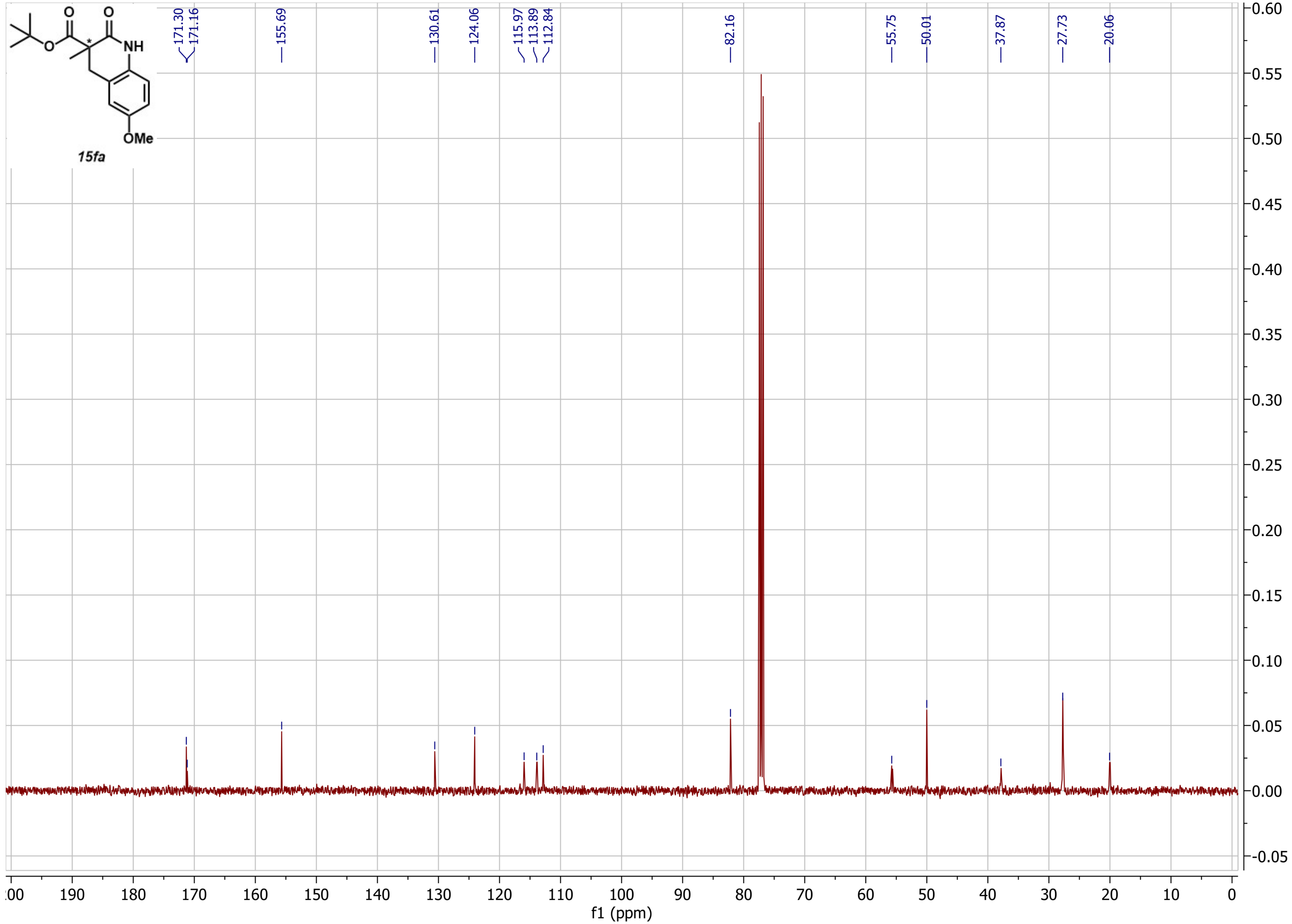

$^{13}C\{1H\}$ NMR. Solvent: $CDCl_3$. B_0 = 100 MHz.

*Compound **22aa***

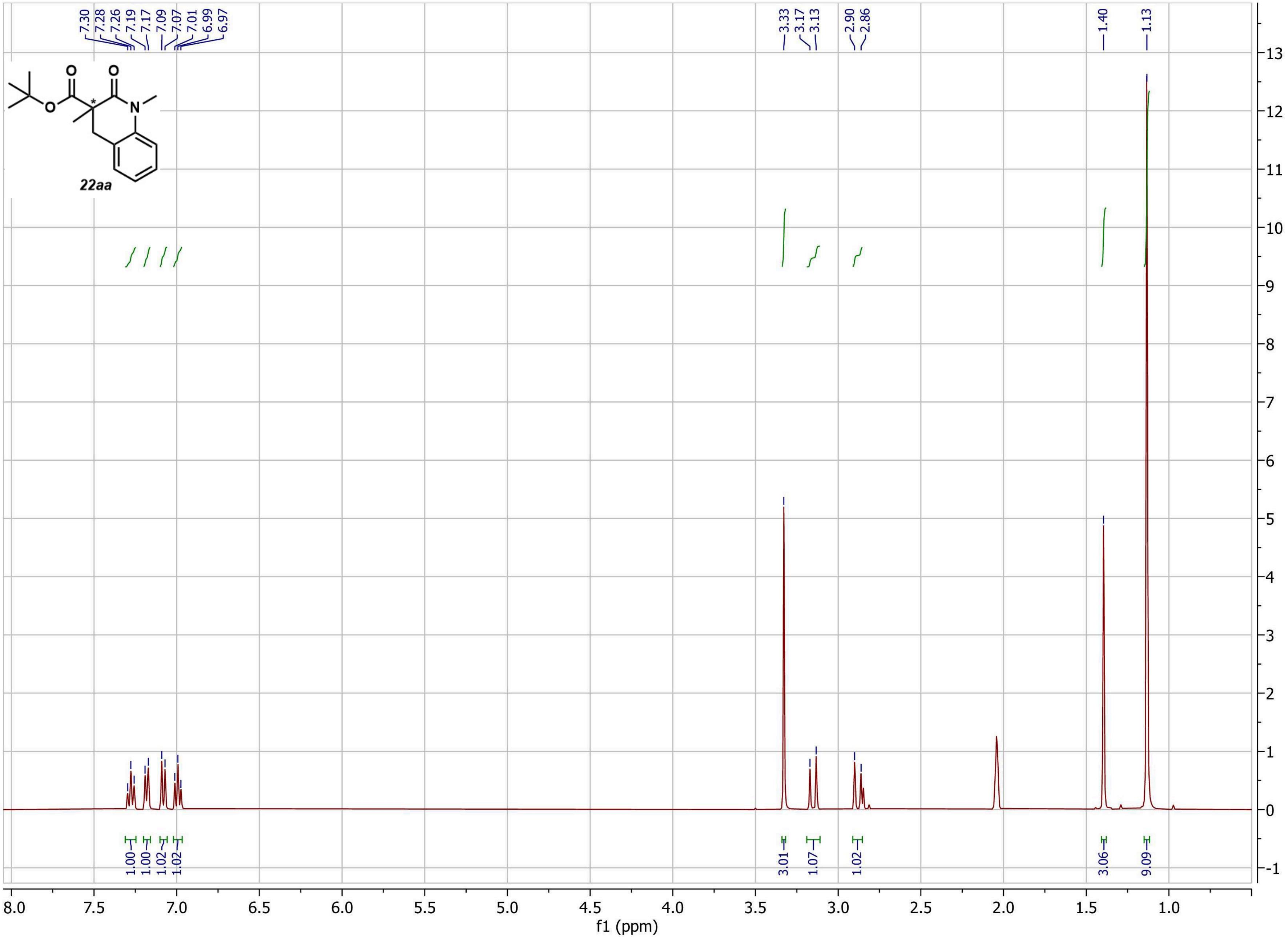

^1H NMR. Solvent: Acetone-d_6. B_0 = 400 MHz.

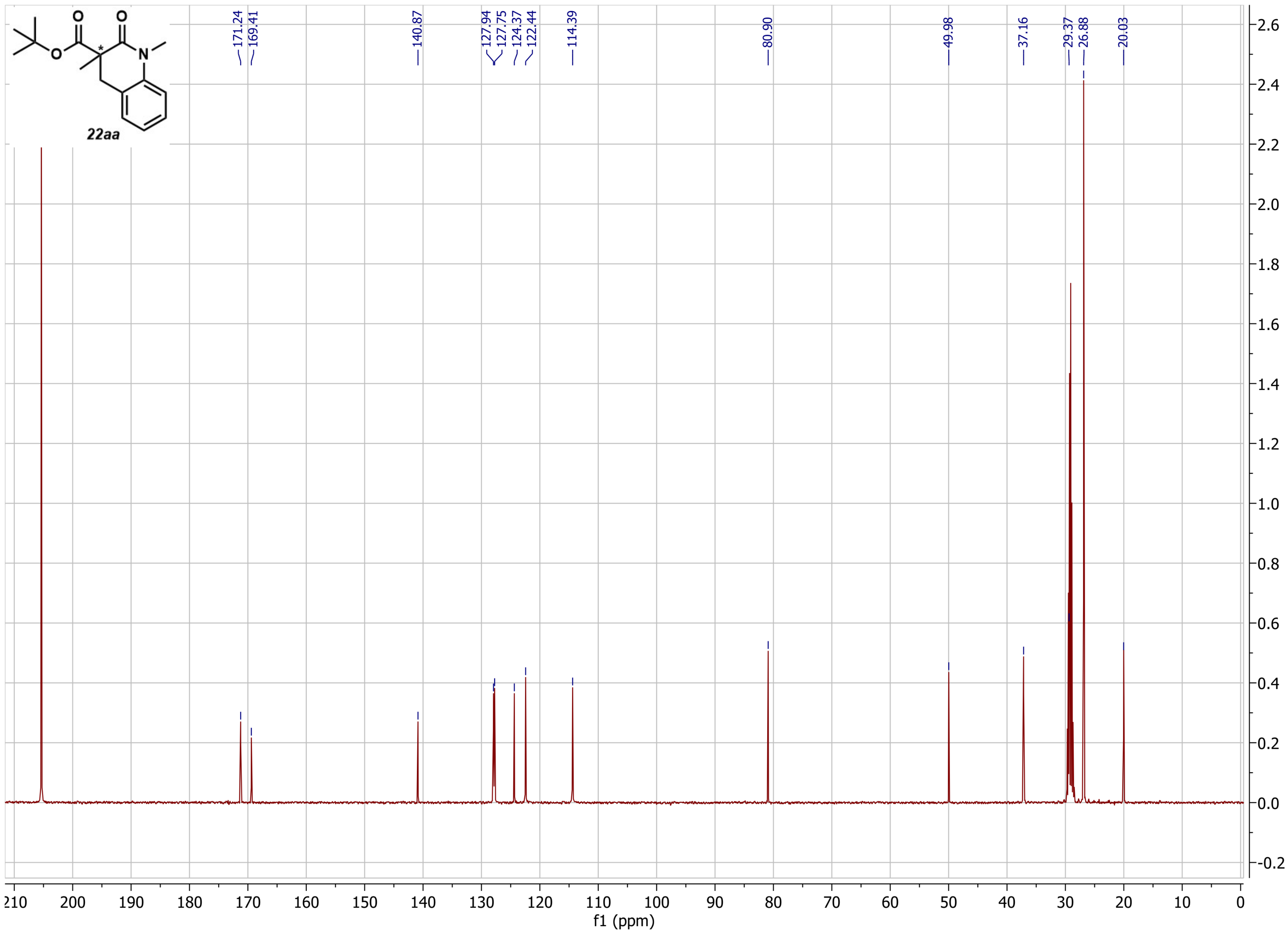

$^{13}C\{1H\}$ NMR. Solvent: Acetone-d_6. B_0 = 100 MHz.

*Compound **23aa***

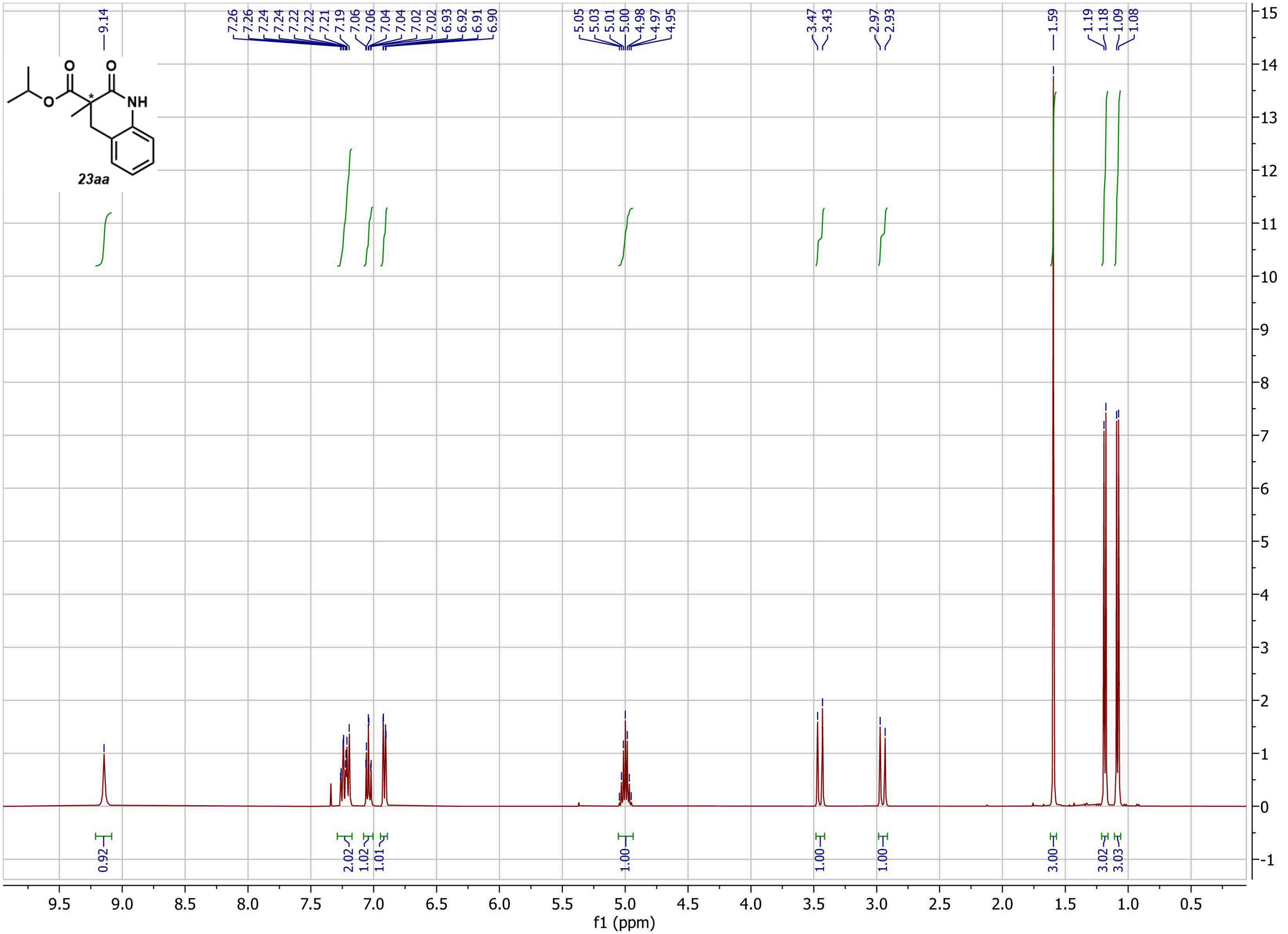

23aa

^1H NMR. Solvent: $CDCl_3$. B_0 = 400 MHz.

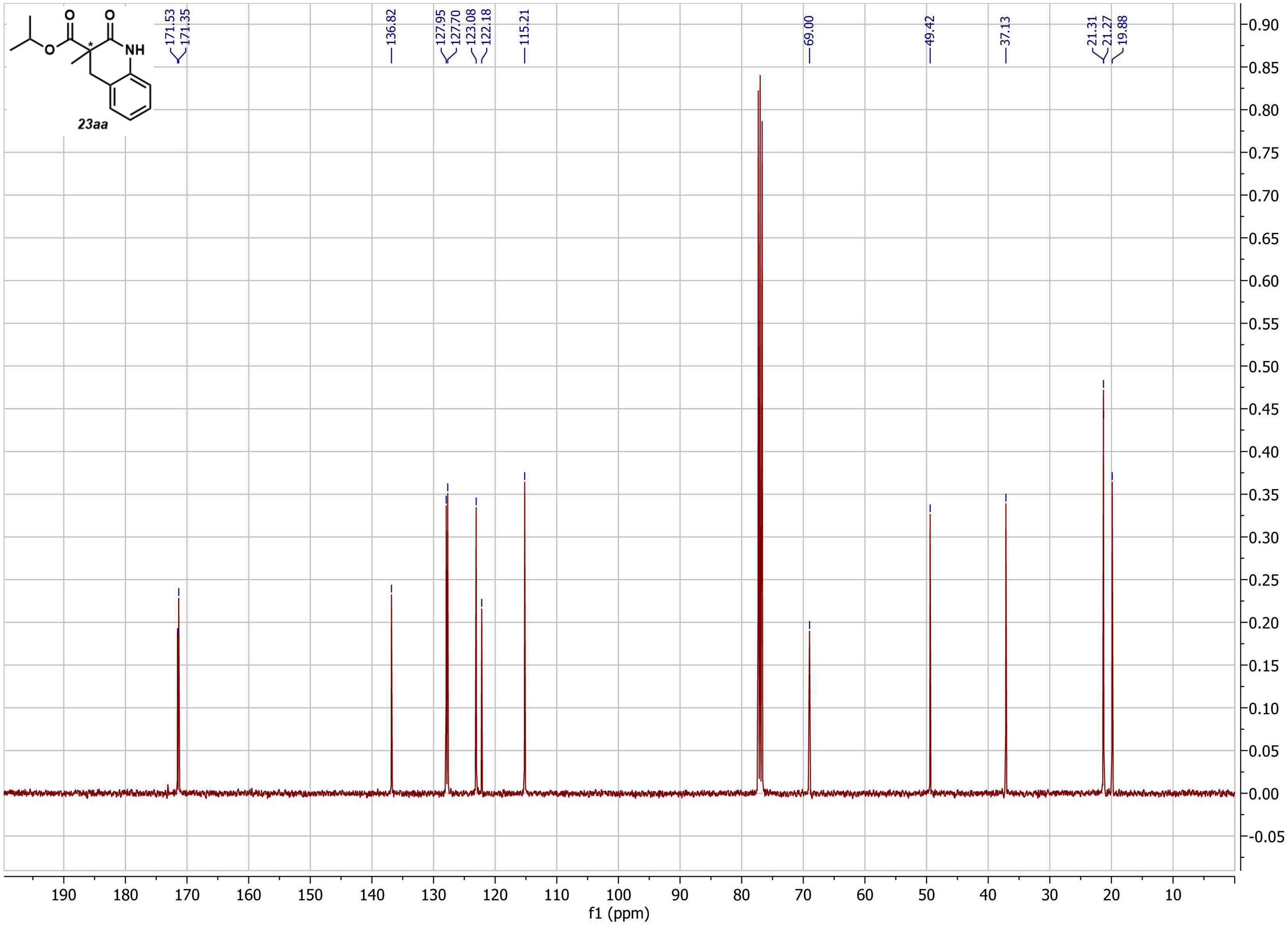

$^{13}C\{1H\}$ NMR. Solvent: $CDCl_3$. B_0 = 100 MHz.

*Compound **26***

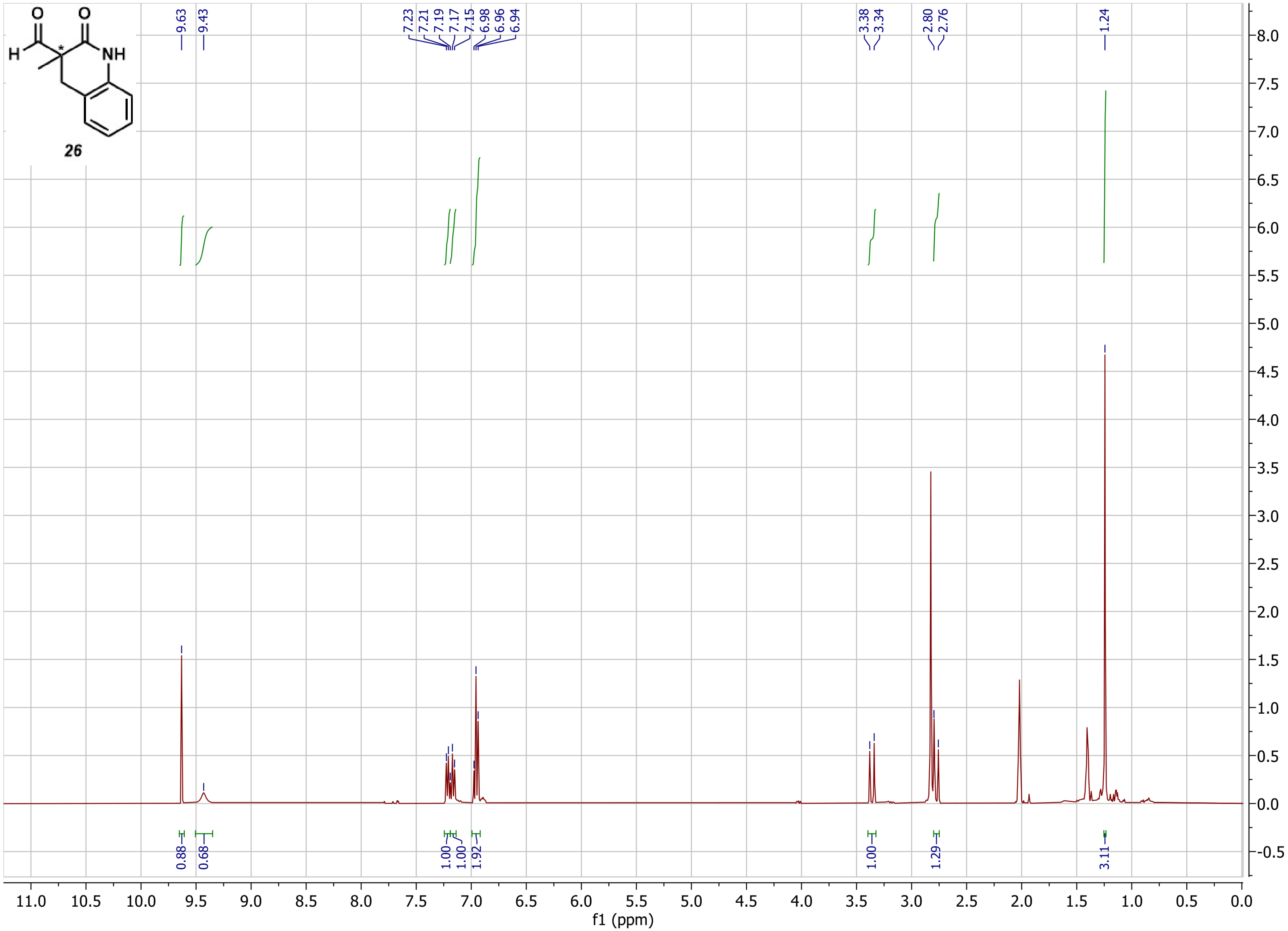

^{1}H NMR. Solvent: Acetone-d_6. B_0 = 400 MHz.

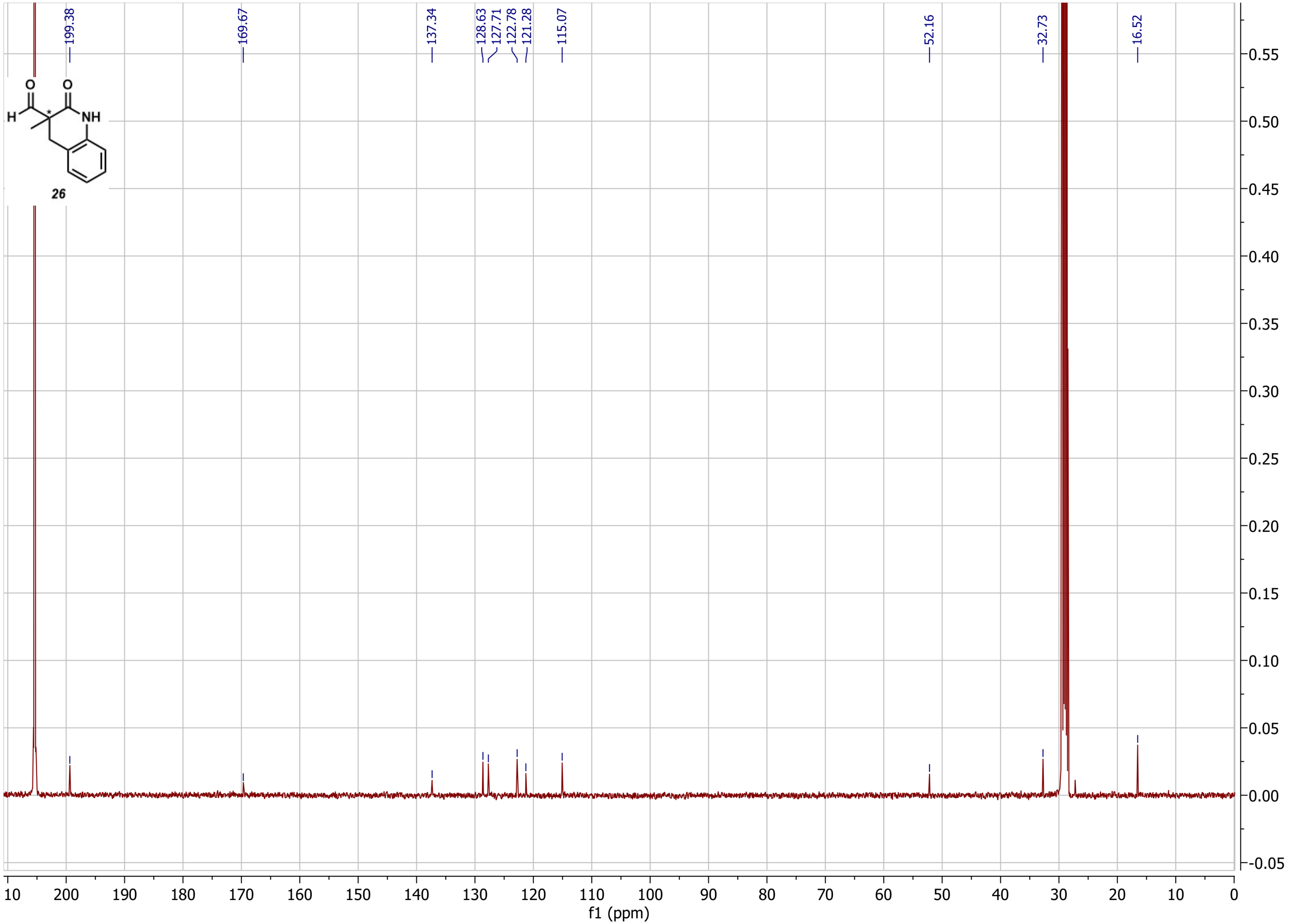

$^{13}C\{^1H\}$ NMR. Solvent: Acetone-d_6. B_0 = 100 MHz.

Compound ***27***

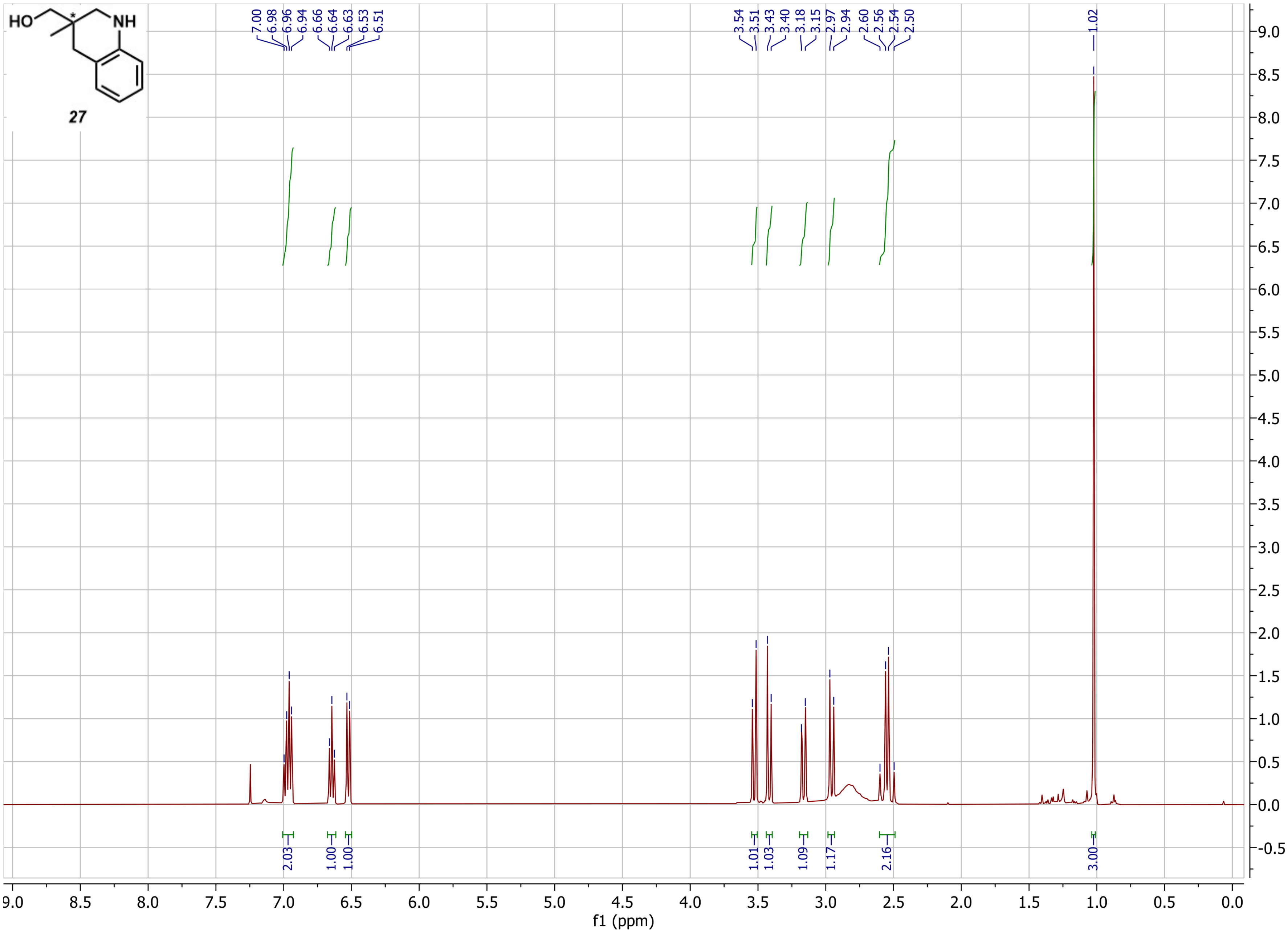

^{1}H NMR. Solvent: $CDCl_3$. B_0 = 400 MHz.

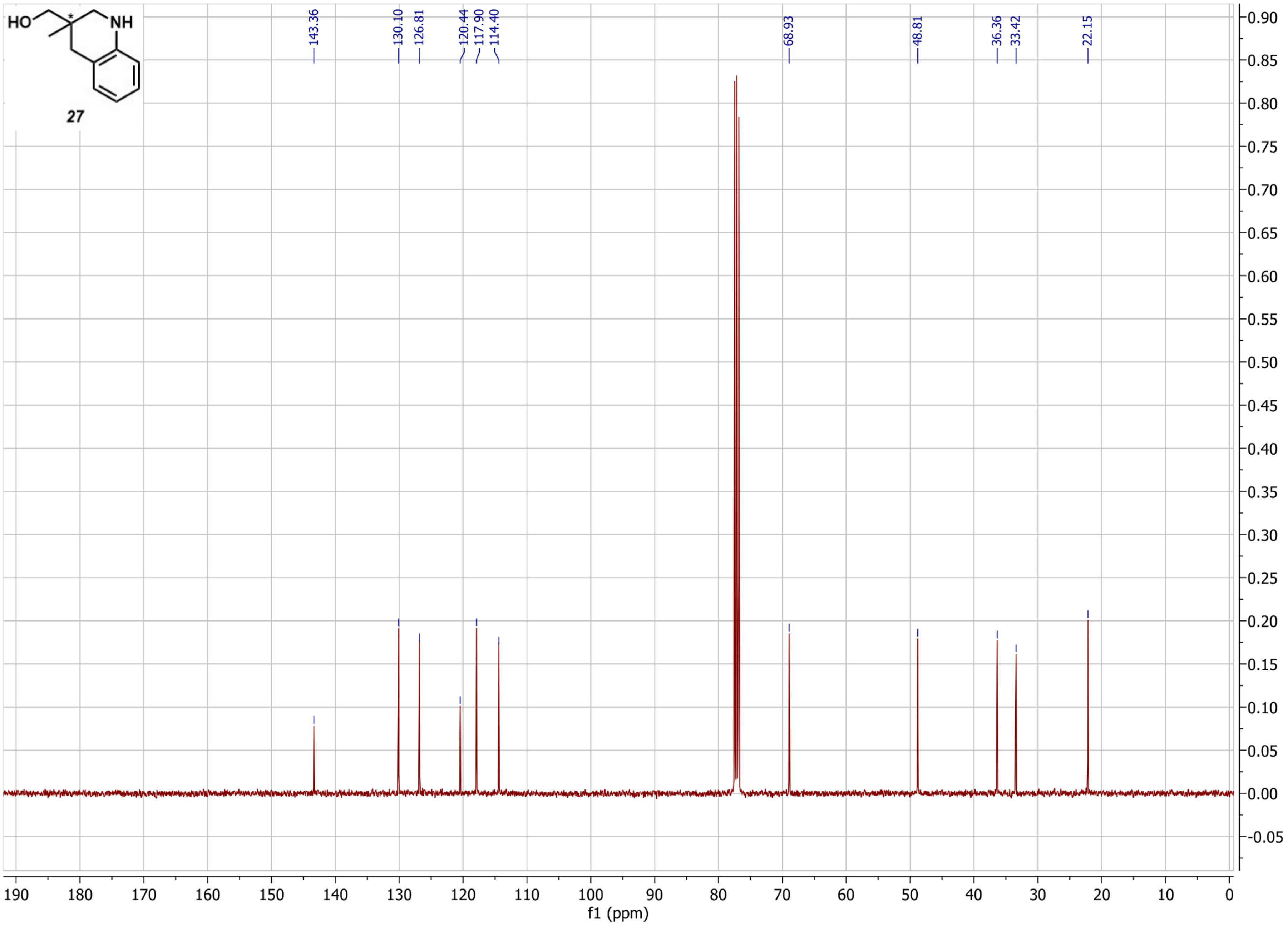

$^{13}C\{1H\}$ NMR. Solvent: $CDCl_3$. B_0 = 100 MHz.

Compound ***28***

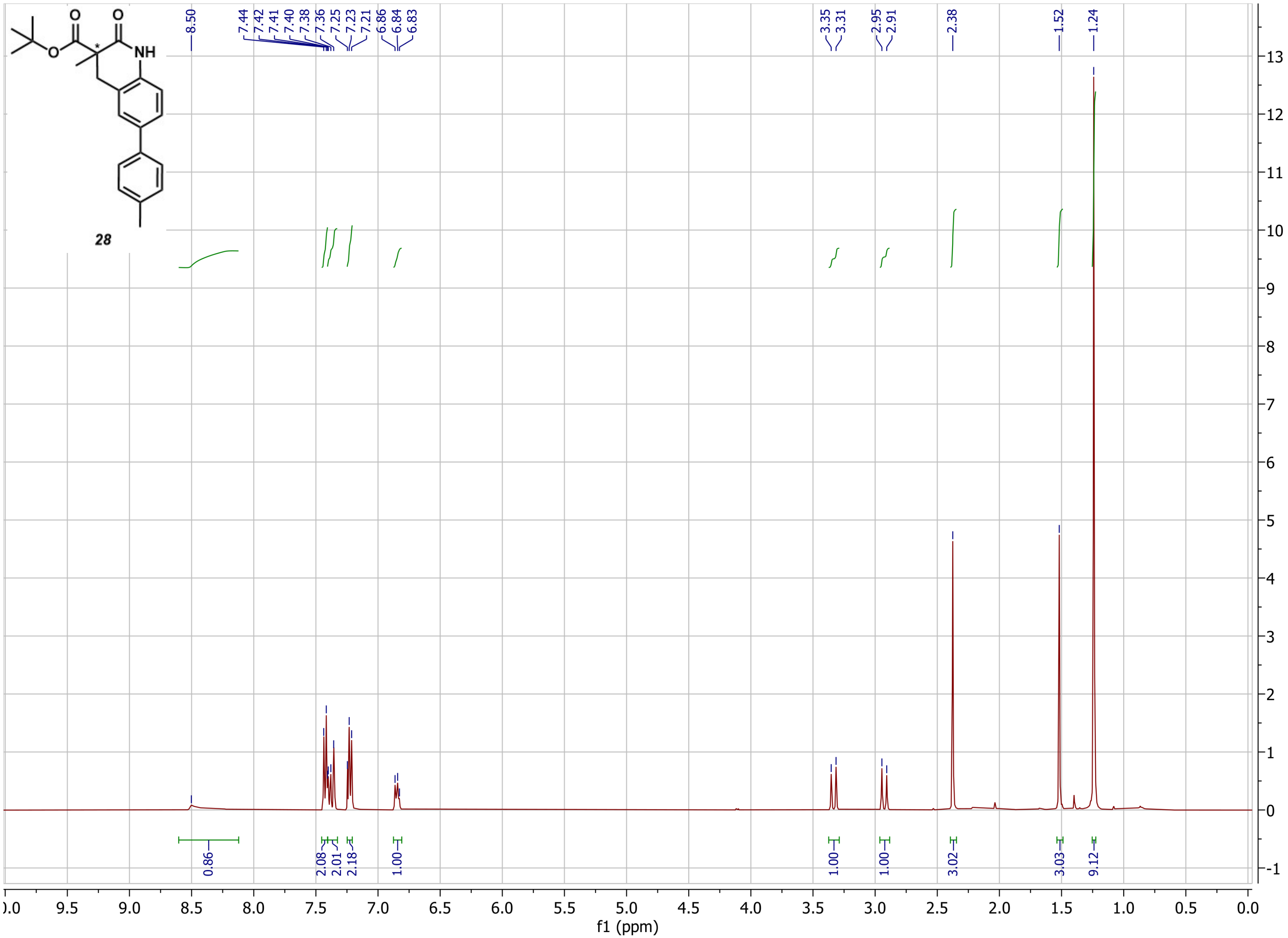

^{1}H NMR. Solvent: $CDCl_3$. B_0 = 400 MHz.

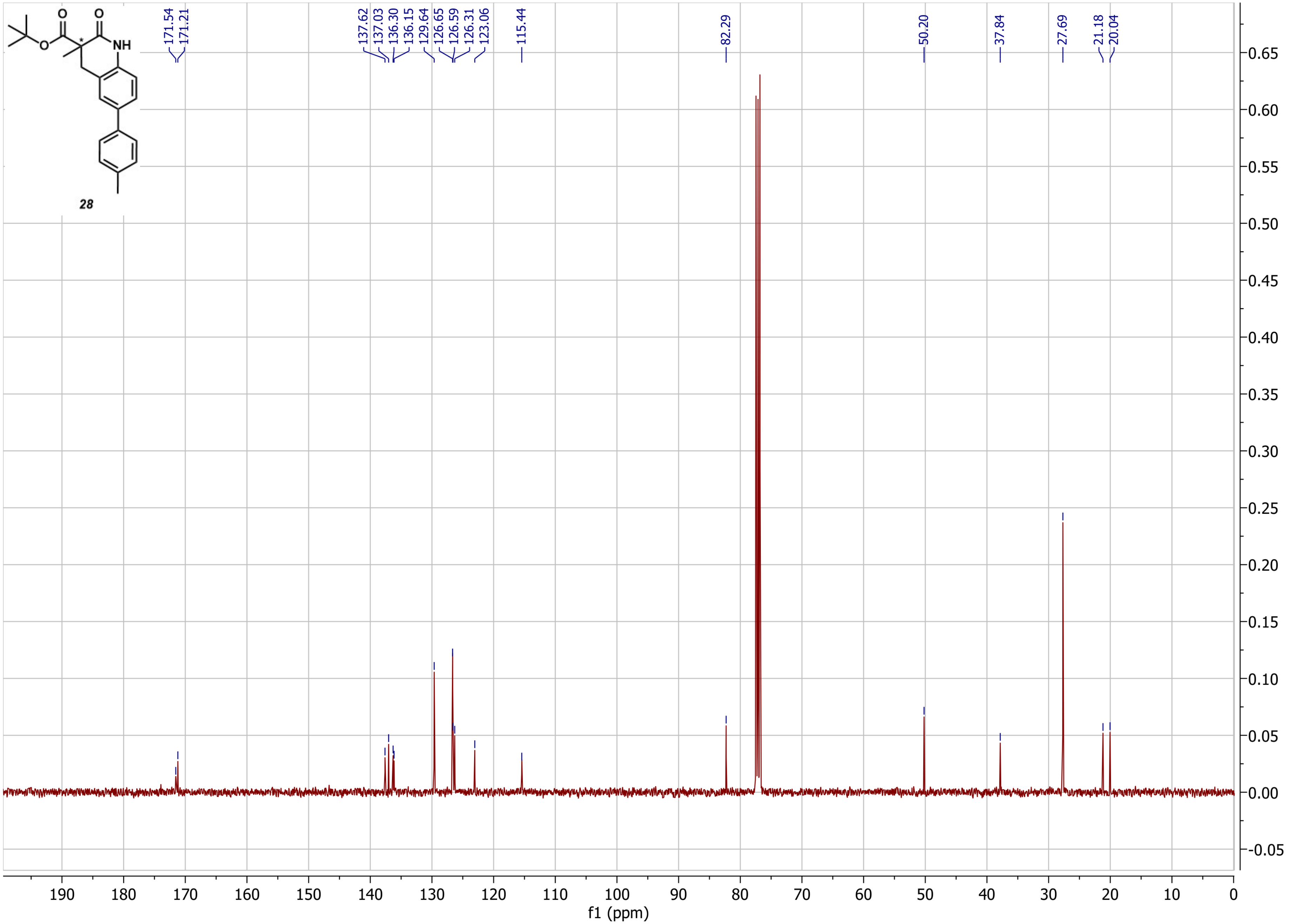

$^{13}C\{^1H\}$ NMR. Solvent: $CDCl_3$. B_0 = 100 MHz.

Copies of Chiral Chromatograms
All HPLC traces run on Chiralpak AD-H 4.6 mm x 250 mm x 5 µm column.

Racemic Compound **15aa**
HPLC (OD-H, isopropanol/n-hexanes = 10/90, flow rate = 1.0 mL/min, l = 254 nm) tR = 7.94 min, 12.46 min.

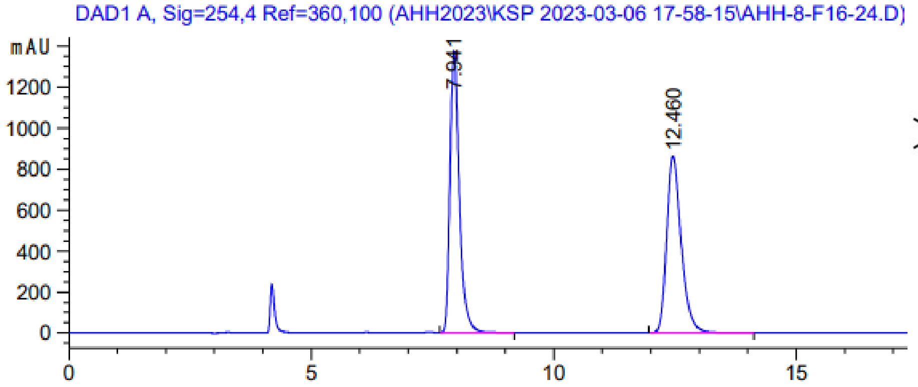

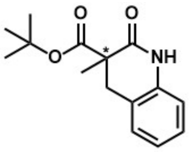

15aa

Peak #	RetTime [min]	Sig	Type	Area [mAU*s]	Height [mAU]	Area %
1	7.941	1	VV	1.80582e4	1375.76416	50.0815
2	12.460	1	VV	1.80015e4	861.18481	49.9241

Enantioenriched Compound **15aa**
HPLC (OD-H, isopropanol/n-hexanes = 10/90, flow rate = 1.0 mL/min, l = 254 nm) tR = 7.91 min (minor), 12.35 min (major).

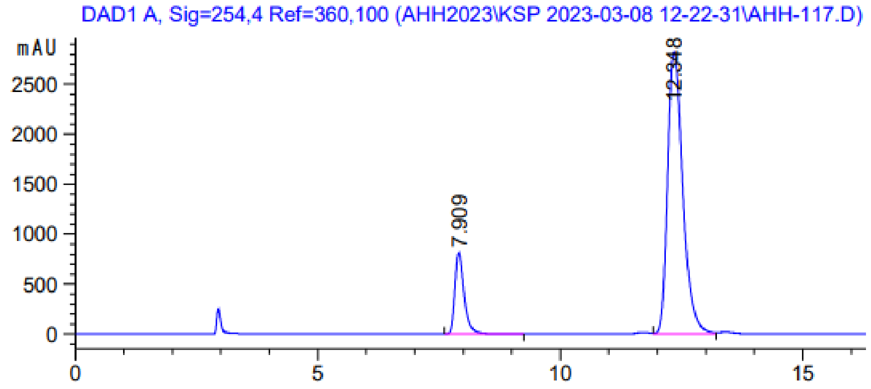

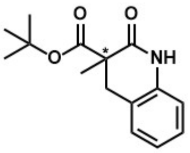

15aa

Peak #	RetTime [min]	Sig	Type	Area [mAU*s]	Height [mAU]	Area %
1	7.909	1	VV	1.06749e4	813.58063	14.7402
2	12.348	1	VV	6.17477e4	2823.57495	85.2626

Racemic Compound **15ab**

HPLC (OD-H, isopropanol/n-hexanes = 10/90, flow rate = 1.0 mL/min, l = 254 nm) tR = 7.94 min, 15.51 min.

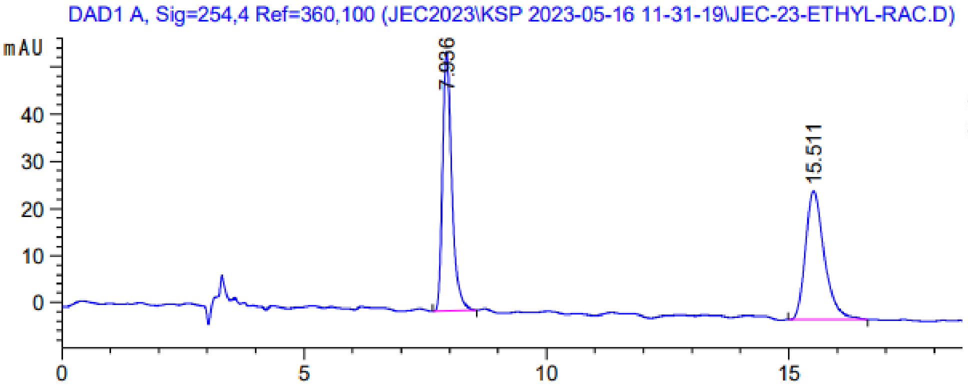

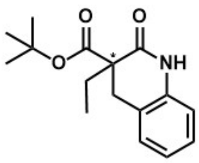

15ab

Peak #	RetTime [min]	Sig	Type	Area [mAU*s]	Height [mAU]	Area %
1	7.936	1	BB	720.94287	55.06948	49.1802
2	15.511	1	BB	746.97729	27.46064	50.9562

Enantioenriched compound **15ab**

HPLC (OD-H, isopropanol/n-hexanes = 10/90, flow rate = 1.0 mL/min, l = 254 nm) tR = 7.99 min (minor), 15.65 min (major).

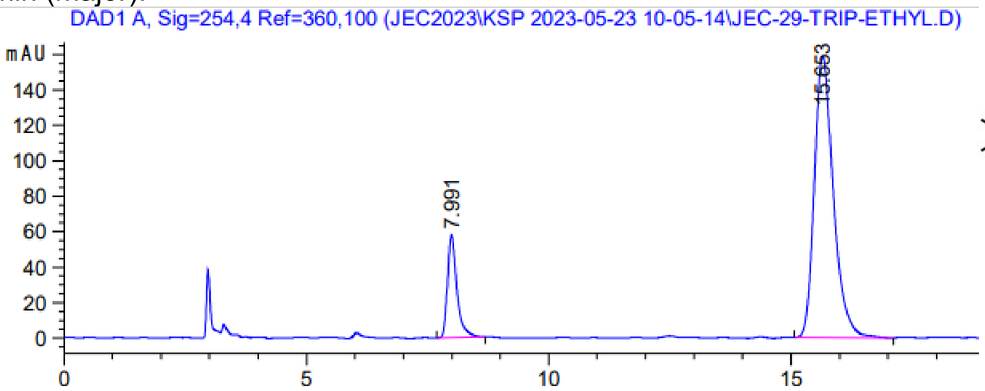

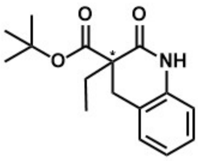

15ab

Peak #	RetTime [min]	Sig	Type	Area [mAU*s]	Height [mAU]	Area %
1	7.991	1	BB	773.96924	57.84583	15.2251
2	15.653	1	BB	4311.54980	158.63329	84.8143

Racemic compound **15ac**
HPLC (OD-H, isopropanol/n-hexanes = 10/90, flow rate = 1.0 mL/min, l = 254 nm) tR = 7.58 min, 15.20 min.

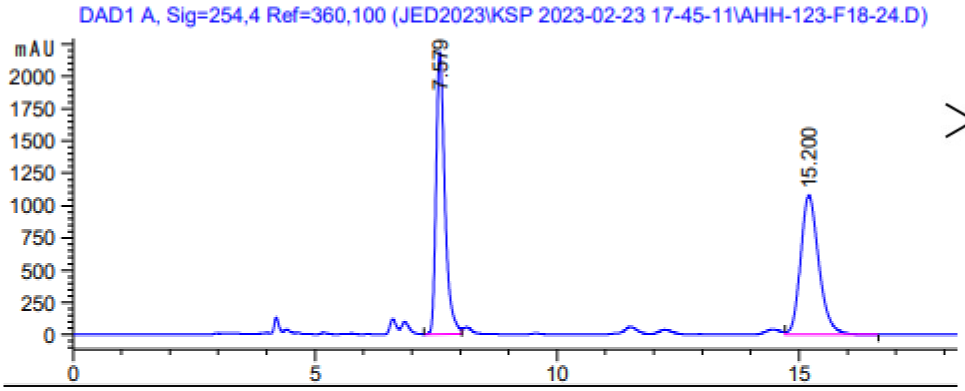

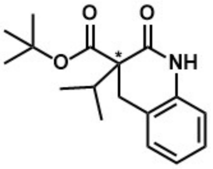

15ac

Peak #	RetTime [min]	Sig	Type	Area [mAU*s]	Height [mAU]	Area %
1	7.579	1	BV	2.78860e4	2184.30981	49.4721
2	15.200	1	VB	2.84831e4	1079.02991	50.5314

Enantioenriched compound **15ac**
HPLC (OD-H, isopropanol/n-hexanes = 10/90, flow rate = 1.0 mL/min, l = 254 nm) tR = 7.62 min (minor), 15.29 min (major).

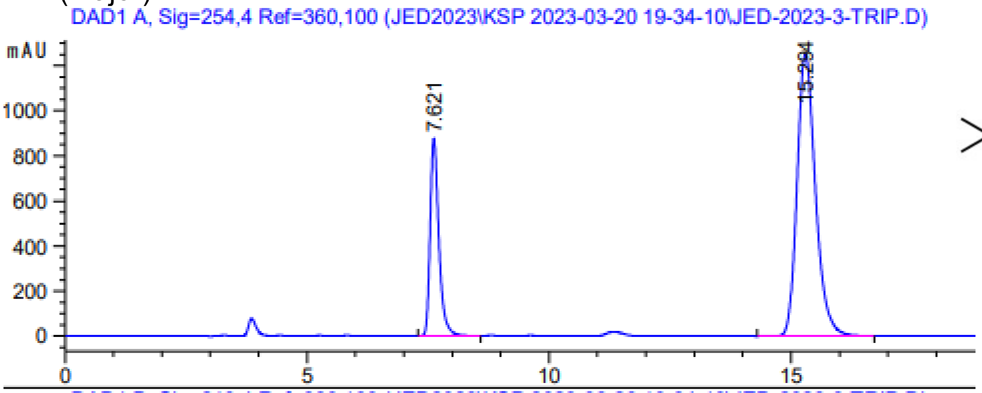

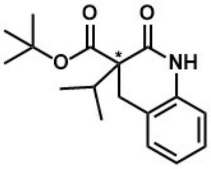

15ac

Peak #	RetTime [min]	Sig	Type	Area [mAU*s]	Height [mAU]	Area %
1	7.621	1	VV	1.11527e4	877.59088	25.1243
2	15.294	1	BB	3.32395e4	1254.00476	74.8802

Racemic compound **15ad**

HPLC (OD-H, isopropanol/n-hexanes = 10/90, flow rate = 1.0 mL/min, l = 254 nm) tR = 7.72 min, 21.76 min.

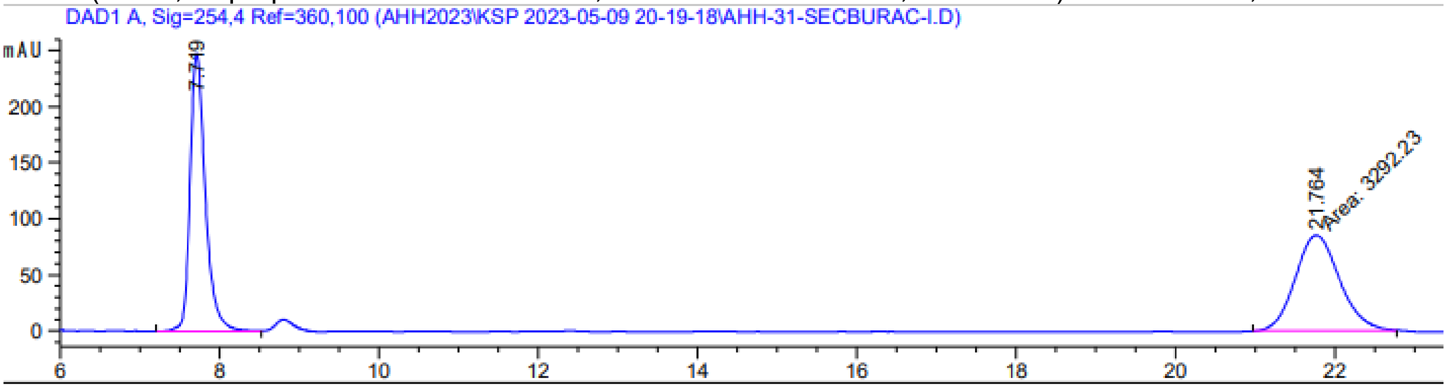

Peak #	RetTime [min]	Sig	Type	Area [mAU*s]	Height [mAU]	Area %
1	7.719	1	BV	3306.61670	248.15155	50.1242
2	21.764	1	MM	3292.23364	85.12388	49.9061

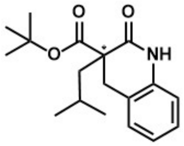

15ad

Enantioenriched compound **15ad**

HPLC (OD-H, isopropanol/n-hexanes = 10/90, flow rate = 1.0 mL/min, l = 254 nm) tR = 7.59 min (minor), 20.40 min (major).

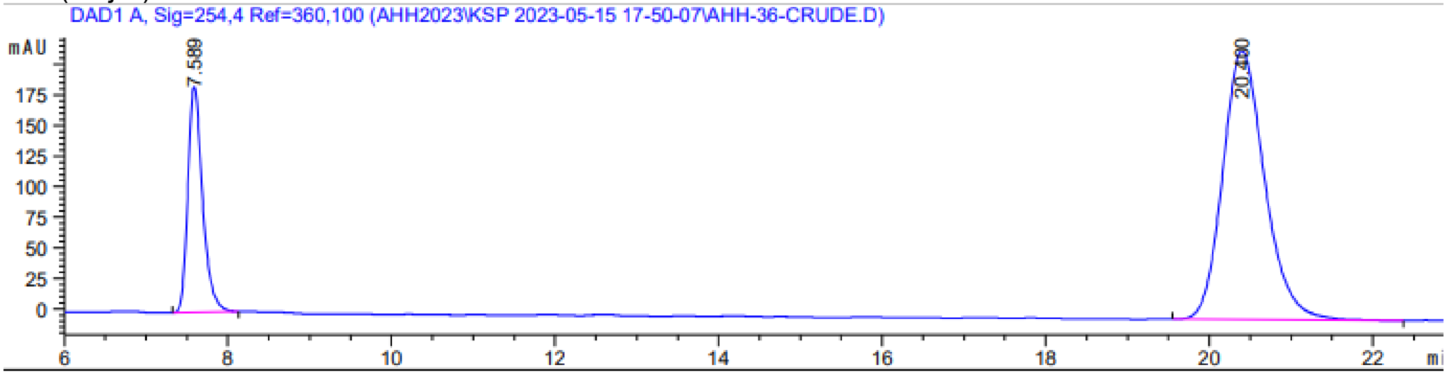

Peak #	RetTime [min]	Sig	Type	Area [mAU*s]	Height [mAU]	Area %
1	7.589	1	BB	2300.29443	184.78535	22.5878
2	20.400	1	BB	7885.51563	220.34418	77.4319

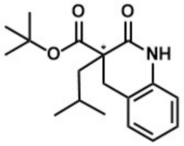

15ad

Racemic compound **15ae**

HPLC (OD-H, isopropanol/n-hexanes = 10/90, flow rate = 1.0 mL/min, l = 254 nm) tR = 17.37 min, 19.95 min.

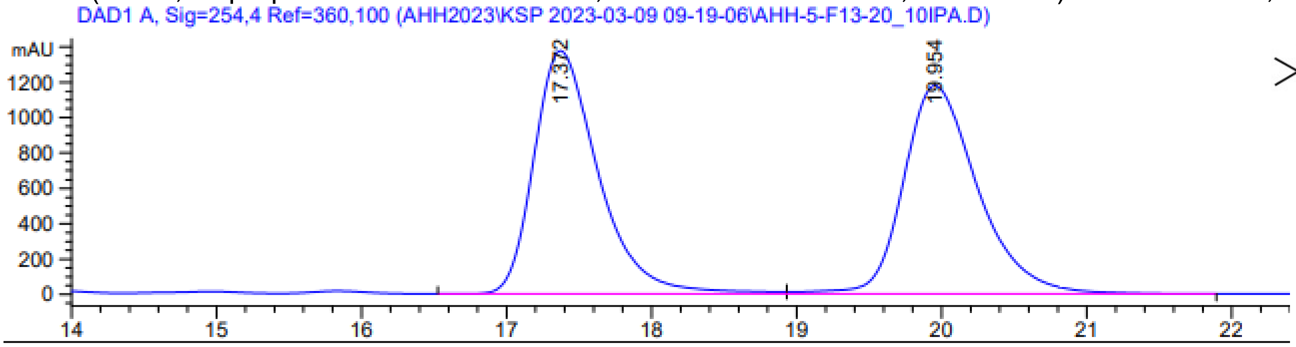

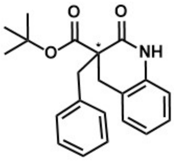

15ae

Peak #	RetTime [min]	Sig	Type	Area [mAU*s]	Height [mAU]	Area %
1	17.372	1	BV	4.25791e4	1375.83154	50.2741
2	19.954	1	VB	4.21169e4	1178.63574	49.7283

Enantioenriched compound **15ae**

HPLC (OD-H, isopropanol/n-hexanes = 10/90, flow rate = 1.0 mL/min, l = 254 nm) tR = 17.93 min (minor), 20.59 min (major).

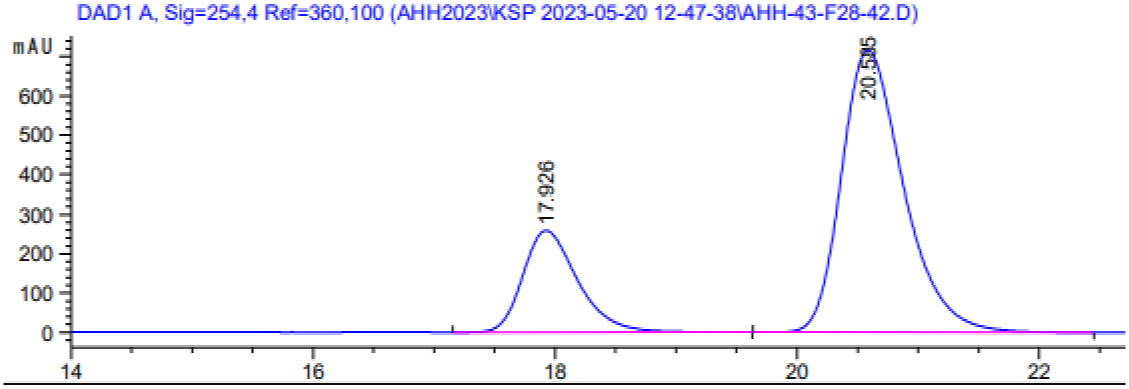

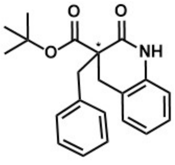

15ae

Peak #	RetTime [min]	Sig	Type	Area [mAU*s]	Height [mAU]	Area %
1	17.926	1	BB	8170.46777	259.40460	24.1515
2	20.585	1	BB	2.56615e4	717.52692	75.8544

Racemic compound **15af**

HPLC (OD-H, isopropanol/n-hexanes = 10/90, flow rate = 1.0 mL/min, l = 254 nm) tR = 11.50 min, 34.56 min.

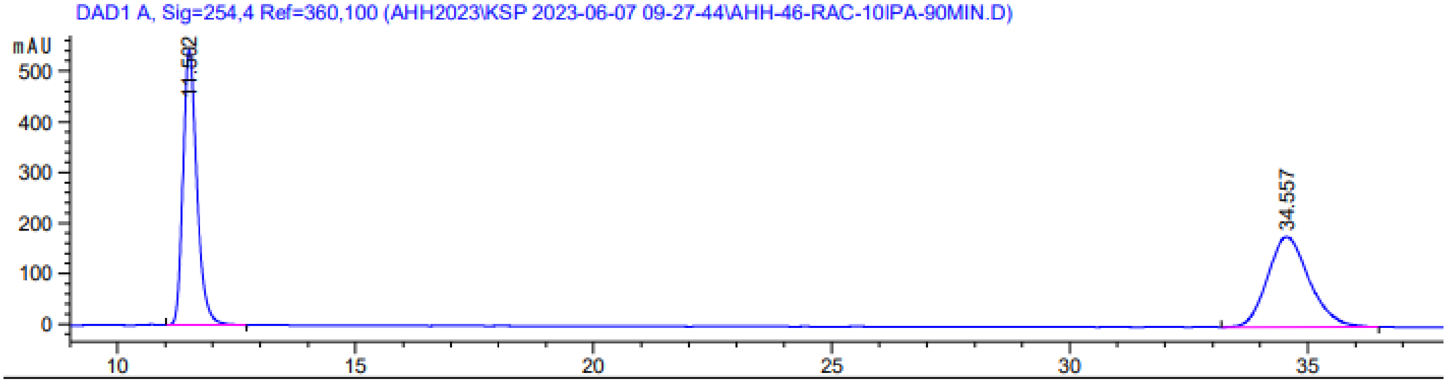

Peak #	RetTime [min]	Sig	Type	Area [mAU*s]	Height [mAU]	Area %
1	11.502	1	BB	1.09091e4	545.06958	50.1748
2	34.557	1	BB	1.08351e4	177.68890	49.8344

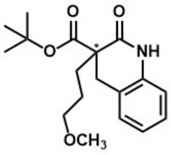

15af

Enantioenriched compound **15af**

HPLC (OD-H, isopropanol/n-hexanes = 10/90, flow rate = 1.0 mL/min, l = 254 nm) tR = 11.56 min (minor), 34.95 min (major).

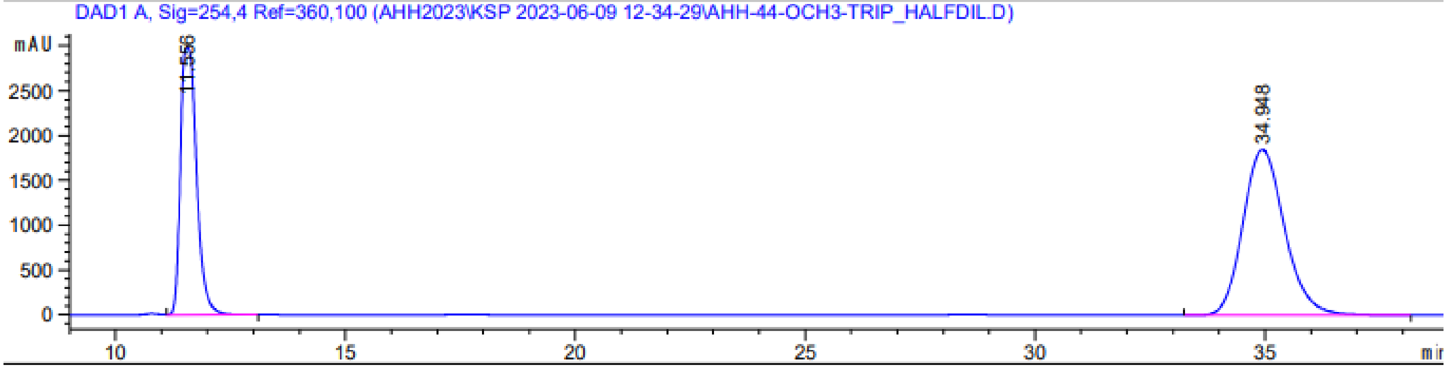

Peak #	RetTime [min]	Sig	Type	Area [mAU*s]	Height [mAU]	Area %
1	11.556	1	VB	7.25658e4	2985.05078	38.2911
2	34.948	1	BB	1.16947e5	1849.00061	61.7099

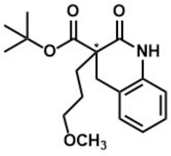

15af

Racemic compound **15ba**

HPLC (OD-H, isopropanol/n-hexanes = 6/94, flow rate = 1.0 mL/min, l = 254 nm) tR = 7.50 min, 8.74 min.

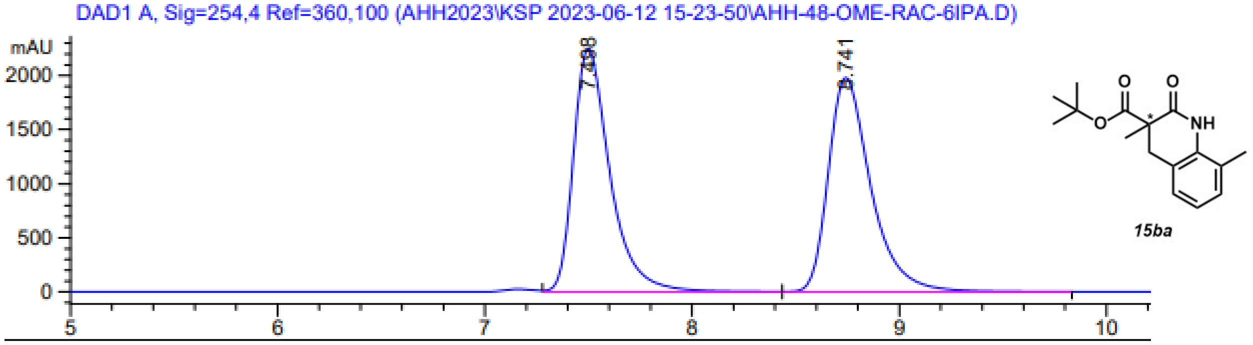

Peak #	RetTime [min]	Sig	Type	Area [mAU*s]	Height [mAU]	Area %
1	7.498	1	VB	2.76281e4	2252.55859	49.4635
2	8.741	1	BB	2.82295e4	1979.48730	50.5401

Enantioenriched compound **15ba**

HPLC (OD-H, isopropanol/n-hexanes = 6/94, flow rate = 1.0 mL/min, l = 254 nm) tR = 7.50 min (minor), 8.74 min (major).

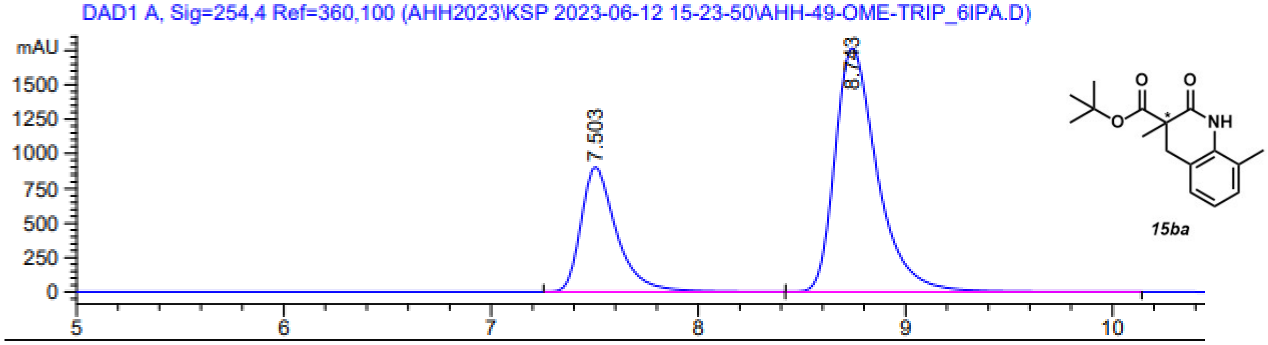

Peak #	RetTime [min]	Sig	Type	Area [mAU*s]	Height [mAU]	Area %
1	7.503	1	VB	1.08364e4	899.96790	30.1316
2	8.743	1	BB	2.51292e4	1766.80725	69.8739

Racemic compound **15cb**

HPLC (OD-H, isopropanol/n-hexanes = 10/90, flow rate = 1.0 mL/min, l = 254 nm) tR = 6.76 min, 14.36 min.

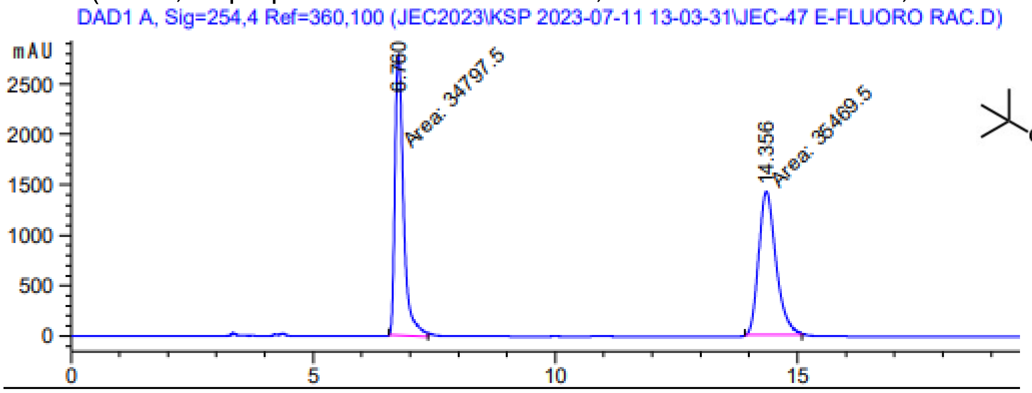

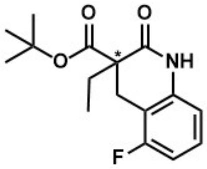

15cb

Peak #	RetTime [min]	Sig	Type	Area [mAU*s]	Height [mAU]	Area %
1	6.760	1	MM	3.47975e4	2788.28784	49.5233
2	14.356	1	MM	3.54695e4	1423.15857	50.4796

Enantioenriched compound **15cb**

HPLC (OD-H, isopropanol/n-hexanes = 10/90, flow rate = 1.0 mL/min, l = 254 nm) tR = 6.74 min (minor), 13.97 min (major).

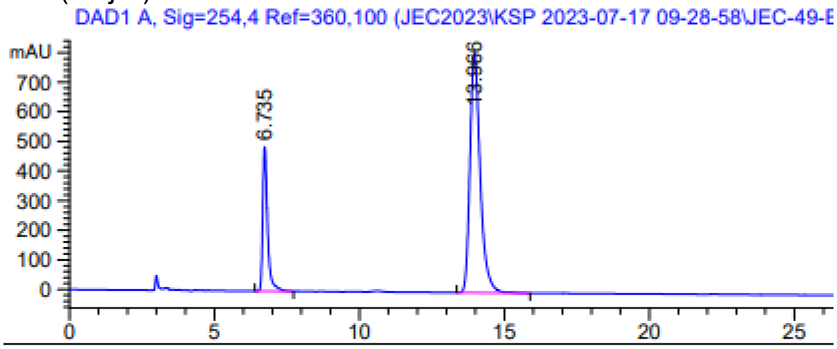

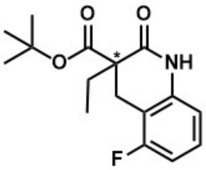

15cb

Peak #	RetTime [min]	Sig	Type	Area [mAU*s]	Height [mAU]	Area %
1	6.735	1	BB	5637.40723	487.39581	22.0327
2	13.966	1	BB	1.99511e4	811.17310	77.9751

Racemic compound **15da**

HPLC (OD-H, isopropanol/n-hexanes = 10/90, flow rate = 1.0 mL/min, l = 254 nm) tR = 6.26 min, 8.41 min.

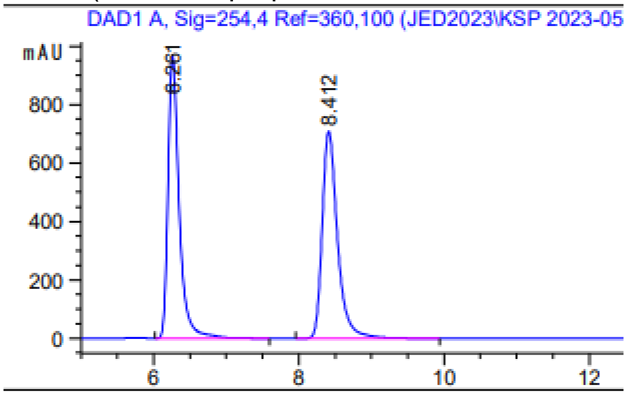

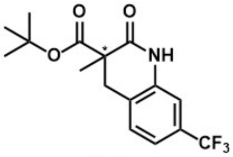

15da

Peak #	RetTime [min]	Sig	Type	Area [mAU*s]	Height [mAU]	Area %
1	6.261	1	VB	1.03344e4	967.61176	49.9120
2	8.412	1	BB	1.03728e4	707.95837	50.0976

Enantioenriched compound **15da**

HPLC (OD-H, isopropanol/n-hexanes = 10/90, flow rate = 1.0 mL/min, l = 254 nm) tR = 6.28 min (minor), 8.55 min (major).

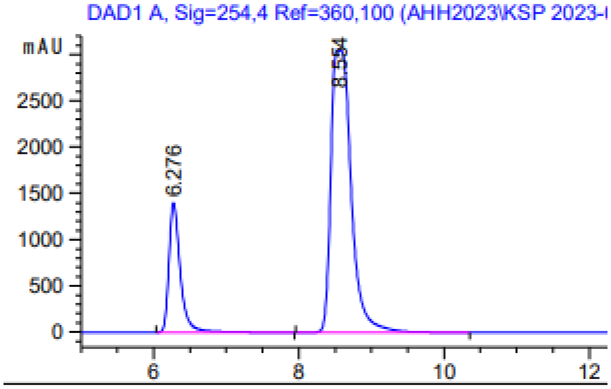

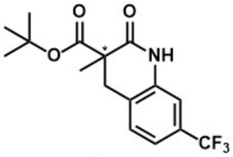

15da

Peak #	RetTime [min]	Sig	Type	Area [mAU*s]	Height [mAU]	Area %
1	6.276	1	VB	1.51740e4	1398.55640	20.4014
2	8.554	1	BB	5.92054e4	3057.07617	79.6013

Racemic compound **15ea**

HPLC (OD-H, isopropanol/n-hexanes = 6/94, flow rate = 1.0 mL/min, l = 254 nm) tR = 10.83 min, 19.75 min.

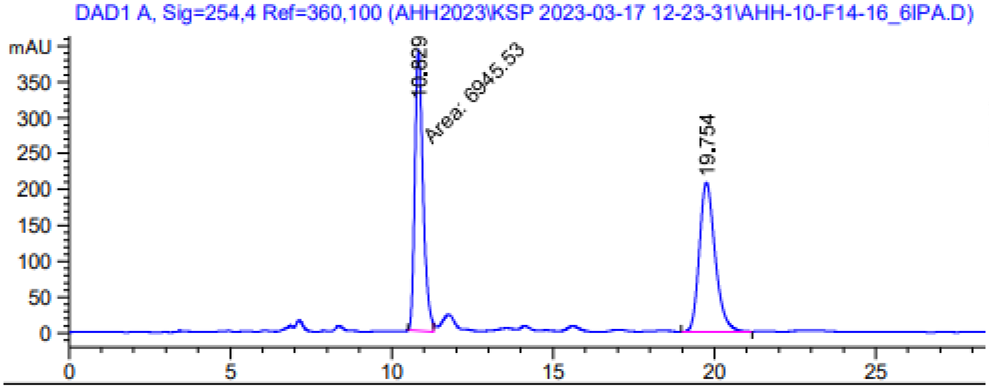

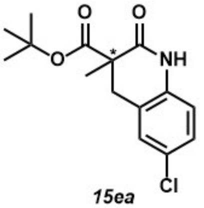

15ea

Peak #	RetTime [min]	Sig	Type	Area [mAU*s]	Height [mAU]	Area %
1	10.829	1	MM	6945.52979	391.81067	49.6683
2	19.754	1	BB	7040.28809	207.16495	50.3460

Enantioenriched compound **15ea**

HPLC (OD-H, isopropanol/n-hexanes = 6/94, flow rate = 1.0 mL/min, l = 254 nm) tR = 10.81 min (minor), 19.59 min (major).

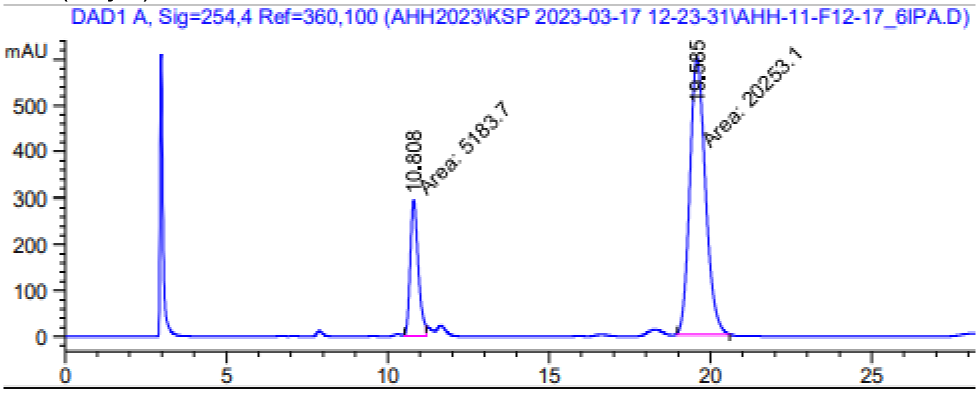

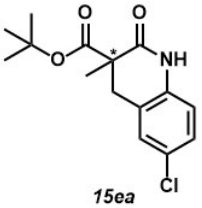

15ea

Peak #	RetTime [min]	Sig	Type	Area [mAU*s]	Height [mAU]	Area %
1	10.808	1	MM	5183.70410	295.62332	20.3804
2	19.585	1	MM	2.02531e4	599.63745	79.6275

Racemic compound **15eb**

HPLC (OD-H, isopropanol/n-hexanes = 10/90, flow rate = 1.0 mL/min, l = 254 nm) tR = 7.29 min, 16.44 min.

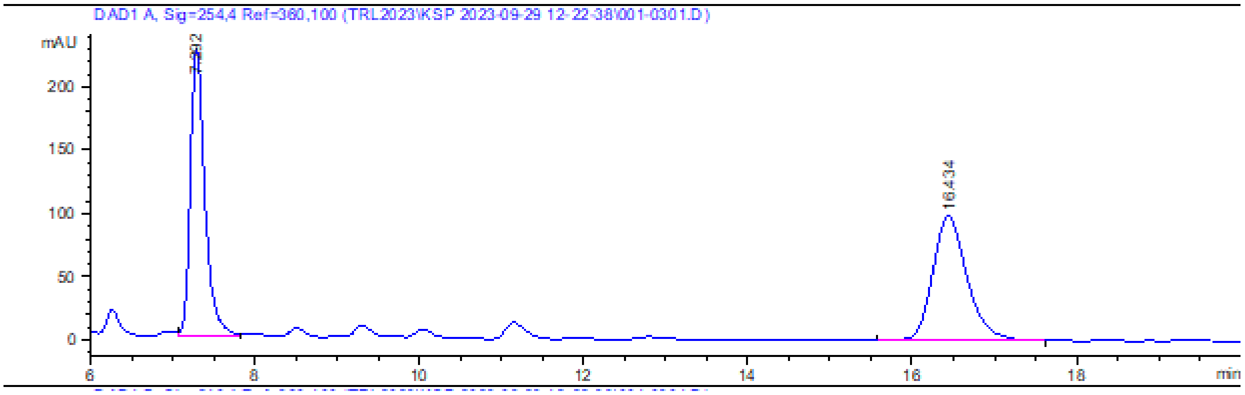

Peak #	RetTime [min]	Type	Width [min]	Area [mAU*s]	Height [mAU]	Area %
1	7.291	BV	0.1984	4949.88428	369.23288	51.1810
2	16.437	BB	0.4451	4721.44238	160.56915	48.8190

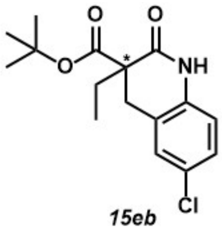

15eb

Enantioenriched compound **15eb**

HPLC (OD-H, isopropanol/n-hexanes = 10/90, flow rate = 1.0 mL/min, l = 254 nm) tR = 7.13 min (minor), 15.78 min (major).

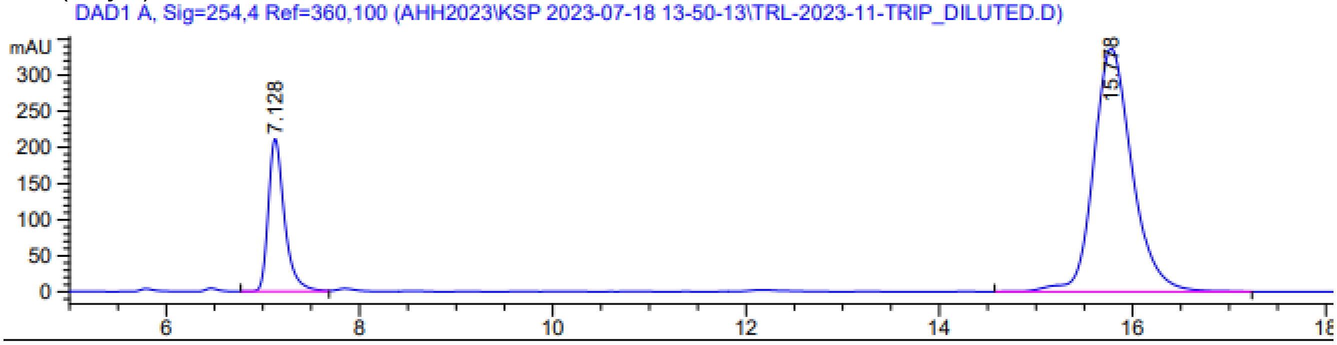

Peak #	RetTime [min]	Sig	Type	Area [mAU*s]	Height [mAU]	Area %
1	7.128	1	BV	2477.96118	211.07867	20.9981
2	15.778	1	BB	9324.94043	336.89780	79.0189

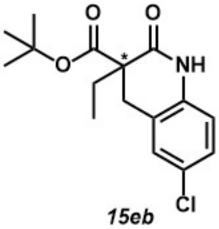

15eb

Racemic compound **15fa**

HPLC (OD-H, isopropanol/n-hexanes = 10/90, flow rate = 1.0 mL/min, l = 254 nm) tR = 10.71 min, 17.27 min.

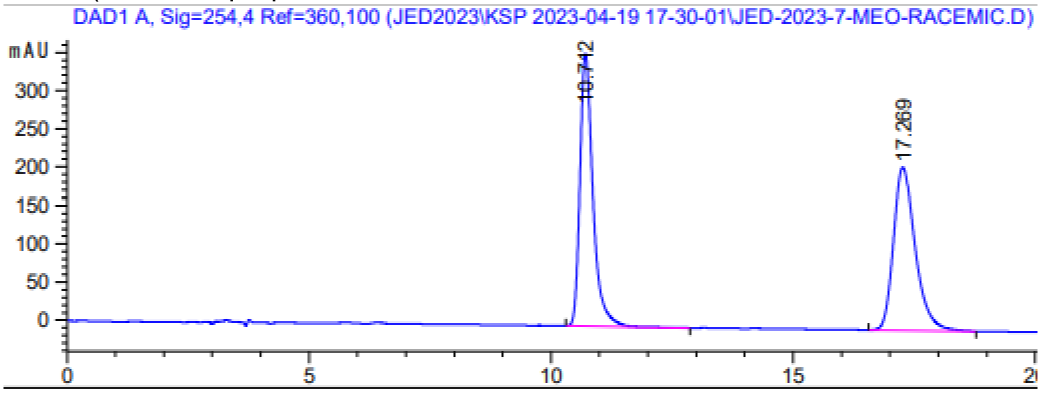

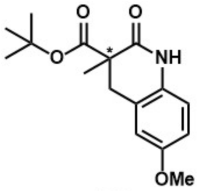

15fa

Peak #	RetTime [min]	Sig	Type	Area [mAU*s]	Height [mAU]	Area %
1	10.712	1	BB	6689.73096	356.32520	50.1700
2	17.269	1	BB	6646.38867	212.77480	49.8450

Enantioenriched compound **15fa**

HPLC (OD-H, isopropanol/n-hexanes = 10/90, flow rate = 1.0 mL/min, l = 254 nm) tR = 10.66 min (minor), 17.15 min (major).

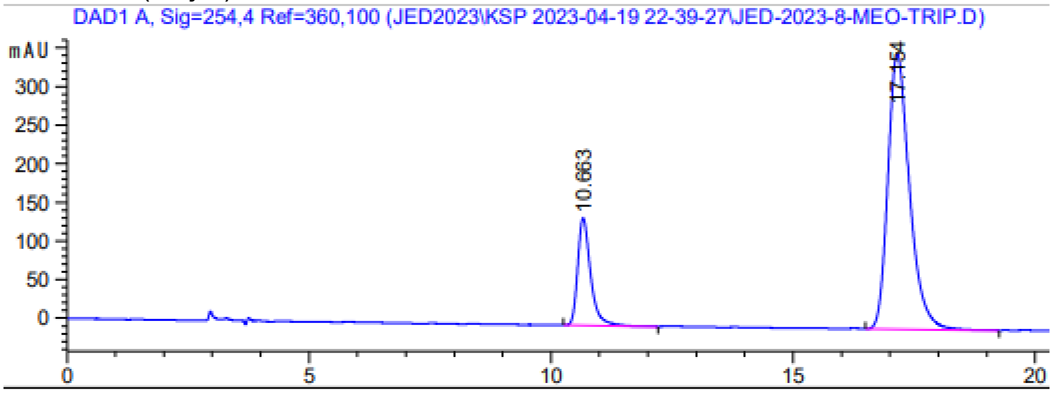

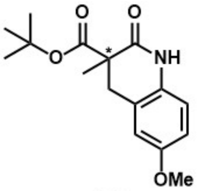

15fa

Peak #	RetTime [min]	Sig	Type	Area [mAU*s]	Height [mAU]	Area %
1	10.663	1	BB	2611.17456	139.56532	19.0266
2	17.154	1	BB	1.11146e4	356.75491	80.9879

Racemic compound **22aa**

HPLC (OD-H, isopropanol/n-hexanes = 1/99, flow rate = 1.0 mL/min, l = 254 nm) tR = 13.53 min, 14.52 min.

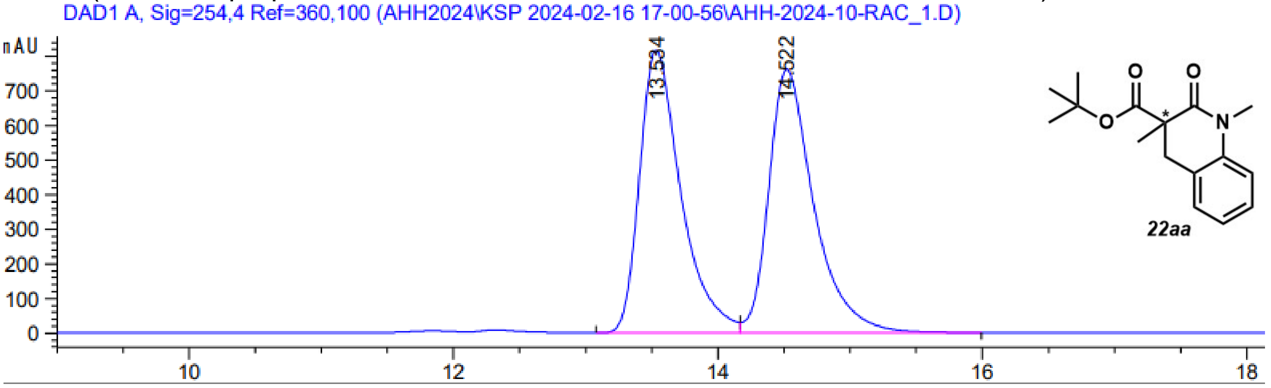

Peak #	RetTime [min]	Sig	Type	Area [mAU*s]	Height [mAU]	Area %
1	13.534	1	BV	1.77818e4	817.02588	49.6654
2	14.522	1	VB	1.80235e4	761.55005	50.3402

Enantioenriched compound **22aa**

HPLC (OD-H, isopropanol/n-hexanes = 1/99, flow rate = 1.0 mL/min, l = 254 nm) tR = 13.71 min (major), 14.71 min (minor).

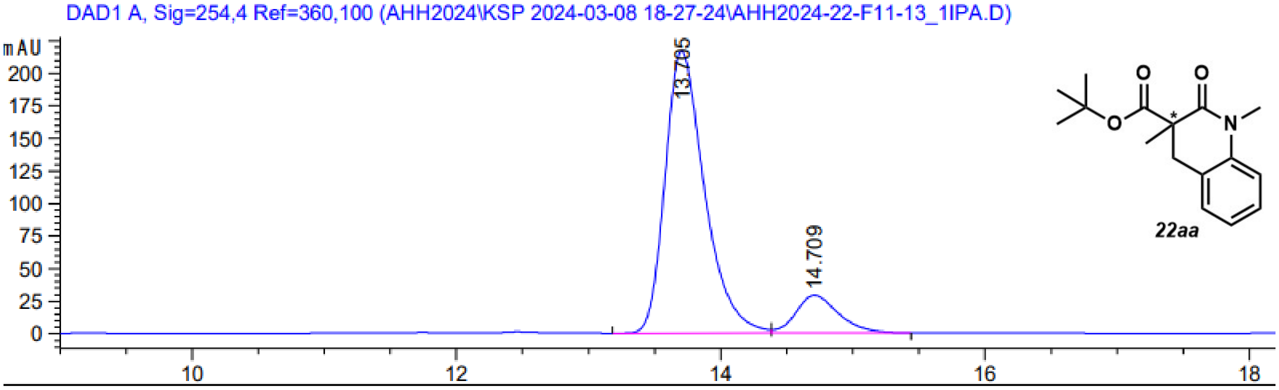

Peak #	RetTime [min]	Sig	Type	Area [mAU*s]	Height [mAU]	Area %
1	13.705	1	BV	4505.33789	216.96422	87.3807
2	14.709	1	VB	652.64880	29.01754	12.6581

Racemic compound **23aa**

HPLC (OD-H, isopropanol/n-hexanes = 10/90, flow rate = 1.0 mL/min, l = 254 nm) tR = 9.19 min, 17.07 min.

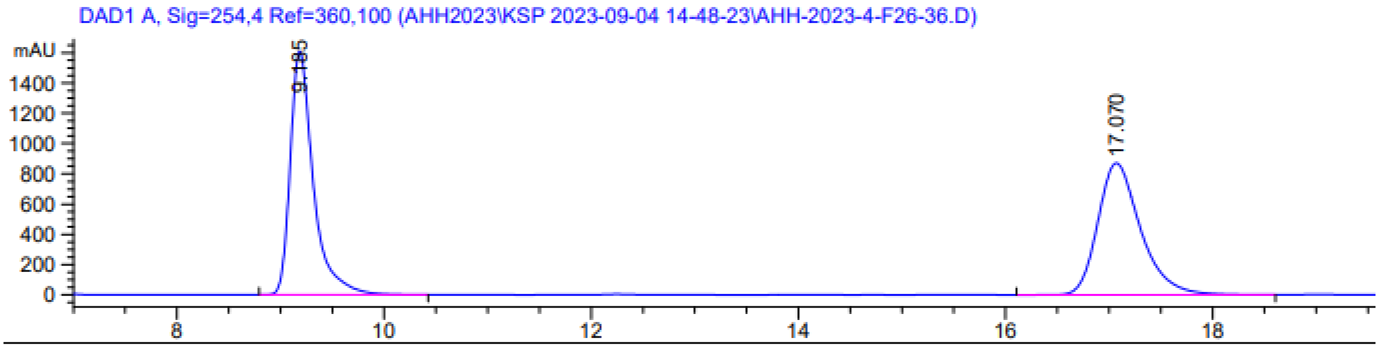

Peak #	RetTime [min]	Sig	Type	Area [mAU*s]	Height [mAU]	Area %
1	9.185	1	BB	2.49536e4	1609.22180	49.9633
2	17.070	1	BB	2.49923e4	869.62250	50.0407

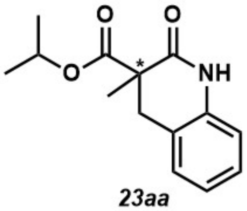

23aa

Enantioenriched compound **26**

HPLC (OD-H, isopropanol/n-hexanes = 10/90, flow rate = 1.0 mL/min, l = 254 nm) tR = 12.15 min (minor), 15.01 min (major).

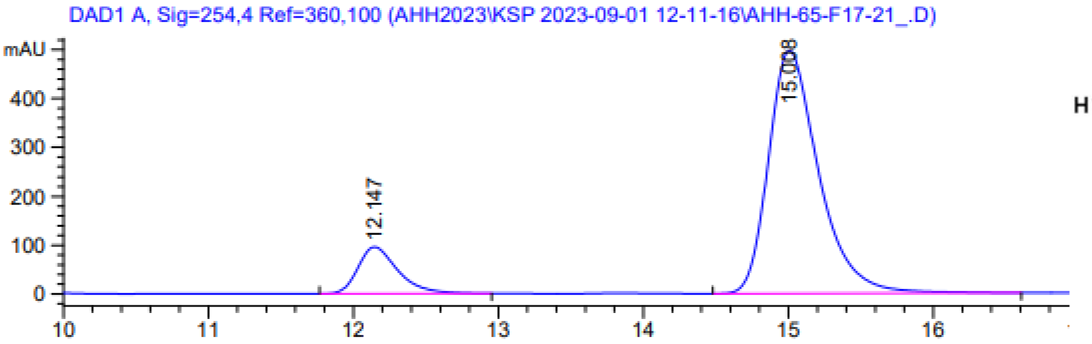

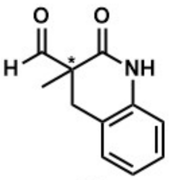

26

Peak #	RetTime [min]	Sig	Type	Area [mAU*s]	Height [mAU]	Area %
1	12.147	1	BB	1828.90674	95.77036	13.2438
2	15.008	1	BB	1.19826e4	497.58649	86.7707

Enantioenriched compound **27**
HPLC (OD-H, isopropanol/n-hexanes = 10/90, flow rate = 1.0 mL/min, l = 254 nm) tR = 12.05 min (minor), 16.76 min (major).

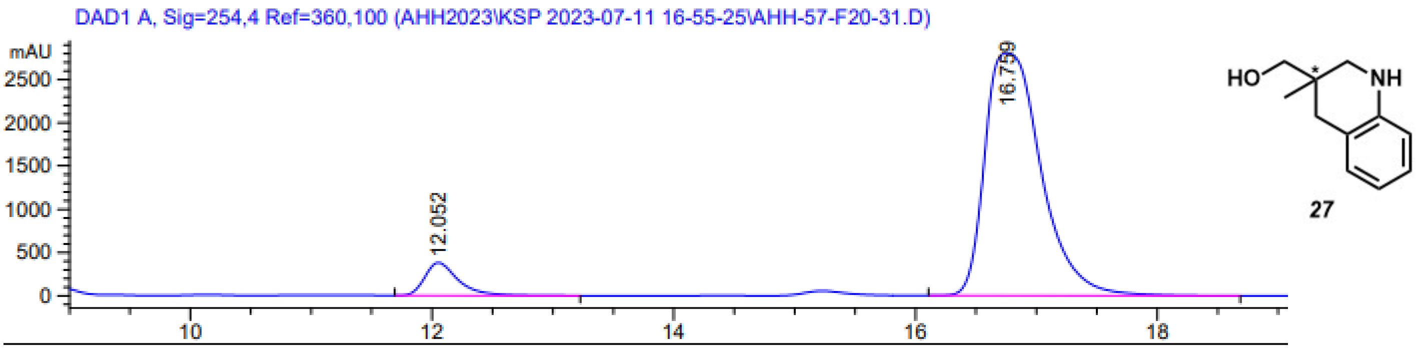

Peak #	RetTime [min]	Sig	Type	Area [mAU*s]	Height [mAU]	Area %
1	12.052	1	BB	7369.56885	381.43591	7.5228
2	16.759	1	BB	9.05952e4	2807.84131	92.4792

Enantioenriched compound **28**
HPLC (OD-H, isopropanol/n-hexanes = 10/90, flow rate = 1.0 mL/min, l = 254 nm) tR = 16.03 min (minor), 18.74 min (major).

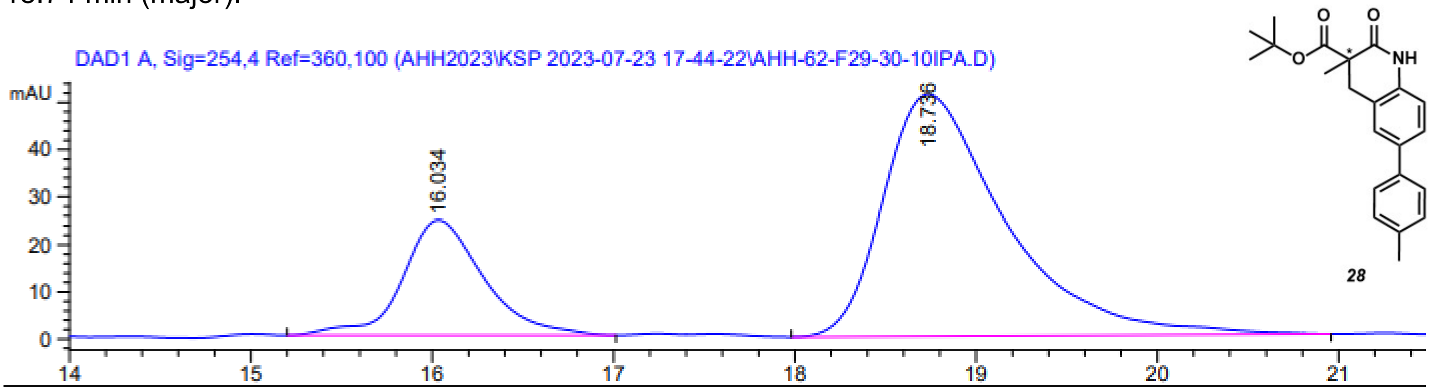

Peak #	RetTime [min]	Sig	Type	Area [mAU*s]	Height [mAU]	Area %
1	16.034	1	BB	759.76532	24.38554	24.0577
2	18.736	1	BB	2400.33081	51.14306	76.0056

DFT Figure and Coordinates

**Computational details:**	
Software package: Gaussian, version 16C.01	Solvent model: CPCM
DFT functional: M06-2X	Solvent: Toluene
Basis set: 6-311+G(d) for N, O, and P elements	Barrier height: ΔG≠ (kcal/mol)
Basis set: 6-31G(d) for C and H elements	

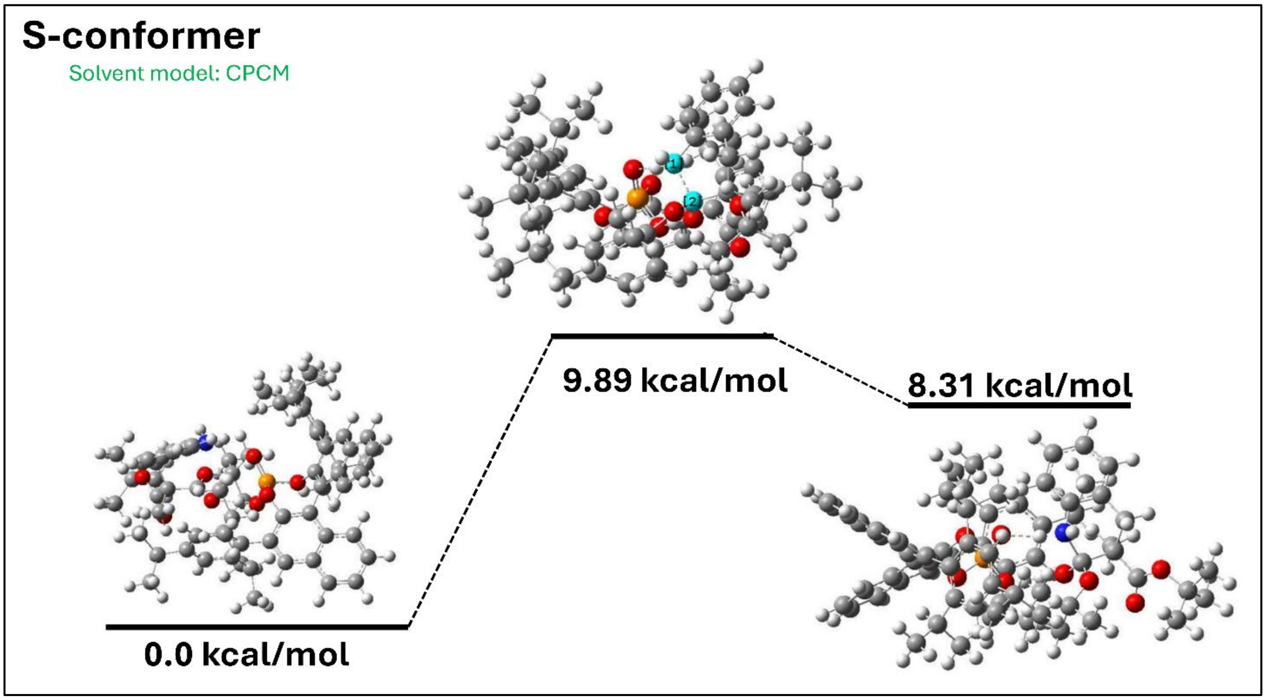

DFT calculations reveal transition state of C-N bond forming step through bi-dentate binding with CPA catalyst TRIP for major S-conformer.

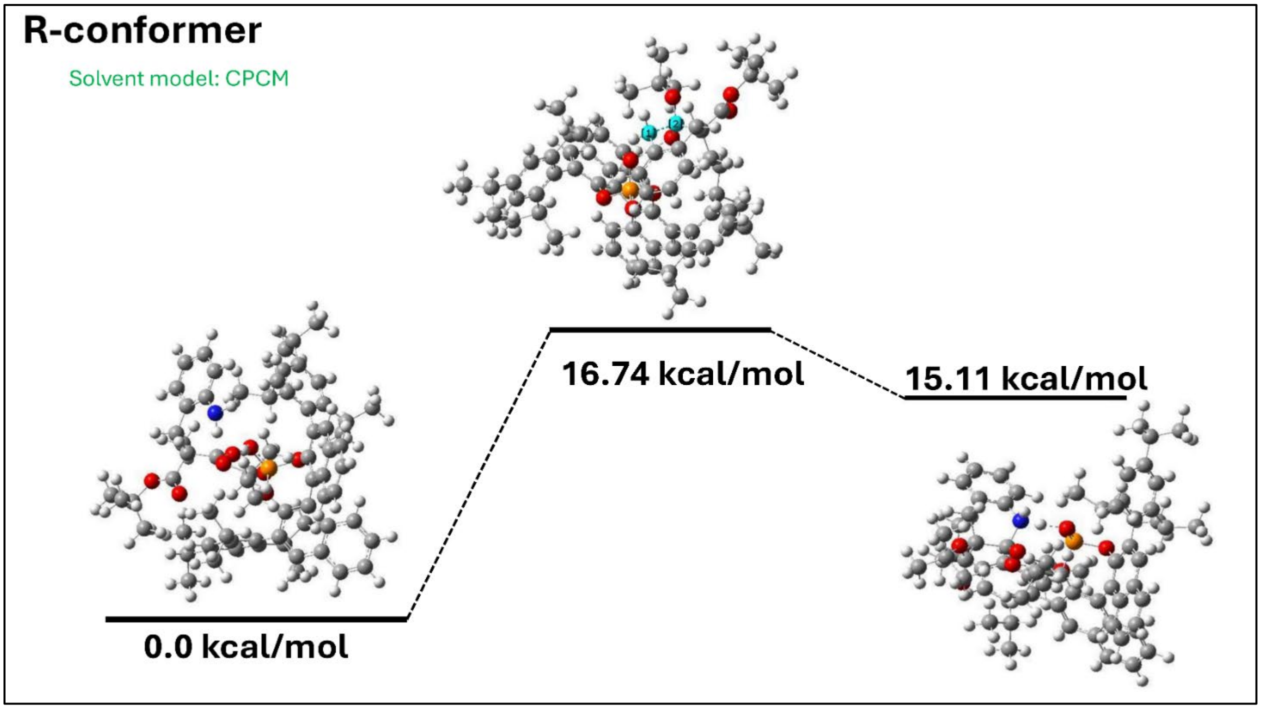

DFT calculations reveal transition state of C-N bond forming step through mono-dentate binding with CPA catalyst TRIP for minor R-conformer.

Cartesian Coordinates

1. Cartesian coordinates of the S-conformer reactant structure

C	-7.32508	-2.11521	-0.92612
C	-5.96884	-2.15707	-0.73303
C	-5.22278	-0.96286	-0.54826
C	-5.88898	0.29413	-0.60118
C	-7.30035	0.30229	-0.77170
C	-7.99679	-0.86984	-0.92929
H	-3.34350	-1.98267	-0.22621
H	-7.88839	-3.03300	-1.05949
H	-5.44104	-3.10627	-0.70097
C	-3.83327	-1.01381	-0.26357
C	-5.11538	1.49456	-0.45086
H	-7.83025	1.24829	-0.76644
H	-9.07475	-0.84177	-1.05168
C	-3.77899	1.36373	-0.13479
C	-3.10651	0.11959	0.00166
C	-5.72263	2.85072	-0.55717
C	-6.47279	3.28123	-1.70199
C	-5.54394	3.75487	0.46914
C	-7.10000	4.55931	-1.67165
C	-6.58958	2.49806	-2.88241
C	-6.12364	5.04962	0.50980
C	-7.86660	4.98561	-2.78890
C	-6.92495	5.40648	-0.54639
C	-7.32311	2.94524	-3.95260
H	-6.08131	1.54139	-2.93543
C	-7.98236	4.19665	-3.90353
H	-8.34775	5.95876	-2.74488
H	-7.41346	6.37748	-0.54170
H	-7.39396	2.33605	-4.84790
H	-8.56470	4.53348	-4.75491
O	-4.77348	3.38521	1.56171
O	-3.00901	2.50724	0.02897
P	-3.17819	3.37741	1.36869
O	-2.79001	2.47355	2.58147
H	-1.94697	2.66824	3.07345
O	-2.51986	4.68255	1.21794
C	-5.84736	5.97820	1.64857
C	-6.34488	5.69189	2.93707
C	-5.10278	7.15391	1.42548
C	-6.14254	6.61750	3.96016
C	-4.92851	8.04968	2.48566
C	-5.44959	7.81087	3.75374
H	-6.54990	6.40427	4.94715
H	-4.37344	8.96980	2.31456
C	-1.67749	0.06807	0.43928
C	-0.65633	0.48898	-0.43053
C	-1.36020	-0.39680	1.73501
C	0.67480	0.39665	-0.01316
C	-0.01682	-0.47582	2.10169
C	1.01666	-0.09561	1.24426
H	1.45946	0.71355	-0.69561
H	0.24285	-0.83984	3.09400
C	-2.42619	-0.82016	2.73760
H	-3.37428	-0.36310	2.43477
C	2.45661	-0.29603	1.68143

H	2.48348	-0.16142	2.77112
C	-0.94923	0.99849	-1.83310
H	-2.03297	1.09008	-1.95151
C	-4.48306	7.49690	0.07738
H	-4.55905	6.61739	-0.56909
C	-7.12209	4.42389	3.25668
H	-7.16701	3.80182	2.35827
C	-5.29705	8.82761	4.86961
H	-4.70371	9.66167	4.47420
C	1.79167	4.69405	-0.15855
C	3.00662	4.39600	0.45371
C	3.11052	4.49340	1.84002
C	2.02840	4.88772	2.62664
C	0.82171	5.23890	1.99369
C	0.70832	5.12114	0.60337
H	1.68810	4.61583	-1.23660
H	3.86018	4.07841	-0.13652
H	4.04724	4.23620	2.33028
H	-0.23862	5.37335	0.13350
N	-0.26619	5.63845	2.78299
H	-0.08256	6.42017	3.40394
H	-1.13018	5.75366	2.25980
C	2.13080	4.87244	4.13260
H	3.18699	4.87761	4.41625
H	1.68562	5.76261	4.58925
C	1.50231	3.60799	4.78515
C	1.81125	3.51794	6.29036
C	-0.03210	3.63326	4.71835
C	2.00458	2.32317	4.11707
H	1.65862	2.28125	3.08167
H	3.09996	2.30187	4.12356
H	1.62471	1.44683	4.64765
O	2.51896	4.54356	6.74210
O	-0.53866	4.48386	5.58917
O	-0.66164	2.88626	4.00073
O	1.42669	2.58879	6.95857
C	2.85734	4.68303	8.16130
C	-1.97621	4.59153	5.88515
C	-2.57077	3.21597	6.18212
H	-1.90104	2.64354	6.83258
H	-3.52097	3.35834	6.70613
H	-2.76695	2.64065	5.27607
C	-2.69425	5.29423	4.74150
H	-3.75602	5.39541	4.99265
H	-2.28385	6.29606	4.57971
H	-2.61252	4.73778	3.80757
C	-1.98364	5.45314	7.14169
H	-1.46323	6.39813	6.95848
H	-3.01548	5.67220	7.43121
H	-1.48915	4.93102	7.96638
C	3.72617	3.51370	8.61439
H	4.10767	3.72577	9.61783
H	3.16324	2.58038	8.64309
H	4.58164	3.39825	7.94152
C	1.57591	4.81434	8.97801
H	0.99808	3.88854	8.96852
H	1.83593	5.05474	10.01335
H	0.96055	5.62731	8.57946

C	3.65026	5.98353	8.18969
H	3.95635	6.20796	9.21520
H	4.54574	5.89833	7.56744
H	3.04000	6.81076	7.81635
C	-8.56667	4.75068	3.65576
H	-8.59596	5.35413	4.56961
H	-9.07521	5.31096	2.86497
H	-9.12915	3.83021	3.84278
C	-6.42005	3.60628	4.34813
H	-6.38692	4.15789	5.29503
H	-6.95877	2.66966	4.52686
H	-5.39447	3.36404	4.05194
C	-5.23865	8.65189	-0.59322
H	-4.80573	8.87565	-1.57369
H	-6.29846	8.41721	-0.73169
H	-5.17525	9.55853	0.01934
C	-2.99270	7.83806	0.19876
H	-2.56794	8.00139	-0.79745
H	-2.83372	8.75398	0.77792
H	-2.45073	7.01765	0.67594
C	-6.66400	9.37880	5.29372
H	-6.54723	10.15418	6.05781
H	-7.19662	9.80998	4.44102
H	-7.28854	8.58272	5.71418
C	-4.55112	8.24887	6.07675
H	-4.45386	9.00206	6.86553
H	-5.09126	7.39208	6.49669
H	-3.54866	7.91147	5.79788
C	-2.59978	-2.34595	2.74340
H	-1.65676	-2.82968	3.02288
H	-3.36198	-2.64173	3.47213
H	-2.89645	-2.73145	1.76477
C	-2.12154	-0.34656	4.16479
H	-1.84677	0.71042	4.19319
H	-3.00246	-0.49418	4.79822
H	-1.30113	-0.91914	4.61119
C	2.89114	-1.73756	1.37887
H	2.85919	-1.92104	0.29890
H	3.91408	-1.91608	1.72674
H	2.22885	-2.46073	1.86442
C	3.43642	0.69846	1.05871
H	4.42357	0.58980	1.51963
H	3.55660	0.51801	-0.01554
H	3.10317	1.73225	1.19282
C	-0.45758	-0.00691	-2.88230
H	0.62886	-0.13428	-2.81982
H	-0.92171	-0.98717	-2.73360
H	-0.70013	0.34061	-3.89192
C	-0.34491	2.38552	-2.07254
H	-0.69502	3.09661	-1.31761
H	0.74940	2.35464	-2.03116
H	-0.63135	2.75694	-3.06224

2. Cartesian coordinates of the S-conformer transition state structure

C	-7.33691	-2.15589	-0.81255
C	-5.98028	-2.20431	-0.62175
C	-5.22854	-1.01465	-0.43200

C	-5.89172	0.24457	-0.47504
C	-7.30233	0.26031	-0.64804
C	-8.00393	-0.90808	-0.81107
H	-3.34433	-2.04148	-0.15939
H	-7.90383	-3.07094	-0.94949
H	-5.45669	-3.15606	-0.59681
C	-3.83406	-1.07186	-0.16787
C	-5.11621	1.44166	-0.31361
H	-7.82610	1.21009	-0.64018
H	-9.08164	-0.87532	-0.93459
C	-3.78125	1.30366	0.00260
C	-3.10365	0.05823	0.09914
C	-5.70377	2.80509	-0.43545
C	-6.41816	3.23762	-1.60282
C	-5.51814	3.71970	0.58066
C	-7.00455	4.53522	-1.60884
C	-6.53861	2.43709	-2.77147
C	-6.05065	5.03528	0.57961
C	-7.73852	4.96368	-2.74674
C	-6.81944	5.39905	-0.49766
C	-7.23989	2.88607	-3.86245
H	-6.05953	1.46458	-2.79909
C	-7.86083	4.15758	-3.84844
H	-8.18917	5.95223	-2.72906
H	-7.28099	6.38277	-0.51677
H	-7.31436	2.26213	-4.74728
H	-8.41839	4.49571	-4.71577
O	-4.79801	3.34401	1.70158
O	-3.01541	2.44142	0.20075
P	-3.19344	3.25661	1.57889
O	-2.82519	2.37768	2.77943
H	-1.92097	2.67275	3.36849
O	-2.49508	4.55884	1.44831
C	-5.77809	5.97926	1.70735
C	-6.40105	5.78926	2.95657
C	-4.92205	7.07965	1.50409
C	-6.22280	6.74943	3.95265
C	-4.78322	8.01873	2.53069
C	-5.43744	7.88486	3.75230
H	-6.72049	6.60975	4.91096
H	-4.14675	8.88688	2.37288
C	-1.65324	0.02858	0.46247
C	-0.69510	0.47789	-0.46456
C	-1.25192	-0.41195	1.74186
C	0.65683	0.44754	-0.11403
C	0.11105	-0.42958	2.04120
C	1.08134	-0.00718	1.13240
H	1.39310	0.78593	-0.83913
H	0.43436	-0.76786	3.02347
C	-2.25055	-0.87141	2.79533
H	-3.22009	-0.42261	2.55429
C	2.55029	-0.10506	1.50063
H	2.61253	-0.02887	2.59444
C	-1.07529	0.96410	-1.85504
H	-2.16638	1.00105	-1.92681
C	-4.14597	7.28180	0.20997
H	-4.15200	6.33340	-0.33759
C	-7.27039	4.57888	3.25910

H	-7.28703	3.93110	2.37751
C	-5.29835	8.93928	4.83434
H	-4.68020	9.74842	4.42511
C	1.64231	4.82855	-0.18705
C	2.94561	4.79158	0.30331
C	3.18359	4.82088	1.67838
C	2.12051	4.90993	2.57157
C	0.82715	4.97039	2.05443
C	0.56246	4.91789	0.69201
H	1.45926	4.79006	-1.25579
H	3.78131	4.72051	-0.38542
H	4.19839	4.75756	2.06117
H	-0.46437	4.94611	0.33941
N	-0.22748	5.06894	3.03876
H	-0.20970	5.98412	3.49807
H	-1.15208	4.95721	2.57889
C	2.24468	4.82618	4.06642
H	3.28423	4.65278	4.35451
H	1.93637	5.76356	4.54716
C	1.36647	3.66634	4.58794
C	1.53012	3.46566	6.10203
C	-0.16627	3.93637	4.36238
C	1.75476	2.34492	3.91039
H	1.46329	2.34592	2.85692
H	2.83995	2.20029	3.97573
H	1.24417	1.51220	4.39594
O	2.30268	4.39663	6.65945
O	-0.59234	4.75603	5.39578
O	-0.84764	2.90853	4.02814
O	1.03262	2.54083	6.69364
C	2.62499	4.37925	8.08588
C	-1.97126	4.81044	5.85951
C	-2.43197	3.45696	6.40287
H	-1.64870	3.02558	7.03371
H	-3.33048	3.60959	7.01009
H	-2.66740	2.74901	5.60700
C	-2.90404	5.32476	4.76681
H	-3.90483	5.48491	5.18204
H	-2.56026	6.28364	4.35879
H	-3.00767	4.60942	3.94972
C	-1.89278	5.82211	6.99861
H	-1.47621	6.76973	6.64257
H	-2.89235	6.00784	7.40280
H	-1.25684	5.43765	7.80066
C	3.33360	3.07915	8.45778
H	3.74279	3.17774	9.46790
H	2.65476	2.22659	8.43510
H	4.16506	2.89487	7.76991
C	1.35641	4.60650	8.90044
H	0.63942	3.79581	8.75523
H	1.61607	4.65938	9.96228
H	0.89377	5.55516	8.61192
C	3.57500	5.56196	8.23070
H	3.87009	5.67472	9.27770
H	4.47459	5.40541	7.62825
H	3.08769	6.48436	7.90229
C	-8.71795	4.99313	3.54967
H	-8.77510	5.62584	4.44218

H	-9.14206	5.55376	2.71072
H	-9.34070	4.10981	3.72434
C	-6.68509	3.76244	4.41801
H	-6.67450	4.34629	5.34569
H	-7.28807	2.86544	4.59425
H	-5.65995	3.45154	4.19316
C	-4.80291	8.35524	-0.66793
H	-4.23525	8.49452	-1.59399
H	-5.83011	8.09025	-0.93398
H	-4.82973	9.31483	-0.13878
C	-2.67815	7.64546	0.46763
H	-2.12273	7.63169	-0.47631
H	-2.57897	8.65106	0.89032
H	-2.21801	6.92495	1.14912
C	-6.66048	9.53176	5.21336
H	-6.53934	10.33468	5.94769
H	-7.17136	9.93918	4.33601
H	-7.30837	8.76766	5.65668
C	-4.58615	8.38272	6.07200
H	-4.48858	9.15460	6.84266
H	-5.15015	7.54628	6.50203
H	-3.58569	8.01907	5.81965
C	-2.39936	-2.39975	2.77414
H	-1.43496	-2.87338	2.99132
H	-3.11614	-2.72474	3.53578
H	-2.74233	-2.76834	1.80397
C	-1.87418	-0.42217	4.21305
H	-1.60993	0.63734	4.25043
H	-2.71890	-0.59563	4.88793
H	-1.02680	-0.99770	4.60283
C	3.09936	-1.48011	1.09463
H	3.03558	-1.60541	0.00773
H	4.14888	-1.58315	1.39013
H	2.52683	-2.28691	1.56204
C	3.40684	1.01133	0.90004
H	4.41950	0.97250	1.31473
H	3.49534	0.90425	-0.18689
H	2.98639	2.00035	1.10854
C	-0.58052	-0.02070	-2.92216
H	0.51279	-0.09178	-2.90960
H	-0.98578	-1.02255	-2.74821
H	-0.88681	0.30736	-3.92092
C	-0.55251	2.37862	-2.12702
H	-0.92048	3.07612	-1.36827
H	0.54280	2.40605	-2.12010
H	-0.88922	2.72429	-3.11017

3. Cartesian coordinates of the S-conformer product structure

C	-7.48724	-2.03523	-0.92058
C	-6.13512	-2.13852	-0.71819
C	-5.34013	-0.98256	-0.49929
C	-5.95472	0.30174	-0.52426
C	-7.36185	0.37407	-0.71042
C	-8.10625	-0.76326	-0.90198
H	-3.49750	-2.08571	-0.23772
H	-8.08686	-2.92547	-1.08025
H	-5.64858	-3.11008	-0.70718

C	-3.94974	-1.09794	-0.22813
C	-5.13676	1.46577	-0.33275
H	-7.84856	1.34327	-0.68970
H	-9.18064	-0.68684	-1.03525
C	-3.81083	1.27347	-0.00737
C	-3.18129	-0.00040	0.06379
C	-5.66767	2.85366	-0.43785
C	-6.34886	3.32866	-1.60878
C	-5.45900	3.74501	0.59638
C	-6.88930	4.64590	-1.60544
C	-6.47983	2.55021	-2.79146
C	-5.95991	5.07469	0.60792
C	-7.58871	5.11690	-2.74821
C	-6.69661	5.48305	-0.47556
C	-7.14685	3.03937	-3.88672
H	-6.03525	1.56165	-2.82632
C	-7.72202	4.33209	-3.86400
H	-8.00459	6.12050	-2.72227
H	-7.12837	6.48062	-0.48334
H	-7.22925	2.43085	-4.78162
H	-8.25320	4.70253	-4.73473
O	-4.77352	3.33327	1.71826
O	-3.01194	2.37469	0.22173
P	-3.16039	3.14312	1.64518
O	-2.81017	2.28505	2.82284
H	-1.51116	2.62294	3.59604
O	-2.42633	4.44135	1.48532
C	-5.70215	5.98634	1.76526
C	-6.36262	5.77508	2.99223
C	-4.83047	7.08202	1.61350
C	-6.20165	6.70898	4.01554
C	-4.70650	7.99308	2.66750
C	-5.39466	7.83730	3.86729
H	-6.72817	6.55243	4.95574
H	-4.05590	8.85710	2.54781
C	-1.73080	-0.06694	0.42236
C	-0.77142	0.37764	-0.50570
C	-1.33231	-0.49845	1.70501
C	0.57810	0.36795	-0.14667
C	0.02871	-0.49073	2.01613
C	0.99882	-0.05899	1.11115
H	1.31556	0.70494	-0.87151
H	0.35041	-0.81527	3.00349
C	-2.33664	-0.98668	2.73925
H	-3.30517	-0.53468	2.50081
C	2.46725	-0.12128	1.48986
H	2.52196	-0.03673	2.58351
C	-1.15009	0.83808	-1.90550
H	-2.24119	0.86004	-1.98268
C	-4.02107	7.31102	0.34484
H	-4.05381	6.39084	-0.24717
C	-7.25090	4.56688	3.24434
H	-7.25769	3.94396	2.34519
C	-5.27235	8.86076	4.98079
H	-4.61916	9.66414	4.61701
C	1.65028	4.58409	-0.20516
C	2.95083	4.72226	0.27427
C	3.19168	4.85729	1.64249

C	2.12789	4.88184	2.53845
C	0.83835	4.75893	2.02808
C	0.56874	4.59882	0.67665
H	1.46910	4.46682	-1.26819
H	3.78615	4.70702	-0.41828
H	4.20800	4.93463	2.01754
H	-0.45723	4.49887	0.33542
N	-0.23446	4.82796	3.01733
H	-0.28882	5.77868	3.40143
H	-1.15992	4.65508	2.52261
C	2.25110	4.92173	4.03468
H	3.29685	4.84663	4.34028
H	1.87070	5.86766	4.44197
C	1.45287	3.73938	4.62725
C	1.57085	3.67484	6.16024
C	-0.07816	3.88817	4.30225
C	1.99435	2.40680	4.08554
H	1.77381	2.29933	3.02140
H	3.08093	2.37225	4.22520
H	1.53272	1.57177	4.61255
O	2.26165	4.69935	6.65332
O	-0.62250	4.64058	5.32625
O	-0.59649	2.68891	4.02396
O	1.12519	2.76302	6.80863
C	2.60568	4.79257	8.07360
C	-2.01753	4.63747	5.76710
C	-2.50929	3.23326	6.12330
H	-1.72829	2.68816	6.66127
H	-3.38225	3.33409	6.77659
H	-2.81559	2.65693	5.24814
C	-2.90899	5.30832	4.72941
H	-3.92196	5.42094	5.12988
H	-2.53964	6.30943	4.47463
H	-2.99466	4.72165	3.81318
C	-1.94389	5.48970	7.02971
H	-1.49066	6.46174	6.81044
H	-2.95042	5.65229	7.42616
H	-1.34335	4.98288	7.79012
C	3.44440	3.58689	8.48850
H	3.84266	3.76232	9.49243
H	2.85464	2.66979	8.50158
H	4.28901	3.46244	7.80331
C	1.33909	4.93588	8.90904
H	0.72462	4.03526	8.85814
H	1.61871	5.11169	9.95238
H	0.75497	5.79298	8.56047
C	3.43668	6.06828	8.13361
H	3.74962	6.25868	9.16394
H	4.32930	5.97279	7.50852
H	2.85034	6.92201	7.78206
C	-8.69933	4.98740	3.52097
H	-8.76768	5.59427	4.43052
H	-9.10281	5.57624	2.69120
H	-9.33345	4.10555	3.65836
C	-6.69337	3.71299	4.39004
H	-6.69396	4.27127	5.33361
H	-7.30725	2.81708	4.53106
H	-5.66763	3.39886	4.17235

C	-4.61747	8.44914	-0.49340
H	-4.03121	8.60350	-1.40537
H	-5.65116	8.23901	-0.78326
H	-4.61227	9.38595	0.07573
C	-2.54576	7.59803	0.65047
H	-1.97247	7.62999	-0.28236
H	-2.41588	8.56472	1.14897
H	-2.13104	6.80872	1.28337
C	-6.63462	9.47715	5.31961
H	-6.52578	10.25755	6.07977
H	-7.09852	9.91948	4.43311
H	-7.31811	8.71748	5.71453
C	-4.62548	8.25806	6.23297
H	-4.54604	9.00870	7.02632
H	-5.22442	7.42392	6.61752
H	-3.62222	7.87944	6.01575
C	-2.47561	-2.51476	2.66933
H	-1.51383	-2.98935	2.89634
H	-3.20964	-2.86731	3.40176
H	-2.79022	-2.85505	1.67936
C	-1.98267	-0.57705	4.17258
H	-1.77358	0.49198	4.24178
H	-2.82202	-0.80997	4.83627
H	-1.11220	-1.12713	4.54716
C	3.05347	-1.48445	1.09659
H	2.99722	-1.61951	0.01046
H	4.10414	-1.55848	1.39688
H	2.49978	-2.30196	1.56795
C	3.29881	1.01232	0.88516
H	4.30479	1.01534	1.31762
H	3.40909	0.89034	-0.19824
H	2.84221	1.99096	1.06592
C	-0.63716	-0.15806	-2.95346
H	0.45702	-0.21403	-2.93501
H	-1.02945	-1.16220	-2.76396
H	-0.94331	0.14848	-3.95910
C	-0.64436	2.25395	-2.20020
H	-1.03510	2.95995	-1.46105
H	0.45043	2.29596	-2.18097
H	-0.97309	2.57485	-3.19434

4. Cartesian coordinates of the R-conformer reactant structure

C	-6.74513	-1.60599	-1.31699
C	-5.37741	-1.69043	-1.34529
C	-4.57437	-0.52770	-1.20403
C	-5.19647	0.74565	-1.07599
C	-6.61594	0.79447	-1.01409
C	-7.36652	-0.34915	-1.12939
H	-2.70146	-1.61203	-1.17778
H	-7.35219	-2.49997	-1.41518
H	-4.88102	-2.65100	-1.45342
C	-3.16263	-0.63125	-1.10570
C	-4.36761	1.92003	-0.99209
H	-7.10913	1.74724	-0.85837
H	-8.44832	-0.28904	-1.06804
C	-3.00855	1.73543	-0.84226
C	-2.37994	0.45994	-0.83185

C	-4.94977	3.28907	-1.01422
C	-5.88996	3.70114	-2.02159
C	-4.60714	4.20945	-0.04264
C	-6.54603	4.95574	-1.88389
C	-6.17127	2.92048	-3.17590
C	-5.20138	5.49622	0.07563
C	-7.50308	5.35678	-2.85365
C	-6.19488	5.81881	-0.81367
C	-7.07764	3.34795	-4.11530
H	-5.65139	1.97922	-3.31828
C	-7.76838	4.57169	-3.94630
H	-8.00361	6.31203	-2.72095
H	-6.71293	6.76870	-0.71402
H	-7.26811	2.73977	-4.99387
H	-8.49328	4.89040	-4.68822
O	-3.69949	3.86528	0.94401
O	-2.17469	2.84033	-0.74378
P	-2.12898	3.72661	0.60697
O	-1.60388	5.10026	0.06733
H	-1.58657	5.29031	-0.90146
O	-1.40630	3.19839	1.76293
C	-4.76933	6.43461	1.15518
C	-3.98077	7.55540	0.83139
C	-5.15646	6.19497	2.48740
C	-3.64089	8.44854	1.85076
C	-4.78539	7.11179	3.47006
C	-4.03537	8.24958	3.17146
H	-3.03980	9.32282	1.61280
H	-5.09140	6.93154	4.49900
C	-0.93954	0.31849	-0.47096
C	-0.58507	-0.24167	0.77771
C	0.05758	0.74466	-1.36604
C	0.77409	-0.39020	1.06597
C	1.40032	0.57026	-1.02521
C	1.78157	-0.00123	0.18384
H	1.04992	-0.81938	2.02786
H	2.17313	0.89052	-1.72213
C	-0.27147	1.38680	-2.70358
H	-1.36014	1.45080	-2.80387
C	3.24462	-0.17751	0.54101
H	3.83426	0.18891	-0.30898
C	-1.54544	-0.71935	1.87288
H	-0.94228	-0.67342	2.78779
C	-5.98014	4.98114	2.88849
H	-6.13174	4.35462	2.00407
C	-3.49109	7.81475	-0.58636
H	-3.53225	6.86591	-1.13305
C	-3.64523	9.23256	4.25848
H	-3.07242	10.03758	3.78106
C	-1.38756	11.15108	-2.77714
C	-0.11296	10.73803	-2.39304
C	0.44125	9.61937	-3.00299
C	-0.24268	8.88698	-3.97961
C	-1.52246	9.32546	-4.37721
C	-2.07873	10.45660	-3.75927
H	-1.84564	12.02044	-2.31539
H	0.44216	11.28127	-1.63614
H	1.44314	9.30081	-2.72660

H	-3.06674	10.78911	-4.06850
N	-2.20531	8.71576	-5.43506
H	-2.08627	7.71169	-5.50943
H	-3.18185	8.97860	-5.50107
C	0.42451	7.69587	-4.62673
H	1.49033	7.91694	-4.75901
H	0.01448	7.51174	-5.62468
C	0.36909	6.35939	-3.80976
C	1.11879	5.35741	-4.71662
C	-1.07454	5.88627	-3.61730
C	1.02476	6.49938	-2.43550
H	0.43029	7.16360	-1.80429
H	2.03175	6.90680	-2.53768
H	1.09573	5.52752	-1.93976
O	2.40059	5.29223	-4.37478
O	-1.75954	5.80806	-4.73990
O	-1.54524	5.60835	-2.53408
O	0.62430	4.78200	-5.65219
C	3.38350	4.57824	-5.19871
C	-3.06319	5.11612	-4.79191
C	-2.87825	3.66783	-4.34840
H	-2.07717	3.19585	-4.92794
H	-3.80999	3.12770	-4.53980
H	-2.64926	3.59211	-3.28045
C	-4.09538	5.85801	-3.95111
H	-5.09323	5.50295	-4.22775
H	-4.04633	6.93605	-4.14305
H	-3.94832	5.68098	-2.88414
C	-3.41346	5.18431	-6.27252
H	-3.53827	6.22211	-6.59918
H	-4.35552	4.65658	-6.44583
H	-2.62756	4.71824	-6.87282
C	3.09494	3.08080	-5.18121
H	3.86063	2.56747	-5.77115
H	2.11591	2.85485	-5.60808
H	3.13982	2.69639	-4.15787
C	3.39276	5.15363	-6.61234
H	2.49713	4.87763	-7.16973
H	4.26871	4.76905	-7.14321
H	3.47061	6.24513	-6.57321
C	4.69594	4.88226	-4.48794
H	5.51769	4.36832	-4.99433
H	4.65440	4.54006	-3.45013
H	4.89641	5.95752	-4.49658
C	-4.39225	8.81761	-1.31807
H	-4.38550	9.78327	-0.79849
H	-5.42806	8.47158	-1.37658
H	-4.02231	8.98212	-2.33659
C	-2.03974	8.30063	-0.63797
H	-1.93572	9.31395	-0.23564
H	-1.70469	8.33882	-1.68070
H	-1.37653	7.63481	-0.07667
C	-7.36556	5.40603	3.39098
H	-7.96829	4.52759	3.64353
H	-7.89944	5.98423	2.63003
H	-7.28154	6.02701	4.28969
C	-5.25117	4.12835	3.93333
H	-5.83493	3.23026	4.16117

H	-5.11155	4.68144	4.86858
H	-4.26846	3.81937	3.56500
C	-2.74580	8.57034	5.30860
H	-2.42965	9.30070	6.06062
H	-1.85279	8.13892	4.84684
H	-3.28033	7.76568	5.82543
C	-4.88016	9.85695	4.91794
H	-4.58282	10.59995	5.66512
H	-5.47807	9.09208	5.42575
H	-5.51860	10.34750	4.17722
C	0.24724	0.54556	-3.87595
H	1.34018	0.47041	-3.85452
H	-0.03747	1.00494	-4.82921
H	-0.16210	-0.46891	-3.84534
C	0.28203	2.81354	-2.77320
H	-0.12631	3.42719	-1.96600
H	0.01014	3.26744	-3.73231
H	1.37498	2.82095	-2.69396
C	3.59378	-1.65427	0.75781
H	3.04103	-2.06258	1.61097
H	4.66258	-1.77011	0.96489
H	3.34513	-2.25302	-0.12342
C	3.62027	0.65569	1.77182
H	4.68954	0.56199	1.98809
H	3.06750	0.31520	2.65407
H	3.38829	1.71358	1.61739
C	-2.78641	0.13748	2.15980
H	-3.58390	-0.01391	1.42706
H	-2.53444	1.20067	2.19821
H	-3.18779	-0.15124	3.13761
C	-1.93419	-2.19523	1.69688
H	-1.05514	-2.81866	1.50534
H	-2.63703	-2.32570	0.86886
H	-2.42425	-2.56152	2.60532

5. Cartesian coordinates of the R-conformer transition state structure

C	-6.37991	-1.73726	1.01342
C	-5.02488	-1.72614	0.80424
C	-4.36807	-0.55340	0.34762
C	-5.13285	0.61488	0.07135
C	-6.53159	0.57943	0.32239
C	-7.13578	-0.56428	0.78292
H	-2.38527	-1.42457	0.39499
H	-6.87224	-2.63621	1.36980
H	-4.42553	-2.61132	0.99904
C	-2.95548	-0.52044	0.19852
C	-4.46132	1.78474	-0.42690
H	-7.12335	1.47396	0.16069
H	-8.20358	-0.56678	0.97710
C	-3.08594	1.77492	-0.46312
C	-2.29954	0.63266	-0.14155
C	-5.22767	2.99456	-0.83027
C	-6.28000	2.93917	-1.80625
C	-4.96950	4.20785	-0.22904
C	-7.09778	4.08598	-2.00991
C	-6.51186	1.78975	-2.60876

C	-5.85498	5.31832	-0.31345
C	-8.13400	4.03904	-2.97869
C	-6.89404	5.23940	-1.20506
C	-7.51215	1.77994	-3.54899
H	-5.87610	0.91978	-2.48184
C	-8.34029	2.91213	-3.73247
H	-8.75694	4.91904	-3.11347
H	-7.60465	6.05998	-1.27065
H	-7.66699	0.89685	-4.16052
H	-9.13167	2.88715	-4.47454
O	-3.84848	4.35782	0.56644
O	-2.41347	2.88928	-0.91951
P	-2.37656	4.29976	-0.11868
O	-2.35000	5.31244	-1.28842
H	-1.74537	5.01976	-2.25114
O	-1.35390	4.41794	0.93070
C	-5.78000	6.40746	0.70978
C	-5.12172	7.61928	0.46326
C	-6.42467	6.18235	1.94783
C	-5.14081	8.60998	1.45082
C	-6.39434	7.18995	2.90952
C	-5.76468	8.41479	2.67815
H	-4.65355	9.56371	1.26124
H	-6.88017	7.01988	3.86735
C	-0.81592	0.74717	-0.23527
C	-0.02907	0.96259	0.91407
C	-0.22630	0.71159	-1.51320
C	1.34239	1.17078	0.73819
C	1.15050	0.90819	-1.62863
C	1.94935	1.16537	-0.51730
H	1.95208	1.34683	1.62288
H	1.61442	0.87444	-2.61288
C	-1.04322	0.39627	-2.75866
H	-2.10435	0.46674	-2.50184
C	3.43856	1.41143	-0.66803
H	3.64929	1.47605	-1.74379
C	-0.52423	0.91216	2.36022
H	0.25004	1.43246	2.93755
C	-7.10660	4.85760	2.26814
H	-7.36015	4.36760	1.32242
C	-4.41998	7.88777	-0.85684
H	-4.27732	6.92757	-1.36463
C	-5.76059	9.49960	3.73812
H	-5.21618	10.35825	3.32508
C	1.69517	6.65759	1.07303
C	2.98963	6.52904	0.57605
C	3.21749	6.52254	-0.80062
C	2.15669	6.66980	-1.68895
C	0.87390	6.83588	-1.16627
C	0.62058	6.81086	0.19818
H	1.51235	6.62851	2.14163
H	3.82294	6.40516	1.26005
H	4.22117	6.37381	-1.18936
H	-0.39581	6.87269	0.57332
N	-0.17966	6.96090	-2.14251
H	-0.13417	7.85954	-2.62945
H	-1.10217	6.87076	-1.69925
C	2.27207	6.49543	-3.17657

H	3.30051	6.24669	-3.44710
H	2.01394	7.41290	-3.72050
C	1.33923	5.34880	-3.63421
C	1.55774	4.99316	-5.11543
C	-0.16470	5.74771	-3.54905
C	1.57728	4.07193	-2.81850
H	1.22358	4.18264	-1.78884
H	2.64914	3.84099	-2.79522
H	1.03783	3.24033	-3.26857
O	2.46475	5.76998	-5.70056
O	-0.44844	6.59258	-4.57885
O	-1.00572	4.84479	-3.21229
O	0.97017	4.08932	-5.65453
C	2.83595	5.59831	-7.10633
C	-1.78593	6.71793	-5.15608
C	-2.24848	5.39067	-5.75524
H	-1.43902	4.94986	-6.34535
H	-3.10032	5.58263	-6.41555
H	-2.54696	4.67670	-4.98779
C	-2.76955	7.26490	-4.12597
H	-3.74588	7.40297	-4.60170
H	-2.43602	8.24467	-3.76473
H	-2.90892	6.58806	-3.27800
C	-1.56955	7.74247	-6.26433
H	-1.15234	8.66792	-5.85656
H	-2.52275	7.97054	-6.74944
H	-0.88184	7.34622	-7.01621
C	3.38981	4.19615	-7.34458
H	3.84304	4.16154	-8.33997
H	2.60976	3.43691	-7.28699
H	4.16728	3.96944	-6.60812
C	1.63492	5.91151	-7.99186
H	0.82266	5.20029	-7.82947
H	1.93991	5.86016	-9.04154
H	1.27639	6.92502	-7.78777
C	3.92947	6.64246	-7.29466
H	4.27618	6.63295	-8.33175
H	4.77907	6.42861	-6.63964
H	3.54780	7.64051	-7.06156
C	-5.28128	8.78532	-1.75355
H	-5.46419	9.75155	-1.26951
H	-6.25113	8.32077	-1.95761
H	-4.78212	8.97320	-2.71068
C	-3.03373	8.50175	-0.64337
H	-3.09003	9.49539	-0.18784
H	-2.52293	8.62246	-1.60472
H	-2.42434	7.86407	0.00570
C	-8.41344	5.02888	3.04926
H	-8.92778	4.06574	3.12444
H	-9.08394	5.74019	2.55731
H	-8.23330	5.38011	4.07050
C	-6.14693	3.93114	3.03025
H	-6.61928	2.96007	3.21471
H	-5.88572	4.37499	3.99811
H	-5.21854	3.76638	2.47496
C	-5.02695	9.03882	5.00284
H	-4.98733	9.84671	5.74075
H	-4.00346	8.72780	4.77370

H	-5.54263	8.18882	5.46303
C	-7.18411	9.95890	4.07366
H	-7.16315	10.77504	4.80315
H	-7.76561	9.13730	4.50623
H	-7.70775	10.30795	3.17877
C	-0.77455	-1.04920	-3.19866
H	0.28029	-1.17959	-3.46622
H	-1.38146	-1.30597	-4.07328
H	-1.00898	-1.75521	-2.39517
C	-0.79496	1.37600	-3.90843
H	-0.98488	2.40901	-3.60027
H	-1.45811	1.13890	-4.74770
H	0.23355	1.31365	-4.28239
C	4.24912	0.24208	-0.09581
H	4.06691	0.13990	0.97987
H	5.32206	0.40465	-0.24280
H	3.97267	-0.70137	-0.57588
C	3.86728	2.73388	-0.02199
H	4.92778	2.92847	-0.21565
H	3.72777	2.70339	1.06422
H	3.28364	3.57497	-0.40913
C	-1.84463	1.61736	2.69909
H	-2.71930	1.05043	2.36924
H	-1.88264	2.61974	2.26541
H	-1.91407	1.71668	3.78823
C	-0.54732	-0.53881	2.86393
H	0.41129	-1.03652	2.68716
H	-1.32964	-1.11246	2.35710
H	-0.75779	-0.56360	3.93858

6. Cartesian coordinates of the R-conformer product structure

C	-6.60072	-1.83360	-0.26211
C	-5.23057	-1.80797	-0.22349
C	-4.52037	-0.58894	-0.38315
C	-5.24058	0.61746	-0.60891
C	-6.66139	0.56118	-0.62120
C	-7.32090	-0.63072	-0.45190
H	-2.56870	-1.47905	-0.10041
H	-7.13541	-2.76925	-0.13460
H	-4.66236	-2.71961	-0.05985
C	-3.10461	-0.55192	-0.29035
C	-4.51333	1.84378	-0.77816
H	-7.22716	1.47697	-0.75322
H	-8.40605	-0.64978	-0.45700
C	-3.14377	1.82211	-0.60700
C	-2.40887	0.62541	-0.36885
C	-5.20478	3.12791	-1.07003
C	-6.04384	3.30260	-2.21976
C	-5.00229	4.20769	-0.23741
C	-6.72062	4.54199	-2.39426
C	-6.17787	2.30991	-3.22800
C	-5.66305	5.45843	-0.39423
C	-7.54241	4.73230	-3.53664
C	-6.53349	5.58398	-1.44796
C	-6.96291	2.53096	-4.33251
H	-5.63750	1.37478	-3.12444

C	-7.66411	3.75058	-4.48646
H	-8.06050	5.68092	-3.64922
H	-7.08099	6.51369	-1.58031
H	-7.04438	1.76482	-5.09697
H	-8.28700	3.90862	-5.36092
O	-4.12797	4.07567	0.82111
O	-2.43037	3.00352	-0.69887
P	-2.53804	4.18250	0.46893
O	-2.29936	5.44154	-0.33038
H	-1.24441	3.79226	-2.08825
O	-1.75069	3.88681	1.67989
C	-5.42530	6.57289	0.57145
C	-4.79207	7.75753	0.14753
C	-5.85560	6.44368	1.90871
C	-4.63699	8.80319	1.06201
C	-5.66818	7.51172	2.78492
C	-5.06657	8.70394	2.38177
H	-4.15884	9.72529	0.73745
H	-6.00976	7.41143	3.81355
C	-0.92684	0.73264	-0.23482
C	-0.32369	0.91234	1.02224
C	-0.15380	0.76837	-1.41840
C	1.04441	1.21411	1.05248
C	1.20688	1.04933	-1.32518
C	1.81784	1.31434	-0.09917
H	1.50919	1.37861	2.02397
H	1.81172	1.08851	-2.22670
C	-0.78182	0.42890	-2.76629
H	-1.79413	0.85525	-2.78630
C	3.28695	1.68564	-0.02888
H	3.65106	1.76816	-1.06198
C	-0.96577	0.69831	2.39622
H	-0.46584	1.41713	3.05661
C	-6.55595	5.19728	2.42975
H	-6.58480	4.44976	1.63235
C	-4.26124	7.94701	-1.26582
H	-4.26035	6.97060	-1.75765
C	-4.88438	9.85396	3.35318
H	-4.39077	10.66823	2.80755
C	1.88484	6.35772	1.27532
C	3.09735	6.67308	0.66643
C	3.19054	6.77264	-0.72291
C	2.05854	6.58433	-1.50817
C	0.85782	6.28273	-0.87144
C	0.73931	6.15156	0.50469
H	1.82400	6.26177	2.35381
H	3.98273	6.82494	1.27522
H	4.14245	6.99218	-1.19737
H	-0.21020	5.89170	0.96273
N	-0.29082	6.13785	-1.75889
H	-0.53140	7.04677	-2.17111
H	-1.16358	5.83325	-1.17699
C	2.01737	6.57721	-3.00914
H	3.02293	6.65158	-3.42999
H	1.44954	7.43319	-3.39522
C	1.36273	5.25516	-3.47924
C	1.34466	5.16076	-5.01493
C	-0.12922	5.18477	-2.97712

C	2.18169	4.06154	-2.96409
H	2.07372	3.94162	-1.88463
H	3.23931	4.22521	-3.19835
H	1.85065	3.14046	-3.44075
O	1.57608	6.33311	-5.59601
O	-0.95967	5.76608	-3.92332
O	-0.40967	3.91369	-2.58355
O	1.18220	4.11902	-5.59813
C	1.76595	6.46295	-7.04260
C	-2.21890	5.21957	-4.44619
C	-1.91906	4.13051	-5.46698
H	-1.33687	4.52565	-6.30237
H	-2.86509	3.73394	-5.85044
H	-1.35678	3.31321	-5.00812
C	-3.16606	4.69911	-3.36567
H	-4.18940	4.74223	-3.75414
H	-3.12867	5.30390	-2.45357
H	-2.97658	3.65294	-3.10300
C	-2.83344	6.44594	-5.11254
H	-3.11425	7.18902	-4.35819
H	-3.73072	6.15547	-5.66617
H	-2.12125	6.90101	-5.80649
C	3.00327	5.67587	-7.46317
H	3.21540	5.88121	-8.51669
H	2.85385	4.60162	-7.34241
H	3.86922	5.98929	-6.87174
C	0.51694	6.01870	-7.79608
H	0.38154	4.93753	-7.74811
H	0.61410	6.31486	-8.84514
H	-0.36829	6.51288	-7.38315
C	1.99164	7.95967	-7.21532
H	2.17225	8.18829	-8.26924
H	2.85841	8.28462	-6.63273
H	1.11290	8.51860	-6.88071
C	-5.15537	8.89568	-2.07483
H	-5.18559	9.88588	-1.60599
H	-6.18290	8.52660	-2.14049
H	-4.76977	9.01638	-3.09347
C	-2.81662	8.46474	-1.26919
H	-2.76036	9.51256	-0.95557
H	-2.40735	8.41245	-2.28735
H	-2.19582	7.86883	-0.59366
C	-8.00893	5.51275	2.80832
H	-8.52247	4.60555	3.14315
H	-8.55711	5.92465	1.95500
H	-8.05228	6.24438	3.62270
C	-5.80092	4.57741	3.61112
H	-6.29173	3.65071	3.92693
H	-5.78533	5.25565	4.47143
H	-4.76913	4.34590	3.33168
C	-3.98201	9.45483	4.52635
H	-3.81876	10.30706	5.19416
H	-3.00948	9.09990	4.17250
H	-4.44082	8.65137	5.11296
C	-6.23435	10.37420	3.86033
H	-6.09154	11.23444	4.52243
H	-6.76081	9.59832	4.42708
H	-6.87732	10.67970	3.02958

C	-0.90419	-1.09645	-2.91316
H	0.09354	-1.54944	-2.88989
H	-1.37346	-1.35391	-3.86868
H	-1.49612	-1.54047	-2.11000
C	-0.02590	0.98049	-3.97687
H	0.13098	2.05835	-3.89918
H	-0.60093	0.78019	-4.88737
H	0.94740	0.49114	-4.09833
C	4.10414	0.59737	0.67707
H	3.77445	0.48201	1.71565
H	5.16799	0.85670	0.68811
H	3.98848	-0.36898	0.17732
C	3.48883	3.04379	0.65220
H	4.54408	3.33728	0.62680
H	3.17566	3.00626	1.70165
H	2.90224	3.82746	0.16209
C	-2.47062	0.90790	2.60287
H	-3.06507	0.08717	2.19441
H	-2.81970	1.85116	2.18105
H	-2.65713	0.94052	3.68230
C	-0.59014	-0.71423	2.87814
H	0.49262	-0.87198	2.86408
H	-1.04962	-1.46745	2.22704
H	-0.95267	-0.88154	3.89806

Enantioselective Recrystallization Procedure

The lactam was first dissolved in the minimal amount of ethyl acetate in a flat-bottom flask while remaining very warm on the hot plate. After the ethyl acetate-lactam solution cooled to room temperature, hexanes were carefully added until cloudiness was observed. The flask was capped and left at room temperature for several hours. After crystal formation was observed, ice was added to cool the solution and encourage further crystal growth. Vacuum filtration was used to separate the crystals from the mother liquor. These crystals were then sampled and run on the chiral HPLC to determine enantiomeric excess. (Chiralpak AD-H 4.6 mm x 250 mm x 5 μm column (OD-H, isopropanol/n-hexanes = 10/90, flow rate = 1.0 mL/min, l = 254 nm)).

X-ray Crystallography Information

Crystal preparation

The crystals were grown according to the layering method. First, a recrystallization was conducted to improve the ee of the sample, allowing for a higher quality crystal to grow and minimizing the chances of a crystal growing racemically. The solid enantioenriched material was then dissolved in a minimal amount of very hot ethyl acetate in a scintillation vial and placed in an environment prone to little disruption. After the solution had cooled, hexanes were carefully layered on top of the ethyl acetate layer. There was roughly a 5:1 ratio of hexanes to ethyl acetate. The scintillation vial was capped and left untouched. Crystals began forming after approximately one week.

UNC CHAPEL HILL DEPARTMENT OF CHEMISTRY

X-ray Core Laboratory

Report No. 23162

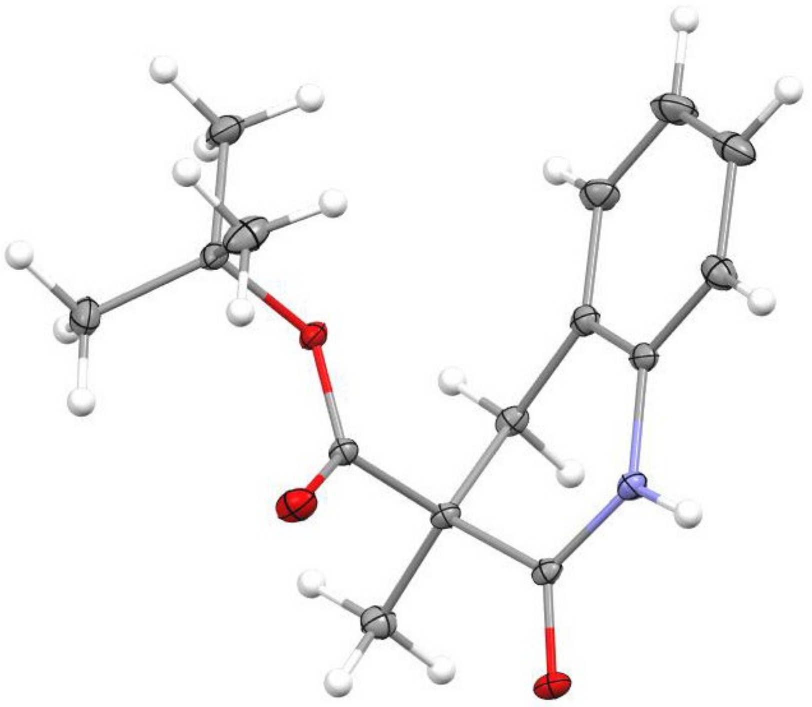

$C_{15} H_{19} N_1 O_3$

Prepared for
Abbie Horchar and Prof. K. Petersen

by
C. Chen

October 2, 2023

THE UNIVERSITY of NORTH CAROLINA at CHAPEL HILL

The sample was submitted by Abigail Horchar (research group of Petersen, Department of Chemistry, the University of North Carolina at Greensboro). A colorless crystal (approximate dimensions 0.250 x 0.070 x 0.050 mm^3) was placed onto the tip of MiTeGen and mounted on a Bruker D8 VENTURE diffractometer and measured at 150 K. The ellipsoid contour is displayed with 50% probability.

Data collection

A preliminary set of cell constants was calculated from reflections harvested from a set of 180 frames. These initial sets of frames were oriented such half a sphere in the reciprocal space was surveyed. This produced initial orientation matrices determined from 793 reflections. The data collection was carried out using Cu Kα radiation (graphite monochromator) with theta-dependent frame window between 0.8-1 second and a detector distance of 3.7 cm. A randomly oriented region of reciprocal space was surveyed to achieve complete data with a redundancy of 6.3. Sections of frames were collected with 1.0º steps in ω and ϕ scans. Data to a resolution of 0.82 Å were considered in the reduction. Final cell constants were calculated from the xyz centroids of 8336 strong reflections from the actual data collection after integration (SAINT).[1] The intensity data were corrected for absorption (SADABS).[2] Please refer to Table 1 for additional crystal and refinement information.

Structure solution and refinement

The space group $P2_12_12_1$ was determined based on intensity statistics and systematic absences. The structure was solved using SHELXT[3] and refined (full-matrix-least squares) using the Oxford University Crystals for Windows system.[4] The intrinsic-phasing solution provided most non-hydrogen atoms from the E-map. Full-matrix least squares / difference Fourier cycles were performed, which located the remaining non-hydrogen atoms. All non-hydrogen atoms were refined with anisotropic displacement parameters. The hydrogen atoms were placed in ideal positions and refined with individual relative isotropic displacement parameters. The final full matrix least squares refinement converged to R1 = 0.0289 and wR2 = 0.0730 (F^2, all data).

Structure description

The structure was found as proposed. Flack parameter for the structure was -0.02(17), confirming the chirality of the structure.

Acknowledgement to be included in publication

This material is based upon work supported by the National Science Foundation under Grant No. (CHE-2117287).

[1] SAINT, Bruker Analytical X-Ray Systems, Madison, WI.

[2] An empirical correction for absorption anisotropy, R. Blessing, Acta Cryst. A51, 33 - 38 (1995).

[3] Sheldrick, G. M. (2015): SHELXT – Integrated space-group and crystal-structure determination. Acta Cryst. A71, 3-8.

[4] Betteridge, P. W.; Carruthers, J. R.; Cooper, R. I.; Prout, K.; Watkin, D. J. J. Appl. Cryst. 2003, 36, 1487.

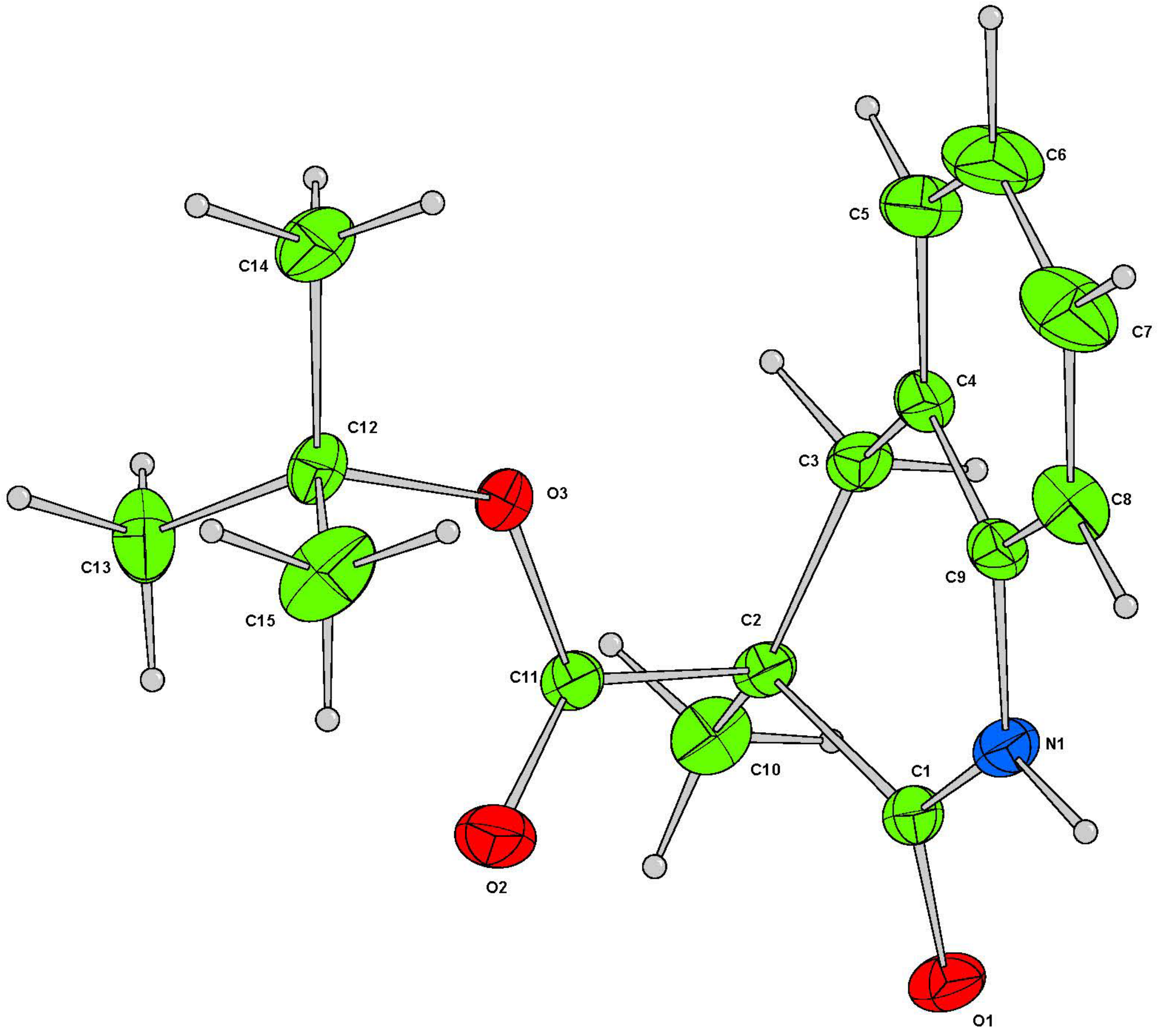

Molecular structure with labels on the asymmetric unit

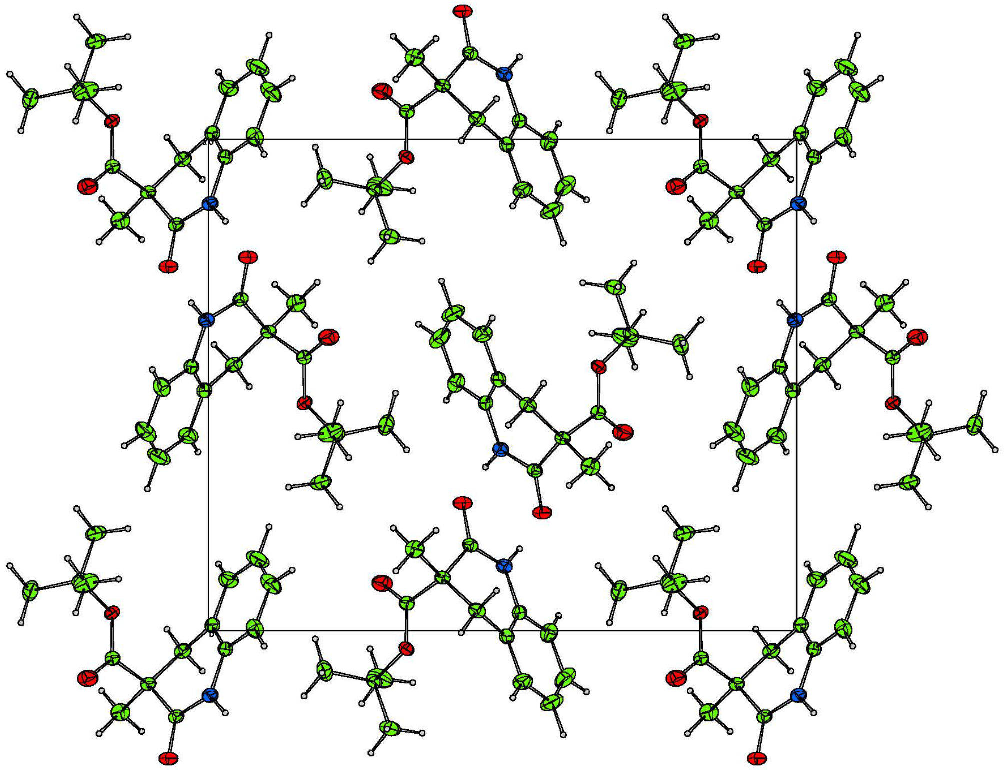

Cell plot, viewed along a- axis

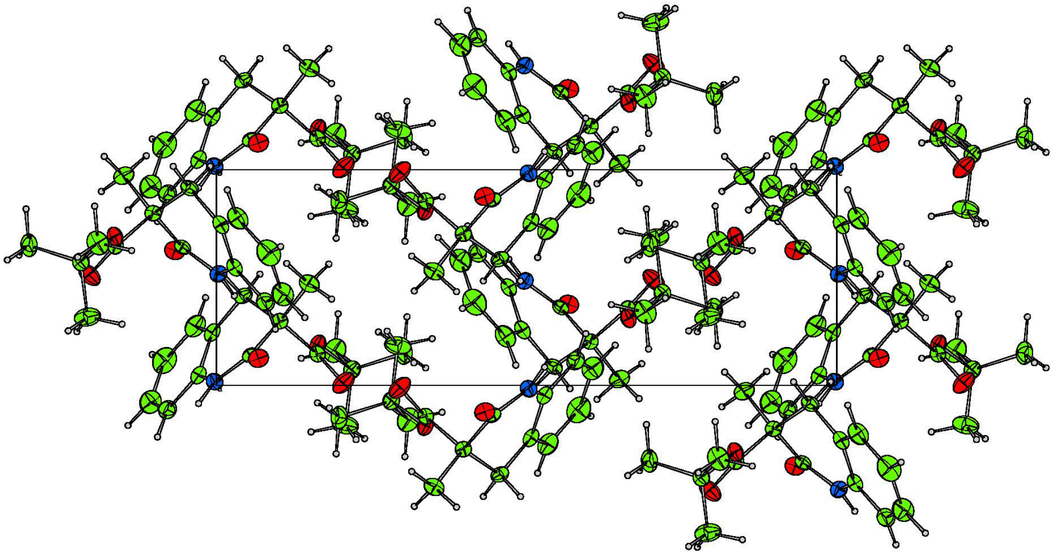

Cell plot, viewed along b- axis

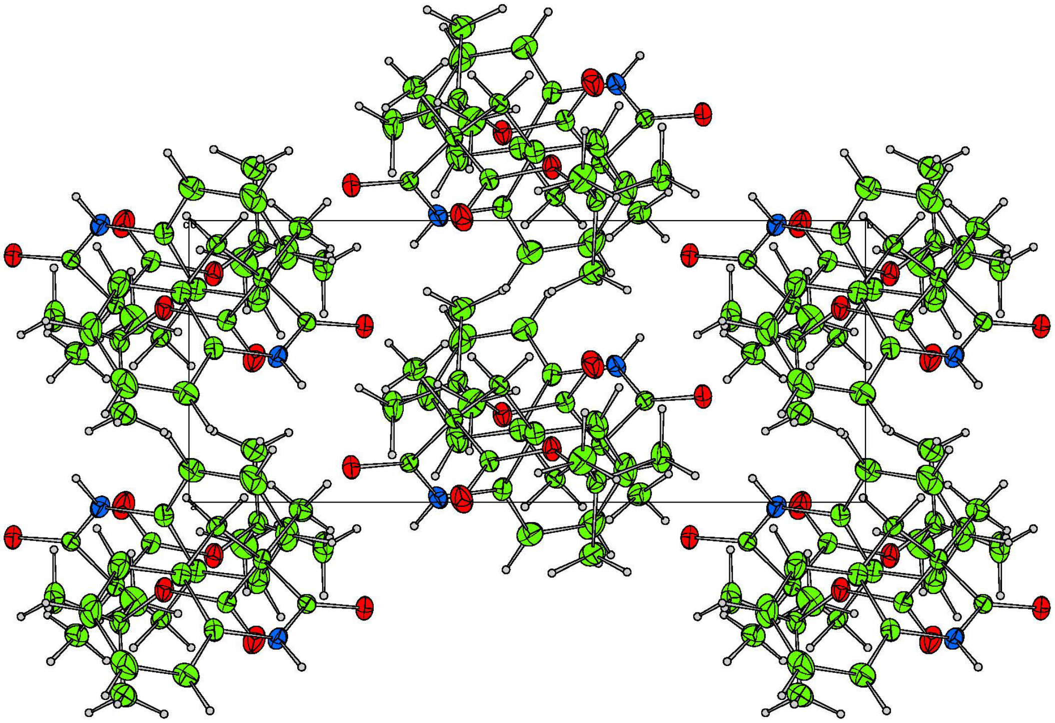

Cell plot, viewed along c- axis

Table 1. Crystal data and structure refinement for 23162.

Empirical formula	C15 H19 N1 O3	
Formula weight	261.32	
Crystal color, shape, size	colorless block, 0.250 x 0.070 x 0.050 mm^3	
Temperature	150 K	
Wavelength	1.54178 Å	
Crystal system, space group	Orthorhombic, $P2_12_12_1$	
Unit cell dimensions	a = 5.8737(2) Å	α= 90°.
	b = 14.1000(4) Å	β= 90°.
	c = 16.8472(4) Å	γ = 90°.
Volume	1395.27(7) $Å^3$	
Z	4	
Density (calculated)	1.244 Mg/m^3	
Absorption coefficient	0.701 mm^{-1}	
F(000)	560	
Data collection		
Diffractometer	Bruker D8 VENTURE, Bruker	
Theta range for data collection	4.088 to 70.060°.	
Index ranges	-7<=h<=7, -17<=k<=15, -20<=l<=20	
Reflections collected	14503	
Independent reflections	2610 [R(int) = 0.042]	
Observed Reflections	2507	
Completeness to theta = 70.060°	99.1 %	
Solution and Refinement		
Absorption correction	Semi-empirical from equivalents	
Max. and min. transmission	0.97 and 0.95	
Solution	Intrinsic phasing methods	
Refinement method	Full-matrix least-squares on F^2	
Weighting scheme	$w = [\sigma^2Fo^2 + AP^2 + BP]^{-1}$, with	
	$P = (Fo^2 + 2 Fc^2)/3$, A = 0.039, B = 0.260	
Data / restraints / parameters	2600 / 4 / 231	
Goodness-of-fit on F^2	1.0009	
Final R indices [I>2sigma(I)]	R1 = 0.0289, wR2 = 0.0717	
R indices (all data)	R1 = 0.0306, wR2 = 0.0730	
Absolute structure parameter	-0.02(17)	
Largest diff. peak and hole	0.17 and -0.13 e.$Å^{-3}$	

Table 2. Atomic coordinates (x 10^4) and equivalent isotropic displacement parameters (Å^{2}x 10^3) for 23162. U(eq) is defined as one third of the trace of the orthogonalized U^{ij} tensor.

	x	y	z	U(eq)
O1	6243(2)	7598(1)	5676(1)	23
O2	5117(2)	5967(1)	7047(1)	31
O3	6893(2)	4634(1)	6635(1)	21
N1	5166(2)	6315(1)	4970(1)	20
C1	6396(2)	6742(1)	5548(1)	18
C2	7972(2)	6083(1)	6021(1)	18
C3	9144(2)	5411(1)	5433(1)	21
C4	7441(2)	4887(1)	4932(1)	21
C5	7742(3)	3966(1)	4658(1)	31
C6	6144(3)	3546(1)	4160(1)	40
C7	4215(3)	4046(1)	3935(1)	37
C8	3863(3)	4957(1)	4211(1)	28
C9	5468(2)	5368(1)	4711(1)	20
C10	9718(3)	6658(1)	6494(1)	27
C11	6454(2)	5559(1)	6627(1)	19
C12	5723(2)	3974(1)	7185(1)	21
C13	6491(3)	4187(1)	8023(1)	33
C14	6602(3)	3017(1)	6911(1)	33
C15	3159(3)	4034(1)	7078(1)	37

Table 3. Bond lengths [Å] and angles [°] for 23162.

O1-C1 1.2288(15) O2-C11 1.2036(16) O3-C11
1.3292(15) O3-C12 1.4824(15) N1-C1 1.3540(16)
N1-C9 1.4150(16) N1-H1 0.888(13) C1-C2
1.5353(17) C2-C3 1.5338(17) C2-C10 1.5315(18)
C2-C11 1.5435(17) C3-C4 1.5035(19) C3-H31
0.976(16) C3-H32 0.999(16) C4-C5 1.3889(19)
C4-C9 1.3938(18) C5-C6 1.390(2) C5-H51
0.947(18) C6-C7 1.387(3) C6-H61 1.00(2) C7-C8
1.382(2) C7-H71 0.96(2) C8-C9 1.3907(19)
C8-H81 0.931(19) C10-H101 0.973(18) C10-
H102 0.956(19) C10-H103 0.986(18) C12-
C13 1.5115(19) C12-C14 1.5170(19) C12-
C15 1.519(2) C13-H131 1.007(19) C13-
H132 0.989(19) C13-H133 1.00(2) C14-H141
0.975(19) C14-H142 1.01(2) C14-H143 0.95(2) C15-
H151 0.96(2) C15-H152 0.98(2) C15-H153
0.96(2)

C11-O3-C12 122.17(10) C1-N1-C9 125.06(11) C1-N1-
H1 117.2(8) C9-N1-H1 117.6(8) N1-C1-O1
121.58(11) N1-C1-C2 115.21(10) O1-C1-C2
123.20(11) C1-C2-C3 107.99(10) C1-C2-C10
110.71(11) C3-C2-C10 111.29(11) C1-C2-C11
106.56(10) C3-C2-C11 113.03(10) C10-C2-C11
107.18(10) C2-C3-C4 111.60(10) C2-C3-H31 112.0(9)
C4-C3-H31 110.5(9) C2-C3-H32 106.0(9) C4-C3-H32
111.4(9) H31-C3-H32 105.2(13) C3-C4-C5
124.15(13) C3-C4-C9 117.62(11) C5-C4-C9
118.21(13) C4-C5-C6 120.81(15) C4-C5-H51
117.0(11) C6-C5-H51 122.2(11) C5-C6-C7
120.00(14) C5-C6-H61 118.7(12) C7-C6-H61
121.3(12) C6-C7-C8 120.18(15) C6-C7-H71
120.3(11) C8-C7-H71 119.5(11) C7-C8-C9
119.31(15) C7-C8-H81 121.2(11) C9-C8-H81
119.4(11) N1-C9-C4 118.78(12) N1-C9-C8
119.67(12) C4-C9-C8 121.47(12) C2-C10-H101
108.4(10) C2-C10-H102 111.7(11) H101-C10-H102
107.4(14) C2-C10-H103 109.9(10) H101-C10-H103
112.8(14) H102-C10-H103 106.7(14) C2-C11-O3
111.32(11) C2-C11-O2 122.50(11) O3-C11-O2
126.13(12) O3-C12-C13 108.71(11) O3-C12-C14
102.17(10) C13-C12-C14 111.04(12) O3-C12-C15

110.49(11)	C13-C12-C15	113.32(13)	C14-C12-C15
110.52(13)	C12-C13-H131	106.7(10)	C12-C13-H132
110.8(10)	H131-C13-H132	113.2(14)	C12-C13-H133
110.9(10)	H131-C13-H133	108.0(15)	H132-C13-H133
107.2(16)	C12-C14-H141	109.6(11)	C12-C14-H142
109.1(11)	H141-C14-H142	109.8(16)	C12-C14-H143
110.0(11)	H141-C14-H143	111.1(16)	H142-C14-H143
107.2(16)	C12-C15-H151	105.9(12)	C12-C15-H152
114.5(12)	H151-C15-H152	109.5(16)	C12-C15-H153
107.9(12)	H151-C15-H153	110.7(16)	H152-C15-H153
108.3(16)			

Symmetry transformations used to generate equivalent atoms:

Table 4. Anisotropic displacement parameters ($Å^2 x 10^3$) for 23162. The anisotropic displacement factor exponent takes the form: $-2\pi^2[h^2 a^{*2}U^{11} + ... + 2 h k a^* b^* U^{12}]$

	U^{11}	U^{22}	U^{33}	U^{23}	U^{13}	U^{12}
O1	26(1)	14(1)	31(1)	0(1)	2(1)	1(1)
O2	35(1)	23(1)	36(1)	1(1)	15(1)	6(1)
O3	28(1)	16(1)	19(1)	4(1)	4(1)	1(1)
N1	21(1)	17(1)	22(1)	1(1)	-2(1)	4(1)
C1	17(1)	16(1)	20(1)	2(1)	4(1)	0(1)
C2	19(1)	14(1)	21(1)	1(1)	0(1)	2(1)
C3	19(1)	19(1)	24(1)	3(1)	4(1)	3(1)
C4	25(1)	19(1)	19(1)	1(1)	7(1)	1(1)
C5	37(1)	22(1)	32(1)	-3(1)	9(1)	5(1)
C6	52(1)	24(1)	44(1)	-14(1)	8(1)	0(1)
C7	41(1)	34(1)	36(1)	-16(1)	3(1)	-10(1)
C8	26(1)	31(1)	25(1)	-4(1)	3(1)	-3(1)
C9	24(1)	17(1)	18(1)	-1(1)	6(1)	-1(1)
C10	24(1)	26(1)	32(1)	1(1)	-5(1)	-2(1)
C11	21(1)	17(1)	19(1)	0(1)	-1(1)	2(1)
C12	24(1)	18(1)	22(1)	5(1)	1(1)	-3(1)
C13	40(1)	36(1)	21(1)	6(1)	1(1)	-5(1)
C14	44(1)	19(1)	37(1)	5(1)	6(1)	0(1)
C15	26(1)	32(1)	54(1)	12(1)	-6(1)	-5(1)

Table 5. Hydrogen coordinates (x 10^4) and isotropic displacement parameters ($Å^2$x 10^3) for 23162.

	x	y	z	U(eq)
H31	10160(30)	4966(11)	5700(9)	25
H32	10160(30)	5818(11)	5100(9)	25
H51	9070(30)	3644(12)	4825(10)	37
H61	6430(40)	2890(14)	3965(11)	48
H71	3130(30)	3766(13)	3574(12)	45
H81	2610(30)	5310(12)	4050(10)	33
H101	10620(30)	6220(12)	6810(10)	32
H102	9000(30)	7089(13)	6853(10)	32
H103	10650(30)	7044(12)	6131(10)	32
H131	5810(30)	3681(13)	8372(11)	39
H132	6050(40)	4836(14)	8180(10)	39
H133	8180(30)	4146(13)	8068(10)	39
H141	5940(30)	2517(13)	7237(11)	40
H142	8310(40)	3005(14)	6961(11)	40
H143	6250(40)	2925(13)	6367(12)	40
H151	2530(40)	3523(14)	7386(11)	45
H152	2480(40)	4636(15)	7248(12)	45
H153	2830(30)	3947(14)	6523(13)	45
H1	4150(30)	6669(9)	4713(8)	26(2)

Table 6. Torsion angles [°] for 23162.

C12-O3-C11-O2	0.27(19)	C12-O3-C11-C2	177.89(10)	C11-O3-
C12-C13	-68.20(14)	C11-O3-C12-C14	174.37(11)	C11-O3-
C12-C15	56.75(16)	C9-N1-C1-O1	170.74(12)	C9-N1-
C1-C2	-9.18(17)	C1-N1-C9-C4	-14.26(18)	C1-N1-
C9-C8	169.09(12)	O1-C1-C2-C3	-137.14(12)	O1-C1-
C2-C10	-15.09(17)	O1-C1-C2-C11	101.14(13)	N1-C1-
C2-C3	42.78(14)	N1-C1-C2-C10	164.83(11)	N1-C1-
C2-C11	-78.94(13)	C1-C2-C3-C4	-54.85(13)	C10-C2-
C3-C4	-176.56(11)	C11-C2-C3-C4	62.77(13)	C1-C2-
C11-O2	-48.96(15)	C1-C2-C11-O3	133.32(10)	C3-C2-
C11-O2	-167.43(12)	C3-C2-C11-O3	14.85(14)	C10-C2-
C11-O2	69.60(15)	C10-C2-C11-O3	-108.12(12)	C2-C3-
C4-C5	-146.60(13)	C2-C3-C4-C9	35.32(15)	C3-C4-
C5-C6	-176.50(14)	C9-C4-C5-C6	1.6(2)	C3-C4-C9-N1
-0.27(17)	C3-C4-C9-C8	176.32(12)	C5-C4-C9-N1	-
178.47(12)	C5-C4-C9-C8	-1.88(19)	C4-C5-C6-C7	-0.3(2)
C5-C6-C7-C8	-0.7(3)	C6-C7-C8-C9	0.5(2)	C7-C8-C9-N1
177.45(13)	C7-C8-C9-C4	0.9(2)		

Symmetry transformations used to generate equivalent atoms:
